# Copper-Catalyzed Petasis-Type Reaction Enables Efficient Synthesis of C1-Substituted Tetrahydro-β-carbolines

**DOI:** 10.1021/acs.orglett.6c01466

**Published:** 2026-04-27

**Authors:** Kai Cheng, Jialong Li, Shucai Cai, Jianyi Wang, Lixin Liang, Rongbiao Tong

**Affiliations:** † School of Medicine, 12664 Guangxi University, Nanning 530004, China; ‡ Department of Chemistry, 58207 The Hong Kong University of Science and Technology, Clear Water Bay, Kowloon, Hong Kong 999077, China

## Abstract

Petasis and Petasis-type reactions represent a powerful transformation for constructing a wide array of functionalized amines from simple amines, aldehydes, and organoborons. An α-oxygen or α-nitrogen directing group on the aldehyde component is typically required to facilitate carbon transfer from organoboron to the iminium ion. Herein, we report the first Petasis-type reaction of previously unexplored *N*-alkyl dihydro-β-carbolinium (DHβC) ions with aryl/alkenyl boronates through novel copper catalysis. The efficiency and utility of this protocol were demonstrated by a broad substrate scope (51 examples, up to 99% yield), excellent scalability (gram scale), and versatile transformations of products. Mechanistic studies suggest that this process proceeds via a novel mechanism involving a copper-mediated intramolecular single-electron transfer (SET). This novel Petasis-type reaction not only significantly broadens the scope of Petasis reactions but also reveals new mechanistic pathways.

## Graphical abstract

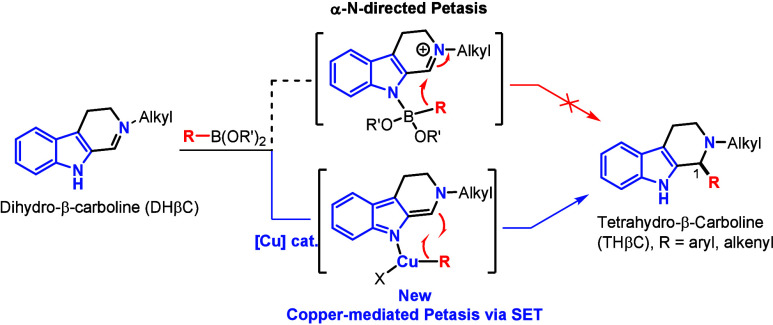

The Petasis reaction, also known as the Petasis-borono-Mannich reaction, is a versatile three-component coupling reaction discovered by Petasis in 1993. It combines an amine, a carbonyl compound (typically an α-hydroxy aldehyde or α-keto acid), and an organoboron reagent (such as vinyl- or arylboronic acids or esters) to efficiently synthesize substituted amines, including β-amino alcohol, allylamines, α-aryl glycines, and other functionalized α-amino acid derivatives (Scheme 1A).^1^ This mild, atom-economic transformation tolerates a broad range of functional groups, requires no anhydrous or inert conditions, and often proceeds with high stereocontrol when chiral substrates are employed.^2^ Its broad substrate scope and utility in accessing biologically relevant molecules have made it a valuable tool in organic synthesis, combinatorial chemistry, and drug discovery.^3^

**Scheme 1:**
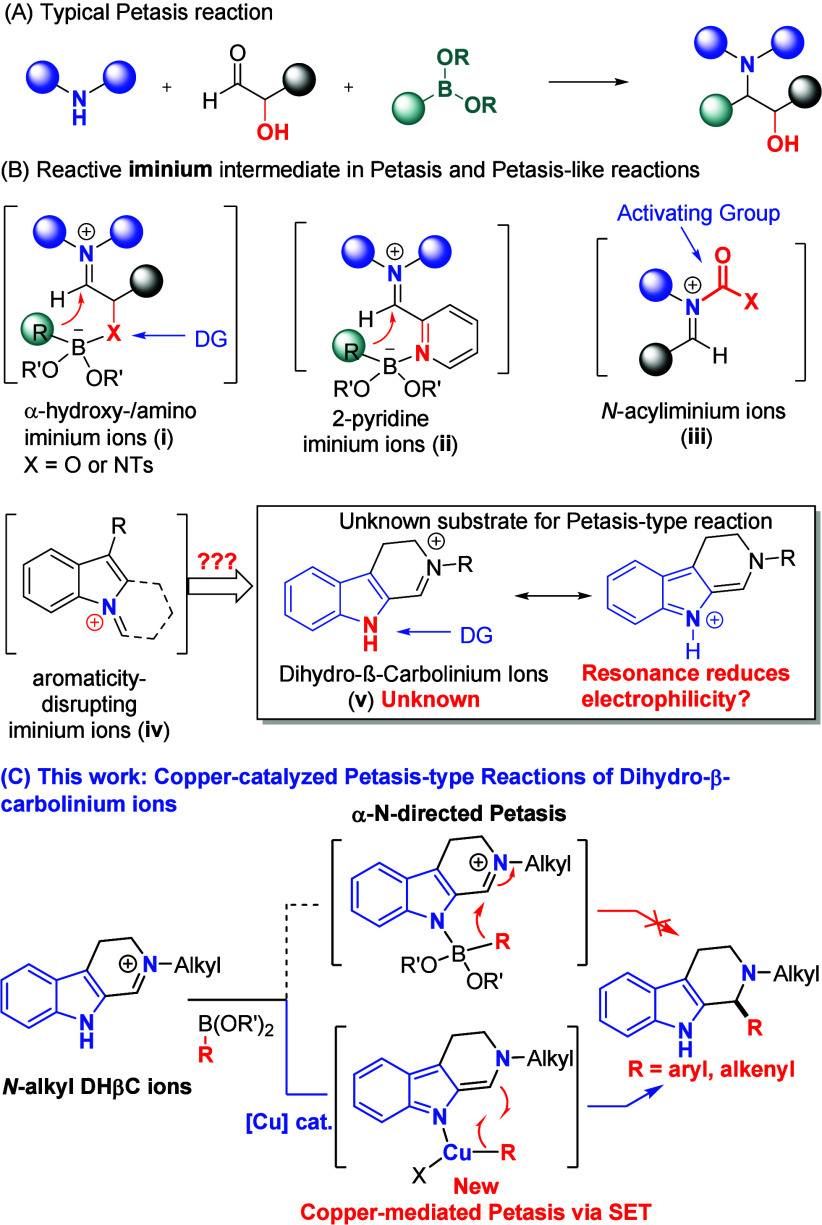
Previously Reported Petasis and Petasis-Type Reactions and This Work

Mechanistically, Petasis and Petasis-type reactions require a directing group on the carbonyl component (usually an aldehyde, such as α-hydroxy aldehyde, salicylaldehyde, and 2-pyridinecarbaldehyde^4^) (Scheme 1B) to facilitate the transfer of the carbon group from the boronic acid through the formation of an “ate” boronate complex (**i** and **ii**) (Scheme 1B). This “ate” complex permits the intramolecular transfer in a five-membered ring fashion.^2^ Recently, non-directed Petasis-type reactions have been developed^5^ through (i) using more reactive vinyl boronic acids or “pre-activated boron species”, such as trifluoroborate salts,^6^ and/or (ii) generating highly electrophilic iminium ion (**iii**) (e.g., *N*-acyliminium ions, *N*-tosyliminium ions, and *N*-nosyliminium ions).^7^ More recently, the Stoltz group reported an elegant non-directed Petasis-type reaction that exploits the novel aromaticity-disrupting iminium ion (**iv**) derived from unique α-hydroxy indoles and highly reactive trifluoroborate salts.^8^ Inspired by Stoltz’s elegant work, we envisioned to employ the versatile dihydro-β-carbolinium (DHβC) ions^9^ (**v**) for the Petasis-type reaction, which, to the best of our knowledge, has not been previously reported (Scheme 1C). This gap may be attributed to the reduced electrophilicity of these ions arising from the resonance stabilization by electron-rich indole.

Nevertheless, we postulate that this unexplored transformation would substantially expand the scope of competent electrophiles in the Petasis manifold. Herein, we disclose a novel copper-catalyzed Petasis-type reaction of *N*-alkyl DHβC ions with aryl/alkenyl boronates. Mechanistic studies reveal that the reaction proceeds via a novel mechanism involving copper-mediated intramolecular single-electron transfer (SET); moreover, indole nitrogen plays a pivotal role in activating the organometallic nucleophiles.

We began our investigations of the Petasis-type reaction using *N*-benzyl or *N*-allyl DHβC ions (**1a**/**1b**)^9a^ and different organoboron compounds **2a**–**2d** (Table 1). Without transition metal catalysis, the reaction did not occur even at reflux temperature (entries 1–4). These results were consistent with the low reactivity of the *N*-alkyl DHβC ions and arylboronates. Inspired by palladium- and copper-catalyzed addition of aryl boronate to iminium ions,^10^ we evaluated a small series of transition metal catalysts for the reaction of **1a** with aryl boronic acid **2b**. It was found that palladium, nickel, ytterbium, indium, and lanthanum did not catalyze the reaction (see Supplementary Table 3 for details). To our delight, CuI was found to effectively catalyze the reaction of **1a** and **2b** to afford product **3a** in 84% yield (entry 5). The reaction of **1a** with vinylboronic ester **2d** under otherwise identical conditions was also effective to provide coupling product **3b** in 50% yield (entry 6). Encouraged by these results, we investigated other copper salts as the catalyst (entries 7–13) and found that different copper salts have considerable effects on the reaction yield. After extensive screening of reaction parameters (shown in Supplementary Tables 1–6), the CuCl_2_-catalyzed reaction of **1b** (*N*-allyl DHβC) with aryl boronic acid **2b** gave the coupling product **3c** in a nearly quantitatively yield (99%, entry 14). For the reaction with vinylboronic ester **2d**, CuI was found to be best in the presence of *t*-BuONa in dichloromethane, which provided product **3b** in 92% isolated yield (entry 15). The difference in optimal conditions is likely due to the inherently lower reactivity of alkenyl pinacol boronates compared to aryl boronic acids. It should be noted that base was essential for the reaction because no reaction occurred even at reflux in the absence of base (entry 16).

**Table 1:**
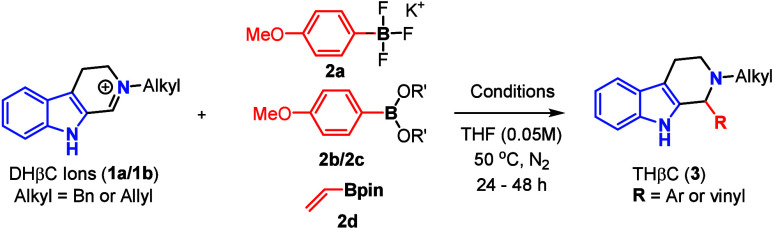
Screening of Reaction Conditions^a^

entry	alkyl	R–[B]	catalyst	base	yield (%)^b^
1^c^	**1a**/**1b**	**2a**–**2d**	N/A	N/A	N.R.^d^
2^c^	**1a**/**1b**	**2a**–**2d**	N/A	*t*BuOK	N.R.^d^
3^c^	**1a**/**1b**	**2a**–**2d**	N/A	K_2_CO_3_	N.R.^d^
4^c^	**1a**/**1b**	**2a**–**2d**	N/A	Et_3_N	N.R.^d^
5	**1a**	**2b**	CuI	Cs_2_CO_3_	84
6	**1a**	**2d**	CuI	Cs_2_CO_3_	50
7	**1a**	**2b**	CuBr	Cs_2_CO_3_	78
8	**1a**	**2b**	CuCl	Cs_2_CO_3_	20
9	**1a**	**2b**	CuCl_2_	Cs_2_CO_3_	27
10	**1a**	**2b**	Cu(OTf)_2_	Cs_2_CO_3_	83
11	**1a**	**2b**	CuOAc	Cs_2_CO_3_	6
12	**1a**	**2b**	CuSO_4_	Cs_2_CO_3_	N.R.^d^
13	**1a**	**2b**	CuBr_2_	Cs_2_CO_3_	97^e^
14	**1b**	**2b**	CuBr_2_	Cs_2_CO_3_	99^e^
15^f^	**1a**	**2d**	CuI	*t*BuONa	92^e^
16^c^	**1a**/**1b**	**2b**	CuI	N/A	N.R.^d^

a Reaction conditions: DHβC ion **1** (0.2 mmol), organoboron reagents (0.4 mmol), base (0.4 mmol), transition metal catalyst (0.04 mmol), solvent (4.0 mL, 0.05 M), 5 Å molecular sieves (100 mg), and 50 °C.

b The yield was determined by NMR of crude material using CH_2_Br_2_ as an internal standard.

c Under reflux.

d No reaction.

e Isolated yield.

f CH_2_Cl_2_ as the reaction solvent.

With the optimized condition in hand, we next explored the substrate scope (Table 2). First, the scope of arylboronic acids was investigated (Table 2A). It was found that substitution on the *para*, *meta*, and *ortho* positions of the arylboronic acids did not significantly affect the arylation (**3a**, **3d**, and **3e**, 96–99%). Monosubstituted arylboronic acids with electron-donating substituents, such as methyl (*meta*- and *para*-Me, **3g** and **3h**), *tert*-butyl (*t*Bu, **3i**), ethyl (Et, **3j**,), hydroxymethyl (CH_2_OH, **3k**), and phenoxy (OPh, **3l**), proved efficient (63–98% yields) in the desired Petasis-type coupling. Moreover, electron-deficient arylboronic acids with electron-withdrawing substituents, such as halides (**3m**, **3n**, and **3o**), trifluoromethyl (CF_3_, **3p**), cyano (CN, **3q**), nitro (NO_2_, **3r**), aldehyde (**3s**), and ester (**3t**), were also well-tolerated (57–82% yields). This wide tolerance overcomes the previously noted limitation that Petasis-type reactions with electron-deficient arylboronic acids remain highly constrained. Notably, substrate **3u** (72% yield), featuring a terminal alkene, is synthetically useful for various subsequent transformations, such as cross-coupling, epoxidation, and hydroboration–oxidation, by using its vinyl moiety. Furthermore, 4-phenyl-substituted (**3v**, 74%) and 3,5-disubstituted (**3w**, 84%) groups and naphylboronic acid (**3y**, 74%) successfully provided the corresponding products. Thiophen-2-yl boronic acid could also be employed (**3z**, 36%), demonstrating the applicability of this protocol to heteroarylboronic acids. Next, various *N*-alkyl groups on nitrogen of the DHβC ions were evaluated (Table 2B). Substituted benzyl groups with either electron-donating (*ortho*-, *meta*-, and *para*-Me, **3aa**–**3ac**) or electron-withdrawing (bromo and nitro, **3ad**–**3ag**) substituents consistently delivered the products with high yields (83–99%). Furthermore, the *N*-methyl DHβC ion proved to be an effective substrate for the coupling (**3ai**, 90%). We then turned our attention to *N*-allyl groups since it could not only serve as a protecting group of amines but also provide handles for further functionalization. A range of *N*-allyl groups, including 1,1- and 1,2-disubstituted and even trisubstituted alkenes (**3aj**–**3am**), were found to be compatible with the reaction conditions. It was noted that alkenyl iodide (**3al**) or conjugate ester (**3am**) as part of the allyl group resulted in poor yields (28–34%), which might be attributed competing copper-catalyzed side reactions (i.e., Michael addition or oxidative addition). Different DHβC ions were also examined (Table 2C). Interestingly, the remote substituent at C5 considerably influenced the reaction yield: 5-methoxy on the indole arene ring yielded **3ao** in 80%, and 5-bromo and 5-chloro led to **3ap** and **3aq** with only 37% and 40%, respectively. The lower yields for halogen-substituted DHβC ions (**3ap** and **3aq**) are likely due to the electron-withdrawing effect of halogens. C1-methylated DHβC ions failed to undergo a Petasis-type reaction probably due to the steric hindrance. Vinyl boronic ester could also react with a range of *N*-alkyl DHβC ions to C1-vinyl THβCs (**3b** and **3as**–**3ay**) in good to excellent yields (73–92%), which could serve as versatile intermediates for synthesis of indole alkaloids.^11^ When styrylboronic pinacol ester was employed, corresponding C1-styryl product **3az** was obtained in 95% yield. Finally, the synthetic utility of C1-aryl THβC **3f** was demonstrated (Table 2E). It was successfully transformed into indole **4** (67% yield) via an iodine-mediated oxidative ring opening^12^ to spirooxindoles **5** and **5′** (85% total yield and 5:1 diastereomer ratio) via an oxidative rearrangement^13^ and to ring-enlargement^14^ product **6** (80% yield) with methyl propiolate, highlighting its versatility in organic synthesis and drug discovery.

**Table 2:**
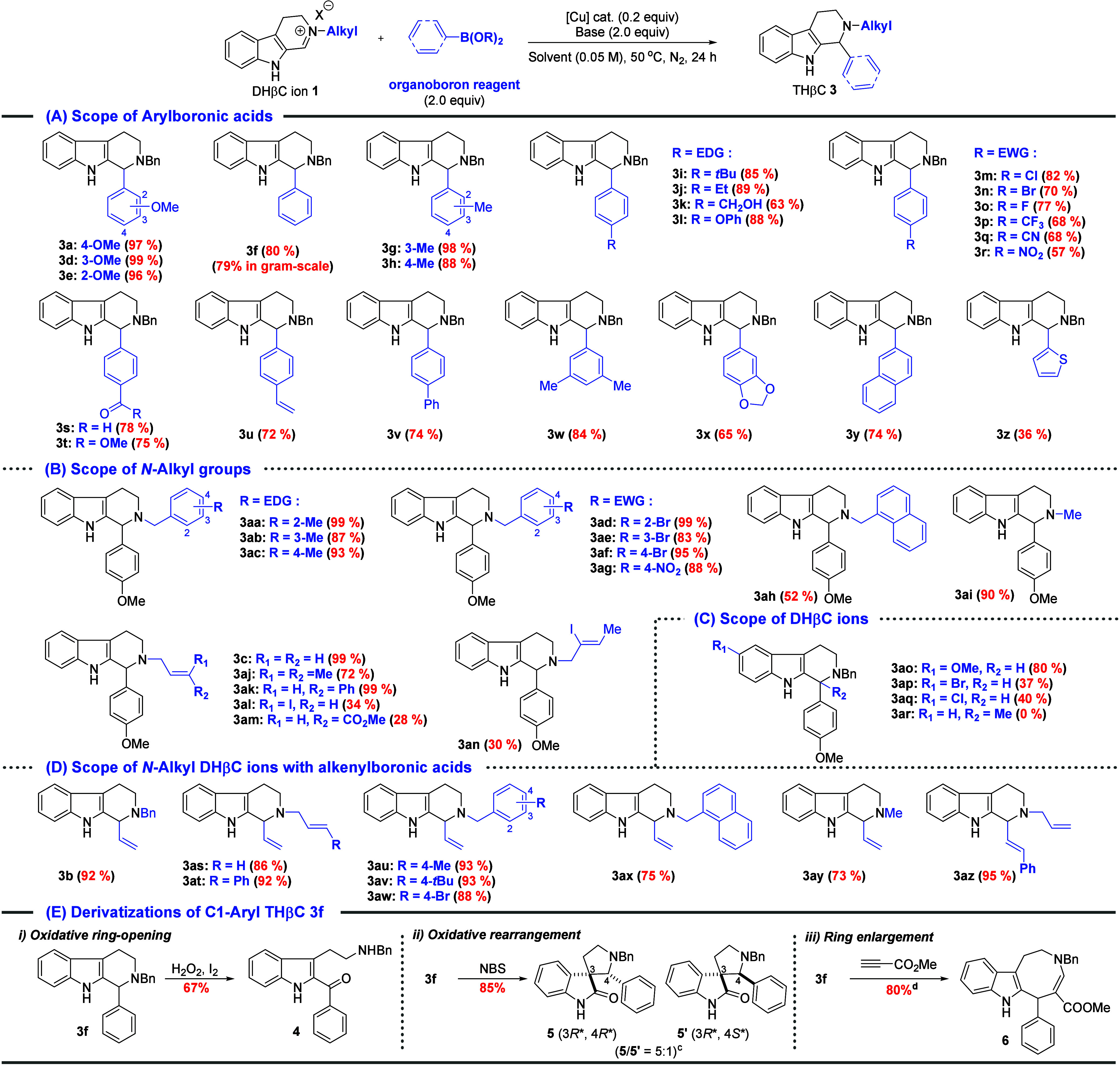
Substrate Scope of the Petasis-Type Reaction of *N*-Alkyl DHβC Ions and Derivatizations of C1-Aryl THβC^a^ ^,^ ^b^

a Reaction conditions for C1-arylation: **1** (DHβC ion, 0.2 mmol), arylboronic acid (0.4 mmol), Cs_2_CO_3_ (0.4 mmol), CuBr_2_ (0.08 mmol), THF (4.0 mL, 0.05 M), 5 Å molecular sieves (100 mg), 50 °C, and 24 h.

b Reaction conditions for C1-alkenylation: **1** (DHβC ion, 0.2 mmol), alkenylboronic acid ester (0.4 mmol), *t*BuONa (0.4 mmol), CuI (0.08 mmol), CH_2_Cl_2_ (4.0 mL, 0.05 M), 5 Å molecular sieves (100 mg), 50 °C, and 24 h.

c Mixture of inseparable diastereomers.

d The yield was based on recovered starting materials.

Despite few previous works reporting nickel- and copper-catalyzed Petasis-type reactions,^15^ the proposed mechanisms differ substantially in the role of metal catalyst and the way of aryl migration, as exemplified by unusual S_N_2,^10d^ cuprate complexation,^10e^ and intramolecular cuprate addition.^10f^ We attempted to elucidate the mechanism of our copper-catalyzed reaction. First, control experiments were designed (Scheme 2A) to probe the role of indole’s N–H directing effect. It was found that indole’s N–H was essential because no reaction occurred even at an elevated temperature when indole nitrogen was alkylated/tosylated. Since the base (Cs_2_CO_3_ or *t*BuONa) was needed to promote the rection, we investigated the base’s effect using nuclear magnetic resonance (NMR) spectroscopy and high-resolution mass spectrometry, both of which suggested the formation of a new enamine species (**int-1b**; see the Supporting Information for details) through deprotonation of indole N–H. This finding was consistent with the essential role of indole N–H as a directing group. It was noted that the copper catalyst was demonstrated to be critical to the success of this reaction in our initial screening (Table 1), and the effect of the copper catalyst on the reaction mechanism was investigated: nucleophilic addition or radical coupling. To elucidate the copper’s role, radical inhibition experiments were designed and conducted (Scheme 2B). It was found that the reaction was significantly suppressed by both radical scavengers 2,2,6,6-tetramethyl-1-piperidinyloxy (TEMPO) or butylated hydroxytoluene (BHT), which suggested that a primary radical pathway was favored probably through a single-electron transfer (SET) process. While more experiments are needed to fully uncover the detailed mechanism, we could propose the outline of a plausible mechanism, as depicted in Scheme 2C. The base would deprotonate indole to generate the imine–enamine intermediate, which complexed with organocuprate derived from transmetalation of organoborate to provide the activated organocuprate species.^10f,16^ The “ate complex” transferred the carbon nucleophile from copper to C1 (enamine or iminium form) through the SET process. This mechanistic profile is consistent with all of the findings reported in this work.

**Scheme 2:**
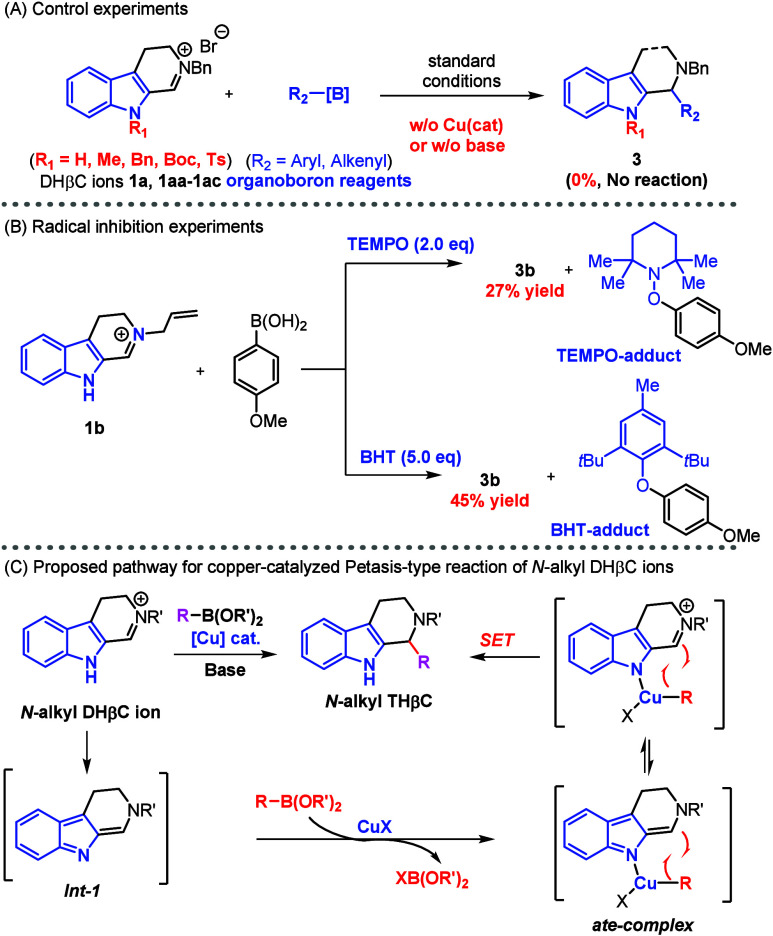
Previously Reported Metal-Catalyzed Petasis-Type Reactions, Control Experiments, and Proposed Mechanism of Our Copper-Catalyzed Reaction

In conclusion, we have successfully developed the first Petasis-type reaction of previously unexplored *N*-alkyl DHβC ions. This work establishes a distinct and efficient pathway for the arylation and alkenylation of these atypical low electrophilic iminium ions through copper-catalyzed coupling with aryl- and alkenylboronic reagents, which proceeds via a novel copper-mediated intramolecular SET mechanism. A key mechanistic insight revealed in the pathways is the pivotal role of indole nitrogen in activating organometallic nucleophiles. Overall, this study not only significantly expands the structural and mechanistic scope of the classic Petasis reaction but also provides a versatile and efficient platform for the modular assembly of privileged β-carboline scaffolds, thereby opening new avenues for synthetic methodology development.

## Supplementary Material



## Data Availability

The data underlying this study are available in the published article and its Supporting Information.

## Notes

Experimental procedures, characterization data, and NMR spectra for all new compounds (PDF)
