# Supplementary material for: Copper-Catalyzed Petasis-Type Reaction Enables Efficient Synthesis of C1-Substituted Tetrahydro-β-carbolines
Source: Org Lett. 2026 Apr 27;28(18):5870–5. doi: 10.1021/acs.orglett.6c01466 (PMC13162319; doi:10.1021/acs.orglett.6c01466)

Supporting Information

Copper-catalyzed Petasis-Type Reaction Enables Efficient Synthesis of C1-Substituted Tetrahydro-beta-carbolines

Kai Cheng[a], Jialong Li[a], Shucai Cai[a], Jianyi Wang[a], Lixin Liang *[a], Rongbiao Tong *[b]

[a] School of Medicine, Guangxi University, Nanning, 530004, P. R. China

[b] Department of Chemistry, The Hong Kong University of Science and Technology, Hong Kong SAR, China

Table of Contents

1. General Information 2

2. Screening of Reaction Conditions 3

3. Experiment Procedure and Characterization Data 9

3.1 Preparation of dihydro-β-carboline 9

3.2 Preparation of N-alkyl dihydro-β-carbolinium (DHβC) Ions 9

3.3 Copper-catalyzed Petasis-type reaction of N-alkyl DHβC ions 9

3.4 Derivatizations of C1-Aryl THβC 3f 26

3.5 Mechanistic studies 28

4. References 34

5. Copies of NMR Spectrums 35

1. General Information

Reactions were carried out in oven or flame-dried glassware under argon atmosphere, unless otherwise noted. Tetrahydrofuran (THF) and toluene were freshly distilled before use from sodium using benzophenone as an indicator. Dichloromethane was freshly distilled before use from calcium hydride (CaH_2). All other solvents were dried over 5Å molecular sieves. Solvents, catalysts and reagents were used as received from commercial suppliers (Energy Chemical) without prior purification for workup, extraction and flash column chromatography. Reactions were monitored by thin-layer chromatography (TLC, 0.25 mm) on pre-coated silica gel plates. Flash chromatography was performed with silica gel 60 (particle size 0.040–0.062 mm). ^{1}H- and ^{13}C-NMR spectra were recorded on Bruker AVIII-400 spectrometer (400 MHz for ^{1}H, 100 MHz for ^{13}C), Bruker AVANCE III 500 MHz spectrometer (500 MHz for ^{1}H, 126 MHz for ^{13}C, 565 MHz for ^{19}F) and Bruker AVANCE III 600 MHz spectrometer (600 MHz for ^{1}H, 151 MHz for ^{13}C). Chemical shifts are reported in parts per million (ppm) as values relative to the internal chloroform (7.26 ppm for ^{1}H and 77.16 ppm for ^{13}C) or dimethyl sulfoxide (2.50 ppm for ^{1}H and 39.52 ppm for ^{13}C). Abbreviations for signal coupling are as follows: s, singlet; d, doublet; t, triplet; q, quartet; m, multiplet. High-resolution mass spectrometry (HRMS) was recorded on an Agilent 6545 Q-TOF mass spectrometer (SN: SG22202E101), equipped with an ESI source (Santa Clara, CA, USA), Q-TOF (AB SCIEX X500R with ESI source, and Agilent 7250 with EI source). UV-Vis spectrometry was measured on Shimadzu UV-2600i UV-Vis spectrophotometer.

2. Screening of Reaction Conditions

Supplementary Table 1. Screening of solvents for Petasis-type coupling of 3,4-dihydro-β-carboline (DHβC) ion **1a** with arylboronic acid [a]

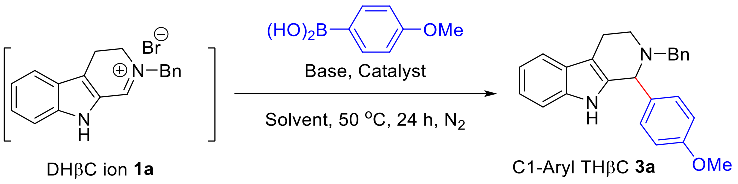

Entry	Catalyst	Base [b]	Solvent [c]	Yield (%) [d]
1	CuI	*t*BuOK	**THF**	**68**
2	CuI	*t*BuOK	EtOAc	33
3	CuI	*t*BuOK	MeCN	50
4	CuI	*t*BuOK	Toluene	60
5	CuI	*t*BuOK	DMSO	< 5
6	CuI	*t*BuOK	HFIP	N.D [e]
7	CuI	*t*BuOK	DCM	< 20

[a] Condition: **1a** (DHβC ion, 0.2 mmol), arylboronic acids (0.4 mmol), base (0.4 mmol), catalyst (0.04 mmol), solvent (4.0 mL, 0.05 M), 5 Å molecular sieves (100 mg), 50 °C, 24 h under the nitrogen atmosphere. [b] *t*BuOK = potassium *tert*-butoxide. [c] THF = tetrahydrofuran; EtOAc = ethyl acetate; MeCN = acetonitrile; DMSO = dimethyl sulfoxide; HFIP = hexafluoroisopropanol; DCM = dichloromethane. [d] Yield of **3a** was determined by NMR analysis of the crude reaction mixture with CH_2Br_2 as the internal standard, unless otherwise specified. [e] Not detected.

Supplementary Table 2. Screening of bases for Petasis-type coupling of DHβC Ion **1a** with arylboronic acid [a]

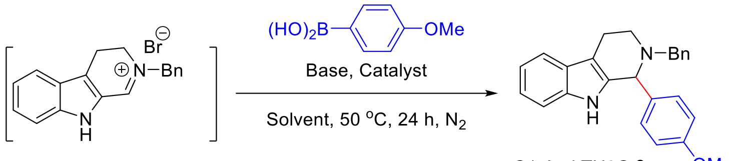

Entry	Catalyst	Base [b]	Solvent [c]	Yield (%) [d]
1	CuI	*t*BuOK	THF	68
2	CuI	*t*BuONa	THF	39
3	CuI	**Cs_2CO_3**	THF	**84**
4	CuI	TMSOK	THF	63
5	CuI	DMAP	THF	0
6	CuI	K_2CO_3	THF	Trace
7	CuI	Et_3N	THF	N.D [e]
8	CuI	DIPEA	THF	N.D [e]
9	CuI	TMEDA	THF	N.D [e]

[a] Condition: **1a** (DHβC ion, 0.2 mmol), arylboronic acids (0.4 mmol), base (0.4 mmol), catalyst (0.04 mmol), solvent (4.0 mL, 0.05 M), 5 Å molecular sieves (100 mg), 50 °C, 24 h under the nitrogen atmosphere. [b] *t*BuOK = potassium *tert*-butoxide; *t*BuONa = sodium *tert*-butoxide; TMSOK = Potassium trimethylsilanolate; DMAP = 4-Dimethylaminopyridine; DIPEA = N,N-Diisopropylethylamine; TMEDA = Tetramethylethylenediamine. [c] THF = tetrahydrofuran. [d] Yield of **3a** was determined by NMR analysis of the crude reaction mixture with CH_2Br_2 as the internal standard, unless otherwise specified. [e] Not detected.

Supplementary Table 3. Screening of catalysts for Petasis-type coupling of DHβC Ion **1a** with arylboronic acid [a]

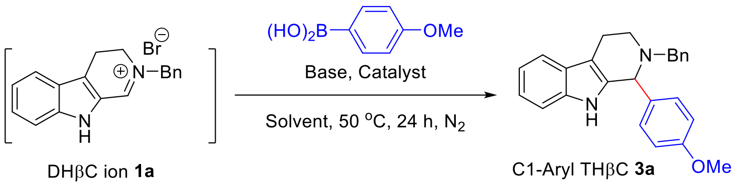

Entry	Catalyst	Base	Solvent [b]	Yield (%) [c]
1	CuI	Cs_2CO_3	THF	84
2	$Pd(OAc)_2$	Cs_2CO_3	THF	Trace
3	$Pd(PPh)_4$	Cs_2CO_3	THF	Trace
4	$PdCl_2(PPh_3)_2$	Cs_2CO_3	THF	Trace
5	$NiCl_2$(dppp)	Cs_2CO_3	THF	Trace
6	$Yb(OTf)_3$	Cs_2CO_3	THF	N.D[d]
7	$In(OTf)_3$	Cs_2CO_3	THF	N.D[d]
8	$La(OTf)_3$	Cs_2CO_3	THF	N.D[d]
9	CuBr	Cs_2CO_3	THF	78
10	CuCl	Cs_2CO_3	THF	20
11	$CuCl_2$	Cs_2CO_3	THF	27
12	$Cu(OTf)_2$	Cs_2CO_3	THF	83
13	CuOAc	Cs_2CO_3	THF	6
14	$CuSO_4$	Cs_2CO_3	THF	N.D[d]
15	**$CuBr_2$**	Cs_2CO_3	THF	**97**

[a] Condition: **1a** (DHβC ion, 0.2 mmol), arylboronic acids (0.4 mmol), base (0.4 mmol), catalyst (0.04 mmol), solvent (4.0 mL, 0.05 M), 5 Å molecular sieves (100 mg), 50 ºC, 24 h under the nitrogen atmosphere. [b] THF = tetrahydrofuran. [c] Yield of **3a** was determined by NMR analysis of the crude reaction mixture with CH_2Br_2 as the internal standard, unless otherwise specified. [d] Not detected.

Supplementary Table 4. Screening of catalysts for Petasis-type coupling of DHβC Ion **1a** with vinyl boronic ester [a]

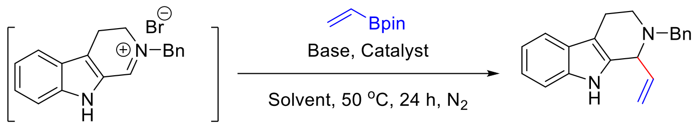

Entry	Catalyst	Base [b]	Solvent [c]	Yield (%) [d]
1	$CuBr_2$	Cs_2CO_3	THF	Trace
2	$CuBr_2$	*t*BuONa	DCM	50
3	CuBr	*t*BuONa	DCM	28
4	CuCl	*t*BuONa	DCM	55
5	$CuCl_2$	*t*BuONa	DCM	30
6	CuOAc	*t*BuONa	DCM	10
7	$Cu(ACN)_4BF_4$	*t*BuONa	DCM	60
8	$CuSO_4$	*t*BuONa	DCM	N.D[e]
**9**	**CuI**	***t*BuONa**	**DCM**	**90**

[a] Condition: **1a** (DHβC ion, 0.2 mmol), vinyl boronic ester (0.4 mmol), base (0.4 mmol), catalyst (0.04 mmol), solvent (4.0 mL, 0.05 M), 5 Å molecular sieves (100 mg), 50 °C, 24 h under the nitrogen atmosphere. [b] *t*BuONa = sodium *tert*-butoxide. [c] THF = tetrahydrofuran; DCM = dichloromethane. [d] Yield of **3b** was determined by NMR analysis of the crude reaction mixture with CH_2Br_2 as the internal standard, unless otherwise specified. [e] Not detected.

Supplementary Table 5. Screening of solvent for Petasis-type coupling of DHβC Ion **1a** with vinyl boronic ester [a]

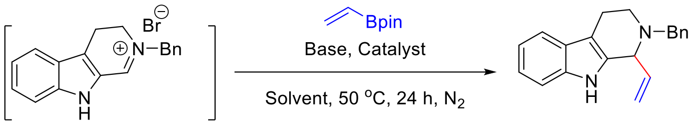

Entry	Catalyst	Base [b]	Solvent [c]	Yield (%) [d]
1	**CuI**	***t*BuONa**	**DCM**	**90**
2	CuI	*t*BuONa	Toluene	62
3	CuI	*t*BuONa	THF	50
4	CuI	*t*BuONa	MeCN	Trace
5	CuI	*t*BuONa	DCE	70
6	CuI	*t*BuONa	MeOH	Trace
7	CuI	*t*BuONa	EtOAc	Trace

[a] Condition: **1a** (DHβC ion, 0.2 mmol), vinyl boronic ester (0.4 mmol), base (0.4 mmol), catalyst (0.04 mmol), solvent (4.0 mL, 0.05 M), 5 Å molecular sieves (100 mg), 50 °C, 24 h under the nitrogen atmosphere. [b] *t*BuONa = sodium *tert*-butoxide. [c] THF = tetrahydrofuran; DCM = dichloromethane; EtOAc = ethyl acetate; MeCN = acetonitrile. [d] Yield of **3b** was determined by NMR analysis of the crude reaction mixture with CH_2Br_2 as the internal standard, unless otherwise specified.

Supplementary Table 6. Screening of bases for Petasis-type coupling of DHβC Ion **1a** with vinyl boronic ester [a]

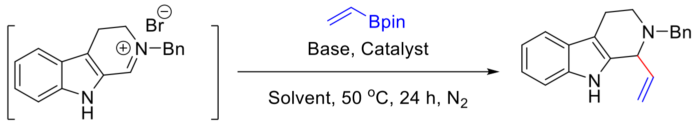

DHβC ion **1a** C1-Vinyl THβC **3b**

Entry	Catalyst	Base [b]	Solvent [c]	Yield (%) [d]
1	**CuI**	***t*BuONa**	**DCM**	**90**
2	CuI	*t*BuOK	DCM	50
3	CuI	*t*BuOLi	DCM	Trace
4	CuI	TMSOK	DCM	70
5	CuI	Cs_2CO_3	DCM	30
6	CuI	Et_3N	DCM	N.D[e]
7	CuI	Na_2CO_3	DCM	N.D[e]
8	CuI	K_2CO_3	DCM	N.D[e]
9	CuI	DMAP	DCM	N.D[e]

[a] Condition: **1a** (DHβC ion, 0.2 mmol), vinyl boronic ester (0.4 mmol), base (0.4 mmol), catalyst (0.04 mmol), solvent (4.0 mL, 0.05 M), 5 Å molecular sieves (100 mg), 50 °C, 24 h under the nitrogen atmosphere. [b] tBuOK = potassium tert-butoxide; tBuONa = sodium tert-butoxide; tBuOLi = lithium tert-butoxide; TMSOK = Potassium trimethylsilanolate; Et_3N = triethylamine DMAP = 4-Dimethylaminopyridine. [c] DCM = dichloromethane. [d] Yield of **3b** was determined by NMR analysis of the crude reaction mixture with CH_2Br_2 as the internal standard, unless otherwise specified. [e] Not detected.

3. Experiment Procedure and Characterization Data

3.1 Preparation of dihydro-β-carboline

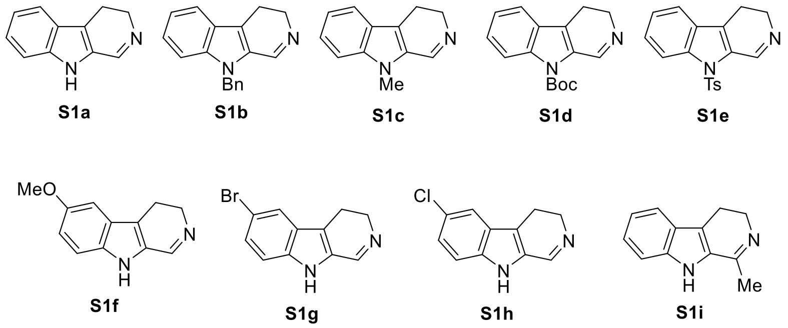

Substrates **S1a** is commercially available. **S1b**[1], **S1c**[2], **S1e**[3], **S1d**[4] **S1f**[1], **S1g**[5], **S1h**[5] and **S1i**[6] are known compounds and reported in related literatures.

3.2 Preparation of *N*-alkyl dihydro-β-carbolinium (DHβC) Ions[7]

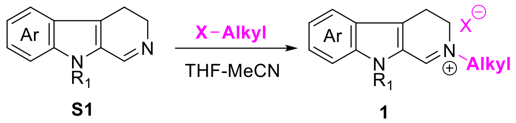

N-Alkyl DHβC ions **1** were prepared following the published procedure[7]: To a solution of dihydro-β-carboline **S1** (1.0 mmol) in 1.25mL anhydrous THF/MeCN (4/1) was added alkyl bromide (1.1 mmol) under a nitrogen atmosphere at room temperature. And then the reaction mixture was placed in a pre-heated oil bath at 50 °C for 16 – 24h, the precipitate **1** was collected and used for further Petasis-type coupling without further purification.

3.3 Copper-catalyzed Petasis-type reaction of N-alkyl DHβC ions

3.3.1 Petasis-type coupling of N-alkyl DHβC ions with arylboronic acids

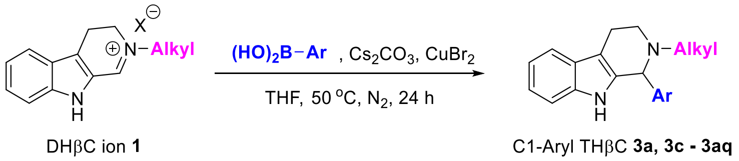

General Procedure A: To a flame-dried Schlenk tube were sequentially added *N*-alkyl DHβC ion **1** (0.2 mmol), $CuBr_2$ (9.0 mg, 20% mmol), Cs_2CO_3 (130 mg, 0.4 mmol), arylboronic acid (0.4 mmol), 5 Å molecular sieves (100 mg) and THF (4.0 mL, 0.05 M) under a nitrogen atmosphere. The reaction mixture in Schlenk tube was placed in a pre-heated oil bath at 50 °C for 24 h. After cooling to room temperature, the crude reaction was filtrated through a short pad of silica gel washed with EtOAc (25 mL). After solvent evaporation, the residue was purified by chromatography on silica gel to afford C1-aryl tetrahydro-β-carboline (THβC) **3a, 3c – 3aq**.

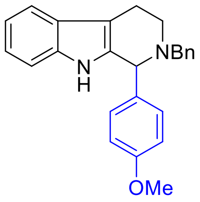

3a (**97 %**)

3a (71.5 mg, 97%) was obtained by using **General Procedure A** and purified by flash chromatography (silica gel, 10:1 hexane : EtOAc) in 97 % yield as white solid. ^{1}H NMR (600 MHz, $CDCl_3$) δ 7.55 – 7.50 (m, 1H), 7.38 – 7.30 (m, 6H), 7.27 (s, 2H), 7.21 – 7.18 (m, 1H), 7.11 (dtdt, J = 9.1, 7.1, 3.5, 1.7 Hz, 2H), 6.93 – 6.86 (m, 2H), 4.61 (s, 1H), 3.91 (d, J = 13.6 Hz, 1H), 3.82 (s, 3H), 3.35 (d, J = 13.3 Hz, 1H), 3.28 – 3.20 (m, 1H), 2.95 – 2.87 (m, 1H), 2.79 (dd, J = 15.1, 4.0 Hz, 1H), 2.67 (d, J = 11.9 Hz, 1H). ^{13}C NMR (151 MHz, $CDCl_3$) δ 159.6, 139.9, 136.4, 135.3, 133.5, 130.3, 128.8, 128.4, 127.4, 127.0, 121.6, 119.5, 118.4, 114.2, 110.9, 109.0, 64.1, 58.3, 55.4, 48.6, 21.3. HRMS (LC/Q-TOF) (m/z) *calcd.* for $C_{25}H_{24}N_2O$ $[M+H]^+$ 369.1961; found 369.1959.

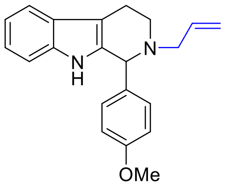

3c (**99 %**)

3c (63.1 mg, 99%) was obtained by using **General Procedure A** and purified by flash chromatography (silica gel, 10:1 hexane : EtOAc) in 99 % yield as white solid. ^{1}H NMR (600 MHz, $CDCl_3$) δ 7.53 (dd, J = 6.6, 2.2 Hz, 1H), 7.28 (d, J = 2.7 Hz, 2H), 7.26 (s, 1H), 7.18 (dd, J = 6.4, 2.0 Hz, 1H), 7.13 – 7.08 (m, 2H), 6.88 (d, J = 8.7 Hz, 2H), 5.92 (dddd, J = 17.4, 10.1, 7.6, 5.2 Hz, 1H), 5.23 – 5.15 (m, 2H), 4.61 (s, 1H), 3.81 (s, 3H), 3.37 – 3.29 (m, 2H), 2.98 (dt, J = 13.9, 7.2 Hz, 2H), 2.85 (dt, J = 15.2, 3.3 Hz, 1H), 2.73 (ddd, J = 13.2, 9.7, 4.2 Hz, 1H). ^{13}C NMR (151 MHz, $CDCl_3$) δ 159.6, 136.4, 135.7, 135.1, 132.9, 130.4, 127.3, 121.6, 119.4, 118.4, 117.9, 114.1, 110.9, 109.1, 63.4, 57.1, 55.4, 48.4, 21.2. HRMS (LC/Q-TOF) (m/z) *calcd.* for $C_{21}H_{22}N_2O$ $[M+H]^+$ 319.1805; found 319.1808.

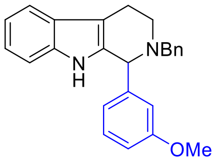

3d (**99 %**)

3d (73.0 mg, 99%) was obtained by using **General Procedure A** and purified by flash chromatography (silica gel, 10:1 hexane : EtOAc) in 99 % yield as white solid. ^{1}H NMR (600 MHz, $CDCl_3$) δ 7.54 (d, J = 7.0 Hz, 1H), 7.40 (d, J = 7.4 Hz, 2H), 7.37 – 7.27 (m, 5H), 7.20 (dd, J = 6.9, 1.4 Hz, 1H), 7.13 (pd, J = 7.0, 1.3 Hz, 2H), 7.08 (d, J = 7.1 Hz, 2H), 6.92 – 6.85 (m, 1H), 4.64 (s, 1H), 3.95 (d, J = 13.6 Hz, 1H), 3.81 (s, 3H), 3.39 (d, J = 13.1 Hz, 1H), 3.33 – 3.23 (m, 1H), 3.00 – 2.88 (m, 1H), 2.81 (d, J = 15.2 Hz, 1H), 2.68 (t, J = 8.4 Hz, 1H). ^{13}C NMR (151 MHz, $CDCl_3$) δ 160.2, 143.3, 139.7, 136.4, 134.9, 129.9, 128.8, 128.4, 127.3, 127.0, 121.6, 121.4, 119.5, 118.4, 114.2, 113.8, 110.9, 108.9, 64.9, 58.6, 55.4, 48.7, 21.4. HRMS (LC/Q-TOF) (m/z) *calcd.* for $C_{25}H_{24}N_2O$ $[M+H]^+$ 369.1961; found 369.1966.

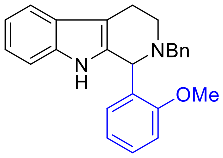

3e (**96 %**)

3e (70.8 mg, 96%) was obtained by using **General Procedure A** and purified by flash chromatography (silica gel, 10:1 hexane : EtOAc) in 96 % yield as white solid. ^{1}H NMR (600 MHz, $CDCl_3$) δ 7.54 (d, J = 7.1 Hz, 1H), 7.40 (d, J = 7.4 Hz, 2H), 7.32 (ddd, J = 28.5, 13.8, 7.3 Hz, 5H), 7.20 (d, J = 7.1 Hz, 1H), 7.16 – 7.05 (m, 4H), 6.91 – 6.86 (m, 1H), 4.64 (s, 1H), 3.95 (d, J = 13.6 Hz, 1H), 3.80 (s, 3H), 3.39 (d, J = 13.6 Hz, 1H), 3.27 (dt, J = 11.6, 4.3 Hz, 1H), 2.94 (ddd, J = 13.7, 7.1, 3.9 Hz, 1H), 2.81 (d, J = 15.3 Hz, 1H), 2.67 (td, J = 11.6, 4.0 Hz,

1H). ^{13}C NMR (151 MHz, $CDCl_3$) δ 160.2, 143.3, 139.7, 136.4, 134.9, 129.8, 128.8, 128.4, 127.3, 127.0, 121.6, 121.4, 119.4, 118.4, 114.2, 113.8, 110.9, 108.9, 64.9 58.6, 55.4, 48.7, 21.4. HRMS (LC/Q-TOF) (m/z) *calcd.* for $C_{25}H_{24}N_2O$ $[M+H]^+$ 369.1961; found 369.1966.

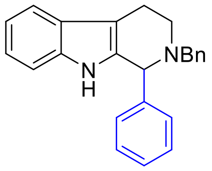

3f (**80 %**)

3f (54.2 mg, 80%) was obtained by using **General Procedure A** and purified by flash chromatography (silica gel, 10:1 hexane : EtOAc) in 80 % yield as white solid. ^{1}H NMR (600 MHz, $CDCl_3$) δ 7.54 (dd, J = 6.8, 1.9 Hz, 1H), 7.49 – 7.45 (m, 2H), 7.42 – 7.36 (m, 4H), 7.34 (t, J = 7.5 Hz, 3H), 7.30 – 7.26 (m, 2H), 7.21 – 7.17 (m, 1H), 7.12 (dtd, J = 12.6, 7.1, 1.5 Hz, 2H), 4.67 (s, 1H), 3.92 (d, J = 13.6 Hz, 1H), 3.40 (d, J = 13.5 Hz, 1H), 3.26 (dt, J = 11.9, 4.7 Hz, 1H), 2.94 (dt, J = 17.4, 6.3 Hz, 1H), 2.86 – 2.78 (m, 1H), 2.73 – 2.64 (m, 1H). ^{13}C NMR (151 MHz, $CDCl_3$) δ 141.6, 139.7, 136.4, 135.0, 129.2, 128.89, 128.86, 128.4, 128.2, 127.3, 127.1, 121.6, 119.5, 118.4, 110.9, 109.1, 64.7, 58.4, 48.5, 21.3. HRMS (LC/Q-TOF) (m/z) *calcd.* for $C_{24}H_{22}N_2$ $[M+H]^+$ 339.1856; found 339.1858.

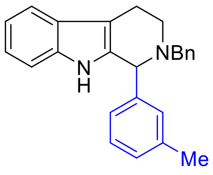

3g (**98 %**)

3g (69.1 mg, 98%) was obtained by using **General Procedure A** and purified by flash chromatography (silica gel, 10:1 hexane : EtOAc) in 98 % yield as white solid. ^{1}H NMR (600 MHz, $CDCl_3$) δ 7.55 (d, J = 6.9 Hz, 1H), 7.38 (d, J = 7.3 Hz, 2H), 7.34 (t, J = 7.5 Hz, 2H), 7.28 (q, J = 6.4, 4.9 Hz, 5H), 7.20 (dd, J = 6.7, 1.6 Hz, 1H), 7.17 – 7.14 (m, 1H), 7.14 – 7.07 (m, 2H), 4.63 (s, 1H), 3.94 (d, J = 13.5 Hz, 1H), 3.38 (d, J = 13.3 Hz, 1H), 3.31 – 3.23 (m, 1H), 3.00 – 2.90 (m, 1H), 2.82 (d, J = 15.2 Hz, 1H), 2.67 (t, J = 9.0 Hz, 1H), 2.38 (s, 3H). ^{13}C NMR (151 MHz, $CDCl_3$) δ 141.4, 139.7, 138.6, 136.4, 135.2, 129.7, 129.0, 128.9, 128.7, 128.3, 127.3, 127.0, 126.3, 121.6, 119.5, 118.4, 110.9, 108.9, 64.9, 58.5, 48.6, 21.6, 21.4. HRMS (LC/Q-TOF) (m/z) *calcd.* for $C_{25}H_{24}N_2$ $[M+H]^+$ 353.2012; found 353.2016.

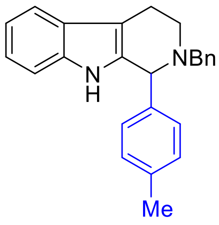

3h (**88 %**)

3h (62.0 mg, 88%) was obtained by using **General Procedure A** and purified by flash chromatography (silica gel, 10:1 hexane : EtOAc) in 88 % yield as white solid. ^{1}H NMR (500 MHz, $CDCl_3$) δ 7.55 – 7.52 (m, 1H), 7.39 – 7.32 (m, 6H), 7.32 – 7.27 (m, 2H), 7.21 – 7.17 (m, 3H), 7.14 – 7.08 (m, 2H), 4.63 (d, J = 1.9 Hz, 1H), 3.92 (d, J = 13.5 Hz, 1H), 3.37 (d, J = 13.6 Hz, 1H), 3.25 (ddd, J = 11.8, 5.3, 3.5 Hz, 1H), 2.93 (dddd, J = 15.1, 9.6, 5.3, 2.1 Hz, 1H), 2.81 (dtd, J = 15.3, 4.0, 1.6 Hz, 1H), 2.67 (ddd, J = 11.8, 9.6, 4.3 Hz, 1H), 2.37 (s, 3H). ^{13}C NMR (126 MHz, $CDCl_3$) δ 139.7, 138.4, 137.9, 136.4, 135.2, 129.6, 129.1, 128.9, 128.3, 127.4, 127.0, 121.6, 119.4, 118.4, 110.9, 109.0, 64.5, 58.4, 48.5, 21.34, 21.30. HRMS (LC/Q-TOF) (m/z) *calcd.* for $C_{25}H_{24}N_2$ $[M+H]^+$ 353.2012; found 353.2016.

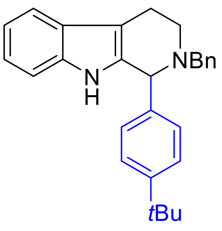

3i (85%)

3i (67.1 mg, 85%) was obtained by using **General Procedure A** and purified by flash chromatography (silica gel, 10:1 hexane : EtOAc) in 85 % yield as white solid. ^{1}H NMR (500 MHz, $CDCl_3$) δ 7.56 – 7.52 (m, 1H), 7.41 – 7.32 (m, 8H), 7.33 – 7.27 (m, 2H), 7.20 – 7.17 (m, 1H), 7.15 – 7.09 (m, 2H), 4.66 (s, 1H), 3.94 (d, J = 13.5 Hz, 1H), 3.40 (d, J = 13.5 Hz, 1H), 3.26 (dt, J = 12.0, 4.5 Hz, 1H), 2.99 – 2.87 (m, 1H), 2.86 – 2.79 (m, 1H), 2.70 (d, J = 11.9 Hz, 1H), 1.35 (s, 9H). ^{13}C NMR (126 MHz, $CDCl_3$) δ 151.1, 139.8, 138.2, 136.4, 135.1, 128.9, 128.8, 128.4, 127.4, 127.0, 125.8, 121.5, 119.4, 118.4, 110.9, 108.9, 64.2, 58.4, 48.4, 34.7, 31.5, 21.2. HRMS (LC/Q-TOF) (m/z) *calcd.* for $C_{28}H_{30}N_2$ $[M+H]^+$ 395.2482; found 395.2485.

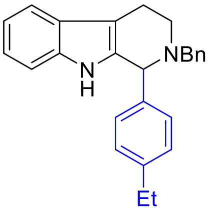

3j (89 %)

3j (65.2 mg, 89%) was obtained by using **General Procedure A** and purified by flash chromatography (silica gel, 10:1 hexane : EtOAc) in 89 % yield as white solid. ^{1}H NMR (600 MHz, $CDCl_3$) δ 7.53 (dd, J = 7.5, 1.5 Hz, 1H), 7.39 – 7.31 (m, 6H), 7.30 – 7.26 (m, 2H), 7.23 – 7.18 (m, 3H), 7.11 (dtd, J = 12.3, 7.0, 1.5 Hz, 2H), 4.64 (s, 1H), 3.93 (d, J = 13.6 Hz, 1H), 3.38 (d, J = 13.6 Hz, 1H), 3.25 (dt, J = 12.0, 4.4 Hz, 1H), 2.98 – 2.89 (m, 1H), 2.85 – 2.78 (m, 1H), 2.67 (q, J = 7.6 Hz, 3H), 1.26 (t, J = 7.6 Hz, 3H). ^{13}C NMR (151 MHz, $CDCl_3$) δ 144.1, 139.7, 138.5, 136.3, 135.1, 129.0, 128.8, 128.3, 128.2 , 127.3, 126.9, 121.5, 119.3, 118.3, 110.8, 108.8, 64.4, 58.2, 48.4, 28.6, 21.2, 15.5. HRMS (LC/Q-TOF) (m/z) *calcd.* for $C_{26}H_{26}N_2$ $[M+H]^+$ 367.2169; found 367.2174.

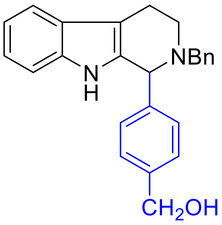

3k (63%)

3k (46.4 mg, 63%) was obtained by using **General Procedure A** and purified by flash chromatography (silica gel, 3:1 hexane : EtOAc) in 63 % yield as white solid. ^{1}H NMR (500 MHz, DMSO-d6) δ 10.29 (s, 1H), 7.41 (d, J = 7.6 Hz, 1H), 7.33 (q, J = 3.9, 3.5 Hz, 8H), 7.27 – 7.20 (m, 2H), 7.00 (ddd, J = 8.1, 7.0, 1.3 Hz, 1H), 6.95 (td, J = 7.5, 1.1 Hz, 1H), 5.16 (t, J = 5.7 Hz, 1H), 4.72 (s, 1H), 4.49 (d, J = 5.6 Hz, 2H), 3.72 (d, J = 13.7 Hz, 1H), 3.47 (d, J = 13.7 Hz, 1H), 3.02 (dt, J = 11.8, 5.1 Hz, 1H), 2.80 – 2.68 (m, 2H), 2.61 (ddd, J = 12.0, 7.1, 5.1 Hz, 1H). ^{13}C NMR (126 MHz, DMSO-d6) δ 141.8, 140.1, 139.4, 136.4, 134.9, 128.6, 128.4, 128.2, 126.9, 126.5, 126.4, 120.6, 118.3, 117.6, 111.1, 107.0, 62.7, 62.6, 57.2, 46.6, 20.4. HRMS (LC/Q-TOF) (m/z) *calcd.* for $C_{25}H_{24}N_2O$ $[M+H]^+$ 369.1961; found 369.1966.

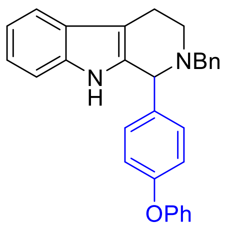

3l (88%)

3l (75.8 mg, 88%) was obtained by using **General Procedure A** and purified by flash chromatography (silica gel, 10:1 hexane : EtOAc) in 88 % yield as white solid. ^{1}H NMR (600 MHz, $CDCl_3$) δ 7.55 (d, J = 7.0 Hz, 1H), 7.45 – 7.25 (m, 10H), 7.25 – 7.21 (m, 1H), 7.17 – 7.10 (m, 3H), 7.09 – 7.04 (m, 2H), 7.01 (d, J = 8.6 Hz, 2H), 4.67 (s, 1H), 3.95 (d, J = 13.5 Hz, 1H), 3.42 (d, J = 13.6 Hz, 1H), 3.26 (dt, J = 12.0, 4.6 Hz, 1H), 2.93 (dt, J = 15.1, 7.7 Hz, 1H), 2.87 – 2.78 (m, 1H), 2.72 (d, J = 12.2 Hz, 1H). ^{13}C NMR (151 MHz, $CDCl_3$) δ 157.4, 156.9, 139.7, 136.4, 136.1, 134.8, 130.4, 129.9, 128.9, 128.4, 127.3, 127.1, 123.7, 121.7, 119.5, 119.4, 118.8, 118.5, 110.9, 109.1, 63.9, 58.3, 48.4, 21.2. HRMS (LC/Q-TOF) (m/z) *calcd.* for $C_{30}H_{26}N_2O$ $[M+H]^+$ 431.2118; found 431.2123.

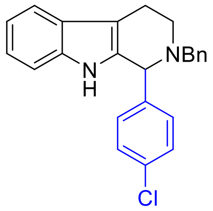

3m (82 %)

3m (61.2 mg, 82%) was obtained by using **General Procedure A** and purified by flash chromatography (silica gel, 10:1 hexane : EtOAc) in 82 % yield as white solid. ^{1}H NMR (600 MHz, $CDCl_3$) δ 7.57 – 7.52 (m, 1H), 7.41 – 7.32 (m, 8H), 7.28 (ddd, J = 9.9, 6.4, 3.1 Hz, 2H), 7.23 – 7.19 (m, 1H), 7.13 (dtd, J = 17.1, 7.1, 1.3 Hz, 2H), 4.66 (s, 1H), 3.88 (d, J = 13.5 Hz, 1H), 3.41 (d, J = 13.5 Hz, 1H), 3.23 (dt, J = 12.1, 4.5 Hz, 1H), 2.92 (dt, J = 15.3, 7.0 Hz, 1H), 2.87 – 2.79 (m, 1H), 2.74 – 2.65 (m, 1H). ^{13}C NMR (151 MHz, $CDCl_3$) δ 140.2, 139.3, 136.5, 134.2, 133.9, 130.5, 129.1, 128.8, 128.4, 127.2, 126.1, 121.9, 119.6, 118.5, 111.0, 109.4, 63.8, 58.3, 48.2, 21.1. HRMS (LC/Q-TOF) (m/z) *calcd.* for $C_{24}H_{21}ClN_2$ $[M+H]^+$ 373.1466; found 373.1481.

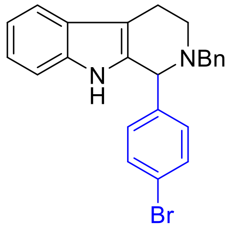

3n (70 %)

3n (58.4 mg, 70%) was obtained by using **General Procedure A** and purified by flash chromatography (silica gel, 10:1 hexane : EtOAc) in 70 % yield as white solid. ^{1}H NMR (600 MHz, $CDCl_3$) δ 7.54 (dd, J = 7.4, 1.5 Hz, 1H), 7.52 – 7.48 (m, 2H), 7.36 – 7.30 (m, 6H), 7.28 (dq, J = 8.0, 2.8 Hz, 1H), 7.25 (s, 1H), 7.23 – 7.20 (m, 1H), 7.13 (dtd, J = 17.2, 7.1, 1.3 Hz, 2H), 4.63 (s, 1H), 3.87 (d, J = 13.5 Hz, 1H), 3.40 (d, J = 13.5 Hz, 1H), 3.22 (ddd, J = 11.9, 5.2, 3.9 Hz, 1H), 2.91 (dddd, J = 14.8, 9.3, 5.2, 2.0 Hz, 1H), 2.82 (dtd, J = 15.3, 4.2, 1.5 Hz, 1H), 2.69 (ddd, J = 13.1, 9.3, 4.3 Hz, 1H). ^{13}C NMR (151 MHz, $CDCl_3$) δ 140.8, 139.4, 136.5, 134.2, 132.0, 130.8, 128.8, 128.4, 127.22, 127.19, 122.1, 121.9, 119.6, 118.5, 111.0, 109.4, 63.9, 58.4, 48.3, 21.2. HRMS (LC/Q-TOF) (m/z) *calcd.* for $C_{24}H_{21}BrN_2$ $[M+H]^+$ 417.0961; found 417.0956.

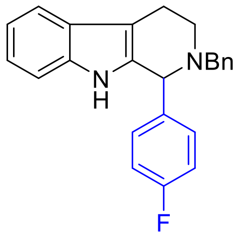

3o (**77 %**)

3o (54.9 mg, 77%) was obtained by using **General Procedure A** and purified by flash chromatography (silica gel, 10:1 hexane : EtOAc) in 77 % yield as white solid. ^{1}H NMR (600 MHz, $CDCl_3$) δ 7.54 (dd, J = 7.3, 1.5 Hz, 1H), 7.43 – 7.40 (m, 2H), 7.37 – 7.33 (m, 4H), 7.30 – 7.25 (m, 2H), 7.23 – 7.20 (m, 1H), 7.13 (dtd, J = 16.0, 7.1, 1.3 Hz, 2H), 7.10 – 7.04 (m, 2H), 4.66 (s, 1H), 3.89 (d, J = 13.5 Hz, 1H), 3.40 (d, J = 13.5 Hz, 1H), 3.26 – 3.21 (m, 1H), 2.95 – 2.88 (m, 1H), 2.82 (dtd, J = 15.3, 4.2, 1.6 Hz, 1H), 2.69 (ddd, J = 13.5, 9.4, 4.3 Hz, 1H). ^{13}C NMR (151 MHz, $CDCl_3$) δ 162.6 (d, J = 246.4 Hz)., 139.5, 136.4, 134.6, 130.7 (d, J = 8.1 Hz), 128.8, 128.4, 127.3, 127.2, 121.8, 119.6, 118.5, 115.8, 115.7, 110.9, 109.3, 63.7, 58.3, 48.3, 21.2. ^{19}F NMR (471 MHz, $CDCl_3$) δ -114.19. HRMS (LC/Q-TOF) (m/z) *calcd.* for $C_{24}H_{21}FN_2$ $[M+H]^+$ 357.1762; found 357.1765.

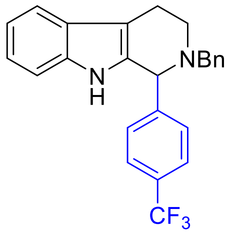

3p (**68 %**)

3p (55.3 mg, 68%) was obtained by using **General Procedure A** and purified by flash chromatography (silica gel, 10:1 hexane : EtOAc) in 68 % yield as white solid. ^{1}H NMR (600 MHz, $CDCl_3$) δ 7.66 (d, J = 8.1 Hz, 2H), 7.61 – 7.56 (m, 3H), 7.40 – 7.35 (m, 4H), 7.31 (dq, J = 7.9, 2.7 Hz, 1H), 7.27 (d, J = 5.7 Hz, 1H), 7.25 – 7.22 (m, 1H), 7.16 (pd, J = 7.0, 1.5 Hz, 2H), 4.76 (s, 1H), 3.88 (d, J = 13.5 Hz, 1H), 3.48 (d, J = 13.5 Hz, 1H), 3.26 (dt, J = 12.0, 4.8 Hz, 1H), 2.95 (dddd, J = 14.0, 8.9, 5.2, 1.9 Hz, 1H), 2.87 (dtd, J = 15.4, 4.3, 1.6 Hz, 1H), 2.74 (ddd, J = 12.7, 8.9, 4.4 Hz, 1H). ^{13}C NMR (151 MHz, $CDCl_3$) δ 145.9, 139.1, 136.5, 133.6, 130.4 (q, J = 32.4 Hz), 129.4, 128.8, 128.5, 127.3, 127.2, 125.8 (q, J = 3.7 Hz), 124.2 (d, J = 272.1 Hz), 122.0, 119.7, 118.6, 111.0, 109.6, 63.8, 58.5, 48.1, 21.0. ^{19}F NMR (471 MHz, $CDCl_3$) δ -62.39. HRMS (LC/Q-TOF) (m/z) *calcd.* for $C_{25}H_{21}F_3N_2$ $[M+H]^+$ 407.1730; found 407.1734.

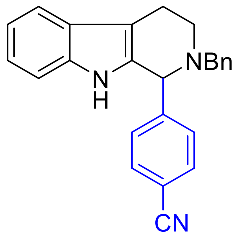

3q (**68 %**)

3q (49.4 mg, 68%) was obtained by using **General Procedure A** and purified by flash chromatography (silica gel, 10:1 hexane : EtOAc) in 68 % yield as white solid. ^{1}H NMR (600 MHz, $CDCl_3$) δ 7.65 – 7.62 (m, 2H), 7.56 – 7.53 (m, 3H), 7.35 (d, J = 5.6 Hz, 5H), 7.31 – 7.27 (m, 1H), 7.25 – 7.22 (m, 1H), 7.15 (dtd, J = 20.5, 7.1, 1.2 Hz, 2H), 4.75 (s, 1H), 3.83 (d, J = 13.5 Hz, 1H), 3.50 (d, J = 13.5 Hz, 1H), 3.20 (dt, J = 9.6, 4.8 Hz, 1H), 2.88 (td, J = 15.4, 13.9, 8.7 Hz, 2H), 2.75 (s, 1H). ^{13}C NMR (151 MHz, $CDCl_3$) δ 147.5, 139.0, 136.5, 132.9, 132.6, 129.8, 128.8, 128.5, 127.4, 127.0, 122.2, 119.8, 118.8, 118.6, 111.9, 111.0, 109.8, 63.5, 58.4, 47.7, 20.7. HRMS (LC/Q-TOF) (m/z) *calcd.* for $C_{25}H_{21}N_3$ $[M+H]^+$ 364.1808; found 364.1813.

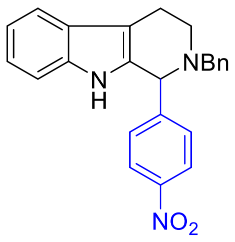

3r (57 %)

3r (43.7 mg, 57%) was obtained by using **General Procedure A** and purified by flash chromatography (silica gel, 10:1 hexane : EtOAc) in 57 % yield as yellow solid. ^{1}H NMR (600 MHz, $CDCl_3$) δ 8.20 (d, J = 8.7 Hz, 2H), 7.60 (d, J = 8.6 Hz, 2H), 7.55 (d, J = 7.7 Hz, 1H), 7.34 (d, J = 5.0 Hz, 5H), 7.29 (q, J = 3.5 Hz, 1H), 7.23 (d, J = 7.9 Hz, 1H), 7.18 – 7.11 (m, 2H), 4.80 (s, 1H), 3.83 (d, J = 13.5 Hz, 1H), 3.51 (d, J = 13.5 Hz, 1H), 3.21 (dt, J = 11.4, 4.8 Hz, 1H), 2.89 (d, J = 14.8 Hz, 2H), 2.76 (s, 1H). ^{13}C NMR (151 MHz, $CDCl_3$) δ 149.6, 147.9, 138.9, 136.6, 132.8, 129.9, 128.8, 128.6, 127.5, 127.1, 124.1, 122.3, 119.9, 118.7, 111.1, 109.9, 63.2, 58.5, 47.8, 20.7. HRMS (LC/Q-TOF) (m/z) *calcd.* for $C_{24}H_{21}N_3O_2$ $[M+H]^+$ 384.1707; found 384.1709.

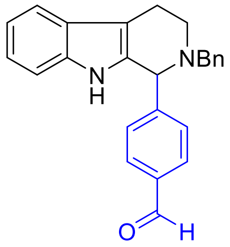

3s (78%)

3s (57.2 mg, 78%) was obtained by using **General Procedure A** and purified by flash chromatography (silica gel, 10:1 hexane : EtOAc) in 78 % yield as white solid. ^{1}H NMR (600 MHz, $CDCl_3$) δ 9.95 (s, 1H), 7.88 – 7.83 (m, 2H), 7.65 – 7.60 (m, 2H), 7.58 – 7.54 (m, 1H), 7.47 (s, 1H), 7.38 – 7.32 (m, 4H), 7.28 (ddt, J = 8.6, 5.3, 2.4 Hz, 1H), 7.24 – 7.20 (m, 1H), 7.18 – 7.09 (m, 2H), 4.77 (s, 1H), 3.86 (d, J = 13.5 Hz, 1H), 3.47 (d, J = 13.5 Hz, 1H), 3.24 (dt, J = 11.9, 4.7 Hz, 1H), 2.98 – 2.90 (m, 1H), 2.86 (dtd, J = 15.4, 4.3, 1.5 Hz, 1H), 2.73 (ddd, J = 12.1, 8.9, 4.2 Hz, 1H). ^{13}C NMR (151 MHz, $CDCl_3$) δ 191.9, 149.0, 139.1, 136.6, 136.3, 133.5, 130.3, 129.8, 128.8, 128.5, 127.3, 127.1, 122.0, 119.7, 118.6, 111.0, 109.5, 64.0, 58.6, 48.1, 21.0. HRMS (LC/Q-TOF) (m/z) *calcd.* for $C_{25}H_{22}N_2O$ $[M+H]^+$ 367.1805; found 367.1809.

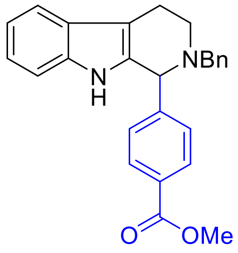

3t (75%)

3t (59.5 mg, 75%) was obtained by using **General Procedure A** and purified by flash chromatography (silica gel, 10:1 hexane : EtOAc) in 75 % yield as white solid. ^{1}H NMR (500 MHz, $CDCl_3$) δ 8.03 (d, J = 8.3 Hz, 2H), 7.55 – 7.50 (m, 3H), 7.40 – 7.32 (m, 5H), 7.28 (dd, J = 6.0, 2.7 Hz, 1H), 7.22 – 7.19 (m, 1H), 7.16 – 7.09 (m, 2H), 4.74 (s, 1H), 3.91 (s, 3H), 3.86 (d, J = 13.5 Hz, 1H), 3.43 (d, J = 13.5 Hz, 1H), 3.23 (dt, J = 11.9, 4.5 Hz, 1H), 2.96 – 2.88 (m, 1H), 2.87 – 2.79 (m, 1H), 2.72 (d, J = 12.3 Hz, 1H). ^{13}C NMR (126 MHz, $CDCl_3$) δ 167.0, 147.0, 139.2, 136.5, 133.9, 130.2, 130.1, 129.1, 128.9, 128.4, 127.24, 127.17, 121.9, 119.6, 118.5, 111.0, 109.4, 64.1, 58.5, 52.3, 48.2, 21.1. HRMS (LC/Q-TOF) (m/z) *calcd.* for $C_{26}H_{24}N_2O_2$ $[M+H]^+$ 397.1911; found 397.1914.

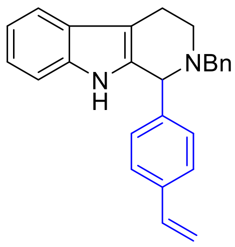

3u (72 %)

3u (52.5 mg, 72%) was obtained by using **General Procedure A** and purified by flash chromatography (silica gel, 10:1 hexane : EtOAc) in 72 % yield as white solid. ^{1}H NMR (600 MHz, $CDCl_3$) δ 7.58 – 7.54 (m, 1H), 7.44 (s, 4H), 7.39 (d, J = 7.1 Hz, 2H), 7.35 (t, J = 7.6 Hz, 2H), 7.32 – 7.26 (m, 2H), 7.22 – 7.17 (m, 1H), 7.14 (tt, J = 7.1, 5.5 Hz, 2H), 6.76 (dd, J = 17.6, 10.9 Hz, 1H), 5.79 (dd, J = 17.6, 0.9 Hz, 1H), 5.29 (dd, J = 10.9, 0.9 Hz, 1H), 4.67 (s, 1H), 3.94 (d, J = 13.6 Hz, 1H), 3.41 (d, J = 13.6 Hz, 1H), 3.30 – 3.22 (m, 1H), 3.00 – 2.89 (m, 1H), 2.83 (dtd, J = 15.2, 4.0, 1.5 Hz, 1H), 2.75 – 2.65 (m, 1H). ^{13}C NMR (151 MHz, $CDCl_3$) δ 141.1, 139.6, 137.6, 136.5, 136.4, 134.8, 129.4, 128.8, 128.4, 127.3, 127.1, 126.7, 121.7, 119.5, 118.4, 114.3, 110.9, 109.1, 64.4, 58.4, 48.5, 21.3. HRMS (LC/Q-TOF) (m/z) *calcd.* for $C_{26}H_{24}N_2$ $[M+H]^+$ 365.2012; found 365.2017.

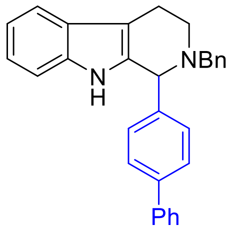

3v (74 %)

3v (61.4 mg, 74%) was obtained by using **General Procedure A** and purified by flash chromatography (silica gel, 10:1 hexane : EtOAc) in 74 % yield as white solid. ^{1}H NMR (600 MHz, $CDCl_3$) δ 7.61 – 7.58 (m, 4H), 7.53 (dd, J = 15.8, 7.5 Hz, 3H), 7.45 (t, J = 7.7 Hz, 2H), 7.41 – 7.32 (m, 6H), 7.27 (d, J = 7.2 Hz, 1H), 7.23 – 7.20 (m, 1H), 7.12 (pd, J = 7.1, 1.4 Hz, 2H), 4.71 (s, 1H), 3.96 (d, J = 13.6 Hz, 1H), 3.42 (d, J = 13.6 Hz, 1H), 3.30 – 3.24 (m, 1H), 2.94 (d, J = 9.6 Hz, 1H), 2.83 (d, J = 15.9 Hz, 1H), 2.70 (s, 1H). ^{13}C NMR (151 MHz, $CDCl_3$) δ 141.2, 140.8, 140.6, 139.7, 136.5, 134.9, 129.6, 128.94, 128.86, 128.4, 127.6, 127.5, 127.3, 127.2, 127.1, 121.7, 119.5, 118.5, 111.0, 109.2, 64.4, 58.5, 48.5, 21.3. HRMS (LC/Q-TOF) (m/z) *calcd.* for $C_{30}H_{26}N_2$ $[M+H]^+$ 415.2169; found 415.2173.

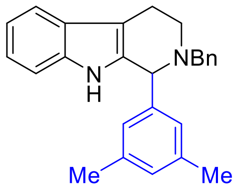

3w (84 %)

3w (61.6 mg, 84%) was obtained by using **General Procedure A** and purified by flash chromatography (silica gel, 10:1 hexane : EtOAc) in 84 % yield as white solid. ^{1}H NMR (600 MHz, $CDCl_3$) δ 7.55 (dd, J = 6.8, 2.0 Hz, 1H), 7.40 – 7.33 (m, 4H), 7.32 – 7.26 (m, 2H), 7.22 – 7.19 (m, 1H), 7.15 – 7.08 (m, 4H), 6.99 (s, 1H), 4.59 (s, 1H), 3.95 (d, J = 13.5 Hz, 1H), 3.36 (d, J = 13.6 Hz, 1H), 3.27 (dt, J = 11.9, 3.8 Hz, 1H), 2.96 (dq, J = 15.7, 5.9 Hz, 1H), 2.85 – 2.79 (m, 1H), 2.66 (t, J = 11.6 Hz, 1H), 2.35 (s, 6H). ^{13}C NMR (151 MHz, $CDCl_3$) δ 141.4, 139.8, 138.4, 136.4, 135.4, 129.9, 128.9, 128.3, 127.4, 127.0, 126.9, 121.5, 119.4, 118.4, 110.9, 108.8, 65.0, 58.6, 48.7, 21.5, 21.4. HRMS (LC/Q-TOF) (m/z) *calcd.* for $C_{26}H_{26}N_2$ $[M+H]^+$ 367.2169; found 367.2174.

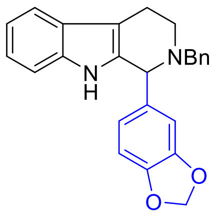

3x (65 %)

3x (49.7 mg, 65%) was obtained by using **General Procedure A** and purified by flash chromatography (silica gel, 10:1 hexane : EtOAc) in 65 % yield as white solid. ^{1}H NMR (500 MHz, $CDCl_3$) δ 7.52 (dd, J = 7.2, 1.6 Hz, 1H), 7.35 (dt, J = 15.0, 7.2 Hz, 5H), 7.29 – 7.26 (m, 1H), 7.21 (dt, J = 8.0, 0.9 Hz, 1H), 7.15 – 7.08 (m, 2H), 6.96 (d, J = 1.7 Hz, 1H), 6.92 (dd, J = 7.8, 1.7 Hz, 1H), 6.81 (d, J = 7.9 Hz, 1H), 5.96 – 5.94 (m, 2H), 4.59 (s, 1H), 3.95 (d, J = 13.5 Hz, 1H), 3.37 (d, J = 13.6 Hz, 1H), 3.24 (dt, J = 11.9, 4.2 Hz, 1H), 2.95 – 2.87 (m, 1H), 2.79 (dt, J = 15.7, 4.2 Hz, 1H), 2.65 (s, 1H). ^{13}C NMR (126 MHz, $CDCl_3$) δ 148.4, 147.7, 139.6, 136.4, 135.5, 135.1, 128.8, 128.4, 127.3, 127.1, 122.4, 121.7, 119.5, 118.5, 110.9, 109.0, 108.14, 108.07, 101.3, 64.5, 58.3, 48.5, 21.3. HRMS (LC/Q-TOF) (m/z) *calcd.* for $C_{25}H_{22}N_2O_2$ $[M+H]^+$ 383.1754; found 383.1758.

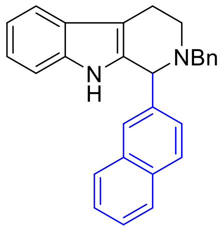

3y (74 %)

3y (57.5 mg, 74%) was obtained by using **General Procedure A** and purified by flash chromatography (silica gel, 10:1 hexane : EtOAc) in 74 % yield as white solid. ^{1}H NMR (500 MHz, DMSO-d6) δ 10.33 (s, 1H), 7.93 (d, J = 7.2 Hz, 2H), 7.90 – 7.85 (m, 2H), 7.56 (dd, J = 8.5, 1.7 Hz, 1H), 7.50 (tt, J = 7.0, 5.2 Hz, 2H), 7.45 (d, J = 7.7 Hz, 1H), 7.32 (d, J = 6.3 Hz, 4H), 7.22 (t, J = 8.3 Hz, 2H), 6.99 (dt, J = 21.2, 7.2 Hz, 2H), 4.89 (s, 1H), 3.79 (d, J = 13.7 Hz, 1H), 3.45 (d, J = 13.7 Hz, 1H), 3.10 (dt, J = 12.0, 4.6 Hz, 1H), 2.80 (q, J = 4.7 Hz, 2H), 2.64 (ddd, J = 12.5, 7.7, 5.0 Hz, 1H). ^{13}C NMR (126 MHz, DMSO-d6) δ 139.4, 136.6, 134.8, 132.8, 132.7, 128.4, 128.2, 128.1, 128.0, 127.8, 127.5, 126.9, 126.6, 126.5, 126.1, 125.9, 120.6, 118.3, 117.7, 111.2, 107.3, 63.5, 57.4, 47.1, 20.7. HRMS (LC/Q-TOF) (m/z) *calcd.* for $C_{28}H_{24}N_2$ $[M+H]^+$ 389.2012; found 389.2016.

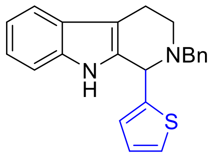

3z (36 %)

3z (24.8 mg, 36%) was obtained by using **General Procedure A** and purified by flash chromatography (silica gel, 10:1 hexane : EtOAc) in 36 % yield as yellow solid. ^{1}H NMR (500 MHz, Chloroform-d) δ 7.57 – 7.53 (m, 1H), 7.51 (s, 1H), 7.47 (d, J = 7.1 Hz, 2H), 7.37 (t, J = 7.5 Hz, 2H), 7.33 – 7.29 (m, 2H), 7.25 (d, J = 7.9 Hz, 1H), 7.19 – 7.11 (m, 3H), 7.02 (dd, J = 5.1, 3.4 Hz, 1H), 5.07 (s, 1H), 4.02 (d, J = 13.6 Hz, 1H), 3.53 (d, J = 13.6 Hz, 1H), 3.28 (dt, J = 12.1, 5.0 Hz, 1H), 2.92 – 2.81 (m, 2H), 2.76 (ddd, J = 12.5, 8.1, 4.6 Hz, 1H). ^{13}C NMR (126 MHz, Chloroform-d) δ 145.8, 139.1, 136.3, 133.9, 128.9, 128.4, 127.3, 127.2, 126.5, 126.4, 126.3, 121.9, 119.5, 118.6, 111.0, 108.6, 59.2, 58.2, 47.9, 20.8. HRMS (LC/Q-TOF) (m/z) *calcd.* for $C_{22}H_{20}N_2S$ $[M+H]^+$ 345.1420; found 345.1424.

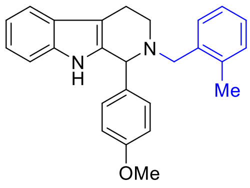

3aa (99 %)

3aa (75.7 mg, 99%) was obtained by using **General Procedure A** and purified by flash chromatography (silica gel, 10:1 hexane : EtOAc) in 99 % yield as white solid. ^{1}H NMR (400 MHz, $CDCl_3$) δ 7.62 – 7.56 (m, 1H), 7.50 – 7.44 (m, 1H), 7.39 (s, 1H), 7.37 – 7.31 (m, 2H), 7.25 – 7.13 (m, 6H), 6.96 – 6.88 (m, 2H), 4.62 (s, 1H), 3.88 (d, J = 13.5 Hz, 1H), 3.84 (s, 3H), 3.42 (d, J = 13.5 Hz, 1H), 3.24 (ddd, J = 11.7, 5.1, 3.9 Hz, 1H), 2.95 (dddd, J = 16.0, 9.1, 5.2, 2.0 Hz, 1H), 2.85 (dtd, J = 15.4, 4.2, 1.5 Hz, 1H), 2.66 (ddd, J = 11.7, 9.1, 4.4 Hz, 1H), 2.28 (s, 3H). ^{13}C NMR (101 MHz, $CDCl_3$) δ 159.4, 137.6, 137.3, 136.3, 135.3, 133.3, 130.4, 130.3, 129.6, 127.3, 126.9, 125.7, 121.5, 119.4, 118.3, 113.9, 110.9, 109.2, 64.6, 56.3, 55.4, 48.2, 21.1, 19.5. HRMS (LC/Q-TOF) (m/z) *calcd.* for $C_{26}H_{26}N_2O$ $[M+H]^+$ 383.2118; found 383.2120.

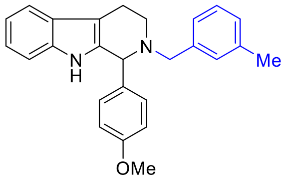

3ab (97 %)

3ab (66.6 mg, 97%) was obtained by using **General Procedure A** and purified by flash chromatography (silica gel, 10:1 hexane : EtOAc) in 87 % yield as white solid. ^{1}H NMR (400 MHz, $CDCl_3$) δ 7.57 (dt, J = 7.3, 2.0 Hz, 1H), 7.42 – 7.33 (m, 3H), 7.27 (td, J = 7.7, 2.0 Hz, 1H), 7.23 – 7.10 (m, 6H), 6.98 – 6.86 (m, 2H), 4.64 (s, 1H), 3.92 (d, J = 13.5 Hz, 1H), 3.84 (d, J = 1.0 Hz, 3H), 3.37 (d, J = 13.4 Hz, 1H), 3.32 – 3.20 (m, 1H), 3.03 – 2.91 (m, 1H), 2.84 (dddd, J = 15.4, 6.1, 4.0, 1.7 Hz, 1H), 2.70 (tdd, J = 9.6, 4.4, 2.2 Hz, 1H), 2.41 (d, J = 2.0 Hz, 3H). ^{13}C NMR (101 MHz, $CDCl_3$) δ 159.4, 139.6, 137.8, 136.3, 135.2, 133.3, 130.3, 129.6, 128.2, 127.7, 127.3, 125.9, 121.5, 119.4, 118.3, 114.1, 110.9, 108.9, 63.9, 58.2, 55.4, 48.4, 21.6, 21.2. HRMS (LC/Q-TOF) (m/z) *calcd.* for $C_{26}H_{26}N_2O$ $[M+H]^+$ 383.2118; found 383.2120.

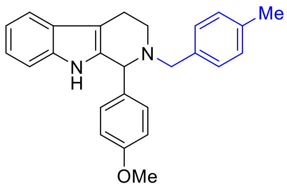

3ac (93 %)

3ac (71.2 mg, 93%) was obtained by using **General Procedure A** and purified by flash chromatography (silica gel, 10:1 hexane : EtOAc) in 93 % yield as white solid. ^{1}H NMR (400 MHz, $CDCl_3$) δ 7.61 – 7.56 (m, 1H), 7.42 – 7.37 (m, 2H), 7.35 (s, 1H), 7.30 (d, J = 7.9 Hz, 2H), 7.24 – 7.13 (m, 5H), 6.99 – 6.90 (m, 2H), 4.63 (d, J = 1.8 Hz, 1H), 3.92 (d, J = 13.4 Hz, 1H), 3.85 (s, 3H), 3.37 (d, J = 13.4 Hz, 1H), 3.28 (ddd, J = 11.7, 5.2, 3.8 Hz, 1H), 2.96 (dddd, J = 14.7, 9.3, 5.2, 2.0 Hz, 1H), 2.84 (dtd, J = 15.3, 4.1, 1.5 Hz, 1H), 2.70 (ddd, J = 11.7, 9.3, 4.3 Hz, 1H), 2.41 (s, 3H). ^{13}C NMR (101 MHz, $CDCl_3$) δ 159.4, 136.6, 136.5, 136.3, 135.3, 133.5, 130.2, 129.0, 128.8, 127.3, 121.5, 119.4, 118.3, 114.1, 110.9, 108.9, 63.8, 57.9, 55.4, 48.3, 21.3. HRMS (LC/Q-TOF) (m/z) *calcd.* for $C_{26}H_{26}N_2O$ $[M+H]^+$ 383.2118; found 383.2120.

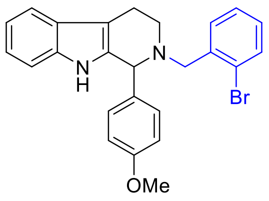

3ad (99 %)

3ad (88.6 mg, 99%) was obtained by using **General Procedure A** and purified by flash chromatography (silica gel, 10:1 hexane : EtOAc) in 99 % yield as white solid. ^{1}H NMR (600 MHz, $CDCl_3$) δ 7.68 (d, J = 7.2 Hz, 1H), 7.60 – 7.52 (m, 2H), 7.40 – 7.30 (m, 4H), 7.23 – 7.19 (m, 1H), 7.19 – 7.10 (m, 3H), 6.94 – 6.86 (m, 2H), 4.72 (s, 1H), 3.82 (s, 4H), 3.72 (d, J = 14.8 Hz, 1H), 3.23 (dt, J = 9.2, 3.9 Hz, 1H), 3.05 – 2.95 (m, 1H), 2.90 – 2.83 (m, 1H), 2.77 (s, 1H). ^{13}C NMR (151 MHz, $CDCl_3$) δ 159.5, 138.9, 136.3, 135.3, 133.1, 132.6, 130.2, 128.2, 127.5, 127.3, 124.3, 121.6, 119.4, 118.3, 114.1, 110.9, 108.9, 64.4, 57.3, 55.3, 48.8, 21.3. HRMS (LC/Q-TOF) (m/z) *calcd.* for $C_{25}H_{23}BrN_2O$ $[M+H]^+$ 447.1067; found 447.1079.

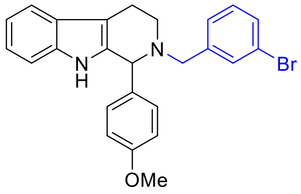

3ae (83 %)

3ae (74.3 mg, 83%) was obtained by using **General Procedure A** and purified by flash chromatography (silica gel, 10:1 hexane : EtOAc) in 83 % yield as white solid. ^{1}H NMR (400 MHz, $CDCl_3$) δ 7.55 – 7.48 (m, 2H), 7.39 – 7.25 (m, 5H), 7.20 – 7.07 (m, 4H), 6.95 – 6.86 (m, 2H), 4.59 (d, J = 1.8 Hz, 1H), 3.86 (d, J = 13.8 Hz, 1H), 3.82 (s, 3H), 3.31 (d, J = 13.8 Hz, 1H), 3.19 (ddd, J = 11.7, 5.2, 3.5 Hz, 1H), 2.93 (dddd, J = 15.0, 9.6, 5.3, 2.1 Hz, 1H), 2.84 – 2.74 (m, 1H), 2.65 (ddd, J = 11.7, 9.6, 4.3 Hz, 1H). ^{13}C NMR (101 MHz, $CDCl_3$) δ 159.5, 142.2, 136.3, 135.0, 133.0, 131.6, 130.2, 130.1, 129.9, 127.3, 127.2, 122.5, 121.6, 119.4, 118.4, 114.2, 110.9, 108.8, 64.1, 57.6, 55.4, 48.6, 21.3. HRMS (LC/Q-TOF) (m/z) *calcd.* for $C_{25}H_{23}BrN_2O$ $[M+H]^+$ 447.1067; found 447.1064.

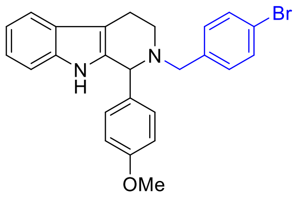

3af (95 %)

3af (85.0 mg, 95%) was obtained by using **General Procedure A** and purified by flash chromatography (silica gel, 10:1 hexane : EtOAc) in 95 % yield as white solid. ^{1}H NMR (600 MHz, $CDCl_3$) δ 7.56 – 7.52 (m, 1H), 7.47 – 7.44 (m, 2H), 7.36 – 7.33 (m, 2H), 7.29 (s, 1H), 7.24 (d, J = 8.0 Hz, 2H), 7.21 – 7.18 (m, 1H), 7.15 – 7.10 (m, 2H), 6.93 – 6.90 (m, 2H), 4.61 (s, 1H), 3.84 (d, J = 13.8 Hz, 1H), 3.82 (s, 3H), 3.32 (d, J = 13.8 Hz, 1H), 3.19 (dt, J = 12.0, 4.3 Hz, 1H), 2.92 (dt, J = 15.8, 9.0 Hz, 1H), 2.84 – 2.78 (m, 1H), 2.67 (s, 1H). ^{13}C NMR (151 MHz, $CDCl_3$) δ 159.6, 138.9, 136.4, 135.1, 133.1, 131.4, 130.5, 130.2, 127.3, 121.7, 120.7, 119.5, 118.4, 114.3, 110.9, 108.9, 64.1, 57.5, 55.4, 48.5, 21.3. HRMS (LC/Q-TOF) (m/z) *calcd.* for $C_{25}H_{23}BrN_2O$ $[M+H]^+$ 447.1067; found 447.1083.

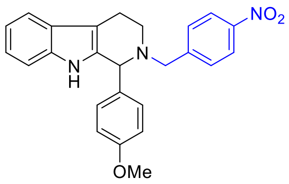

3ag (88 %)

3ag (72.8 mg, 88%) was obtained by using **General Procedure A** and purified by flash chromatography (silica gel, 10:1 hexane : EtOAc) in 88 % yield as yellow solid. ^{1}H NMR (600 MHz, $CDCl_3$) δ 8.21 – 8.10 (m, 2H), 7.59 – 7.46 (m, 3H), 7.35 (d, J = 8.3 Hz, 2H), 7.30 (s, 1H), 7.23 – 7.17 (m, 1H), 7.12 (pd, J = 7.1, 1.4 Hz, 2H), 6.96 – 6.86 (m, 2H), 4.63 (s, 1H), 3.96 (d, J = 14.5 Hz, 1H), 3.81 (s, 3H), 3.46 (d, J = 14.6 Hz, 1H), 3.14 (d, J = 11.6 Hz, 1H), 3.00 – 2.90 (m, 1H), 2.81 (d, J = 15.3 Hz, 1H), 2.70 (s, 1H). ^{13}C NMR (151 MHz, $CDCl_3$) δ 159.8, 148.0, 147.2, 136.4, 134.9, 132.8, 130.3, 129.2, 127.2, 123.7, 121.8, 119.6, 118.4, 114.4, 111.0, 108.8, 64.5, 57.7, 55.4, 49.1, 21.5. HRMS (LC/Q-TOF) (m/z) *calcd.* for $C_{25}H_{23}N_3O_3$ $[M+H]^+$ 414.1812; found 414.1819.

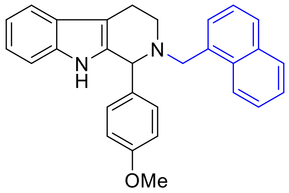

3ah (52 %)

3ah (43.5 mg, 52%) was obtained by using **General Procedure A** and purified by flash chromatography (silica gel, 10:1 hexane : EtOAc) in 52 % yield as white solid. ^{1}H NMR (400 MHz, $CDCl_3$) δ 8.11 (d, J = 8.3 Hz, 1H), 7.89 (d, J = 7.9 Hz, 1H), 7.83 (d, J = 8.1 Hz, 1H), 7.70 – 7.43 (m, 6H), 7.43 – 7.34 (m, 3H), 7.25 – 7.14 (m, 3H), 6.94 (dt, J = 8.6, 2.3 Hz, 2H), 4.70 (s, 1H), 4.37 (d, J = 13.8 Hz, 1H), 3.84 (s, 3H), 3.33 – 3.21 (m, 1H), 2.88 (dddd, J = 9.9, 8.1, 4.5, 1.8 Hz, 2H), 2.78 – 2.69 (m, 1H). ^{13}C NMR (101 MHz, $CDCl_3$) δ 159.5, 136.3, 135.2, 135.1, 133.9, 133.3, 132.5, 130.6, 128.4, 127.8, 127.3, 127.3, 125.6, 125.3, 124.8, 121.6, 119.4, 118.4, 114.0, 110.9, 109.3, 64.6, 56.5, 55.4, 48.2, 21.0. HRMS (LC/Q-TOF) (m/z) *calcd.* for $C_{29}H_{26}N_2O$ $[M+H]^+$ 419.2118; found 419.2121.

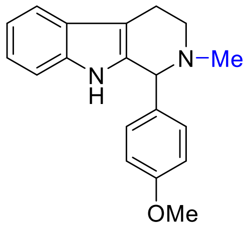

3ai (90 %)

3ai (52.6 mg, 90%) was obtained by using **General Procedure A** and purified by flash chromatography (silica gel, 2:1 hexane : EtOAc) in 90 % yield as white solid. ^{1}H NMR (400 MHz, $CDCl_3$) δ 7.55 (tt, J = 4.4, 2.2 Hz, 1H), 7.42 – 7.33 (m, 1H), 7.29 – 7.25 (m, 2H), 7.21 – 7.16 (m, 1H), 7.16 – 7.09 (m, 2H), 6.93 – 6.87 (m, 2H), 4.31 (d, J = 2.0 Hz, 1H), 3.83 (s, 3H), 3.26 (ddd, J = 11.5, 5.4, 2.5 Hz, 1H), 3.13 (dddd, J = 15.6, 10.4, 5.4, 2.2 Hz, 1H), 2.88 (ddt, J = 15.3, 4.1, 2.0 Hz, 1H), 2.78 (ddd, J = 11.5, 10.4, 4.3 Hz, 1H), 2.35 (s, 3H). ^{13}C NMR (101 MHz, $CDCl_3$) δ 159.6, 136.4, 135.0, 132.4, 130.4, 127.2, 121.6, 119.4, 118.4, 114.1, 110.9, 108.8, 66.7, 55.4, 53.2, 43.4, 21.6. HRMS (LC/Q-TOF) (m/z) *calcd.* for $C_{19}H_{20}N_2O$ $[M+H]^+$ 293.1648; found 293.1652.

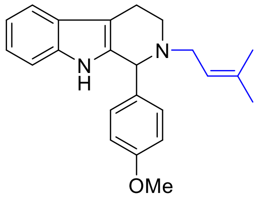

3aj (72 %)

3aj (49.9 mg, 72%) was obtained by using **General Procedure A** and purified by flash chromatography (silica gel, 10:1 hexane : EtOAc) in 72 % yield as yellow solid. ^{1}H NMR (500 MHz, $CDCl_3$) δ 7.57 – 7.50 (m, 1H), 7.31 (h, J = 3.7 Hz, 1H), 7.29 – 7.24 (m, 2H), 7.20 – 7.15 (m, 1H), 7.14 – 7.06 (m, 2H), 6.90 – 6.83 (m, 2H), 5.35 (td, J = 6.0, 2.6 Hz,

1H), 4.60 (s, 1H), 3.81 (s, 3H), 3.33 (ddd, J = 11.8, 5.3, 3.8 Hz, 1H), 3.24 (dd, J = 14.0, 5.9 Hz, 1H), 2.99 (dq, J = 13.3, 4.9 Hz, 2H), 2.86 (dtd, J = 15.4, 4.2, 1.6 Hz, 1H), 2.78 – 2.68 (m, 1H), 1.79 – 1.73 (m, 3H), 1.57 (s, 3H). ^{13}C NMR (126 MHz, $CDCl_3$) δ 159.5, 136.4, 135.6, 135.0, 133.0, 130.4, 127.3, 121.5, 121.3, 119.4, 118.4, 114.1, 110.9, 109.1, 63.6, 55.4, 51.6, 48.4, 26.1, 21.2, 18.2.HRMS (LC/Q-TOF) (m/z) *calcd.* for $C_{23}H_{26}N_2O$ $[M+H]^+$ 347.2118; found 347.2122.

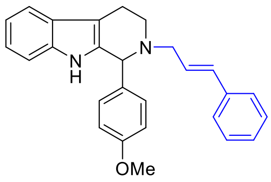

3ak (99 %)

3ak (78.1 mg, 99%) was obtained by using **General Procedure A** and purified by flash chromatography (silica gel, 10:1 hexane : EtOAc) in 99 % yield as white solid. ^{1}H NMR (500 MHz, $CDCl_3$) δ 7.62 – 7.57 (m, 1H), 7.47 – 7.43 (m, 2H), 7.40 – 7.33 (m, 5H), 7.32 – 7.27 (m, 1H), 7.24 – 7.20 (m, 1H), 7.20 – 7.13 (m, 2H), 7.00 – 6.88 (m, 2H), 6.58 (d, J = 15.9 Hz, 1H), 6.39 (ddd, J = 15.9, 7.8, 5.4 Hz, 1H), 4.72 (s, 1H), 3.86 (s, 3H), 3.55 (ddd, J = 14.0, 5.4, 1.7 Hz, 1H), 3.47 – 3.41 (m, 1H), 3.20 (dd, J = 14.1, 7.8 Hz, 1H), 3.06 (dddd, J = 14.5, 9.2, 5.1, 1.9 Hz, 1H), 2.93 (dtd, J = 15.3, 4.2, 1.5 Hz, 1H), 2.89 – 2.80 (m, 1H). ^{13}C NMR (126 MHz, $CDCl_3$) δ 159.6, 137.1, 136.4, 134.9, 133.0, 132.8, 130.4, 128.7, 127.5, 127.3, 126.4, 121.6, 119.4, 118.4, 114.2, 110.9, 109.0, 63.4, 56.5, 55.4, 48.5, 21.1. HRMS (LC/Q-TOF) (m/z) *calcd.* for $C_{27}H_{26}N_2O$ $[M+H]^+$ 395.2118; found 395.2122.

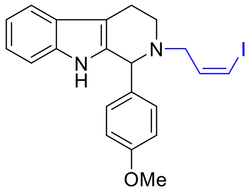

3al (34 %)

3al (30.2 mg, 34%) was obtained by using **General Procedure A** and purified by flash chromatography (silica gel, 10:1 hexane : EtOAc) in 34 % yield as white solid. ^{1}H NMR (400 MHz, $CDCl_3$) δ 7.57 – 7.51 (m, 1H), 7.36 (s, 1H), 7.30 – 7.26 (m, 2H), 7.18 (ddt, J = 7.0, 3.3, 1.7 Hz, 1H), 7.15 – 7.08 (m, 2H), 6.93 – 6.85 (m, 2H), 6.45 – 6.34 (m, 2H), 4.62 (s, 1H), 3.82 (s, 3H), 3.40 – 3.25 (m, 2H), 3.23 – 3.12 (m, 1H), 3.01 (dddd, J = 14.4, 9.0, 5.1, 1.9 Hz, 1H), 2.91 – 2.77 (m, 2H). ^{13}C NMR (101 MHz, $CDCl_3$) δ 159.6, 138.4, 136.3, 134.7, 132.3, 130.5, 127.2, 121.7, 119.5, 118.4, 114.2, 110.9, 108.9, 84.1, 63.9, 58.1, 55.4, 49.1, 21.3. HRMS (LC/Q-TOF) (m/z) *calcd.* for $C_{21}H_{21}IN_2O$ $[M+H]^+$ 445.0771; found 445.0776.

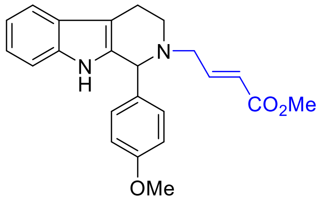

3am (28 %)

3am (21.1 mg, 28%) was obtained by using **General Procedure A** and purified by flash chromatography (silica gel, 10:1 hexane : EtOAc) in 28 % yield as yellow solid. ^{1}H NMR (400 MHz, $CDCl_3$) δ 7.54 – 7.48 (m, 1H), 7.32 – 7.25 (m, 3H), 7.19 – 7.15 (m, 1H), 7.12 – 7.06 (m, 2H), 6.89 – 6.82 (m, 2H), 6.27 (d, J = 2.0 Hz, 1H), 5.96 (d, J = 2.0 Hz, 1H), 4.61 (s, 1H), 3.79 (d, J = 0.8 Hz, 3H), 3.70 (d, J = 0.8 Hz, 3H), 3.49 – 3.43 (m, 1H), 3.29 – 3.22 (m, 1H), 3.19 (d, J = 15.7 Hz, 1H), 2.95 (dddd, J = 15.0, 9.8, 5.3, 2.0 Hz, 1H), 2.81 (dt, J = 15.3, 4.0 Hz, 1H), 2.65 (ddd, J = 11.5, 9.6, 4.1 Hz, 1H). ^{13}C NMR (101 MHz, $CDCl_3$) δ 167.6, 159.5, 138.1, 136.3, 135.4, 133.0, 130.2,

127.2, 126.2, 121.6, 119.4, 118.3, 114.1, 110.9, 108.8, 64.3, 55.4, 54.0, 51.9, 48.9, 21.5. HRMS (LC/Q-TOF) (m/z) *calcd.* for $C_{23}H_{24}N_2O_3$ $[M+H]^+$ 377.1860; found 377.1866.

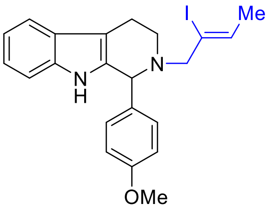

3an (30 %)

3an (27.5 mg, 30%) was obtained by using **General Procedure A** and purified by flash chromatography (silica gel, 10:1 hexane : EtOAc) in 30 % yield as white solid. ^{1}H NMR (400 MHz, $CDCl_3$) δ 7.55 (dt, J = 7.8, 2.9 Hz, 1H), 7.41 – 7.31 (m, 3H), 7.21 – 7.09 (m, 3H), 6.95 – 6.85 (m, 2H), 5.98 – 5.88 (m, 1H), 4.62 (d, J = 1.8 Hz, 1H), 3.82 (s, 3H), 3.49 (dt, J = 13.9, 1.7 Hz, 1H), 3.28 (dt, J = 11.8, 4.8 Hz, 1H), 3.11 (d, J = 14.0 Hz, 1H), 2.96 (dddd, J = 15.6, 8.7, 5.2, 1.9 Hz, 1H), 2.84 (dtd, J = 15.3, 4.5, 1.5 Hz, 1H), 2.64 (ddd, J = 11.8, 8.6, 4.4 Hz, 1H), 1.83 (dd, J = 6.3, 1.5 Hz, 3H). ^{13}C NMR (101 MHz, $CDCl_3$) δ 159.4, 136.2, 135.0, 132.8, 132.0, 130.5, 127.3, 121.6, 119.4, 118.3, 114.0, 110.9, 109.6, 109.0, 65.2, 63.0, 55.4, 47.3, 22.0, 21.0. HRMS (LC/Q-TOF) (m/z) *calcd.* for $C_{22}H_{23}IN_2O$ $[M+H]^+$ 459.0928; found 459.0930.

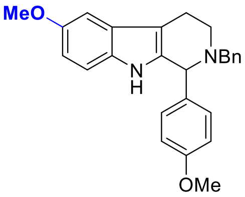

3ao (80 %)

3ao (63.8 mg, 80%) was obtained by using **General Procedure A** and purified by flash chromatography (silica gel, 10:1 hexane : EtOAc) in 80 % yield as white solid. ^{1}H NMR (600 MHz, $CDCl_3$) δ 7.39 – 7.33 (m, 6H), 7.30 – 7.27 (m, 1H), 7.23 (s, 1H), 7.08 (d, J = 8.7 Hz, 1H), 7.00 (d, J = 2.5 Hz, 1H), 6.93 – 6.90 (m, 2H), 6.79 (dd, J = 8.7, 2.5 Hz, 1H), 4.62 (s, 1H), 3.92 (d, J = 13.5 Hz, 1H), 3.87 (s, 3H), 3.82 (s, 3H), 3.38 (d, J = 13.6 Hz, 1H), 3.31 – 3.20 (m, 1H), 2.91 (d, J = 11.3 Hz, 1H), 2.83 – 2.75 (m, 1H), 2.69 (s, 1H). ^{13}C NMR (151 MHz, $CDCl_3$) δ 159.5, 154.1, 139.6, 136.2, 133.4, 131.5, 130.3, 128.9, 128.3, 127.7, 127.1, 114.2, 111.6, 111.4, 108.8, 100.7, 64.1, 58.2, 56.1, 55.4, 48.4, 21.3. HRMS (LC/Q-TOF) (m/z) *calcd.* for $C_{26}H_{26}N_2O_2$ $[M+H]^+$ 399.2067; found 399.2071.

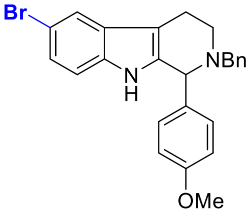

3ap (37 %)

3ap (33.1 mg, 37%) was obtained by using **General Procedure A** and purified by flash chromatography (silica gel, 10:1 hexane : EtOAc) in 37 % yield as white solid. ^{1}H NMR (500 MHz, DMSO-d6) δ 10.51 (s, 1H), 7.58 (s, 1H), 7.31 (d, J = 5.7 Hz, 4H), 7.26 (d, J = 8.4 Hz, 3H), 7.19 (d, J = 8.5 Hz, 1H), 7.14 – 7.08 (m, 1H), 6.92 (d, J = 8.1 Hz, 2H), 4.67 (s, 1H), 3.73 (s, 3H), 3.70 (d, J = 13.6 Hz, 1H), 3.41 (d, J = 14.0 Hz, 1H), 3.00 (dd, J = 11.4, 5.7 Hz, 1H), 2.71 (p, J = 8.5, 6.6 Hz, 2H), 2.56 (p, J = 5.8 Hz, 1H). ^{13}C NMR (126 MHz, DMSO-d6) δ 158.7, 139.4, 136.9, 135.1, 133.2, 130.0, 128.4, 128.3, 128.2, 126.9, 122.9, 120.0, 113.7, 113.1, 110.9, 107.0, 62.2, 57.1, 55.1, 46.5, 20.3. HRMS (LC/Q-TOF) (m/z) *calcd.* for $C_{25}H_{23}BrN_2O$ $[M+H]^+$ 447.1067; found 447.1075.

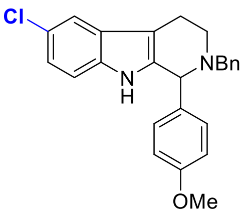

3aq (40 %)

3aq (32.2 mg, 40%) was obtained by using **General Procedure A** and purified by flash chromatography (silica gel, 10:1 hexane : EtOAc) in 40 % yield as white solid. ^{1}H NMR (500 MHz, $CDCl_3$) δ 7.51 – 7.45 (m, 1H), 7.38 – 7.32 (m, 7H), 7.30 – 7.26 (m, 1H), 7.10 – 7.03 (m, 2H), 6.95 – 6.88 (m, 2H), 4.60 (s, 1H), 3.91 (d, J = 13.5 Hz, 1H), 3.82 (s, 3H), 3.36 (d, J = 13.5 Hz, 1H), 3.28 – 3.19 (m, 1H), 2.94 – 2.83 (m, 1H), 2.78 – 2.70 (m, 1H), 2.65 (ddd, J = 14.1, 9.4, 4.1 Hz, 1H). ^{13}C NMR (126 MHz, $CDCl_3$) δ 159.6, 139.4, 136.8, 134.7, 132.9, 130.2, 128.9, 128.5, 128.4, 127.1, 125.1, 121.7, 118.0, 114.3, 111.8, 108.8, 64.0, 58.2, 55.4, 48.4, 21.2. HRMS (LC/Q-TOF) (m/z) *calcd.* for $C_{25}H_{23}ClN_2O$ $[M+H]^+$ 403.1572; found 403.1578.

3.3.2 Gram-scale synthesis of C1-aryl THβC 3f

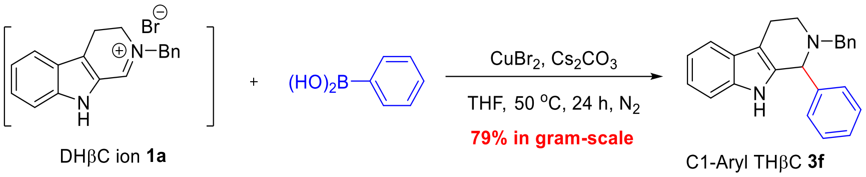

To a flame-dried Schlenk tube were sequentially added DHβC ion **1a** (1.71 g, 5.0 mmol), $CuBr_2$ (223 mg, 20 % mmol), Cs_2CO_3 (3.26 g, 10.0 mmol), phenylboronic acid (1.22 g, 10.0 mmol), 5 Å molecular sieves (2.50 g) and THF (100.0 mL, 0.05 M) under a nitrogen atmosphere. The reaction mixture was placed in a pre-heated oil bath at 50 ºC for 24 h. After cooling to room temperature, the crude reaction was filtrated through a short pad of silica gel washed with EtOAc (50 mL). After solvent evaporation, the residue was purified by flash column chromatography on silica gel (Hexane/EtOAc = 25/1 to 10/1) to afford desired products **3f** (1.33 g, 79%) as white solid.

3.3.3 Petasis-type reaction of *N*-alkyl DHβC ions with alkenyl boronic esters

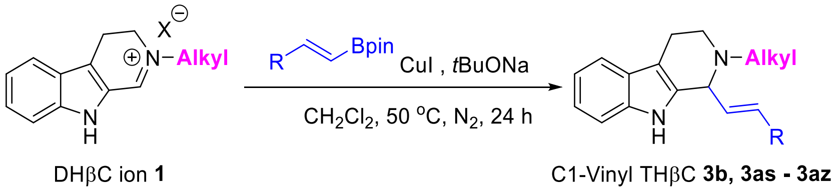

General Procedure B: To a flame-dried Schlenk tube were sequentially added DHβC ion **1** (0.2 mmol), CuI (7.6 mg, 20 % mmol), *t*BuONa (38.5 mg, 0.4 mmol), vinylboronic acid pinacol ester (0.4 mmol), 5 Å molecular sieves (100 mg) and CH_2Cl_2 (4.0 mL, 0.05 M) under a nitrogen atmosphere. The reaction mixture in Schlenk tube was placed in a pre-heated oil bath at 50 ºC for 24 h. After cooling to room temperature, the crude reaction was filtrated through a short pad of silica gel washed with EtOAc (25 mL). After solvent evaporation, the residue was purified by chromatography on silica gel to afford C1-alkenyl THβCs **3b, 3as – 3az**.

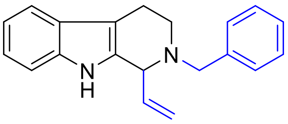

3b (92%)

3b (53.1 mg, 92%) was obtained by using **General Procedure B** and purified by flash chromatography (silica gel, 4:1 hexane : EtOAc) in 92 % yield as yellow oil. ^{1}H NMR (400 MHz, $CDCl_3$) δ 7.70 (s, 1H), 7.51 (d, J = 7.6 Hz, 1H), 7.43 (d, J = 7.0 Hz, 2H), 7.39 – 7.28 (m, 4H), 7.20 – 7.08 (m, 2H), 6.02 (ddd, J = 17.2, 10.1, 8.3 Hz, 1H), 5.50 – 5.39 (m, 2H), 4.21 – 4.08 (m, 2H), 3.51 (d, J = 13.7 Hz, 1H), 3.20 (dt, J = 11.8, 4.8 Hz, 1H), 2.86 – 2.72 (m, 2H), 2.63 (ddd, J = 11.8, 8.1, 5.0 Hz, 1H). ^{13}C NMR (101 MHz, $CDCl_3$) δ 139.2, 138.9, 136.2, 132.9, 129.0, 128.4, 127.6, 127.1, 121.7, 119.5, 119.2, 118.4, 110.9, 108.9, 63.3, 58.4, 47.9, 21.1. HRMS (LC/Q-TOF) (m/z) *calcd.* for $C_{20}H_{20}N_2$ $[M+H]^+$ 289.1699; found 289.1706.

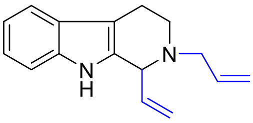

3as (86%)

3as (41.0 mg, 86%) was obtained by using **General Procedure B** and purified by flash chromatography (silica gel, 4:1 hexane : EtOAc) in 86 % yield as yellow oil. ^{1}H NMR (400 MHz, $CDCl_3$) δ 7.76 (s, 1H), 7.53 (dd, J = 7.6, 1.4 Hz, 1H), 7.34 – 7.27 (m, 1H), 7.16 (dtd, J = 18.4, 7.1, 1.3 Hz, 2H), 6.08 – 5.85 (m, 2H), 5.47 – 5.35 (m, 2H), 5.33 – 5.22 (m, 2H), 4.14 (dd, J = 8.4, 1.8 Hz, 1H), 3.55 (ddt, J = 13.9, 5.3, 1.7 Hz, 1H), 3.28 (dt, J = 11.8, 4.8 Hz, 1H), 3.11 (ddd, J = 13.8, 7.7, 1.1 Hz, 1H), 2.91 – 2.78 (m, 2H), 2.68 (ddd, J = 11.7, 8.2, 4.8 Hz, 1H). ^{13}C NMR (101 MHz, $CDCl_3$) δ 138.3, 136.2, 135.4, 132.9, 127.5, 121.6, 119.4, 119.2, 118.3, 118.1, 110.9, 108.7, 62.9, 57.2, 47.6, 21.0. HRMS (LC/Q-TOF) (m/z) *calcd.* for $C_{16}H_{18}N_2$ $[M+H]^+$ 239.1543; found 239.1549.

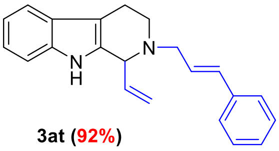

3at (92%)

3at (57.9 mg, 92%) was obtained by using **General Procedure B** and purified by flash chromatography (silica gel, 4:1 hexane : EtOAc) in 92 % yield as yellow oil. ^{1}H NMR (400 MHz, $CDCl_3$) δ 7.74 (s, 1H), 7.54 (d, J = 7.5 Hz, 1H), 7.44 (d, J = 7.2 Hz, 2H), 7.39 – 7.27 (m, 4H), 7.16 (dtd, J = 19.8, 7.2, 1.3 Hz, 2H), 6.61 (d, J = 15.9 Hz, 1H), 6.41 (ddd, J = 15.9, 7.8, 5.4 Hz, 1H), 5.98 (ddd, J = 17.8, 9.7, 8.4 Hz, 1H), 5.44 (ddt, J = 13.3, 2.1, 1.3 Hz, 2H), 4.21 (d, J = 8.4 Hz, 1H), 3.76 – 3.68 (m, 1H), 3.35 (dt, J = 11.7, 4.8 Hz, 1H), 3.27 (dd, J = 14.3, 7.5 Hz, 1H), 2.93 – 2.79 (m, 2H), 2.74 (ddd, J = 11.7, 8.3, 4.7 Hz, 1H). ^{13}C NMR (101 MHz, $CDCl_3$) δ 138.3, 137.1, 136.2, 133.1, 132.8, 128.7, 127.6, 127.6, 127.0, 126.4, 121.7, 119.43, 119.37, 118.4, 110.9, 108.7, 63.1, 56.7, 47.9, 21.0. HRMS (LC/Q-TOF) (m/z) *calcd.* for $C_{22}H_{22}N_2$ $[M+H]^+$ 315.1856; found 315.1864.

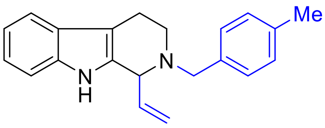

3au (93%)

3au (56.3 mg, 93%) was obtained by using **General Procedure B** and purified by flash chromatography (silica gel, 4:1 hexane : EtOAc) in 93 % yield as yellow oil. ^{1}H NMR (400 MHz, $CDCl_3$) δ 7.70 (s, 1H), 7.57 (dd, J = 7.5, 1.3 Hz, 1H), 7.36 (dd, J = 13.1, 7.7 Hz, 3H), 7.26 – 7.14 (m, 4H), 6.14 – 5.99 (m, 1H), 5.54 – 5.43 (m, 2H), 4.23 – 4.17 (m, 1H), 4.14 (d, J = 13.5 Hz, 1H), 3.53 (d, J = 13.5 Hz, 1H), 3.25 (dt, J = 11.7, 4.8 Hz, 1H), 2.90

– 2.77 (m, 2H), 2.67 (ddd, J = 11.7, 7.9, 5.1 Hz, 1H), 2.44 (s, 3H). ^{13}C NMR (101 MHz, $CDCl_3$) δ 139.0, 136.6, 136.2, 136.1, 133.0, 129.1, 129.0, 127.6, 121.6, 119.4, 119.0, 118.4, 110.9, 108.8, 63.1, 58.1, 47.7, 21.2, 21.1. HRMS (LC/Q-TOF) (m/z) *calcd.* for $C_{21}H_{22}N_2$ $[M+H]^+$ 303.1856; found 303.1861.

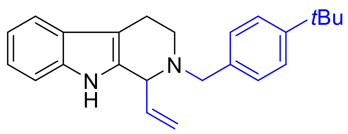

3av (93%)

3av (64.1 mg, 93%) was obtained by using **General Procedure B** and purified by flash chromatography (silica gel, 4:1 hexane : EtOAc) in 93 % yield as yellow oil. ^{1}H NMR (400 MHz, $CDCl_3$) δ 7.70 (s, 1H), 7.54 (dd, J = 7.6, 1.3 Hz, 1H), 7.45 – 7.37 (m, 4H), 7.33 (dt, J = 8.1, 1.0 Hz, 1H), 7.17 (dtd, J = 20.1, 7.1, 1.3 Hz, 2H), 6.04 (ddd, J = 17.1, 10.2, 8.3 Hz, 1H), 5.51 – 5.42 (m, 2H), 4.23 – 4.17 (m, 1H), 4.13 (d, J = 13.7 Hz, 1H), 3.53 (d, J = 13.7 Hz, 1H), 3.25 (dt, J = 11.7, 4.8 Hz, 1H), 2.89 – 2.75 (m, 2H), 2.65 (ddd, J = 11.7, 8.2, 4.9 Hz, 1H), 1.40 (s, 9H). ^{13}C NMR (101 MHz, $CDCl_3$) δ 150.0, 139.0, 136.2, 136.0, 133.0, 128.7, 127.6, 125.3, 121.6, 119.4, 119.1, 118.4, 110.9, 108.8, 63.2, 58.0, 47.8, 34.6, 31.6, 21.1. HRMS (LC/Q-TOF) (m/z) *calcd.* for $C_{24}H_{28}N_2$ $[M+H]^+$ 345.2325; found 345.2333.

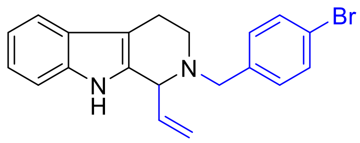

3aw (88%)

3aw (64.6 mg, 88%) was obtained by using **General Procedure B** and purified by flash chromatography (silica gel, 4:1 hexane : EtOAc) in 88 % yield as yellow oil. ^{1}H NMR (400 MHz, $CDCl_3$) δ 7.69 (s, 1H), 7.57 – 7.45 (m, 3H), 7.38 – 7.28 (m, 3H), 7.16 (dtd, J = 21.2, 7.2, 1.3 Hz, 2H), 6.11 – 5.87 (m, 1H), 5.51 – 5.39 (m, 2H), 4.15 (dd, J = 8.3, 1.9 Hz, 1H), 4.06 (d, J = 13.8 Hz, 1H), 3.46 (d, J = 13.9 Hz, 1H), 3.15 (dt, J = 11.7, 4.8 Hz, 1H), 2.87 – 2.73 (m, 2H), 2.62 (ddd, J = 11.7, 7.7, 5.2 Hz, 1H). ^{13}C NMR (101 MHz, $CDCl_3$) δ 138.7, 138.4, 136.2, 132.8, 131.5, 130.6, 127.6, 121.7, 120.8, 119.5, 119.3, 118.4, 110.9, 108.8, 63.3, 57.7, 47.9, 21.1. HRMS (LC/Q-TOF) (m/z) *calcd.* for $C_{20}H_{19}BrN_2$ $[M+H]^+$ 367.0804; found 367.0812.

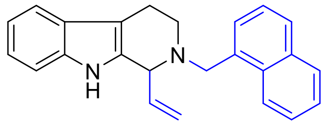

3ax (75%)

3ax (50.8 mg, 75%) was obtained by using **General Procedure B** and purified by flash chromatography (silica gel, 4:1 hexane : EtOAc) in 75 % yield as yellow oil. ^{1}H NMR (400 MHz, $CDCl_3$) δ 8.36 (dd, J = 6.4, 3.4 Hz, 1H), 7.93 – 7.87 (m, 1H), 7.83 (d, J = 8.2 Hz, 1H), 7.73 (s, 1H), 7.62 (d, J = 7.0 Hz, 1H), 7.56 – 7.46 (m, 4H), 7.34 (d, J = 8.0 Hz, 1H), 7.16 (dtd, J = 23.3, 7.2, 1.2 Hz, 2H), 6.15 (ddd, J = 17.2, 10.0, 8.3 Hz, 1H), 5.55 – 5.44 (m, 2H), 4.61 (d, J = 13.6 Hz, 1H), 4.25 (d, J = 8.3 Hz, 1H), 3.87 (d, J = 13.6 Hz, 1H), 3.21 (dt, J = 11.6, 4.6 Hz, 1H), 2.77 (dqd, J = 9.1, 4.9, 1.8 Hz, 2H), 2.67 (ddd, J = 12.4, 7.0, 5.5 Hz, 1H). ^{13}C NMR (101 MHz, $CDCl_3$) δ 138.7, 136.2, 135.0, 134.0, 133.0, 132.5, 128.6, 128.0, 127.6, 127.4, 125.8, 125.7, 125.4, 124.7, 121.7, 119.5, 119.3, 118.4, 110.9, 109.0, 64.2, 56.7, 47.8, 21.0. HRMS (LC/Q-TOF) (m/z) *calcd.* for $C_{24}H_{22}N_2$ $[M+H]^+$ 339.1856;

found 339.1863.

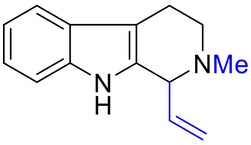

3ay (73%)

3ay (31.0 mg, 73%) was obtained by using **General Procedure B** and purified by flash chromatography (silica gel, 4:1 hexane : EtOAc) in 73 % yield as yellow oil. ^{1}H NMR (400 MHz, $CDCl_3$) δ 7.75 (s, 1H), 7.50 (dd, J = 7.8, 1.2 Hz, 1H), 7.30 (dt, J = 7.9, 0.8 Hz, 1H), 7.15 (ddd, J = 8.1, 7.0, 1.3 Hz, 1H), 7.10 (td, J = 7.5, 1.1 Hz, 1H), 5.86 (ddd, J = 17.1, 9.9, 8.7 Hz, 1H), 5.51 – 5.36 (m, 2H), 3.84 (dt, J = 8.8, 1.9 Hz, 1H), 3.18 (ddd, J = 11.6, 5.5, 3.0 Hz, 1H), 2.96 (dddd, J = 15.5, 10.2, 5.5, 2.2 Hz, 1H), 2.80 (dddd, J = 15.4, 4.5, 3.0, 1.7 Hz, 1H), 2.66 (ddd, J = 11.6, 10.2, 4.4 Hz, 1H), 2.49 (s, 3H). ^{13}C NMR (101 MHz, $CDCl_3$) δ 138.3, 136.3, 132.8, 127.6, 121.7, 119.6, 119.5, 118.4, 111.0, 108.3, 66.1, 52.5, 43.4, 21.4. HRMS (LC/Q-TOF) (m/z) *calcd.* for $C_{14}H_{16}N_2$ $[M+H]^+$ 213.1386; found 213.1391.

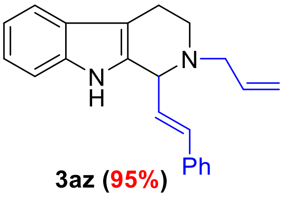

3az (95%)

3az (59.7 mg, 95%) was obtained by using **General Procedure B** and purified by flash chromatography (silica gel, 4:1 hexane : EtOAc) in 95 % yield as yellow oil. ^{1}H NMR (400 MHz, $CDCl_3$) δ 7.79 (s, 1H), 7.56 – 7.50 (m, 1H), 7.47 – 7.40 (m, 2H), 7.40 – 7.32 (m, 2H), 7.32 – 7.26 (m, 2H), 7.13 (dtd, J = 16.9, 7.2, 1.3 Hz, 2H), 6.71 (d, J = 15.9 Hz, 1H), 6.33 (dd, J = 15.9, 8.5 Hz, 1H), 6.01 (dddd, J = 17.6, 10.1, 7.7, 5.2 Hz, 1H), 5.33 – 5.19 (m, 2H), 4.34 (d, J = 8.6 Hz, 1H), 3.60 (ddt, J = 13.8, 5.2, 1.7 Hz, 1H), 3.33 (dt, J = 11.8, 4.8 Hz, 1H), 3.16 (dd, J = 13.9, 7.7 Hz, 1H), 2.99 – 2.79 (m, 2H), 2.73 (ddd, J = 11.8, 8.5, 4.6 Hz, 1H). ^{13}C NMR (101 MHz, $CDCl_3$) δ 136.3, 136.2, 135.1, 134.4, 132.9, 129.1, 128.9, 128.3, 127.5, 126.8, 121.8, 119.5, 118.5, 118.4, 111.0, 108.8, 62.4, 57.5, 47.9, 21.1. HRMS (LC/Q-TOF) (m/z) *calcd.* for $C_{22}H_{22}N_2$ $[M+H]^+$ 315.1856; found 315.1864.

3.4 Derivatizations of C1-Aryl THβC 3f

3.4.1 Oxidative ring-opening [8]

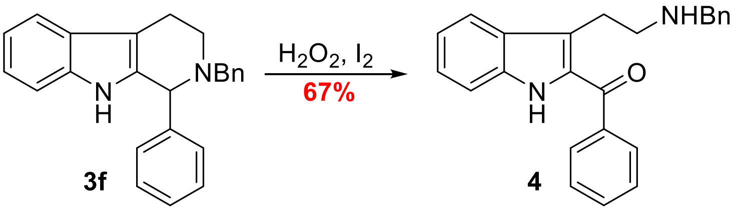

To a solution of compound **3f** (67.69 mg, 0.2 mmol) in EtOH (4.0 mL, 0.05 M) at room temperature was added I_2 (15.23 mg, 0.06 mmol) and 30% *aq.* H_2O_2 (40 μL, 0.4 mmol). The reaction mixture was stirred at 40 °C for 4 - 8 h and then was cooled to room temperature. After TLC analysis indicated the completion of the reaction, the reaction was quenched at 0 °C by addition of *aq. sat.* $Na_2S_2O_3$ (10 mL) and extracted with EtOAc (20 mL×3). The organic layer

was dried over Na_2SO_4, filtered, and concentrated under reduced pressure. After solvent evaporation, the residue was purified by flash column chromatography on silica gel (Hexane/EtOAc 3/1 to 1/1) to provide the desired product **4** (47.5 mg, 67%) as yellow solid. ^{1}H NMR (600 MHz, $CDCl_3$) δ 10.76 (s, 1H), 7.87 (dd, J = 8.6, 1.0 Hz, 1H), 7.79 – 7.75 (m, 2H), 7.44 (d, J = 8.3 Hz, 1H), 7.40 – 7.35 (m, 1H), 7.35 – 7.27 (m, 6H), 7.18 – 7.14 (m, 2H), 7.11 (ddd, J = 8.0, 6.9, 0.9 Hz, 1H), 5.16 (s, 2H), 4.38 (t, J = 8.6 Hz, 2H), 3.13 (t, J = 8.6 Hz, 2H), 1.23 (s, 1H). ^{13}C NMR (151 MHz, $CDCl_3$) δ 165.0, 143.1, 133.7, 132.9, 130.0, 129.52, 129.47, 129.2, 129.22, 128.1, 127.5, 126.8, 124.6, 124.2, 122.3, 121.4, 115.2, 59.1, 50.9, 20.4. HRMS (LC/Q-TOF) (m/z) *calcd.* for $C_{24}H_{22}N_2O$ $[M+H]^+$ 355.1805, Found 355.1802.

3.4.2 Oxidative rearrangement[9]

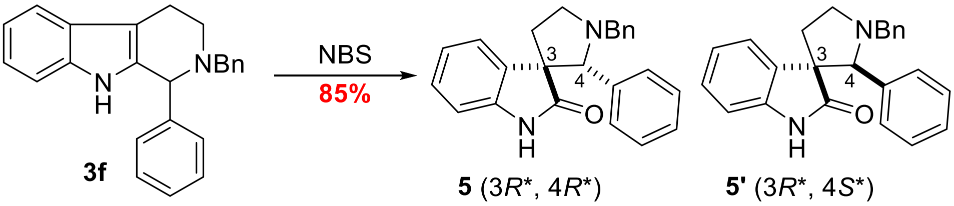

3f **5** (3*R**, 4*R**) **5'** (3*R**, 4*S**)

(**5**/**5'** = 5:1)[c]

To a solution of compound **3f** (67.69 mg, 0.2 mmol) in THF/AcOH/H_2O (1/1/1 – 4.0 mL 0.05 M) at 0°C was added N-Bromosuccinimide (NBS, 7.10 mg, 0.04 mmol). The reaction mixture was stirred at 0 °C for 20 min and then was added NBS (35.60 mg, 0.2 mmol) in several portions for 60 minutes. After TLC analysis indicated the completion of the reaction, the reaction was quenched at 0 °C by addition of *aq. sat.* $NaHCO_3$ (10 mL) and extracted with EtOAc (20 mL×3). The organic layer was dried over Na_2SO_4, filtered, and concentrated under reduced pressure. After solvent evaporation, the residue was purified by flash column chromatography on silica gel (Hexane/EtOAc = 6/1 to 2/1) to provide the desired product **5 & 5'**[10] (4 :4' = 5:1, inseparable) (60.2 mg, 85%) as blue solid. ^{1}H NMR (500 MHz, $CDCl_3$) δ 8.39 (s, 1H), 7.49 (d, J = 7.3 Hz, 1H), 7.45 (d, J = 7.6 Hz, 2H), 7.39 (t, J = 7.5 Hz, 2H), 7.29 (t, J = 7.2 Hz, 1H), 7.26 – 7.20 (m, 2H), 7.09 – 7.01 (m, 4H), 6.97 (t, J = 7.1 Hz, 1H), 6.63 (d, J = 7.5 Hz, 1H), 4.15 (s, 1H), 4.05 (d, J = 13.4 Hz, 1H), 3.42 (tt, J = 9.0, 4.4 Hz, 1H), 3.18 (dd, J = 13.3, 3.2 Hz, 1H), 2.72 (td, J = 9.8, 6.8 Hz, 1H), 2.56 (ddd, J = 12.9, 10.2, 4.2 Hz, 1H), 2.16 – 2.06 (m, 1H).^{13}C NMR (126 MHz, $CDCl_3$) δ 181.2, 139.8, 139.6, 136.6, 133.0, 128.47, 128.45, 128.0, 127.7, 127.6, 127.5, 127.0, 125.9, 122.1, 109.3, 77.2, 59.8, 58.0, 52.1, 34.1. HRMS (LC/Q-TOF) (m/z) *calcd.* for $C_{24}H_{22}N_2O$ $[M+H]^+$ 355.1805, Found 355.1812.

3.4.3 Ring-Enlargement reaction[11]

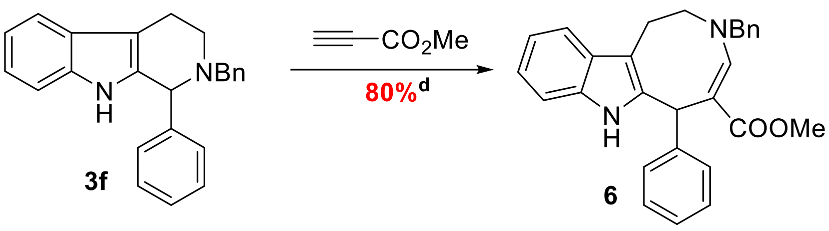

To a solution of compound **3f** (67.69 mg, 0.2 mmo) in anhydrous CH_2Cl_2 (2.0 mL 0.1 M) at room temperature was added methyl propiolate (20.0 µL, 0.22 mmol). The reaction mixture was stirred at R.T. for 7 days. After TLC analysis indicated the completion of the reaction, the reaction mixture was concentrated under reduced pressure. The resulting residue was purified by flash column chromatography (Hexane/EtOAc 15/1 to 5/1) to provide the desired products **6** (35.6 mg, 80 % yield based on recovered starting material, 31.4 mg starting material was recovered) as white solid. ^{1}H NMR (600 MHz, $CDCl_3$) δ 8.44 (s, 1H), 7.91 (s, 1H), 7.36 – 7.33 (m, 2H), 7.26 – 7.21 (m, 5H), 7.20 – 7.12 (m, 4H), 7.12 – 7.08 (m, 3H), 6.04 (s, 1H), 4.41 (d, J = 15.5 Hz, 1H), 4.33 (d, J = 15.5 Hz, 1H), 3.78 (s, 3H), 3.71 – 3.64 (m, 1H), 3.00 – 2.88 (m, 2H), 2.64 – 2.56 (m, 1H). ^{13}C NMR (151 MHz, $CDCl_3$) δ 171.3, 152.5, 146.5, 137.1, 136.7, 134.8, 129.9, 128.8, 128.7, 127.9, 127.3, 126.4, 125.8, 121.5, 119.2, 118.2, 110.8, 107.9, 96.7, 61.3, 51.7, 47.6, 41.6, 25.5. HRMS (LC/Q-TOF) (m/z) *calcd.* for $C_{28}H_{26}N_2O_2$ $[M+H]^+$ 423.2067, Found 423.2069.

3.5 Mechanistic studies

3.5.1 Control experiments

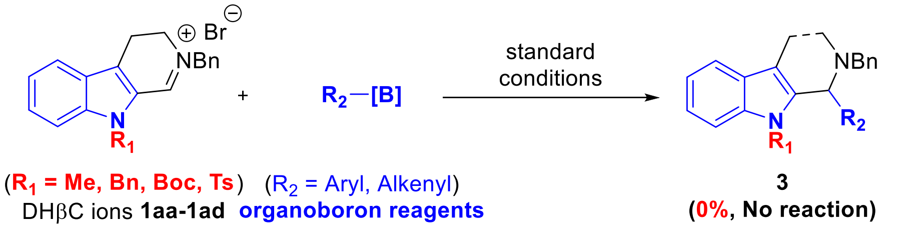

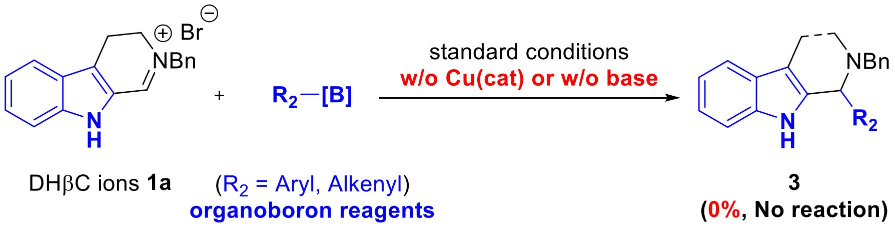

To verify the crucial role of indole nitrogen, *N*-benzyl DHβC ion **1aa – 1ad** with a protection group on indole nitrogen were employing and subjected to the standard reaction conditions. No product **3** was detected by TLC and NMR analysis. To verify the necessary of copper catalysts and base, *N*-benzyl DHβC ion **1a** was used as the substrate to engage the reaction under the standard conditions. While in the absence of copper catalyst or base, no desired products were

observed by TLC.

3.5.2 NMR and HRMS analysis

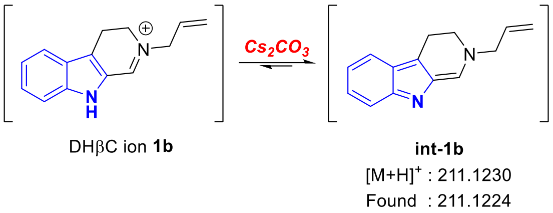

To a flame-dried Schlenk tube were sequentially added DHβC ion **1b** (0.2 mmol), Cs_2CO_3 (130 mg, 0.4 mmol) and THF (4.0 mL, 0.05 M) under a nitrogen atmosphere. The reaction mixture in Schlenk tube was placed in a pre-heated oil bath at 50 °C for 24 h. After cooling to room temperature, the reaction mixture was concentrated under reduced pressure. The resulting residue was dissolved in $CDCl_3$ for NMR analysis directly. Figure S1 shows the results of NMR spectrum.

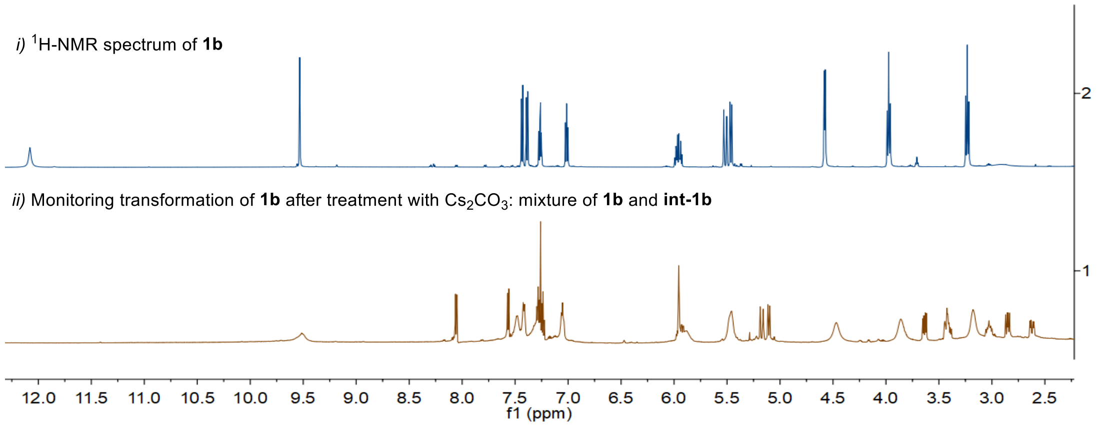

Figure S1. ^{1}H-NMR spectrum of **1b** and a mixture of **1b** and **int-1b**

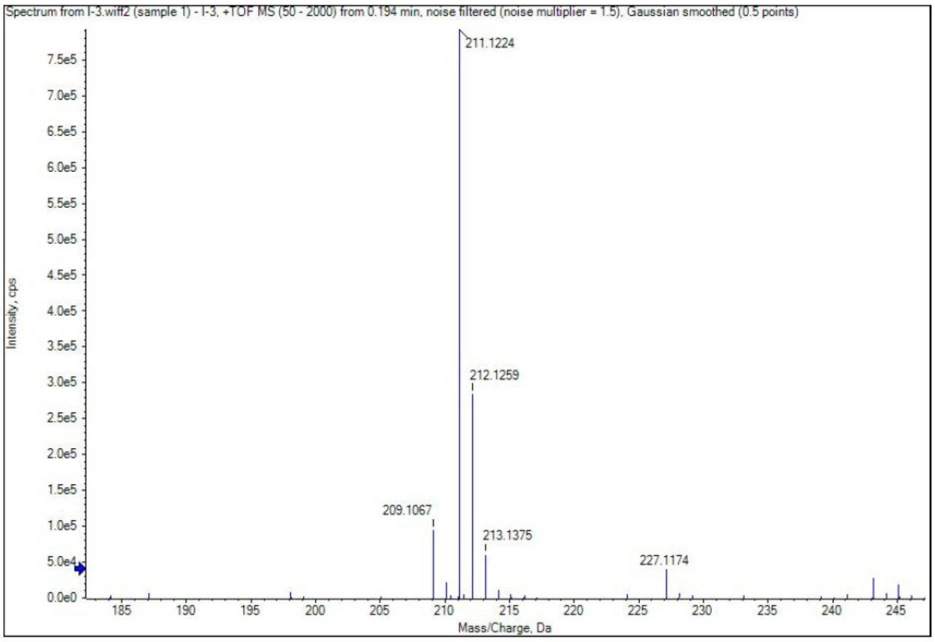

Figure S2. HRMS spectra of **int-1b**

Intermediate **int-1b** can be detected by HRMS (Figure S2), confirming the transformation of **1b** to **int-1b** upon alkaline condition.

3.5.3 UV-Vis spectroscopy analysis

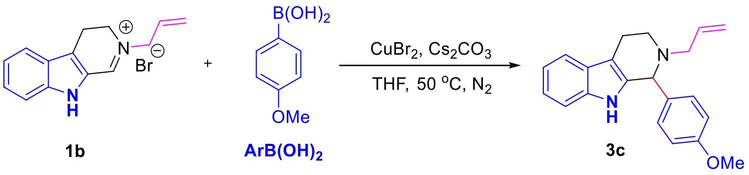

Figure S3 shows the results of measuring the UV-vis. Absorbance of *N*-alkyl D HβC ion **1b** (0.01M, in THF); a mixture of **1b** and Cs_2CO_3 (1:1, 0.01M of **1b**, in THF, treated with 50 ºC oil bath heating for 12 h under nitrogen atmosphere); arylboronic acid $ArB(OH)_2$ (0.01M, in THF); a mixture of $ArB(OH)_2$, $CuBr_2$ and Cs_2CO_3 (1:1:1, 0.01M of $ArB(OH)_2$, in THF, treated with 50 ºC oil bath heating for 12 h under nitrogen atmosphere); a mixture of **1b** and $ArB(OH)_2$ (1:1, 0.01M of **1b**, in THF, treated with 50 ºC oil bath heating for 12 h under nitrogen atmosphere); a mixture of **1b**, $ArB(OH)_2$ and Cs_2CO_3 (1:1:1, 0.01M of **1b**, in THF, treated with 50 ºC oil bath heating for 12 h under nitrogen atmosphere); a mixture of **1b**, $ArB(OH)_2$, $CuBr_2$ and Cs_2CO_3 (1:1:1:1, 0.01M of **1b**, in THF, treated with 50 ºC oil bath heating for 12 h under nitrogen atmosphere). The UV-Vis absorption spectrum were measured on Shimadzu UV-2600i UV-Vis spectrophotometer.

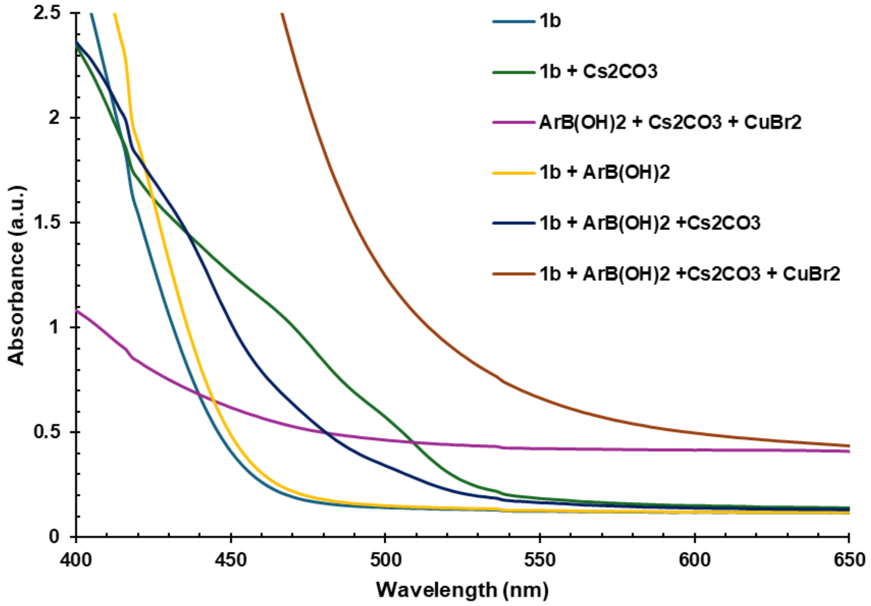

Figure S3. UV-Vis spectroscopy analysis

3.5.4 Radical inhibition experiments

i) Radical inhibition experiment with TEMPO

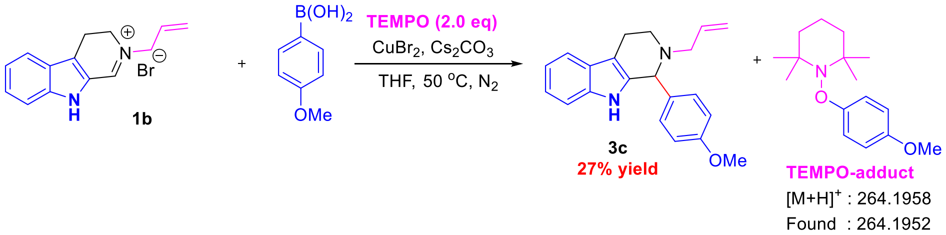

To a flame-dried Schlenk tube were sequentially added DHβC ion **1b** (0.2 mmol), $CuBr_2$ (9.0 mg, 20% mmol), Cs_2CO_3 (130 mg, 0.4 mmol), arylboronic acid (0.4 mmol), TEMPO (2,2,6,6-tetramethyl-1-piperidinyloxy, 62.5 mg, 0.4 mmol), 5 Å molecular sieves (100 mg) and THF (4.0 mL, 0.05 M) under a nitrogen atmosphere. The reaction mixture in Schlenk tube was placed in a pre-heated oil bath at 50 °C for 24 h. After cooling to room temperature, the crude reaction was filtrated through a short pad of silica gel washed with EtOAc (25 mL). After solvent evaporation, the residue was purified by chromatography on silica gel (10:1 hexane: EtOAc) to afford C1-aryl THβC **3c** (17.1 mg, 27% yield).

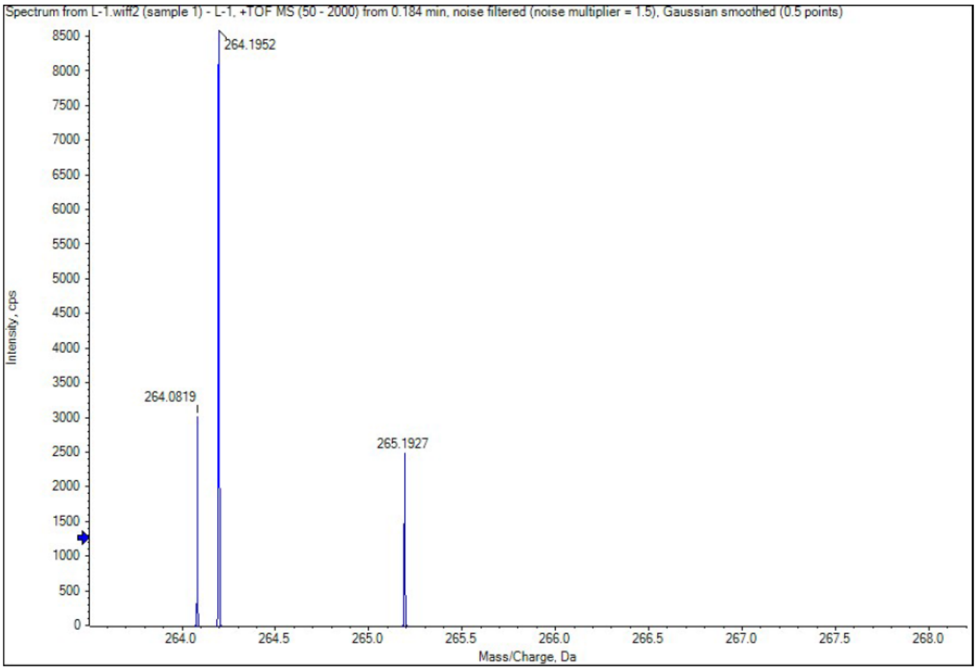

Figure S4. HRMS spectra of TEMPO-adduct

TEMPO-adduct can be detected by HRMS, indicating that the reaction might involve a singlet electron transfer (SET) process.

ii) Radical inhibition experiment with BHT

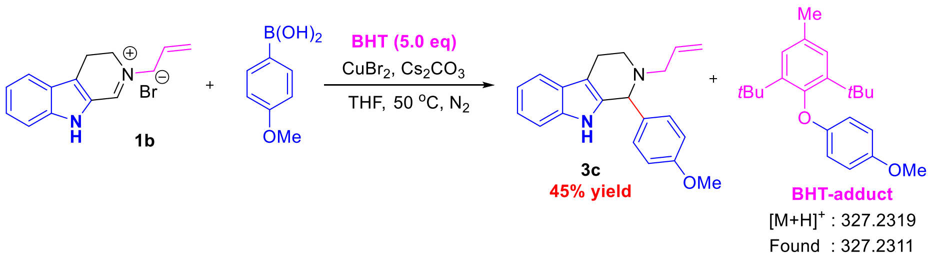

To a flame-dried Schlenk tube were sequentially added DHβC ion **1b** (0.2 mmol), $CuBr_2$ (9.0 mg, 20% mmol), Cs_2CO_3 (130 mg, 0.4 mmol), arylboronic acid (0.4 mmol), BHT (butylated hydroxytoluene, 220.4 mg, 1.0 mmol), 5 Å molecular sieves (100 mg) and THF (4.0 mL, 0.05 M) under a nitrogen atmosphere. The reaction mixture in Schlenk tube was placed in a pre-heated oil bath at 50 ºC for 24 h. After cooling to room temperature, the crude reaction was filtrated through a short pad of silica gel washed with EtOAc (25 mL). After solvent evaporation, the residue was purified by chromatography on silica gel (10:1 hexane: EtOAc) to afford C1-aryl THβC **3c** (28.7 mg, 45% yield).

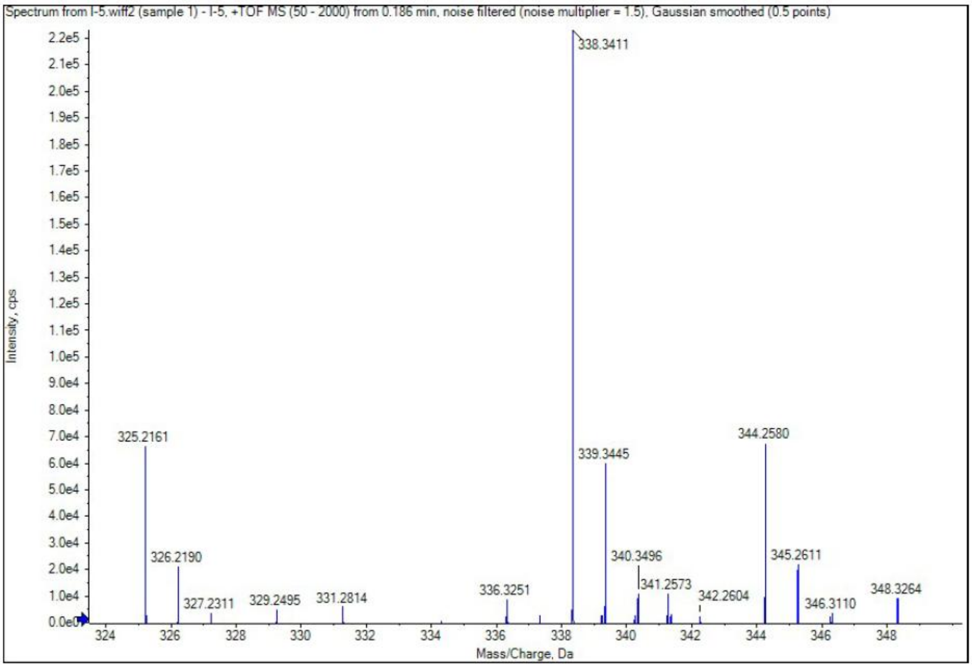

Figure S5. HRMS spectra of BHT-adduct

BHT-adduct can be detected by HRMS, indicating that the reaction might involve a singlet electron transfer (SET) process.

4. References

(1) Bertrand, M.; Poissonnet, G.; Théret-Bettiol, M.-H.; Gaspard, C.; Werner, G. H.; Pfeiffer, B.; Renard, P.; Léonce, S.; Dodd, R. H. Cytotoxic activities of novel hexahydroindolizino[8,7-b]indole derivatives prepared by 1,3-dipolar cycloaddition reactions of 3,4-dihydro-β-carboline ylides. *Biorg. Med. Chem.* **2001**, *9* (8), 2155–2164. DOI: https://doi.org/10.1016/S0968-0896(01)00119-5.

(2) Andriamialisoa, R. Z.; Langlois, N.; Langlois, Y. A new efficient total synthesis of vindorosine and vindoline. *J. Org. Chem.* **1985**, *50* (7), 961–967. DOI: 10.1021/jo00207a011.

(3) González-Gómez, Á.; Domínguez, G.; Pérez Castells, J. Enyne and dienyne metathesis reactions in β-carbolines. *Tetrahedron Lett.* **2005**, *46* (42), 7267–7270. DOI: https://doi.org/10.1016/j.tetlet.2005.07.140.

(4) Yuan, Y.-H.; Han, X.; Zhu, F.-P.; Tian, J.-M.; Zhang, F.-M.; Zhang, X.-M.; Tu, Y.-Q.; Wang, S.-H.; Guo, X. Development of bifunctional organocatalysts and application to asymmetric total synthesis of naucleofficine I and II. *Nature Communications* **2019**, *10* (1), 3394. DOI: 10.1038/s41467-019-11382-8.

(5) Dong, G.; Wang, S.; Miao, Z.; Yao, J.; Zhang, Y.; Guo, Z.; Zhang, W.; Sheng, C. New tricks for an old natural product: discovery of highly potent evodiamine derivatives as novel antitumor agents by systemic structure–activity relationship analysis and biological evaluations. *J. Med. Chem.* **2012**, *55* (17), 7593–7613. DOI: 10.1021/jm300605m.

(6) Ghislieri, D.; Green, A. P.; Pontini, M.; Willies, S. C.; Rowles, I.; Frank, A.; Grogan, G.; Turner, N. J. Engineering an enantioselective amine oxidase for the synthesis of pharmaceutical building blocks and alkaloid natural products. *J. Am. Chem. Soc.* **2013**, *135* (29), 10863–10869. DOI: 10.1021/ja4051235.

(7) Liang, L.; Zhou, S.; Zhang, W.; Tong, R. Catalytic asymmetric alkynylation of 3,4-dihydro-β-carbolinium ions enables collective total syntheses of indole alkaloids. *Angew. Chem. Int. Ed.* **2021**, *60* (47), 25135–25142. DOI: https://doi.org/10.1002/anie.202112383 (acccessed 2025/07/21).

(8) Chauhan, J.; Luthra, T.; Sen, S. Iodine-catalyzed metal-free oxidative ring opening of 1-aryltetrahydro-β-carbolines: facile synthesis of C-2 aroyl and aryl methanimino indole derivatives. *European J. Org. Chem.* **2018**, *2018* (34), 4776–4786. DOI: https://doi.org/10.1002/ejoc.201800879 (acccessed 2025/07/23).

(9) Elderwish, S.; Audebrand, A.; Nebigil, C. G.; Désaubry, L. Discovery of 3,3′-pyrrolidinyl-spirooxindoles as cardioprotectant prohibitin ligands. *Eur. J. Med. Chem.* **2020**, *186*, 111859. DOI: https://doi.org/10.1016/j.ejmech.2019.111859.

(10) Zheng, C.; Xia, Z.-L.; You, S.-L. Unified mechanistic understandings of pictet-spengler reactions. *Chem* **2018**, *4* (8), 1952–1966. DOI: https://doi.org/10.1016/j.chempr.2018.06.006.

(11) Voskressensky, L. G.; Borisova, T. N.; Chervyakova, T. M.; Titov, A. A.; Kozlov, A. V.; Sorokina, E. A.; Samavati, R.; Varlamov, A. V. Synthesis of 6-aryl-Substituted Azocino-[5,4-b]indoles from 1-aryl-Substituted 2-Ethyltetrahydro-β-Carbolines. *Chem. Heterocycl. Compd.* **2014**, *50* (5), 658-669. DOI: 10.1007/s10593-014-1518-z.

5. Copies of NMR Spectrums

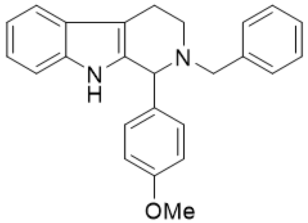

3a

(600 MHz, $CDCl_3$)

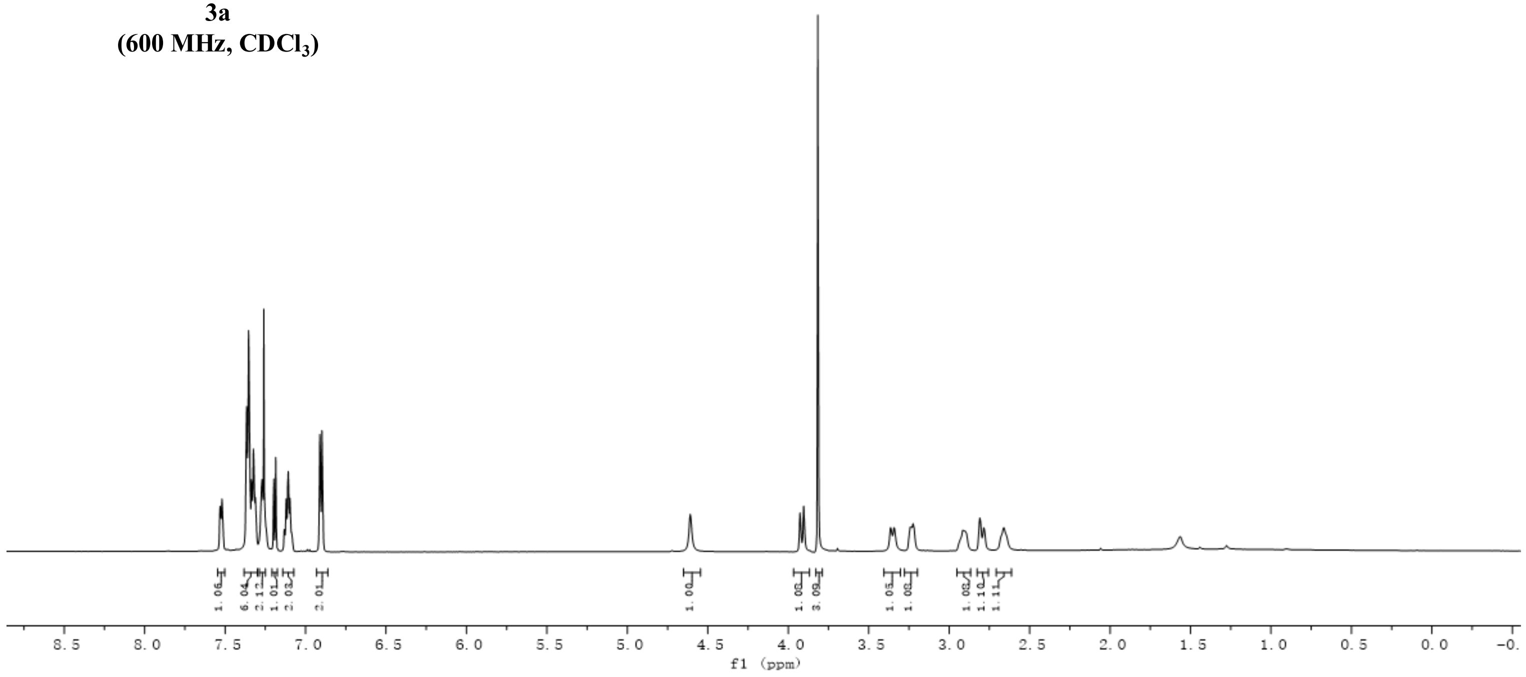

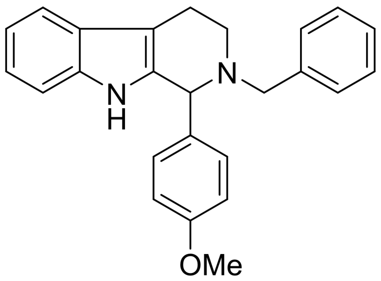

3a

(600 MHz, $CDCl_3$)

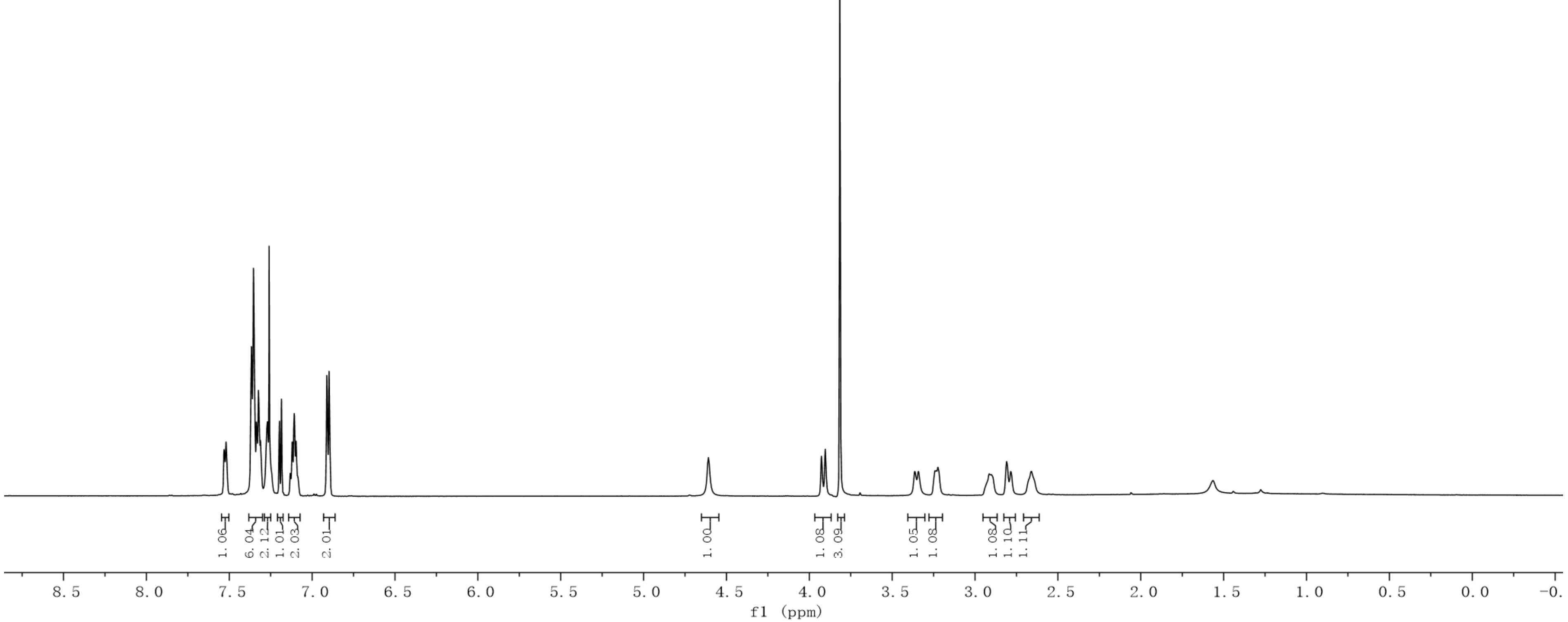

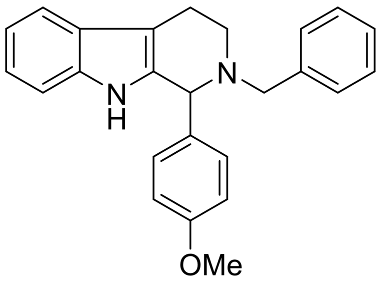

3a

(151 MHz, $CDCl_3$)

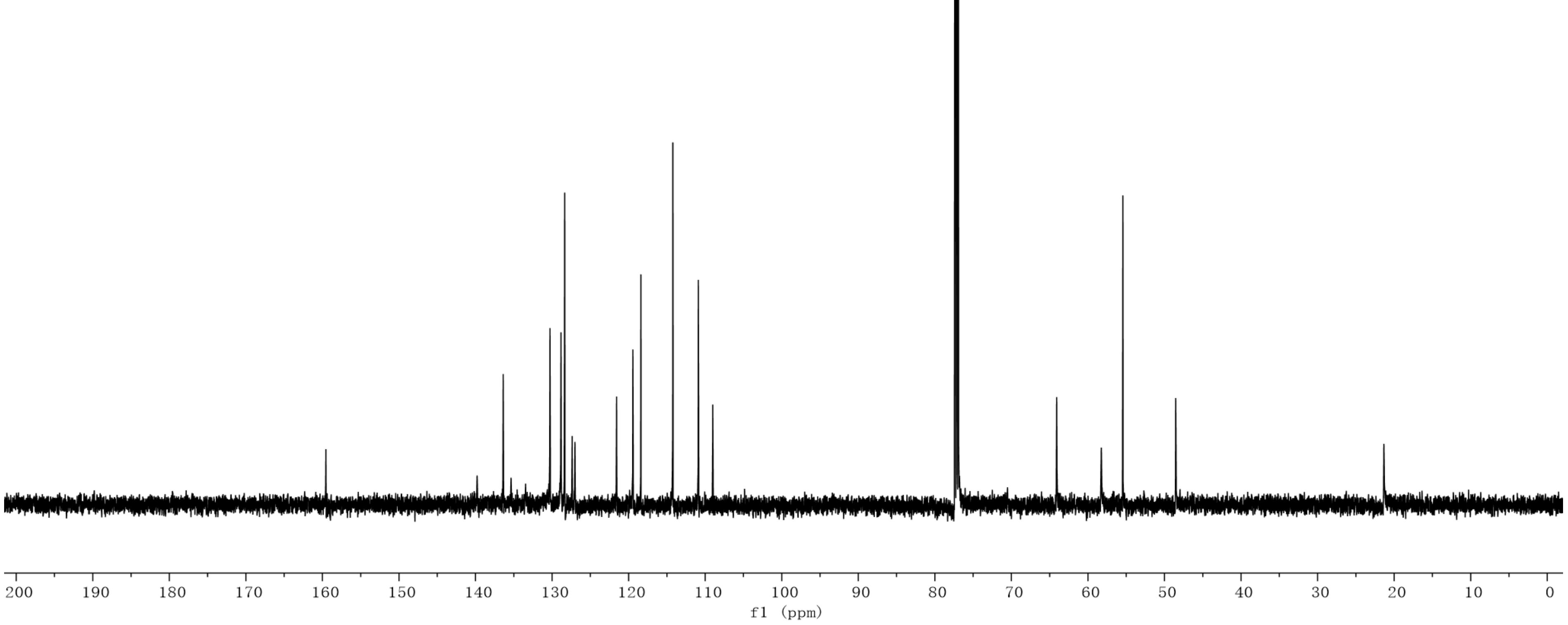

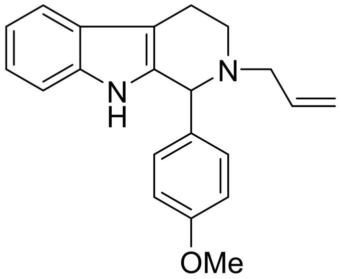

3c

(600 MHz, $CDCl_3$)

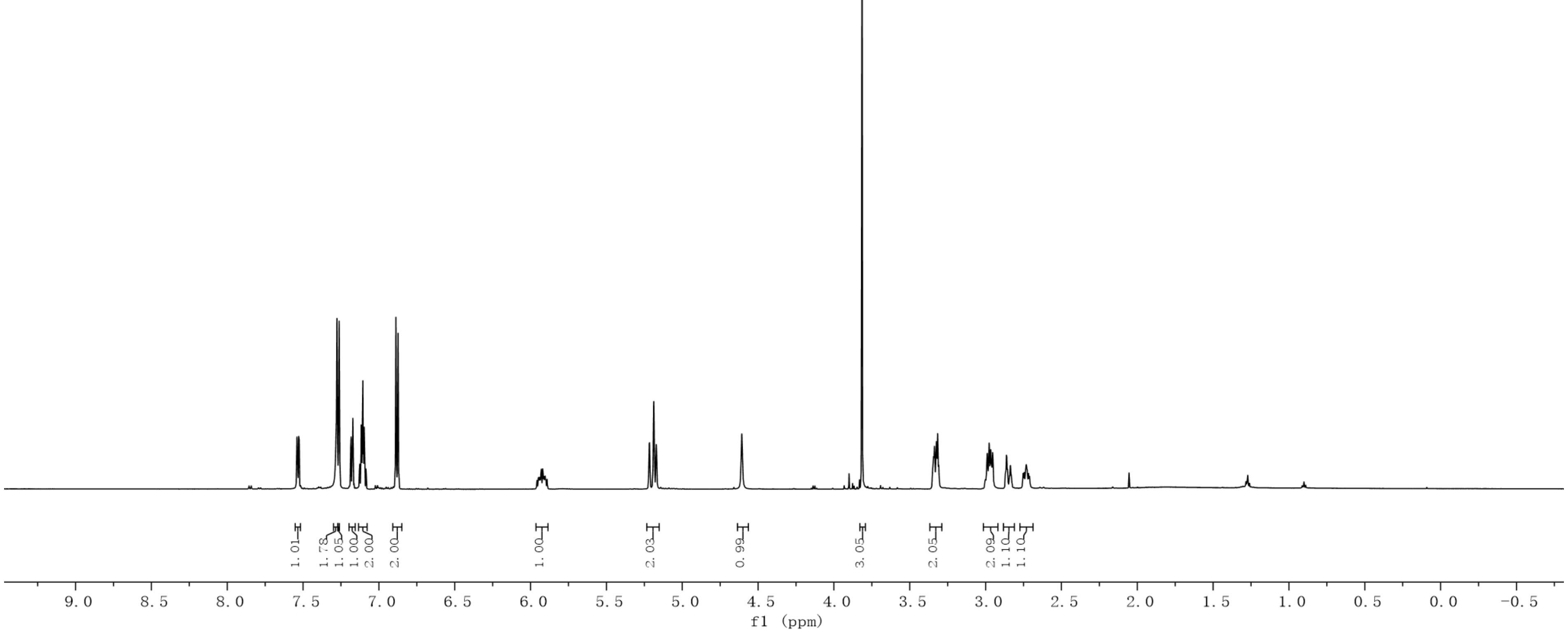

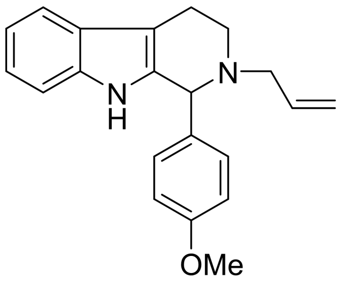

3c
(151 MHz, $CDCl_3$)

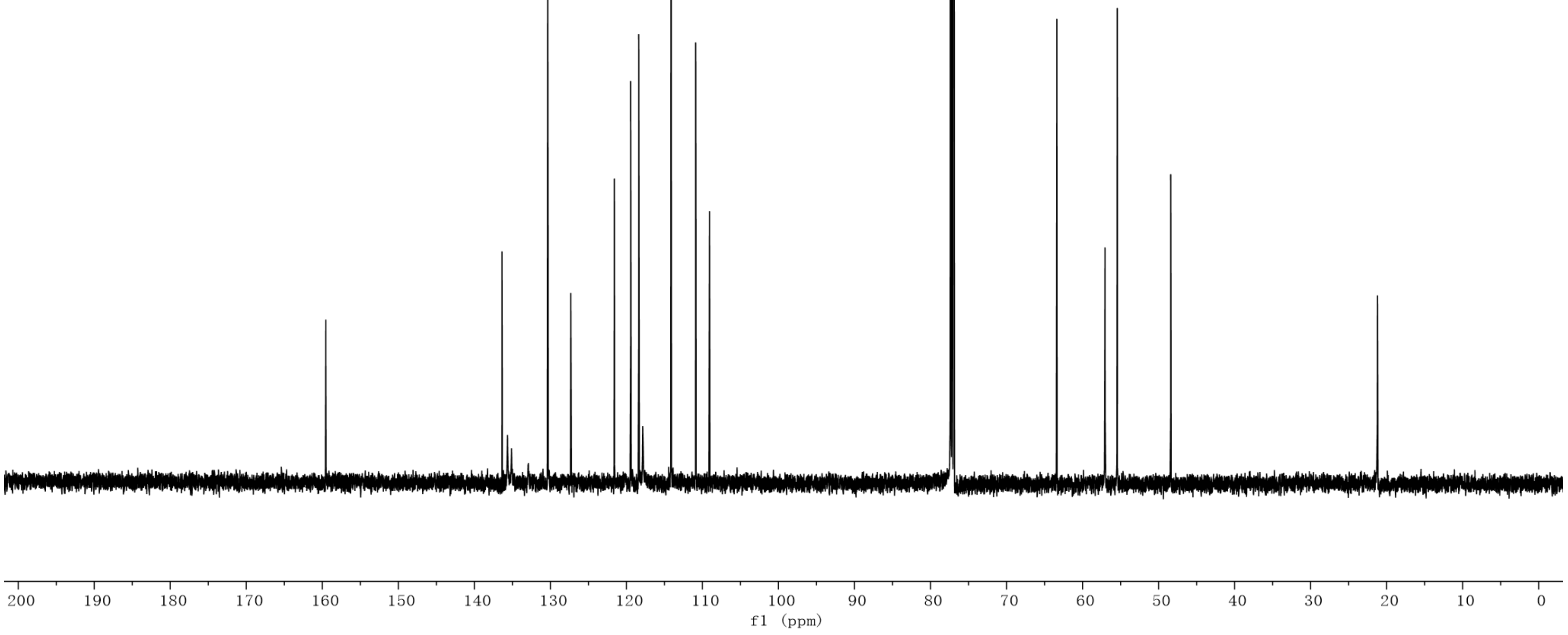

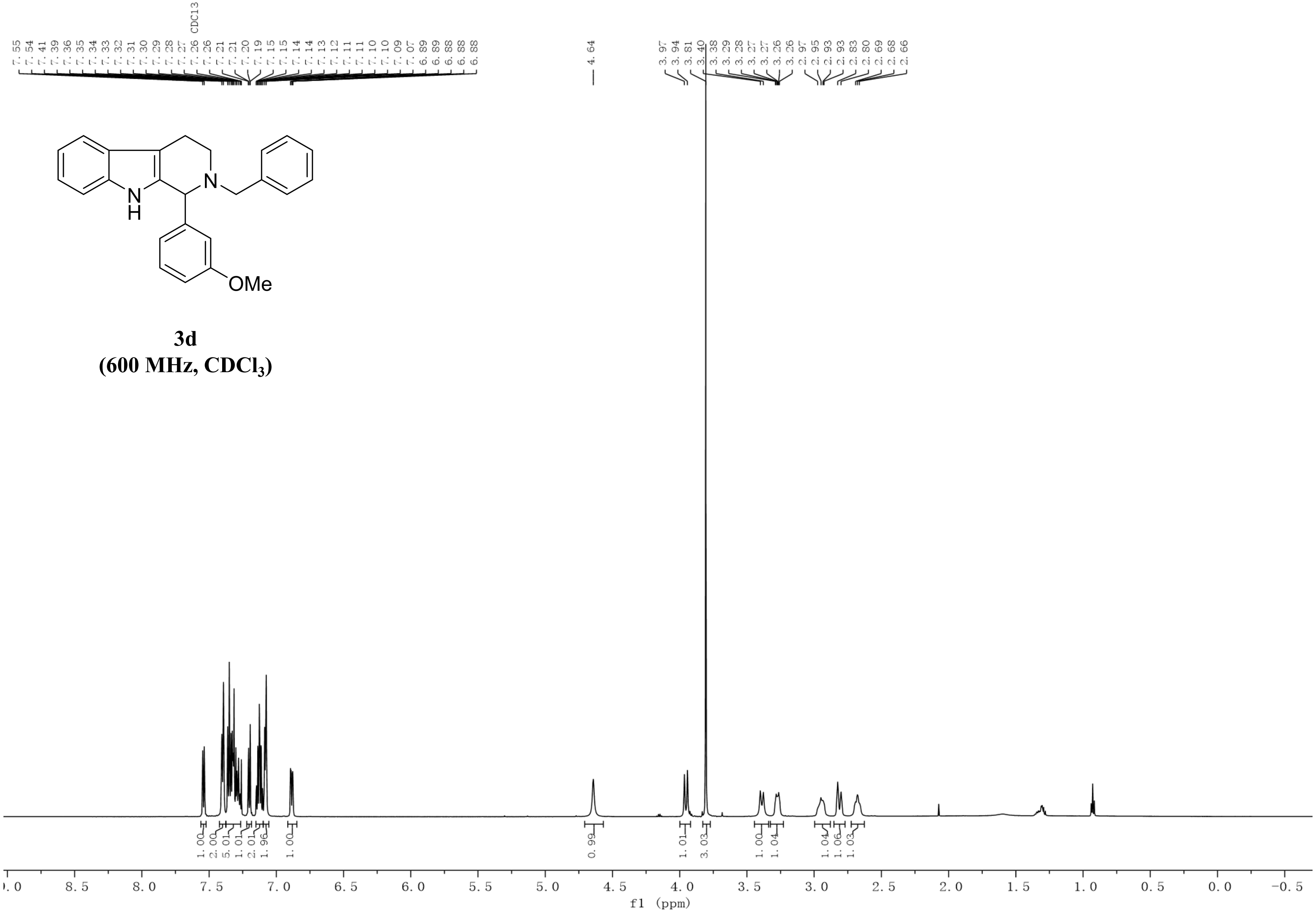

3d
(600 MHz, $CDCl_3$)

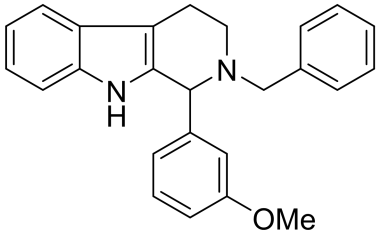

3d
(151 MHz, $CDCl_3$)

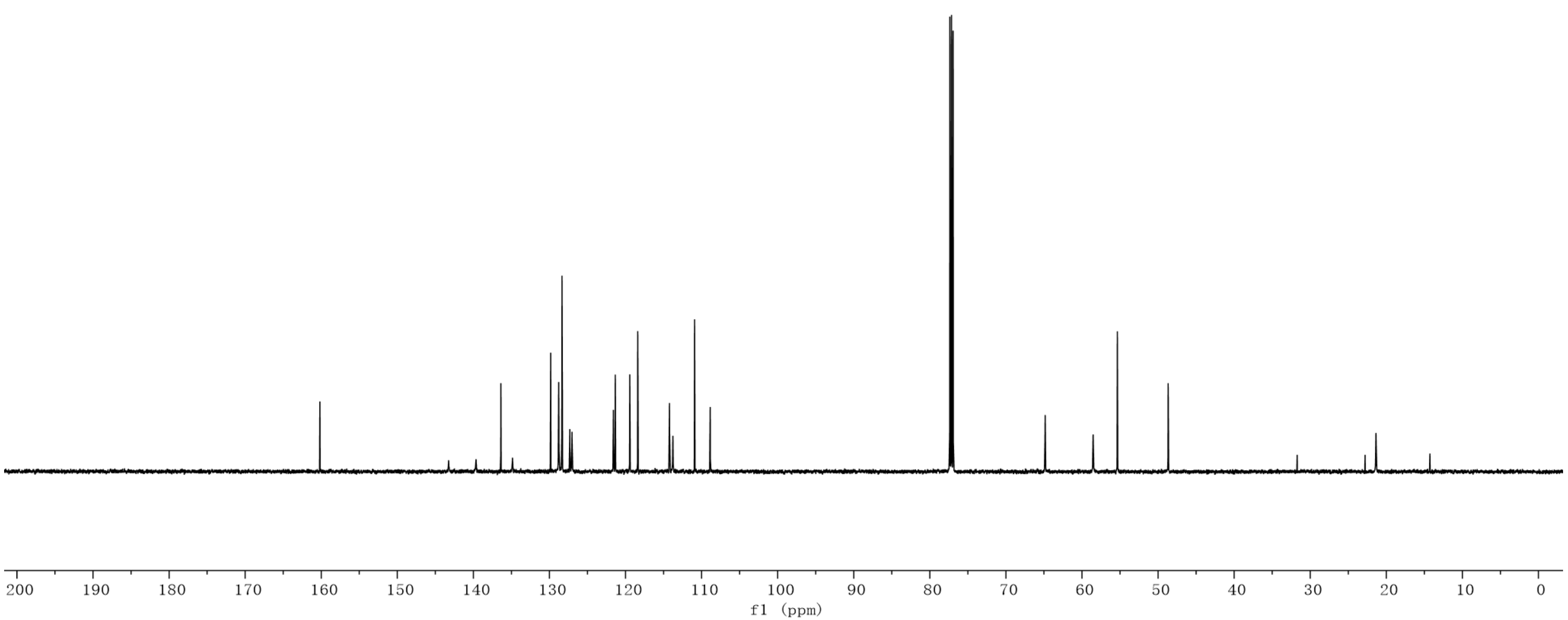

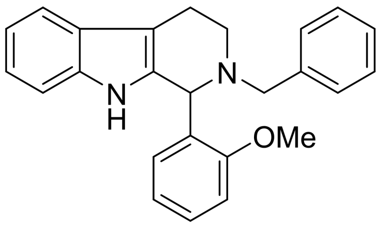

3e
(600 MHz, $CDCl_3$)

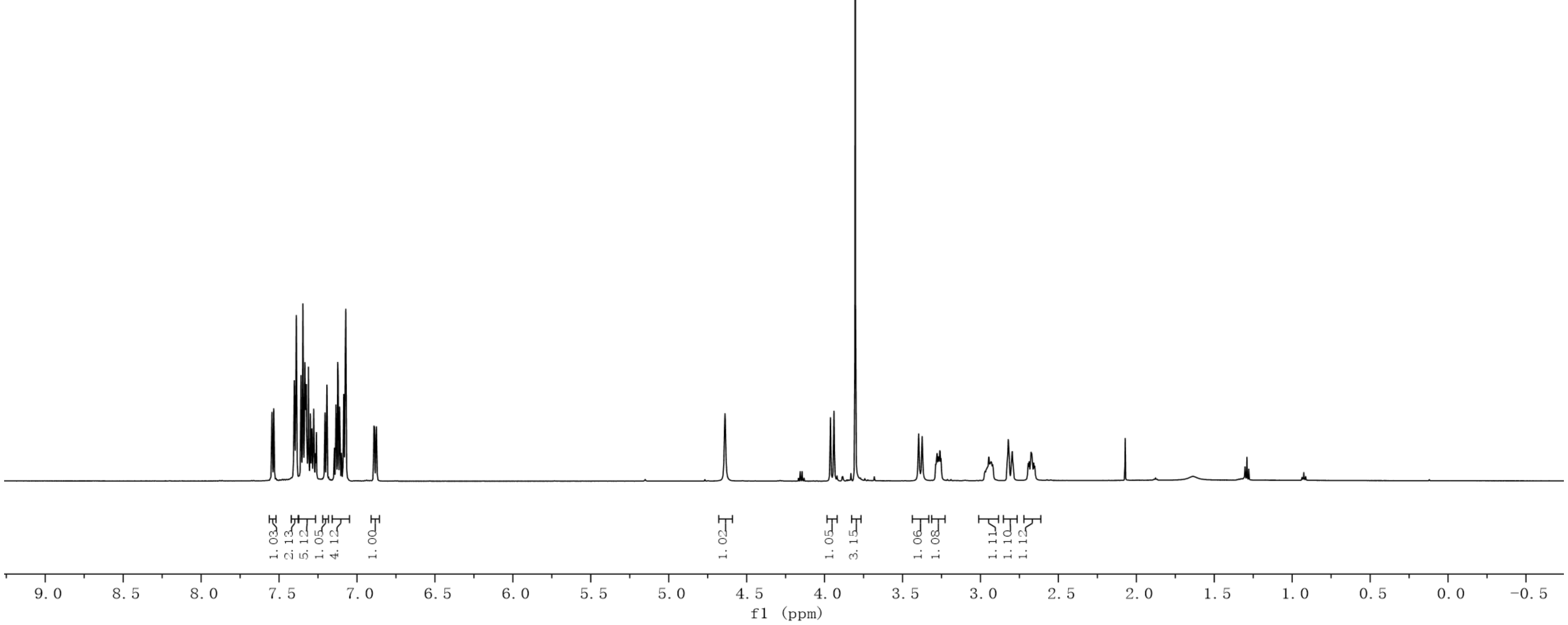

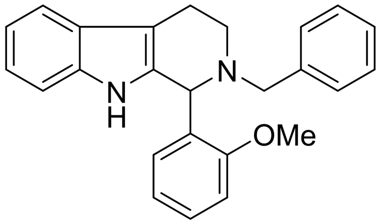

3e
(151 MHz, $CDCl_3$)

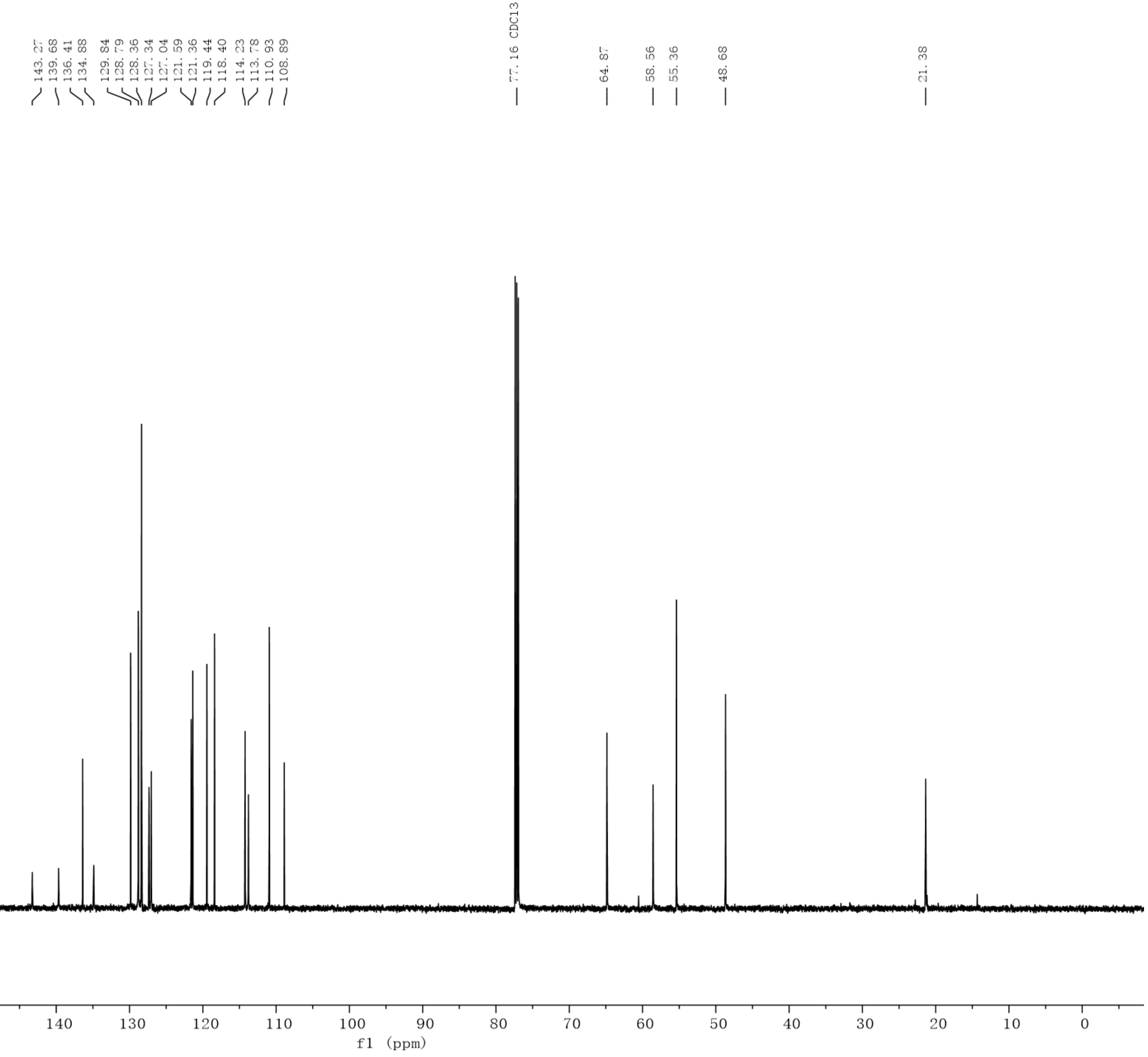

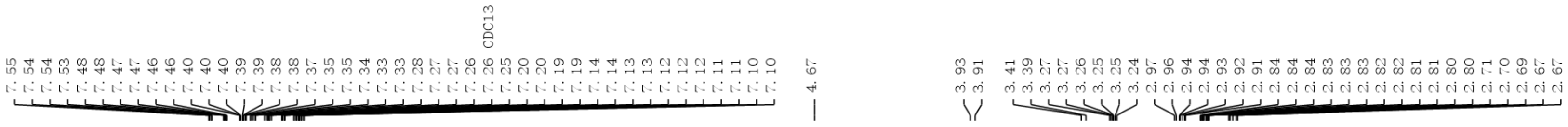

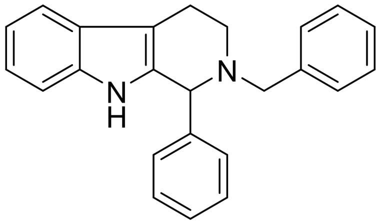

3f
(600 MHz, $CDCl_3$)

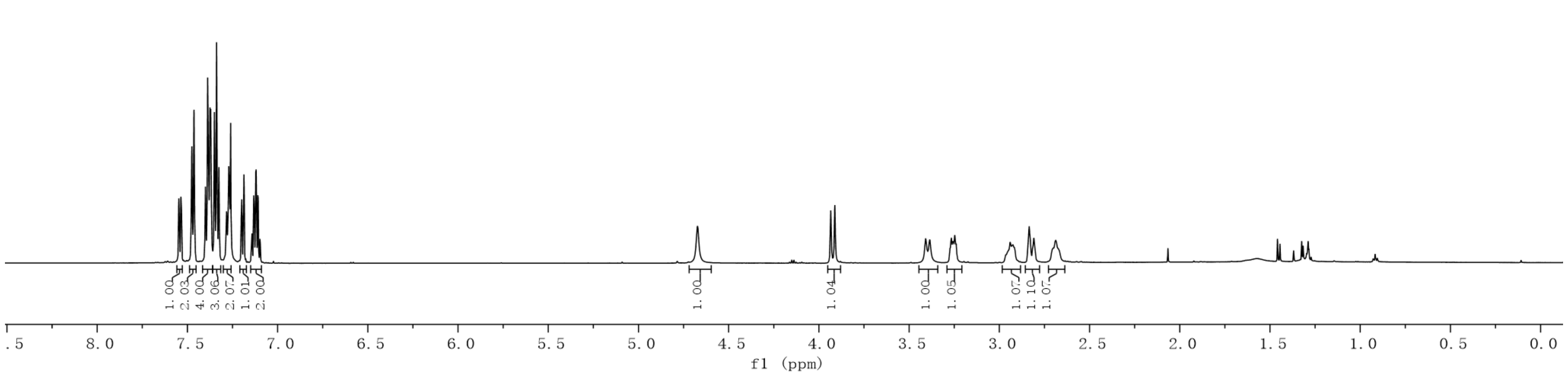

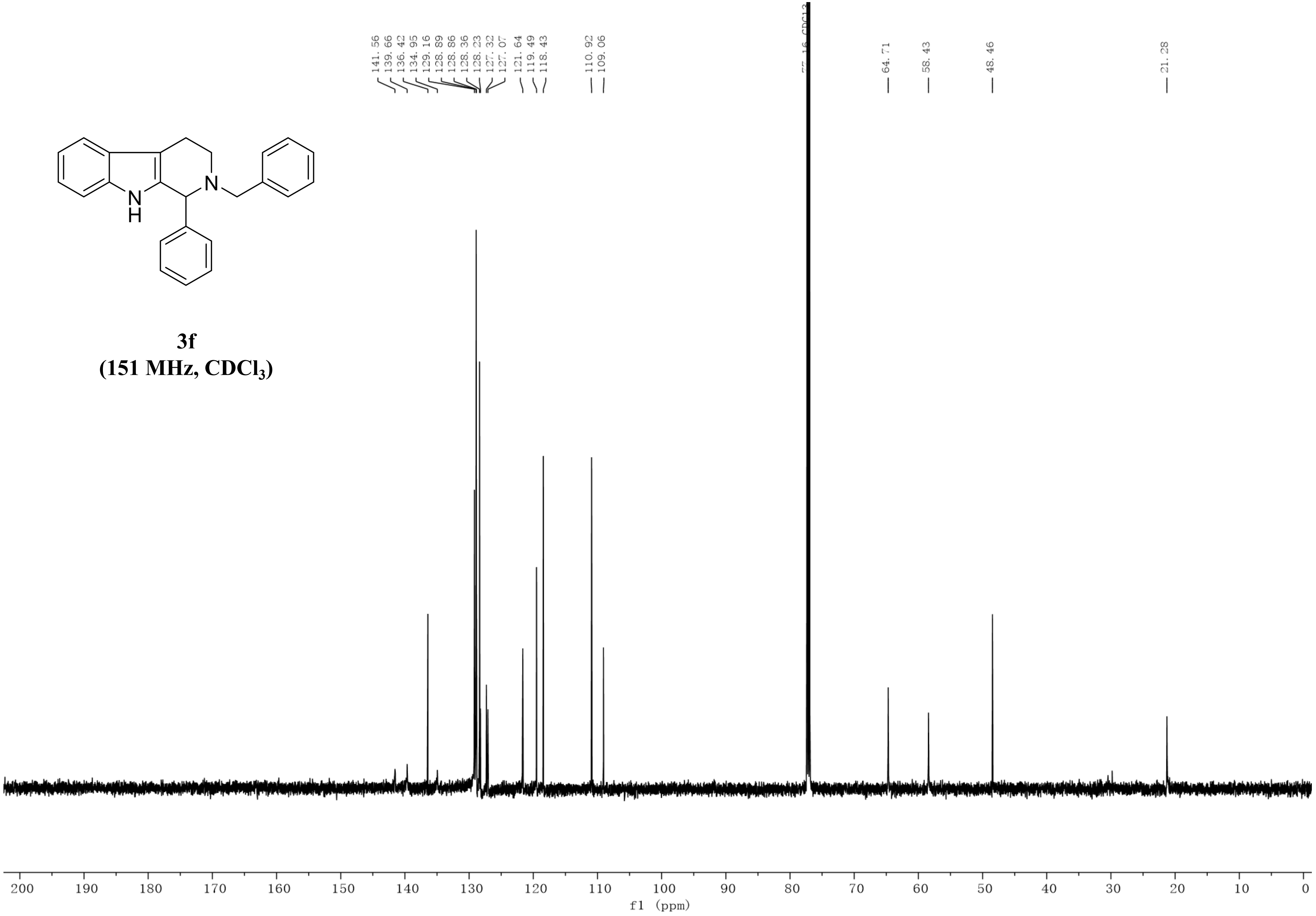

3f
(151 MHz, $CDCl_3$)

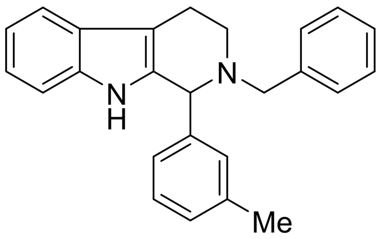

3g
(600 MHz, $CDCl_3$)

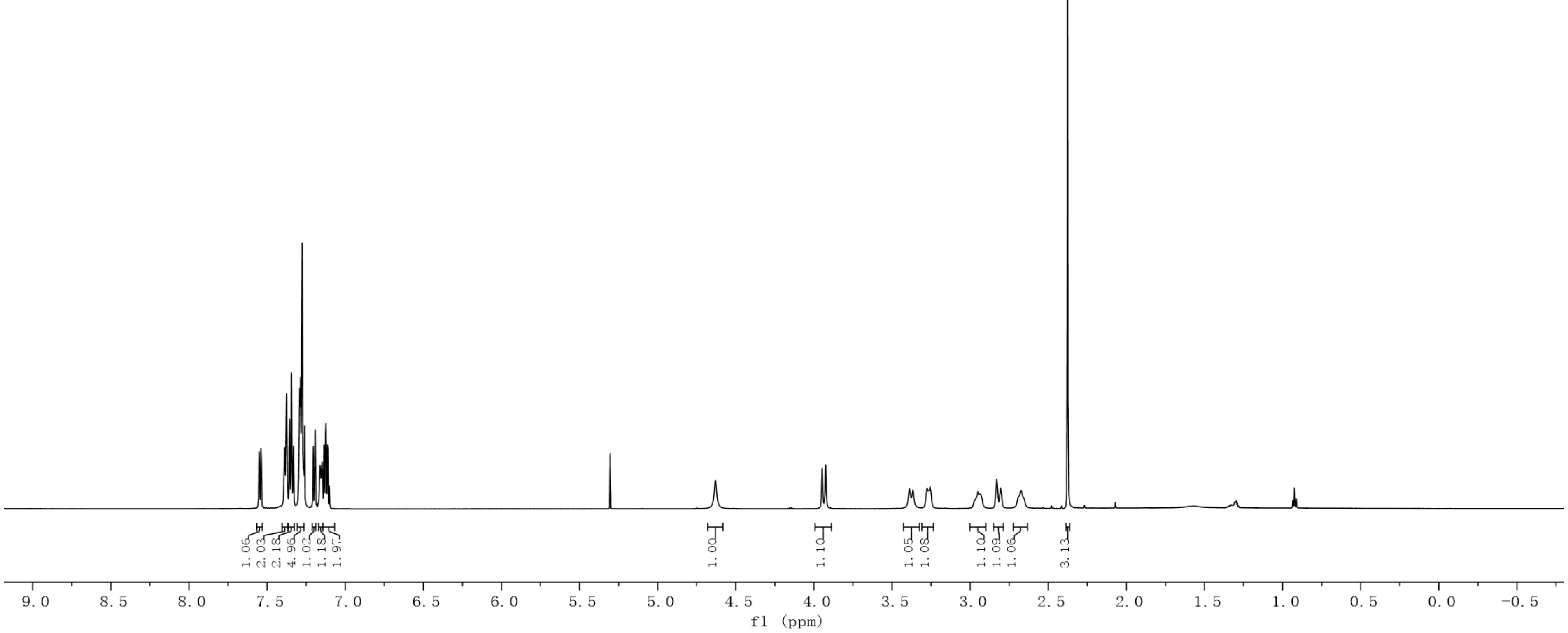

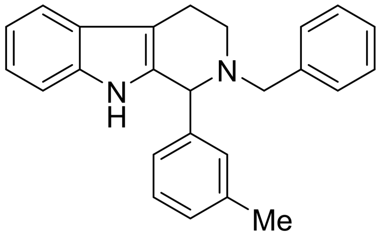

3g
(151 MHz, $CDCl_3$)

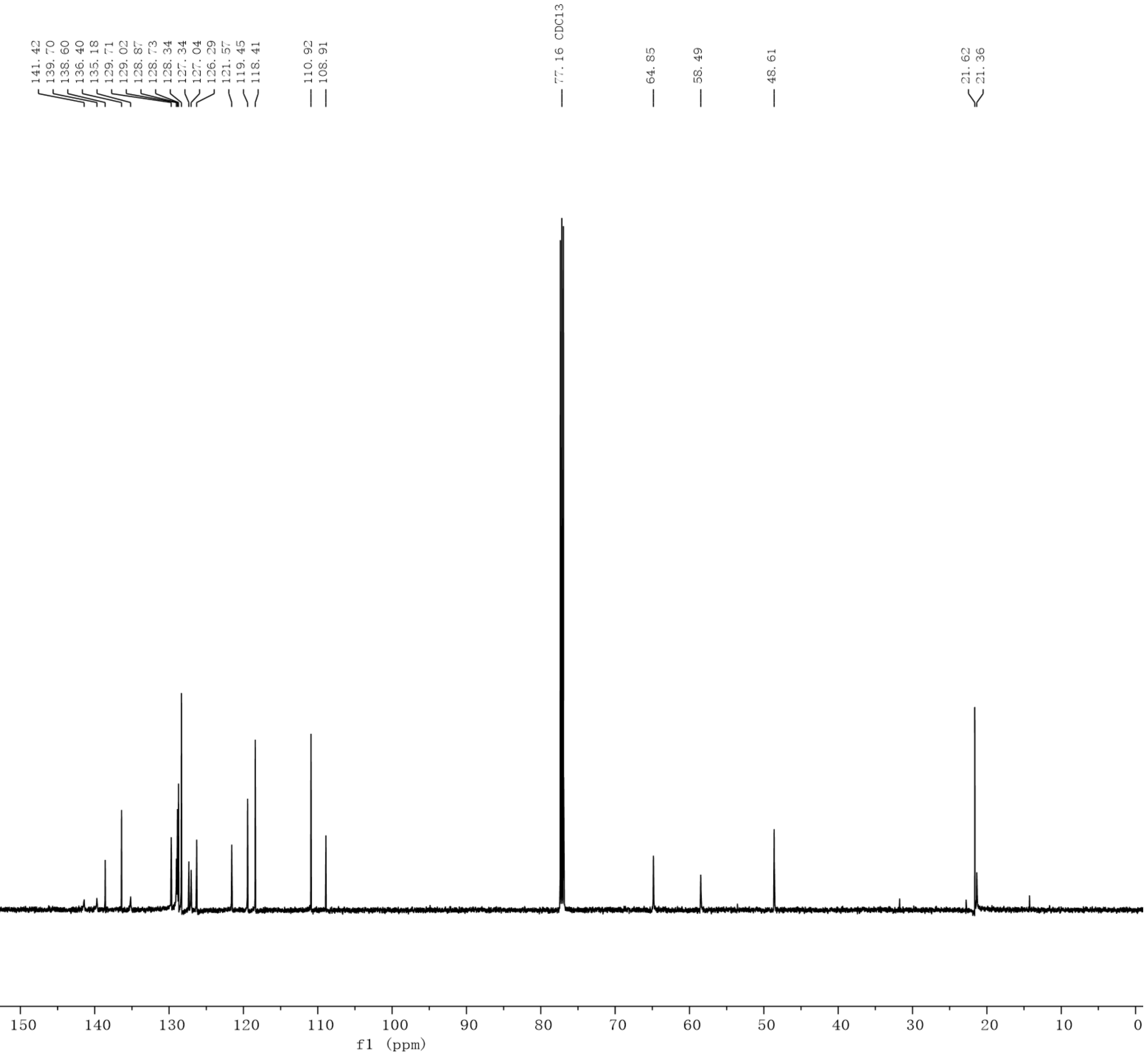

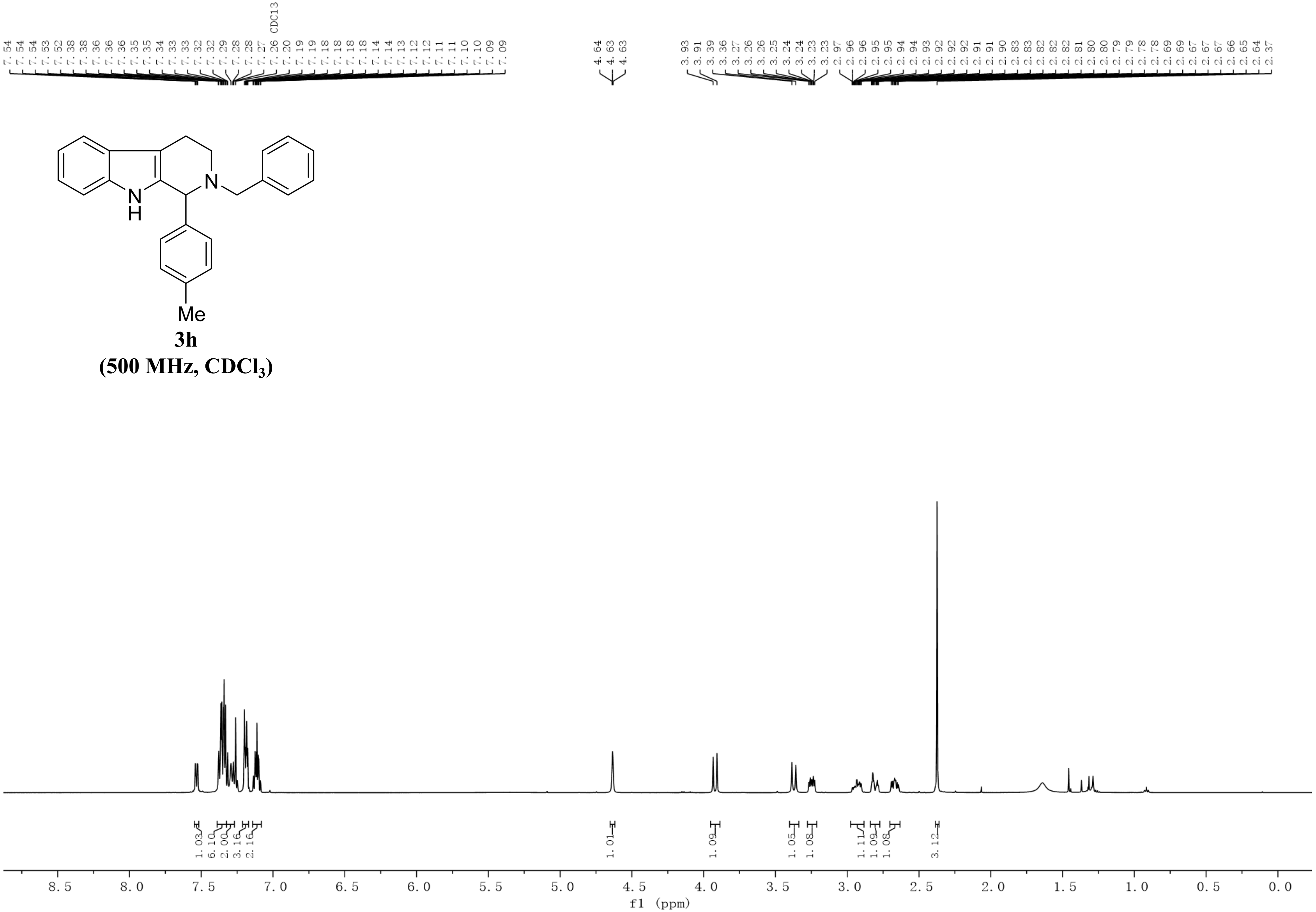

3h
(500 MHz, CDCl3)
f1 (ppm)

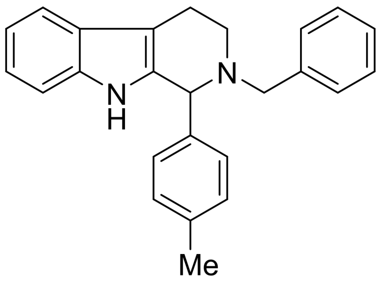

3h
(126 MHz, $CDCl_3$)

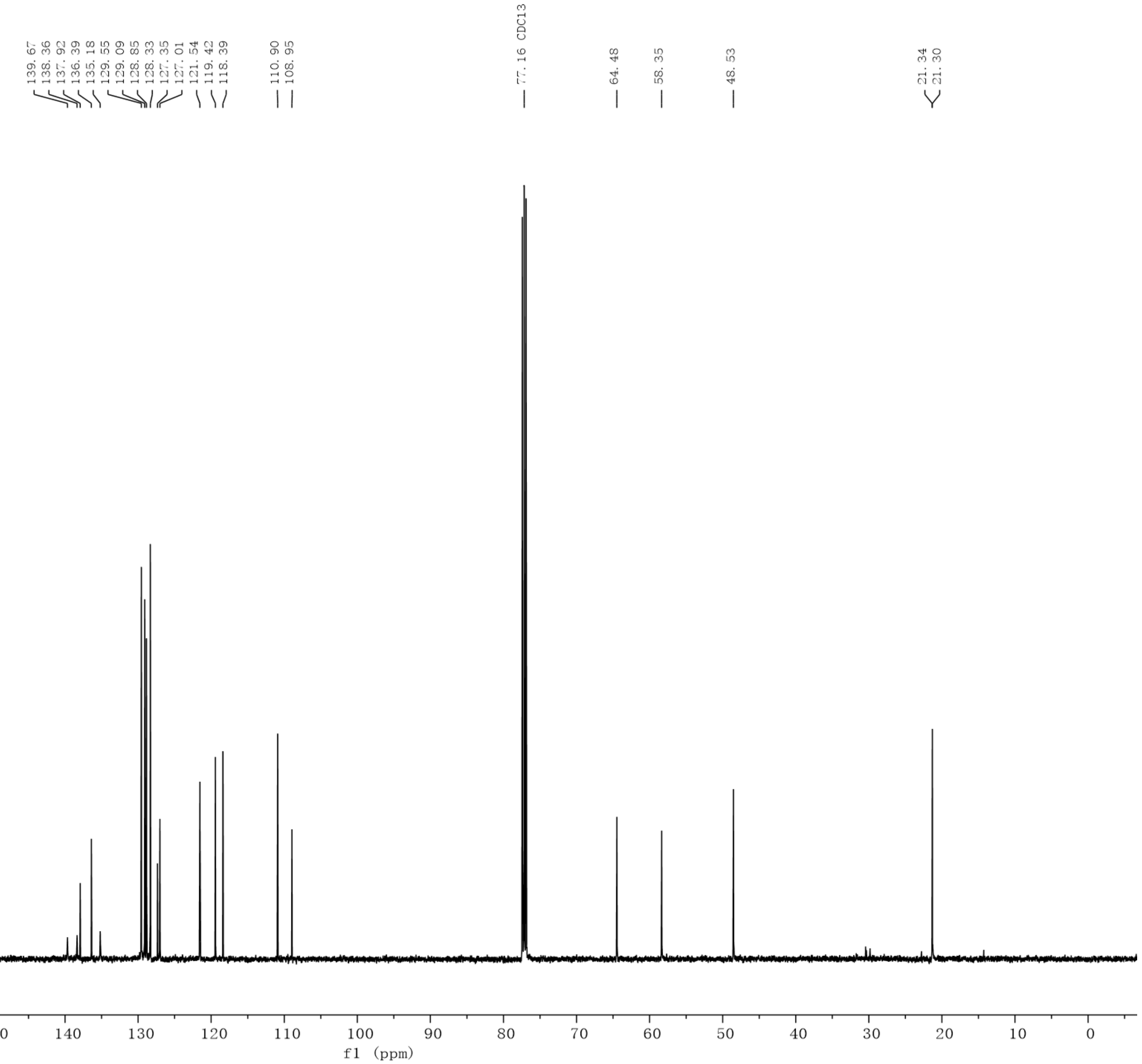

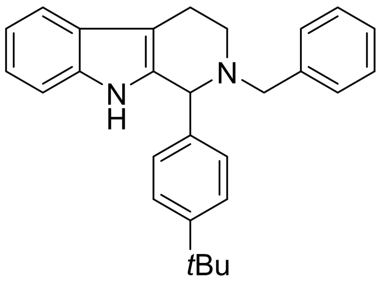

3i
(500 MHz, $CDCl_3$)

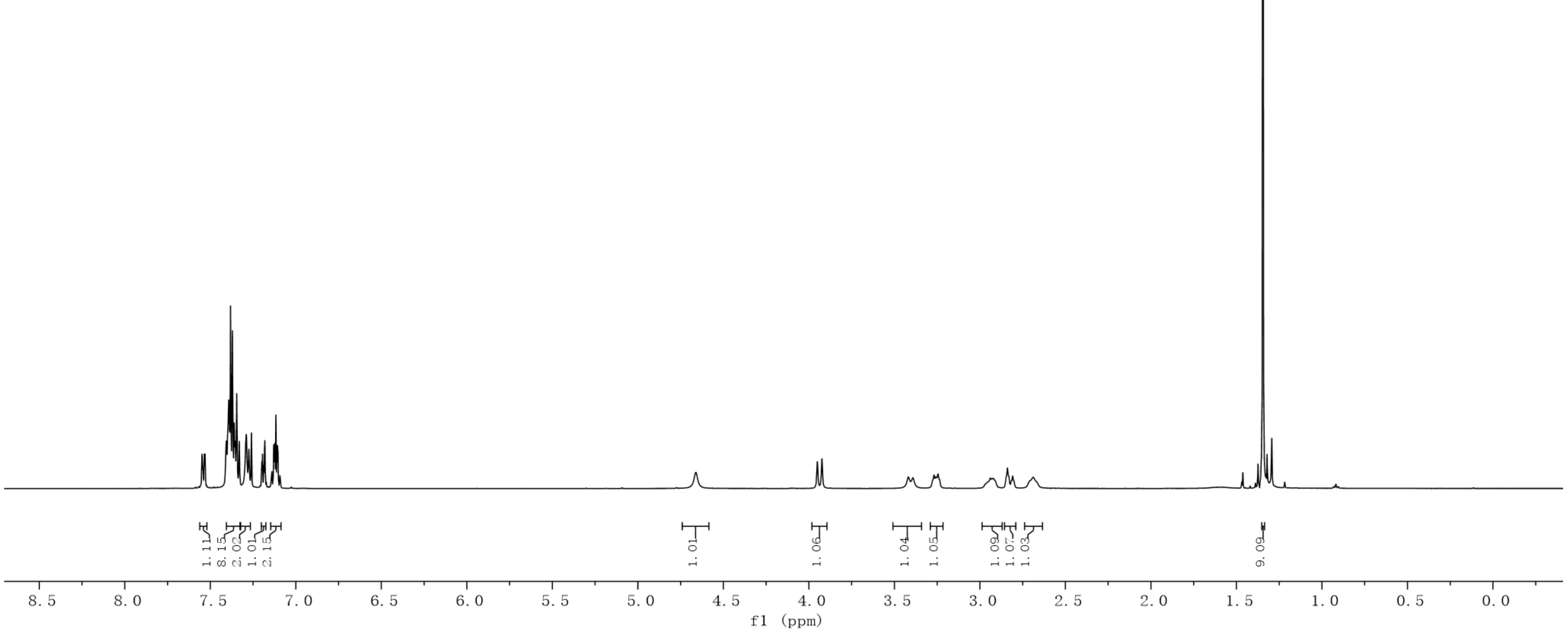

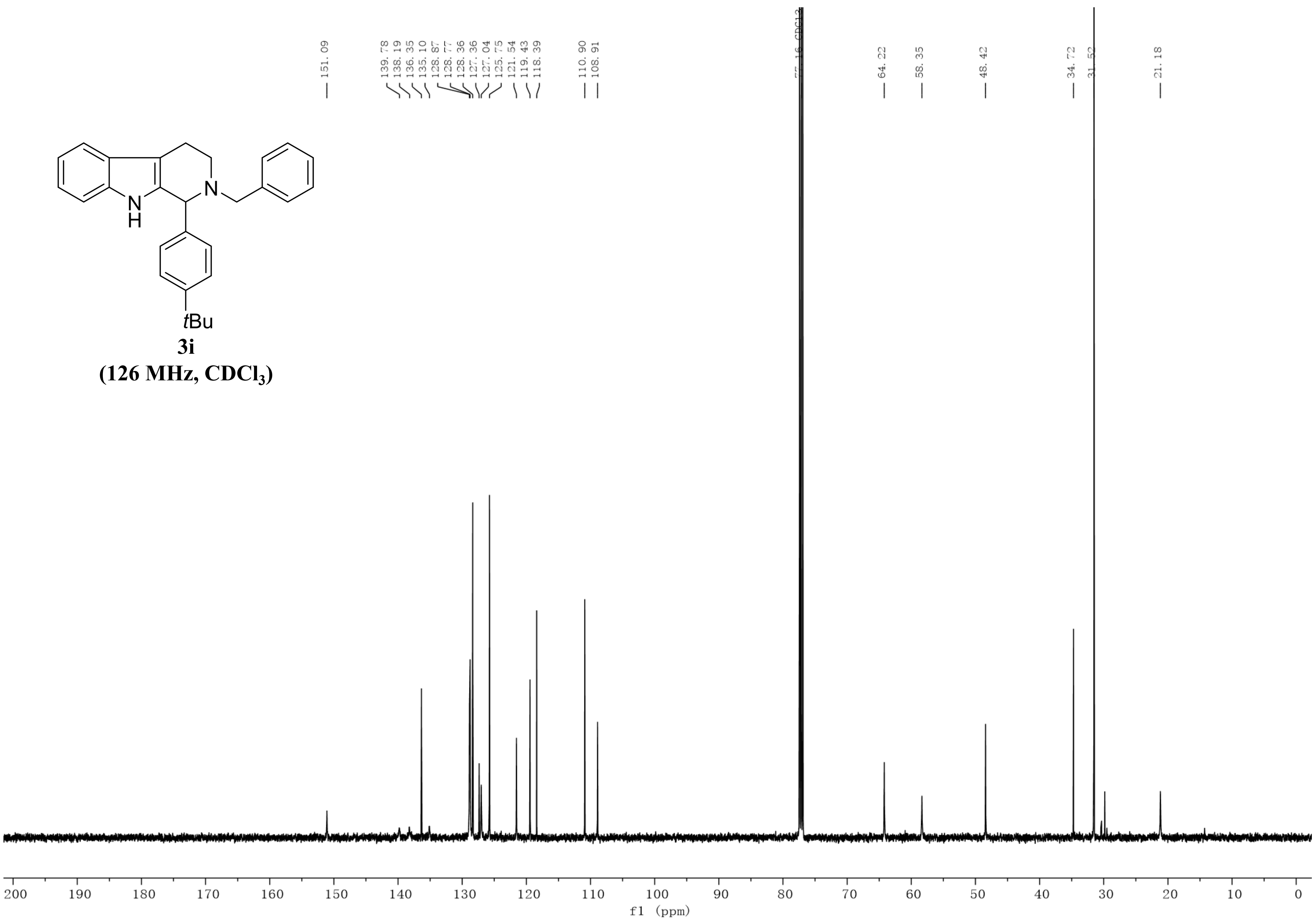

3i

(126 MHz, $CDCl_3$)

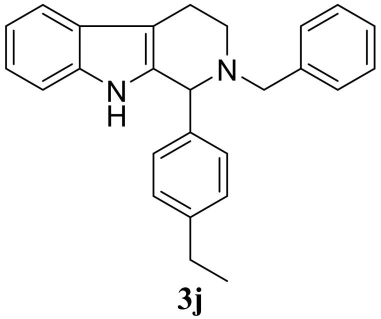

3j
(600 MHz, $CDCl_3$)

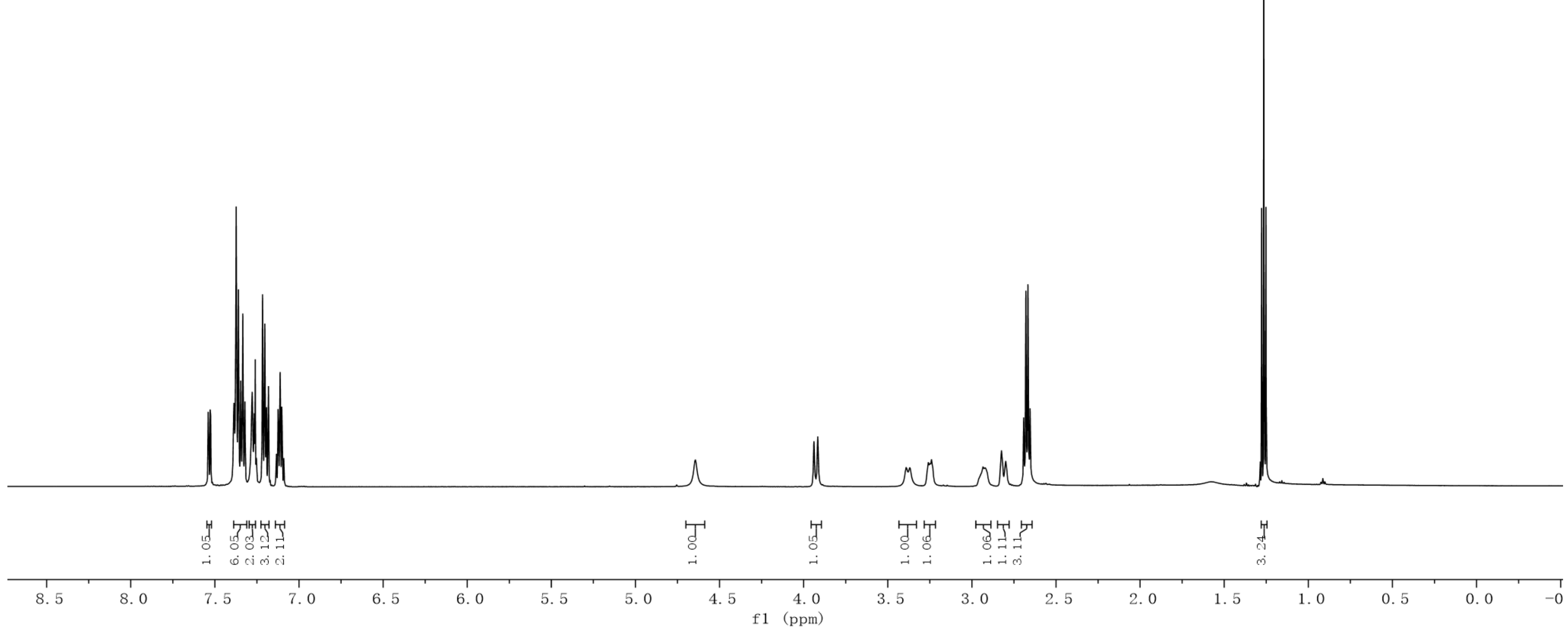

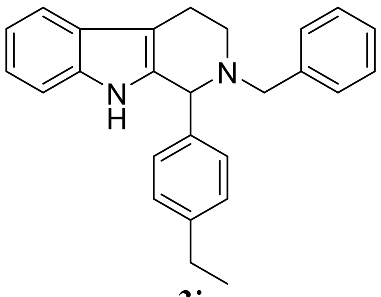

3j
(151 MHz, $CDCl_3$)

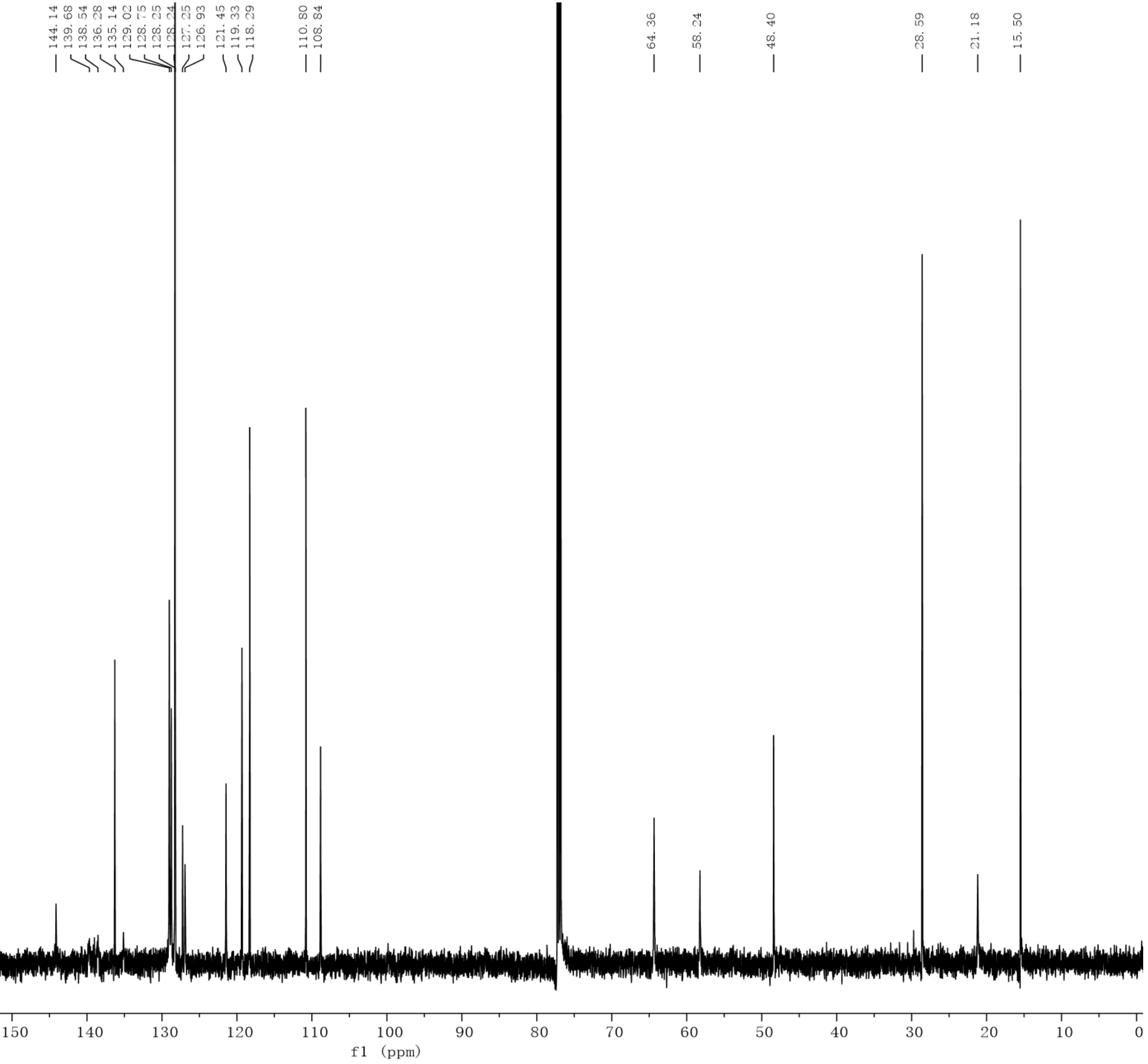

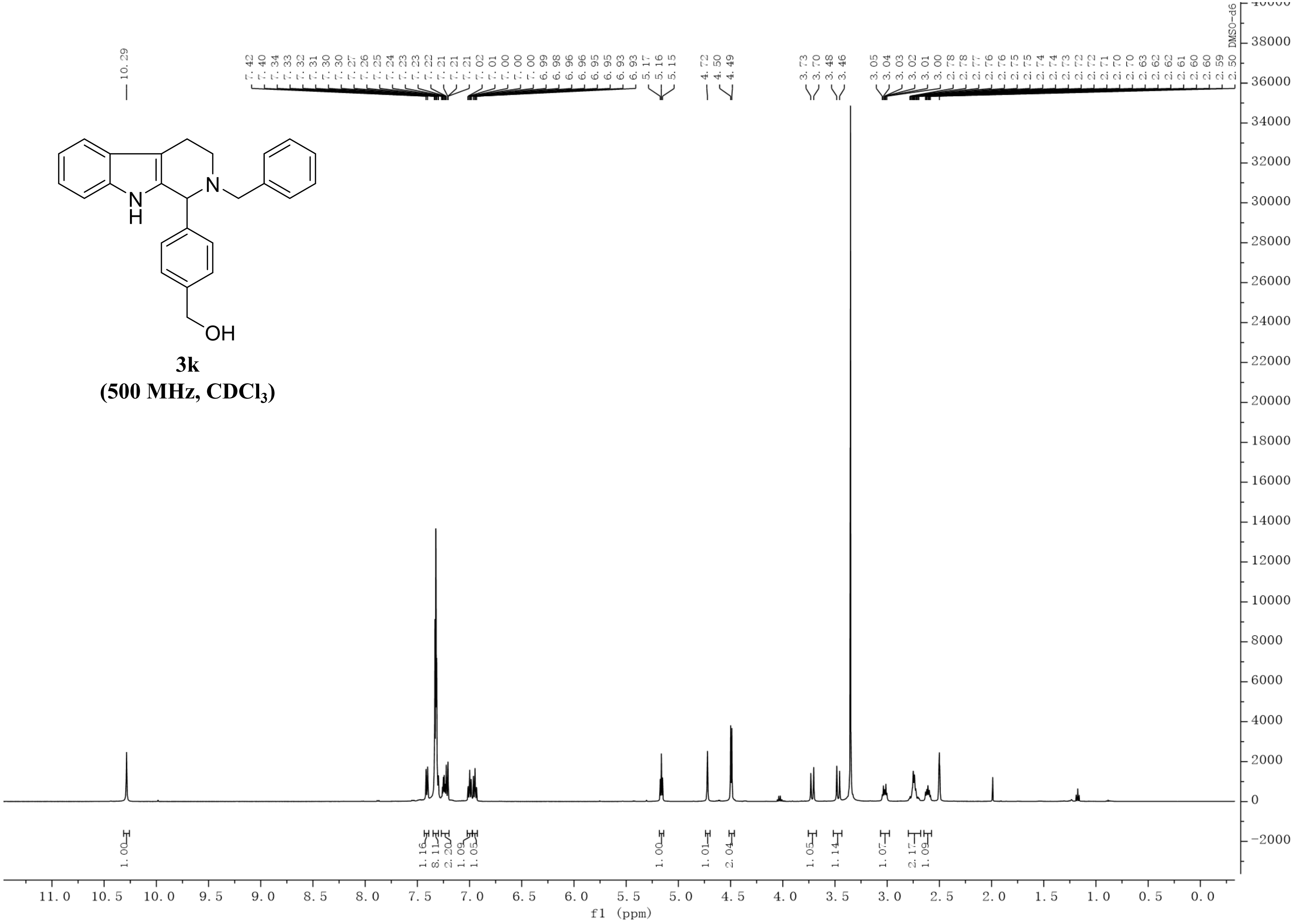

3k
(500 MHz, $CDCl_3$)

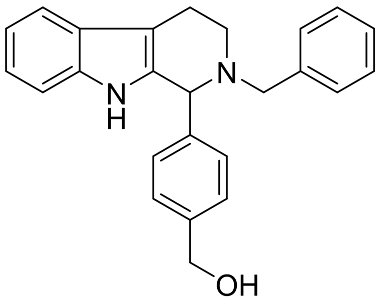

3k
(126 MHz, $CDCl_3$)

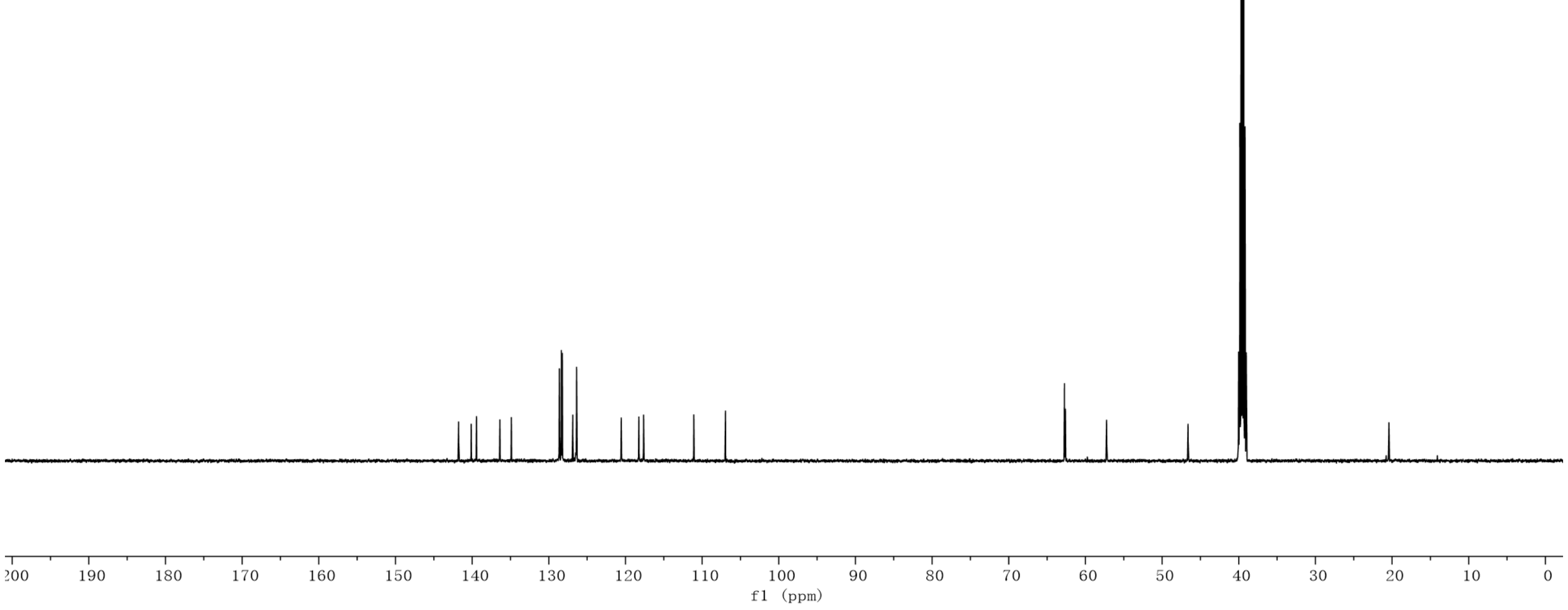

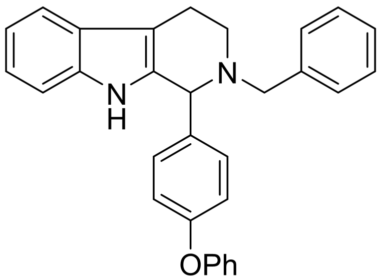

3l
(600 MHz, CDCl$_3$)

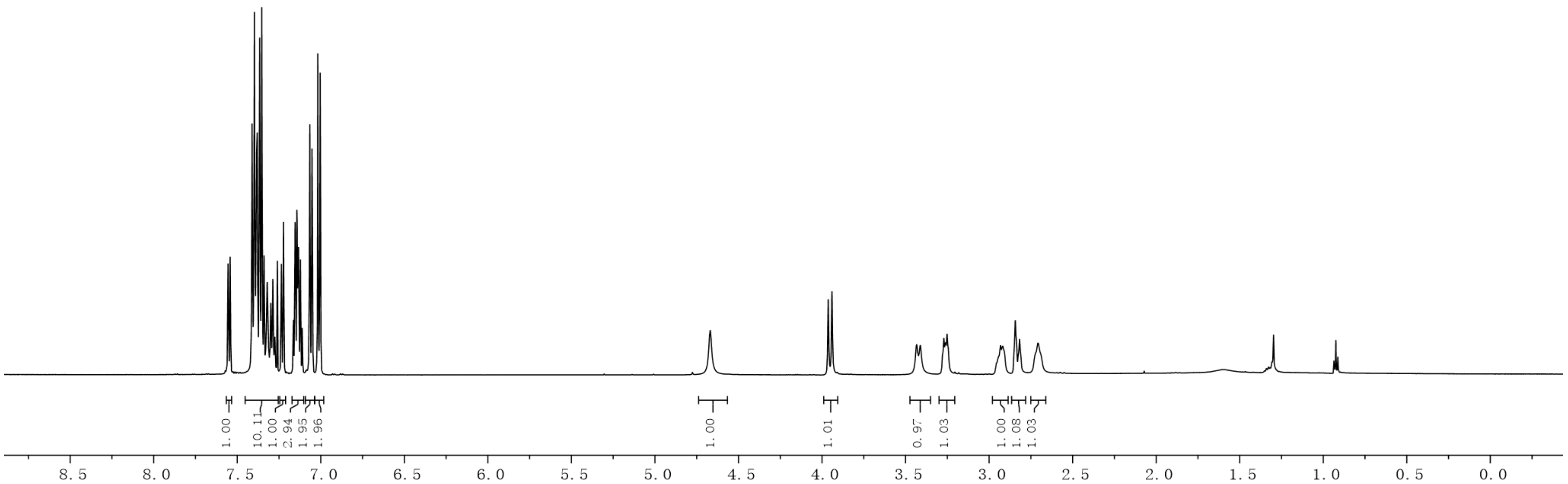

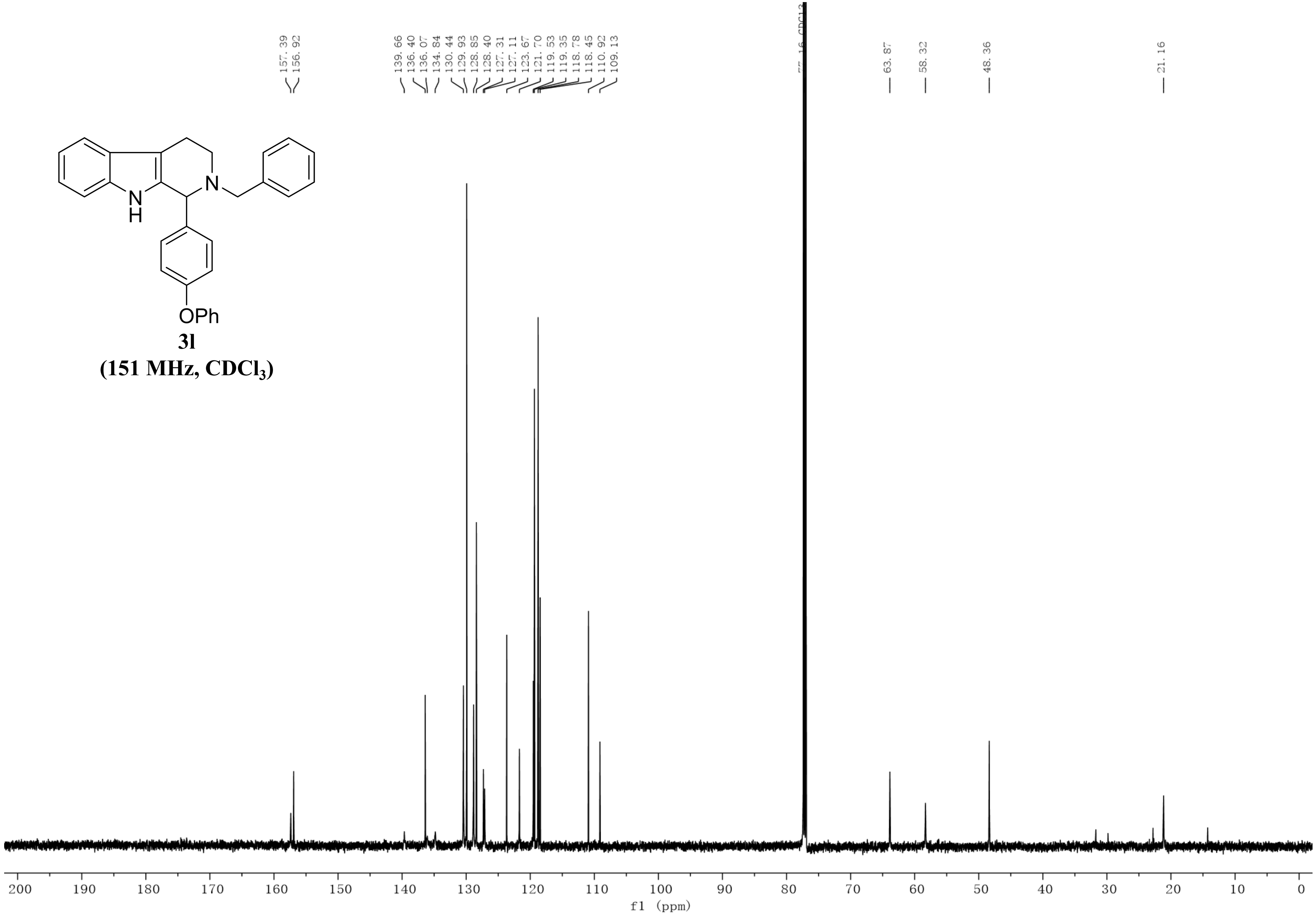

3l

(151 MHz, $CDCl_3$)

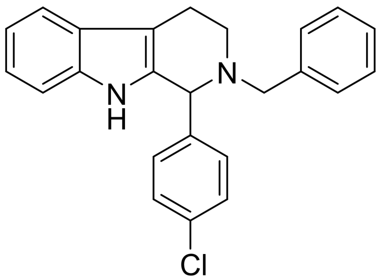

3m
(600 MHz, $CDCl_3$)

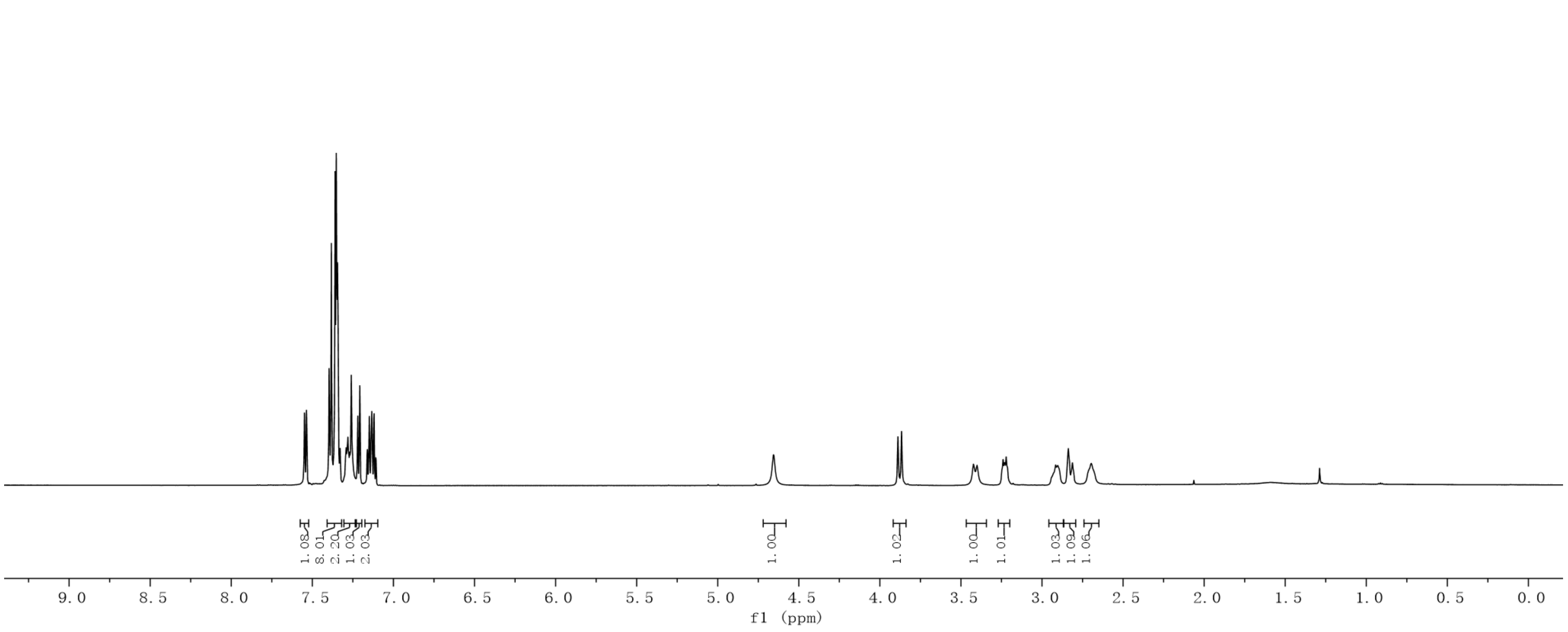

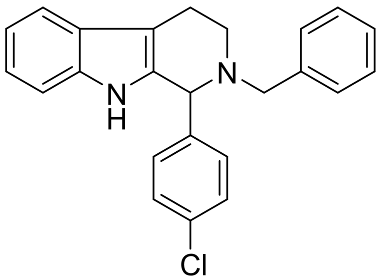

3m

(151 MHz, $CDCl_3$)

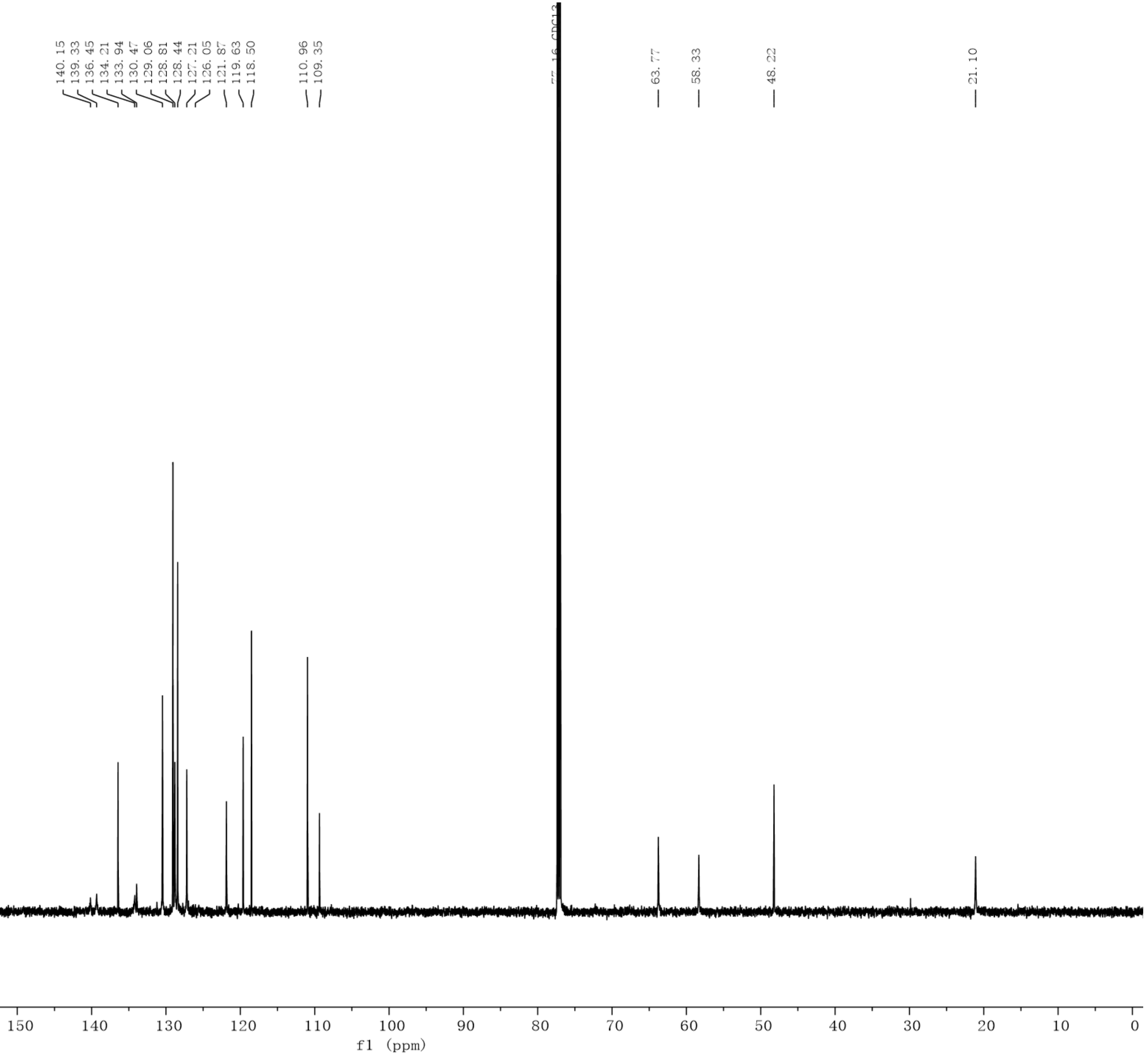

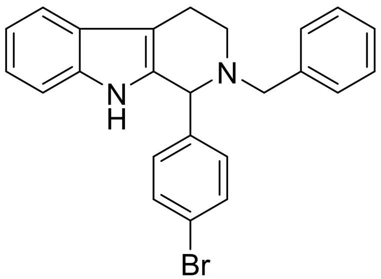

3n
(600 MHz, $CDCl_3$)

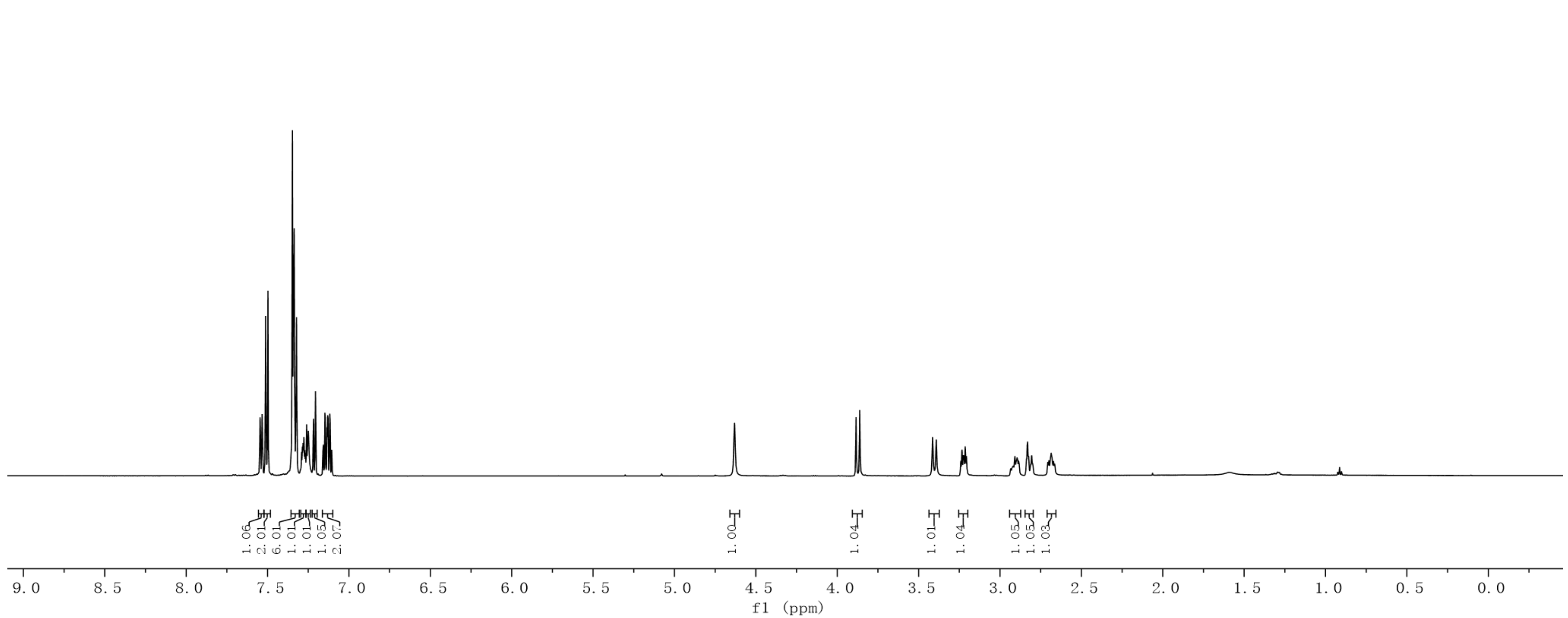

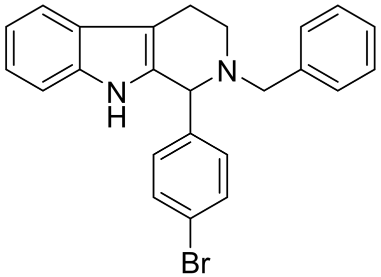

3n
(151 MHz, $CDCl_3$)

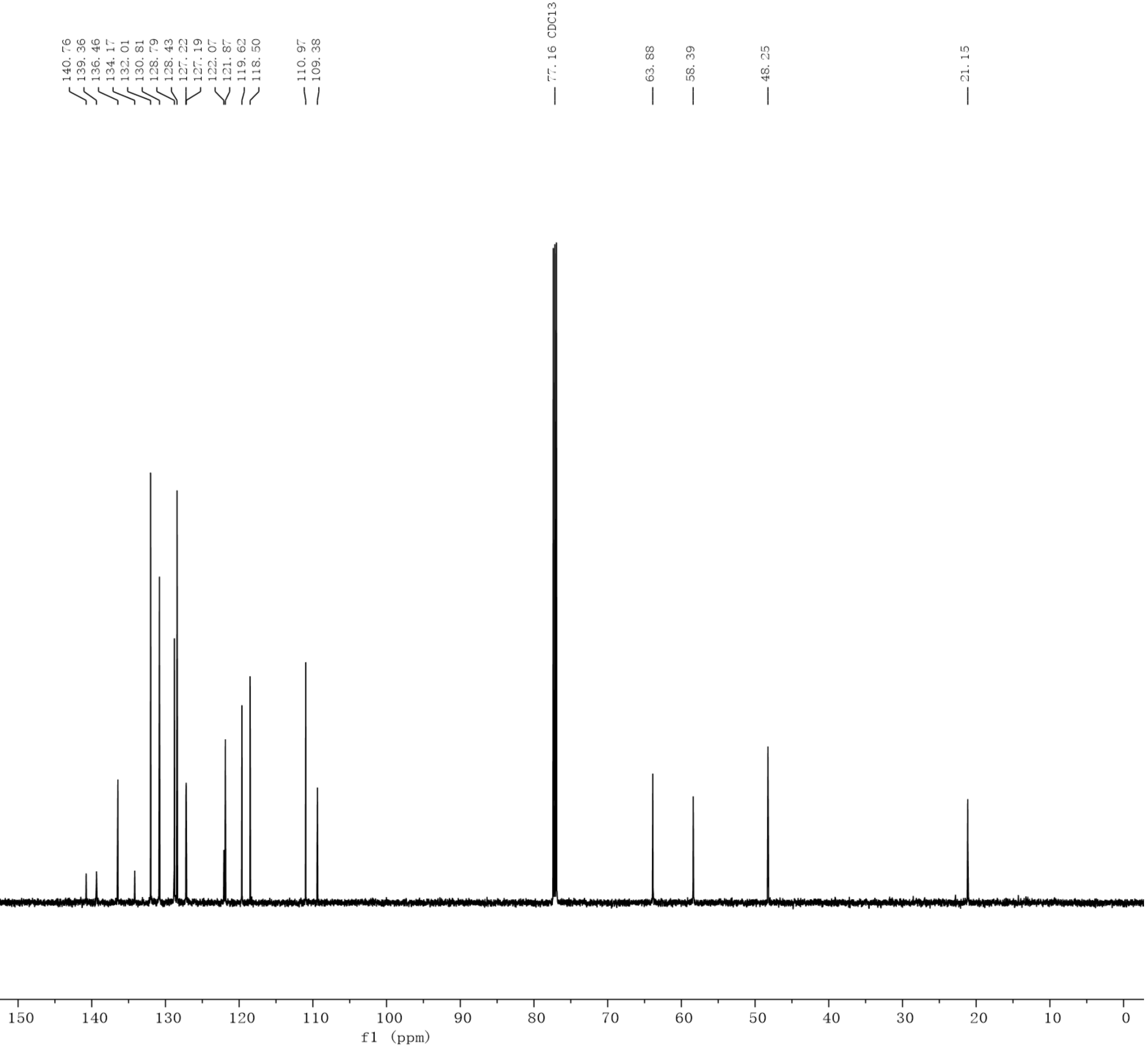

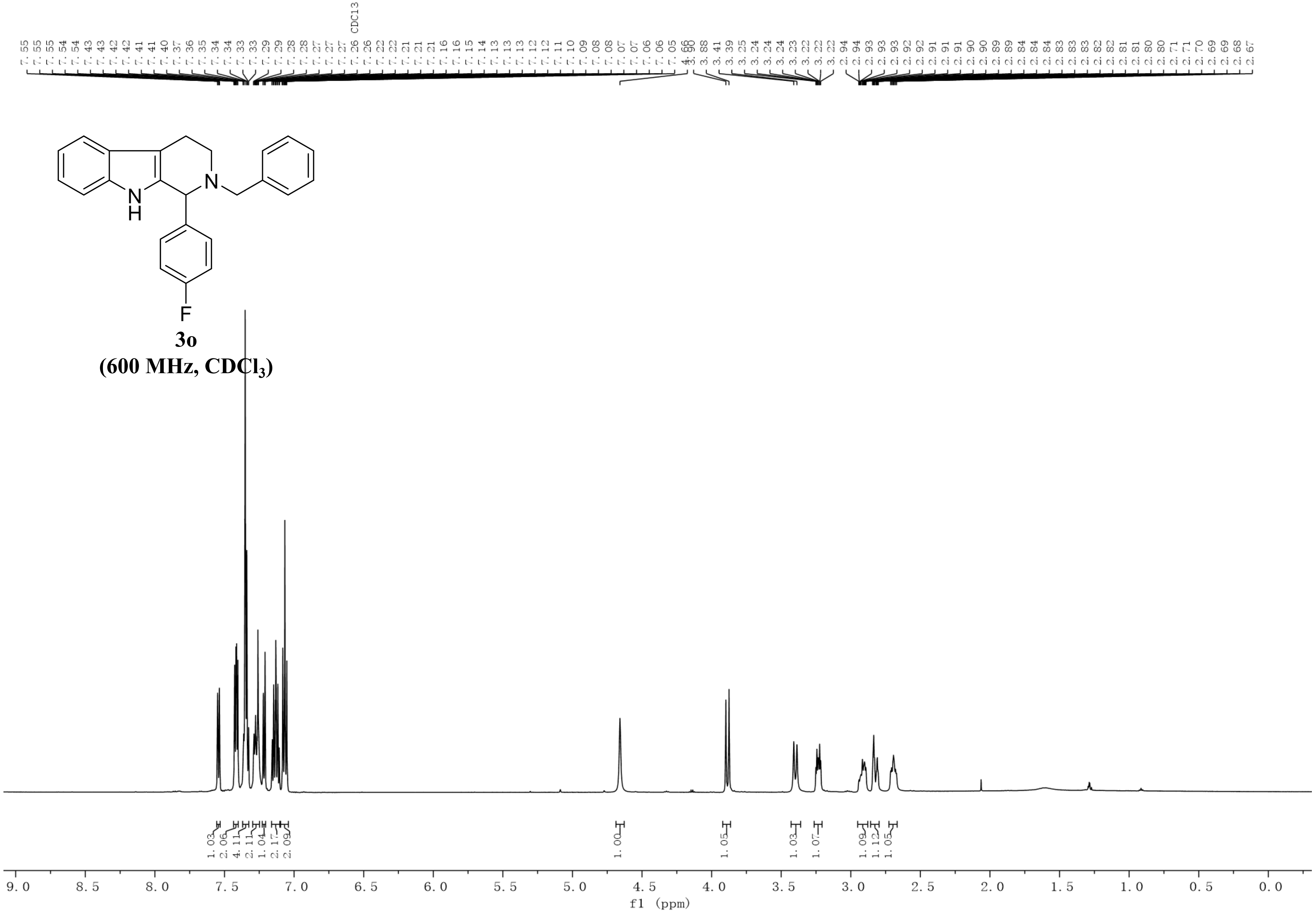

7.55 7.55 7.55 7.54 7.54 7.43 7.43 7.42 7.42 7.41 7.41 7.40 7.37 7.36 7.35 7.34 7.34 7.33 7.33 7.29 7.29 7.28 7.28 7.27 7.27 7.27 7.26 7.26 7.22 7.22 7.21 7.21 7.21 7.16 7.16 7.15 7.14 7.13 7.13 7.13 7.12 7.12 7.11 7.10 7.09 7.08 7.08 7.07 7.07 7.06 7.05 4.66 3.90 3.88 3.41 3.39 3.25 3.24 3.24 3.23 3.22 3.22 3.22 2.94 2.94 2.93 2.93 2.92 2.92 2.91 2.91 2.90 2.90 2.89 2.89 2.84 2.84 2.83 2.83 2.83 2.82 2.82 2.81 2.81 2.80 2.80 2.71 2.71 2.70 2.69 2.69 2.68 2.67
CDCl3
N
N
H
F
3o
(600 MHz, $CDCl_3$)
1.03 2.06 4.11 2.11 1.04 2.17 2.09 1.00 1.05 1.03 1.07 1.09 1.12 1.05
9.0 8.5 8.0 7.5 7.0 6.5 6.0 5.5 5.0 4.5 4.0 3.5 3.0 2.5 2.0 1.5 1.0 0.5 0.0
f1 (ppm)

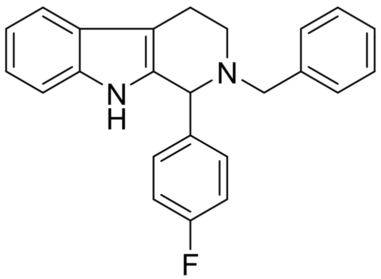

3o
(151 MHz, $CDCl_3$)

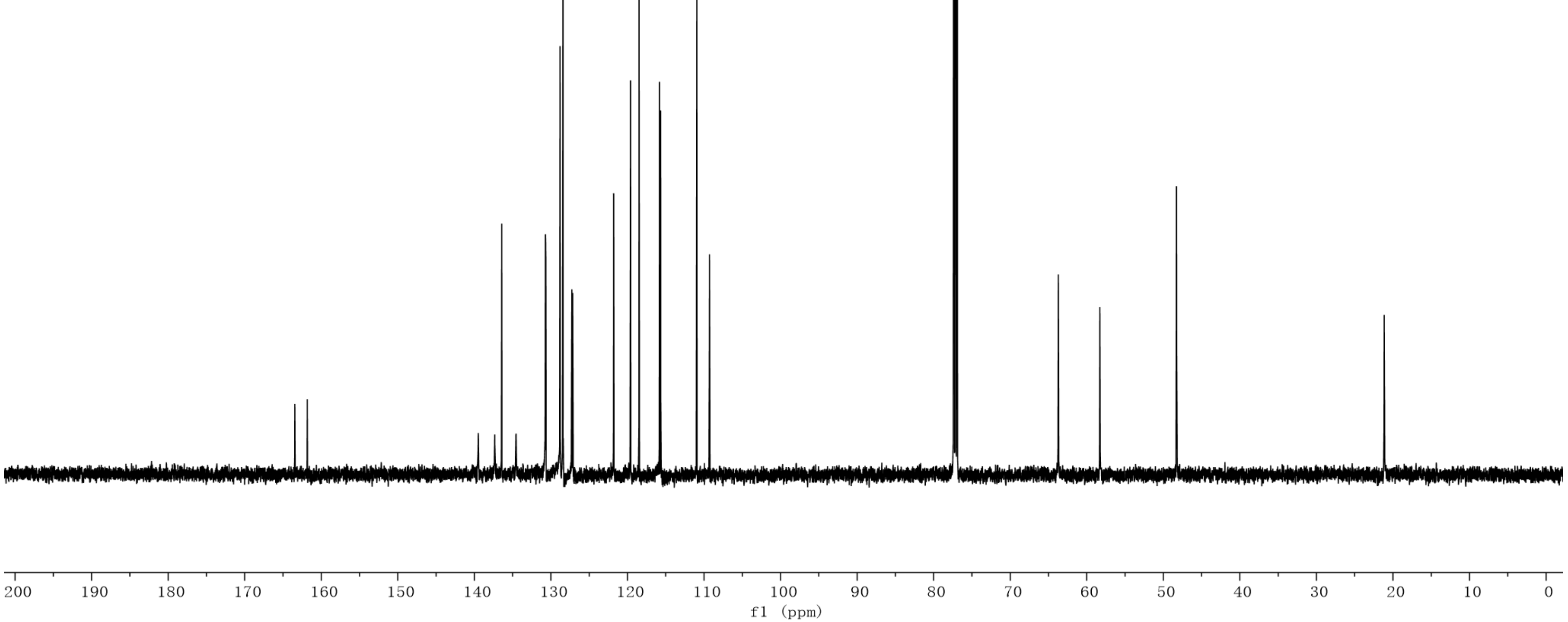

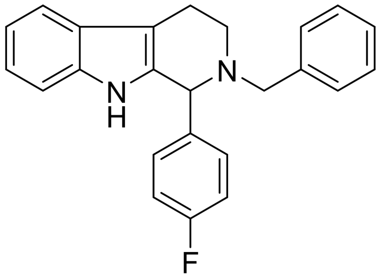

3o
(^{19}F $CDCl_3$)

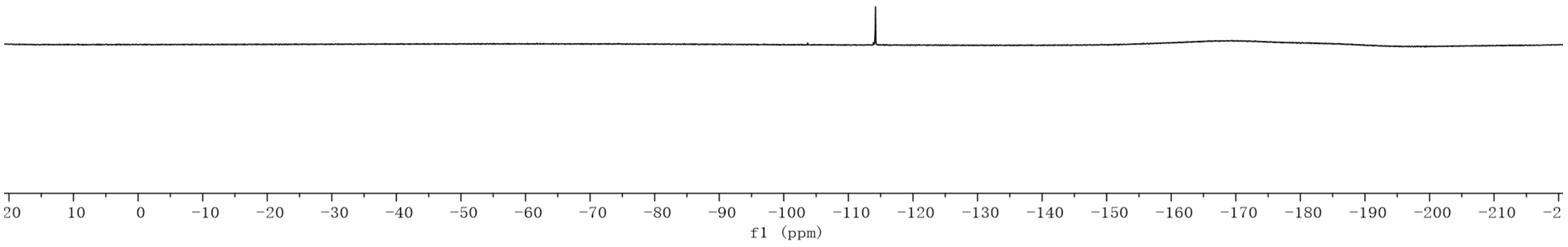

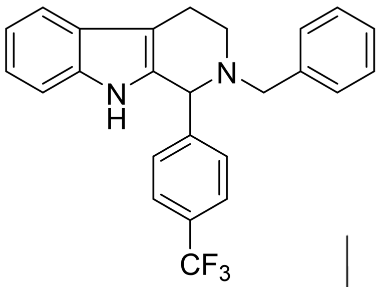

3p
(500 MHz, $CDCl_3$)

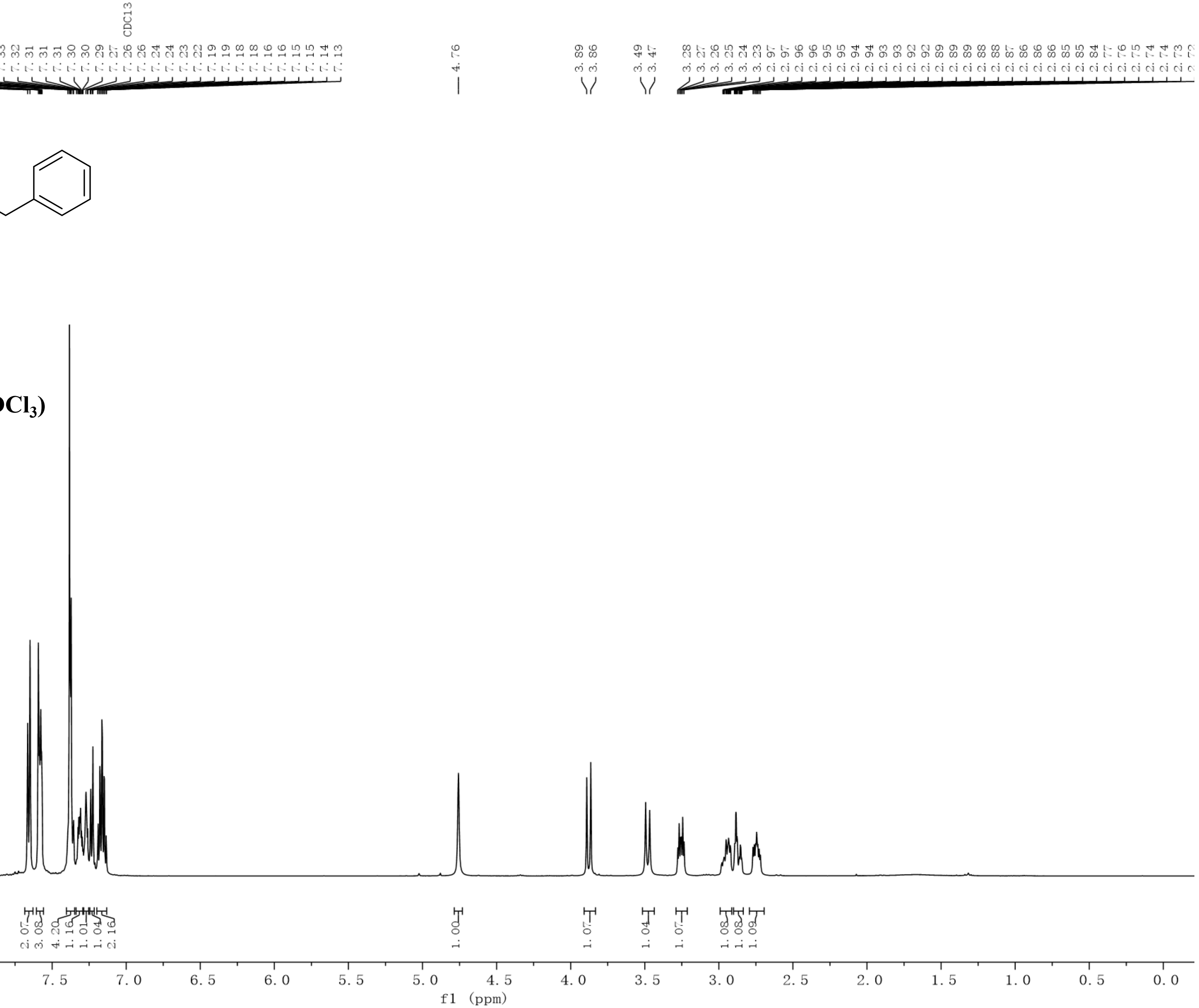

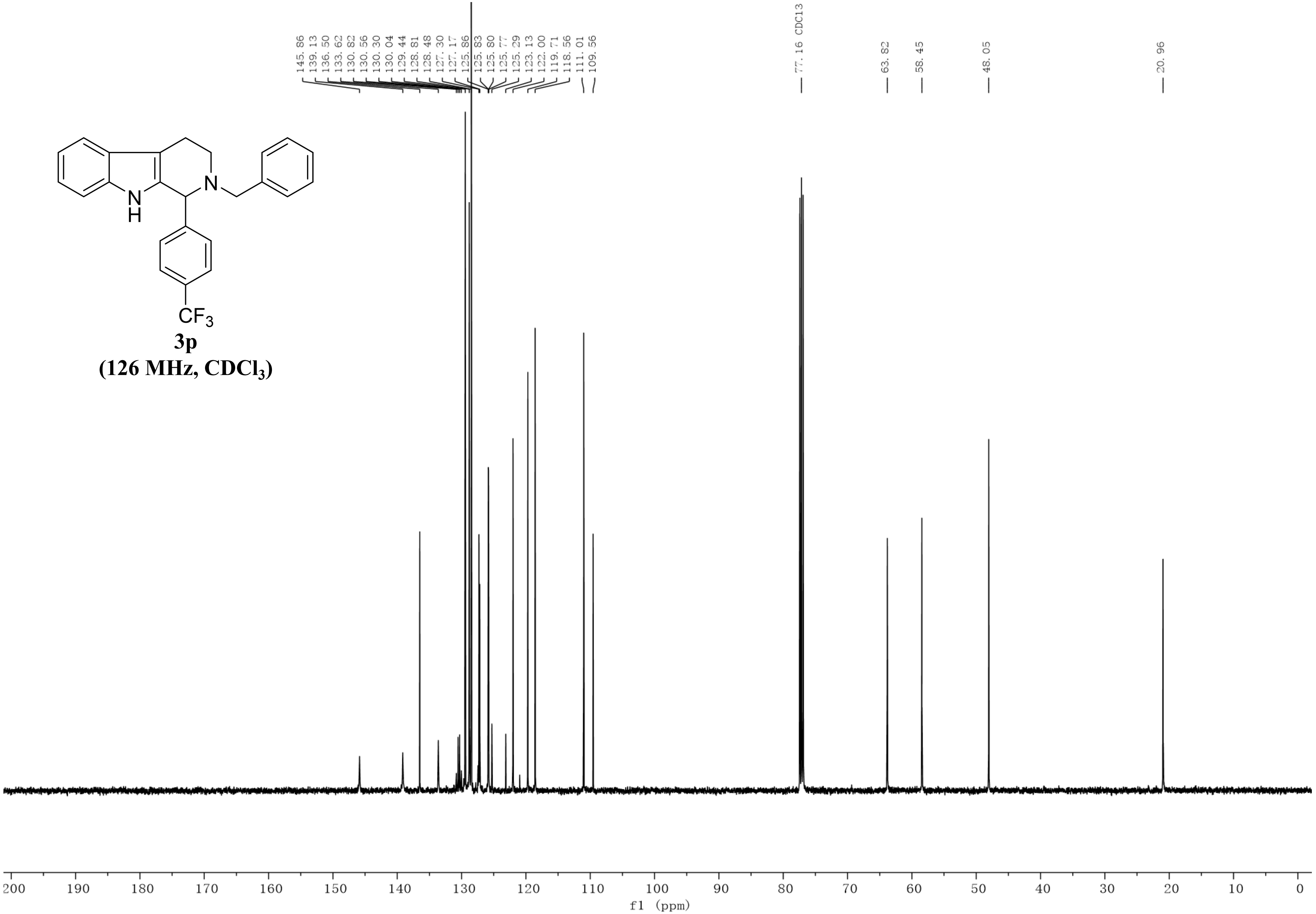
145.86
139.13
136.50
133.62
130.82
130.56
130.30
130.04
129.44
128.81
128.48
127.30
127.17
125.86
125.83
125.80
125.77
125.29
123.13
122.00
119.71
118.56
111.01
109.56
77.16 CDCl3
63.82
58.45
48.05
20.96
N
NH
CF3
3p
(126 MHz, CDCl3)
200
190
180
170
160
150
140
130
120
110
100
90
80
70
60
50
40
30
20
10
0
f1 (ppm)

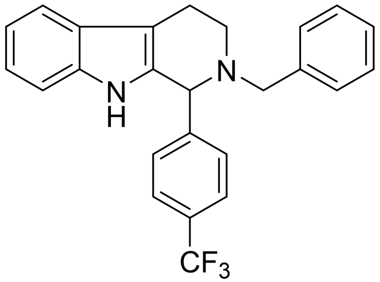
N
H
N
CF3

3p
(^{19}F CDCl$_3$)

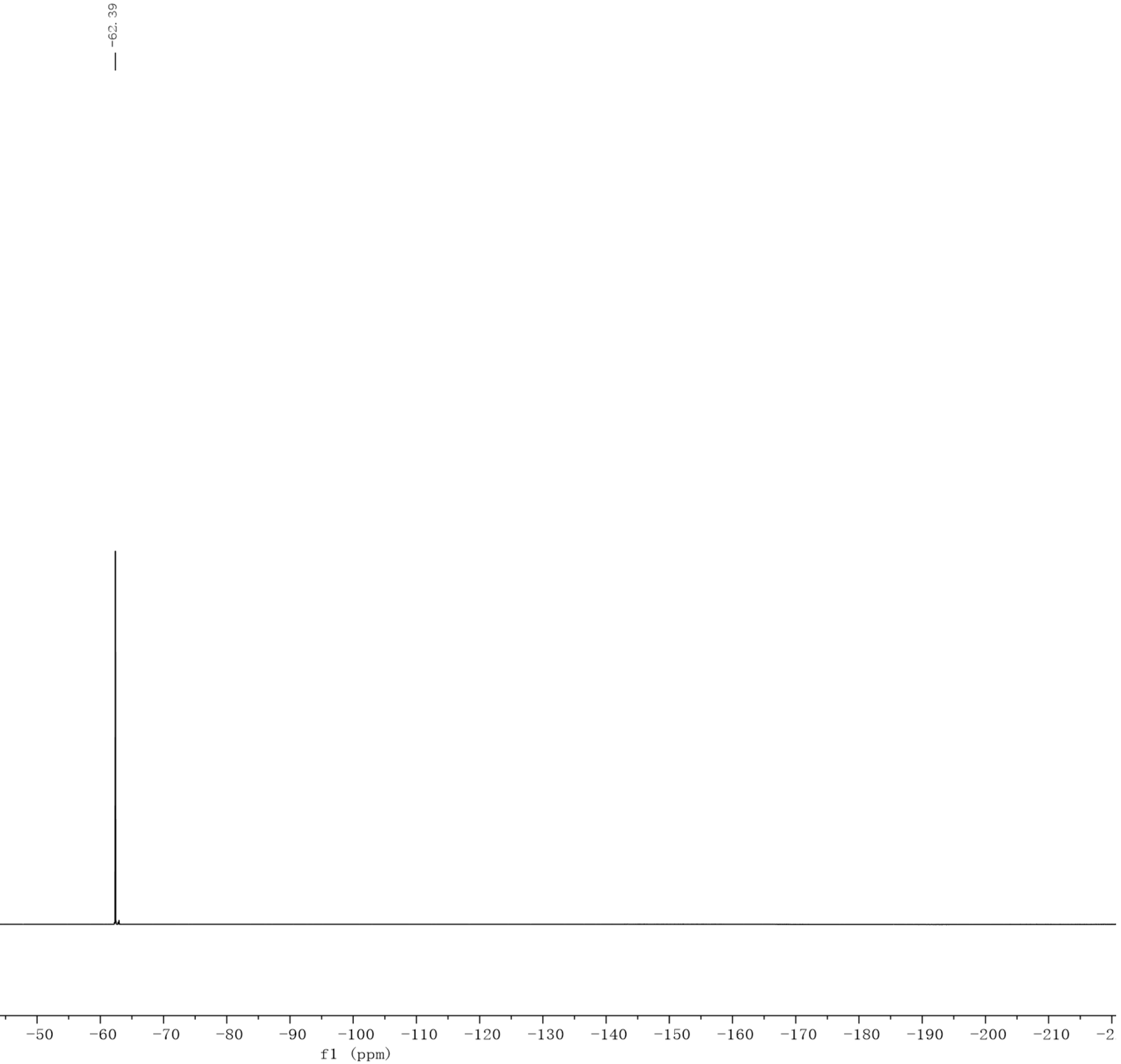
−62.39
20
10
0
−10
−20
−30
−40
−50
−60
−70
−80
−90
−100
−110
−120
−130
−140
−150
−160
−170
−180
−190
−200
−210
−2
f1 (ppm)

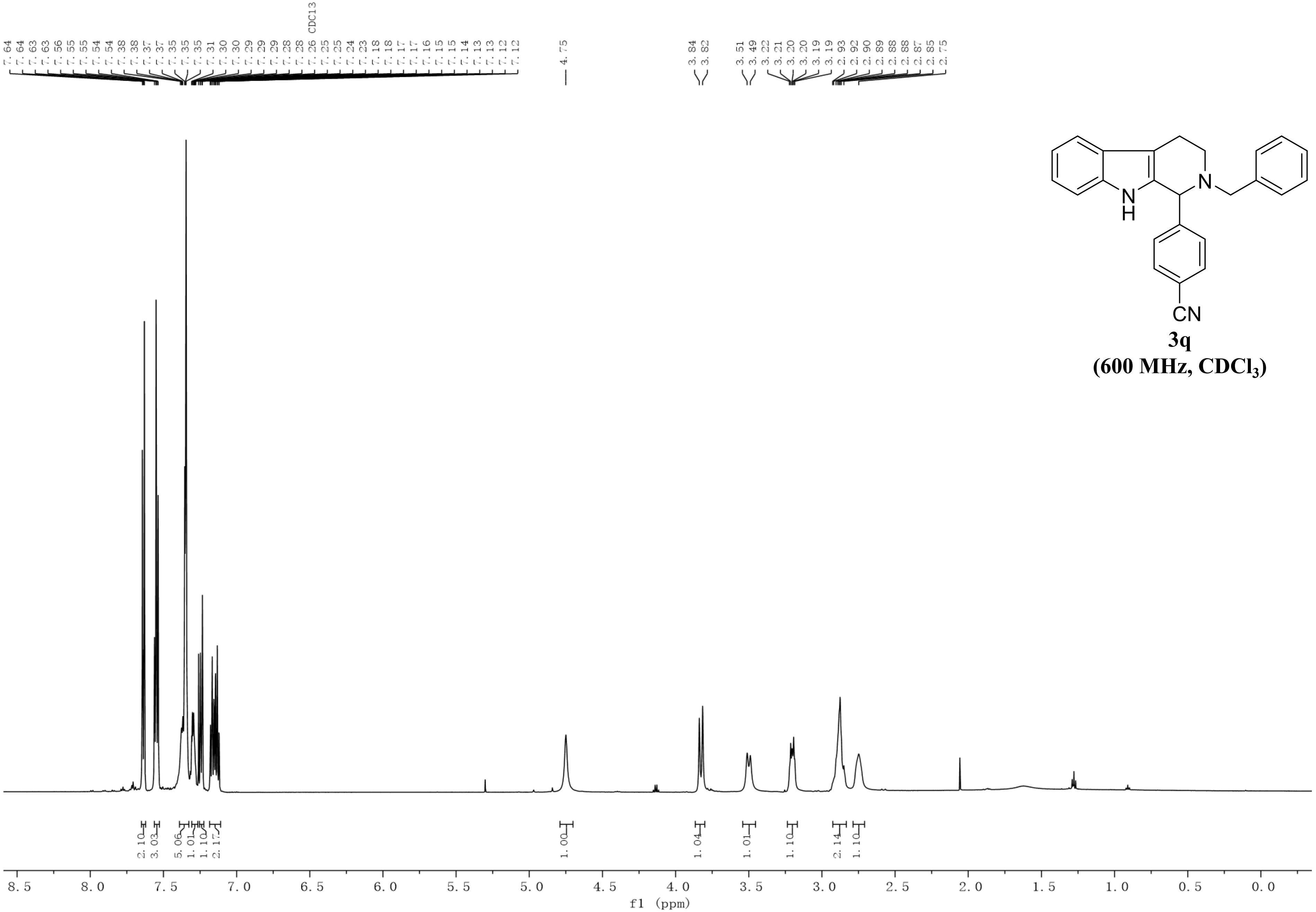

3q
(600 MHz, $CDCl_3$)

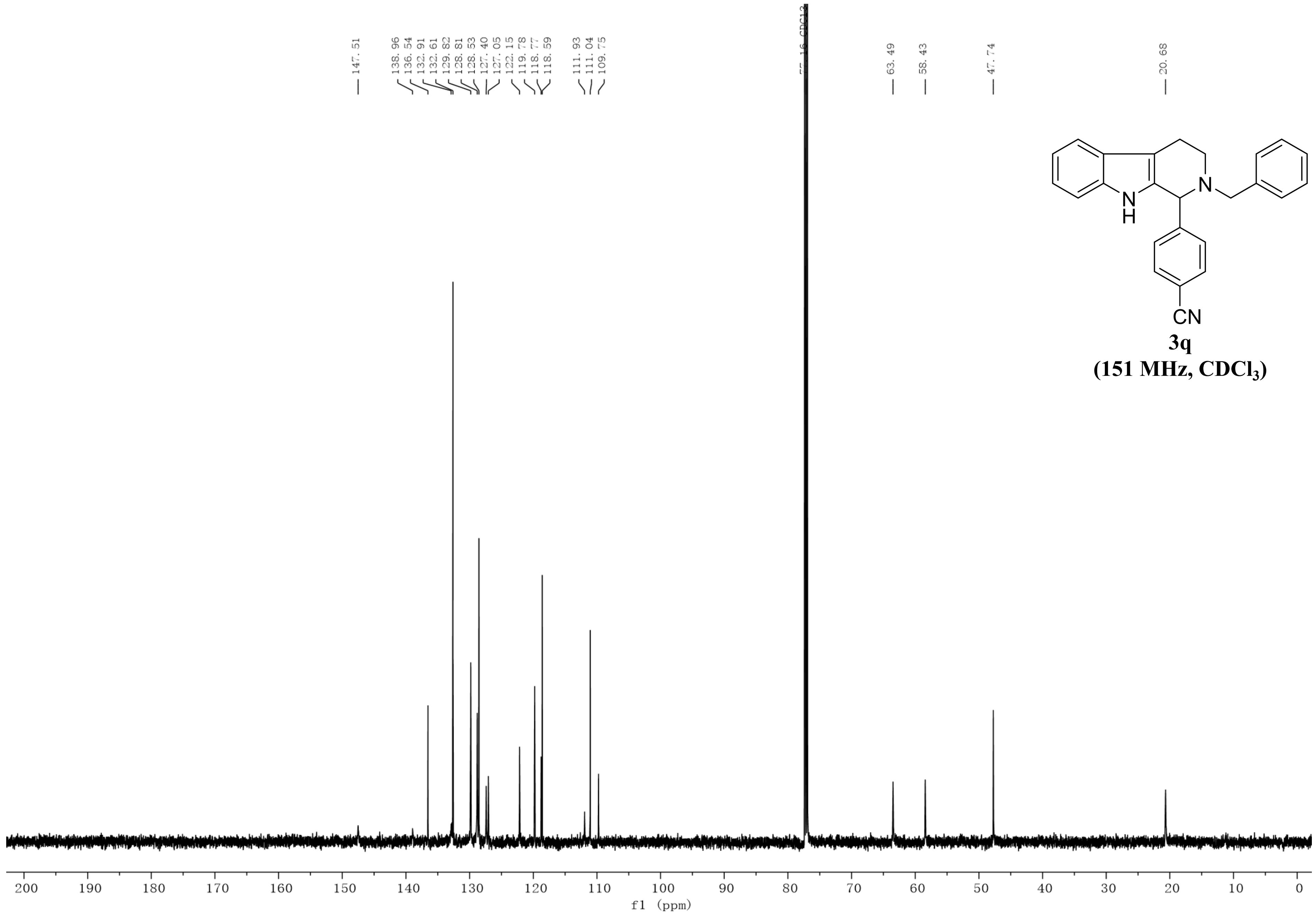

3q

(151 MHz, $CDCl_3$)

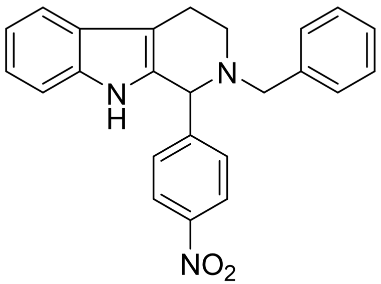

3r
(600 MHz, $CDCl_3$)

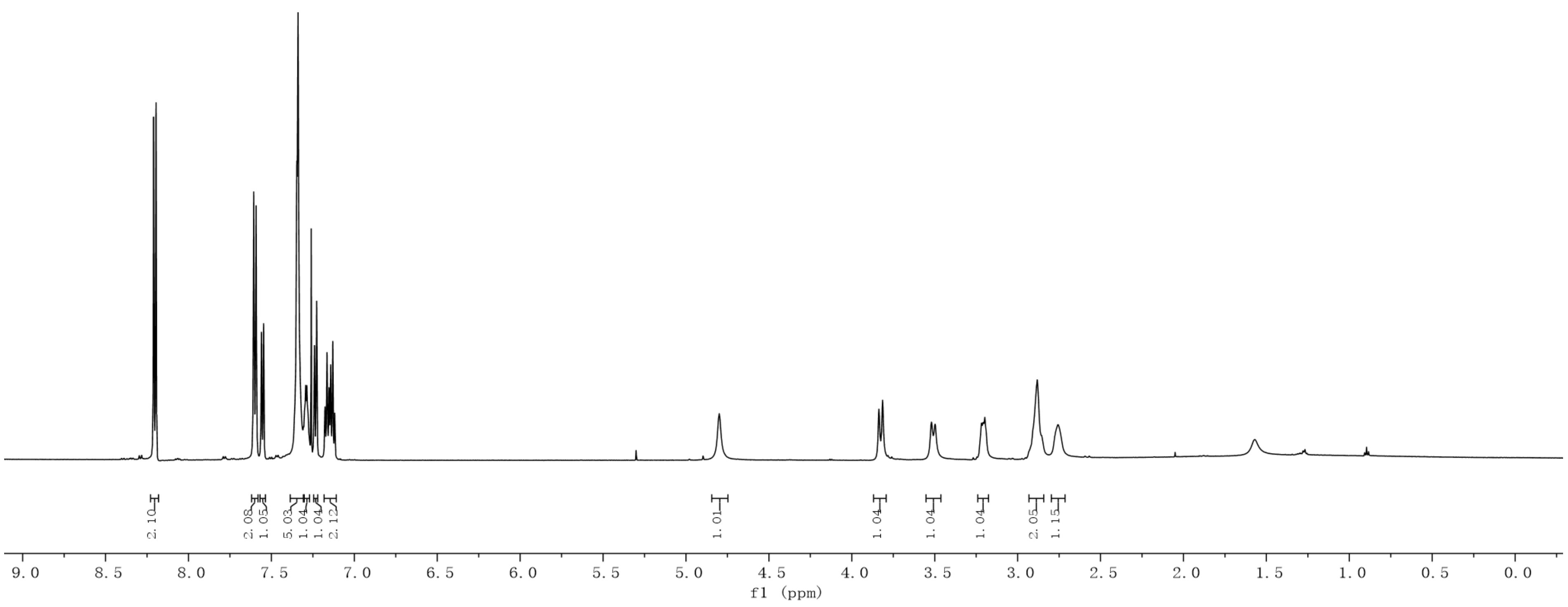

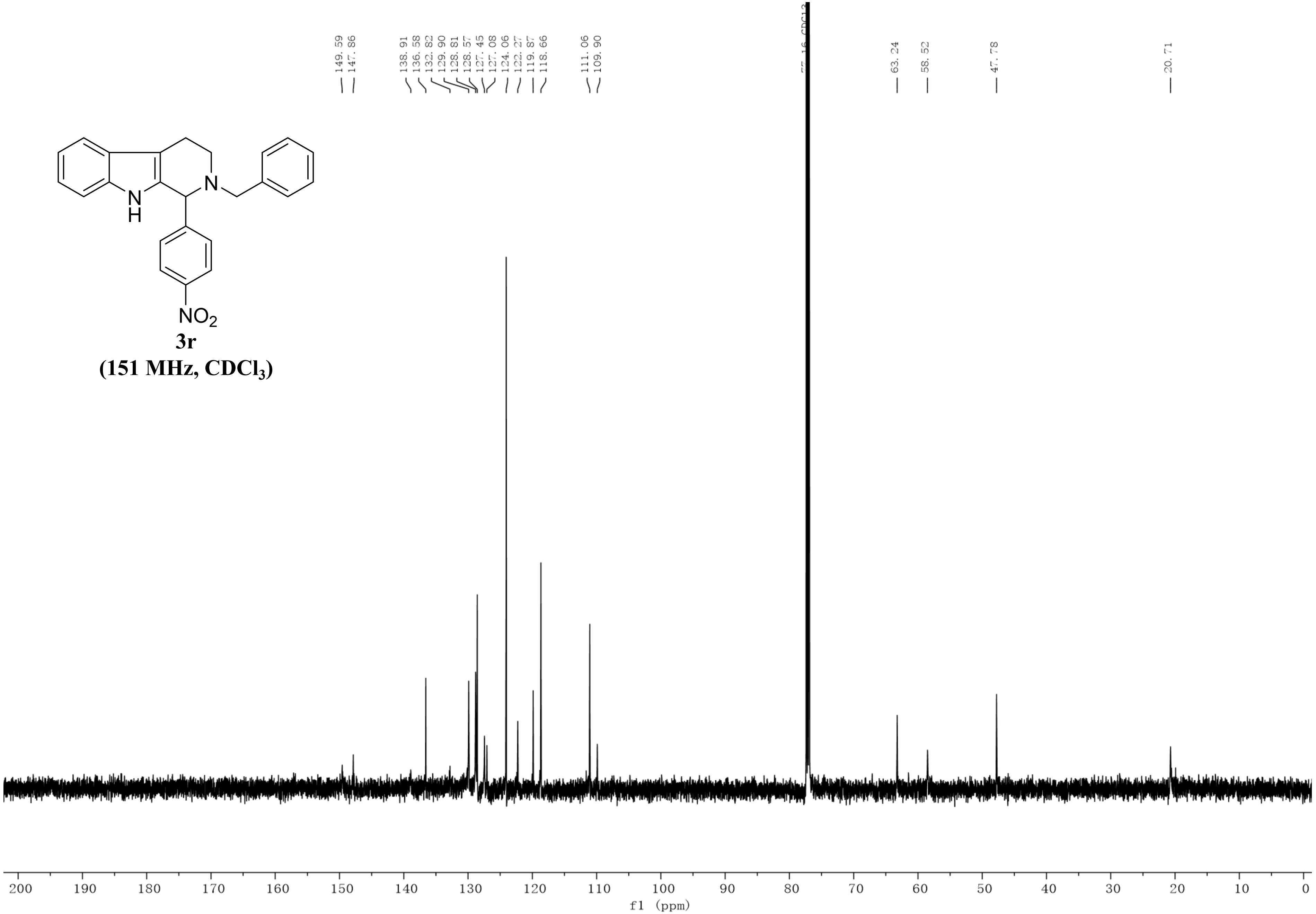

3r

(151 MHz, $CDCl_3$)

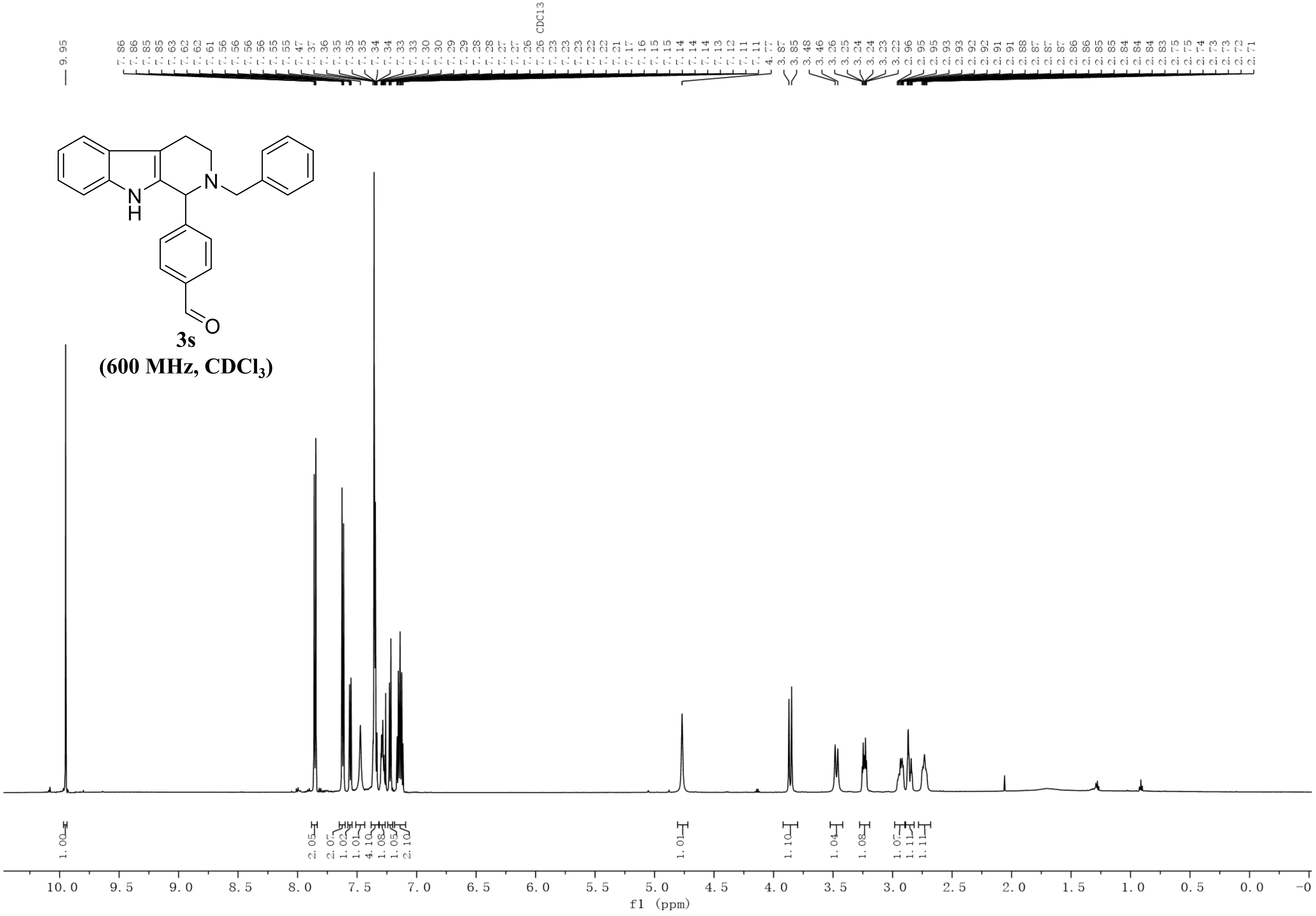

3s
(600 MHz, $CDCl_3$)

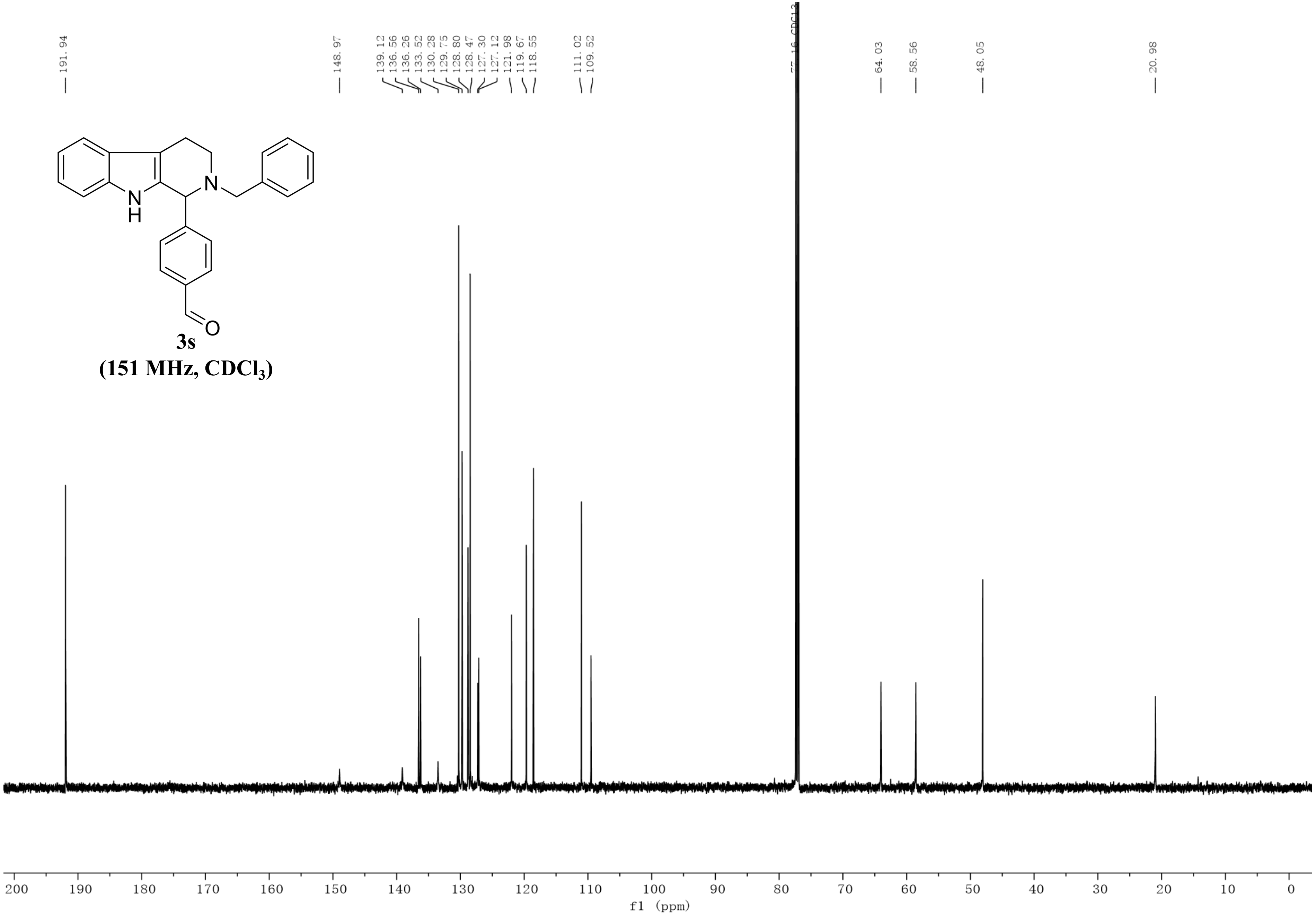
191.94
148.97
139.12
136.56
136.26
133.52
130.28
129.75
128.80
128.47
127.30
127.12
121.98
119.67
118.55
111.02
109.52
77.16 CDCl3
64.03
58.56
48.05
20.98
N
N
H
O
3s
(151 MHz, CDCl3)
200 190 180 170 160 150 140 130 120 110 100 90 80 70 60 50 40 30 20 10 0
f1 (ppm)

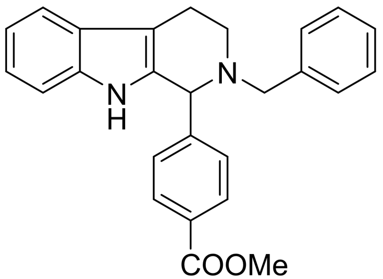

3t
(500 MHz, $CDCl_3$)

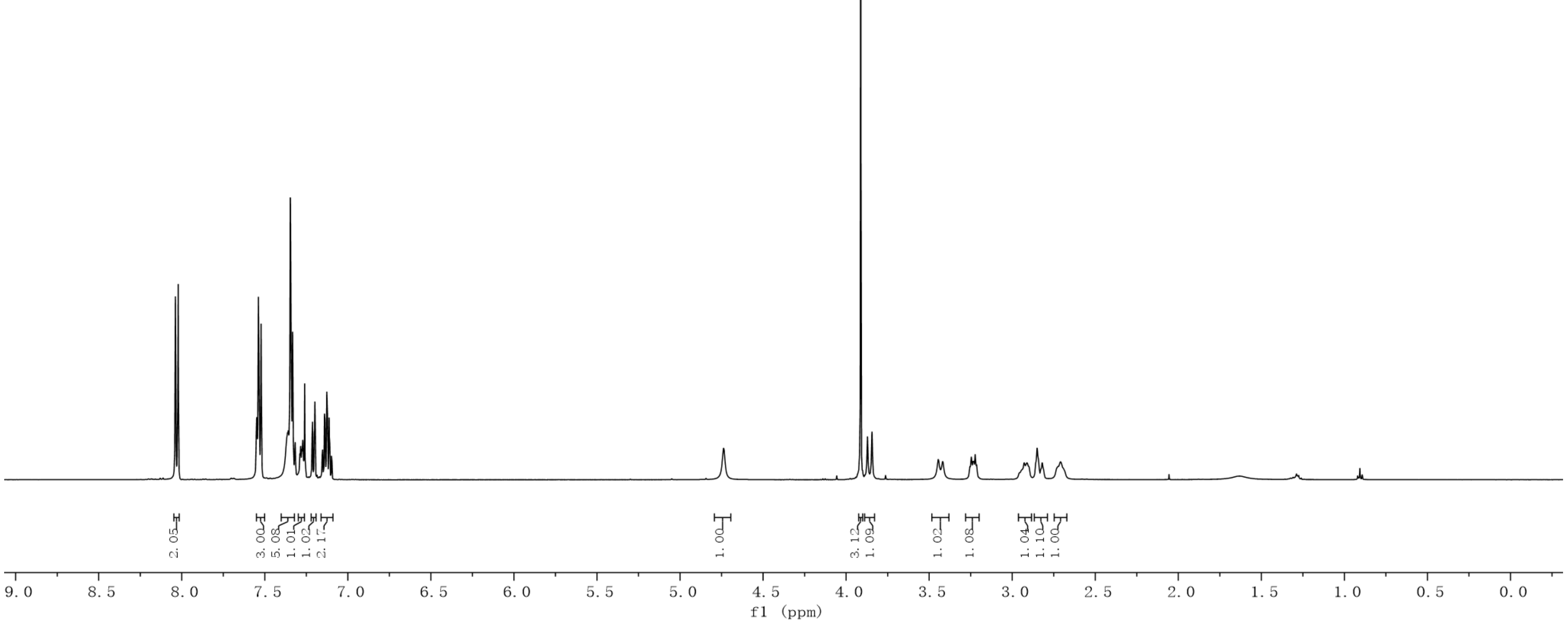

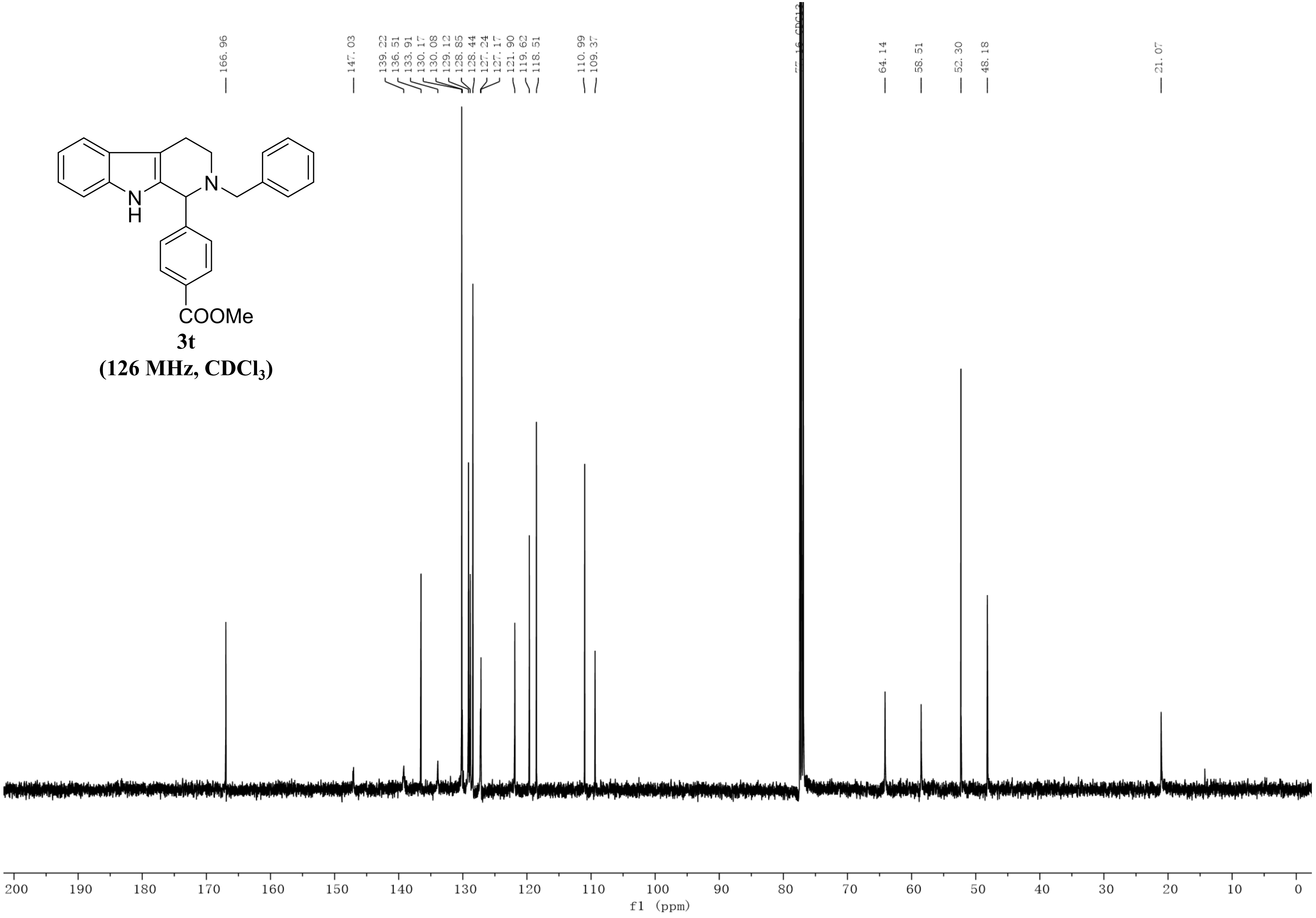

3t
(126 MHz, $CDCl_3$)

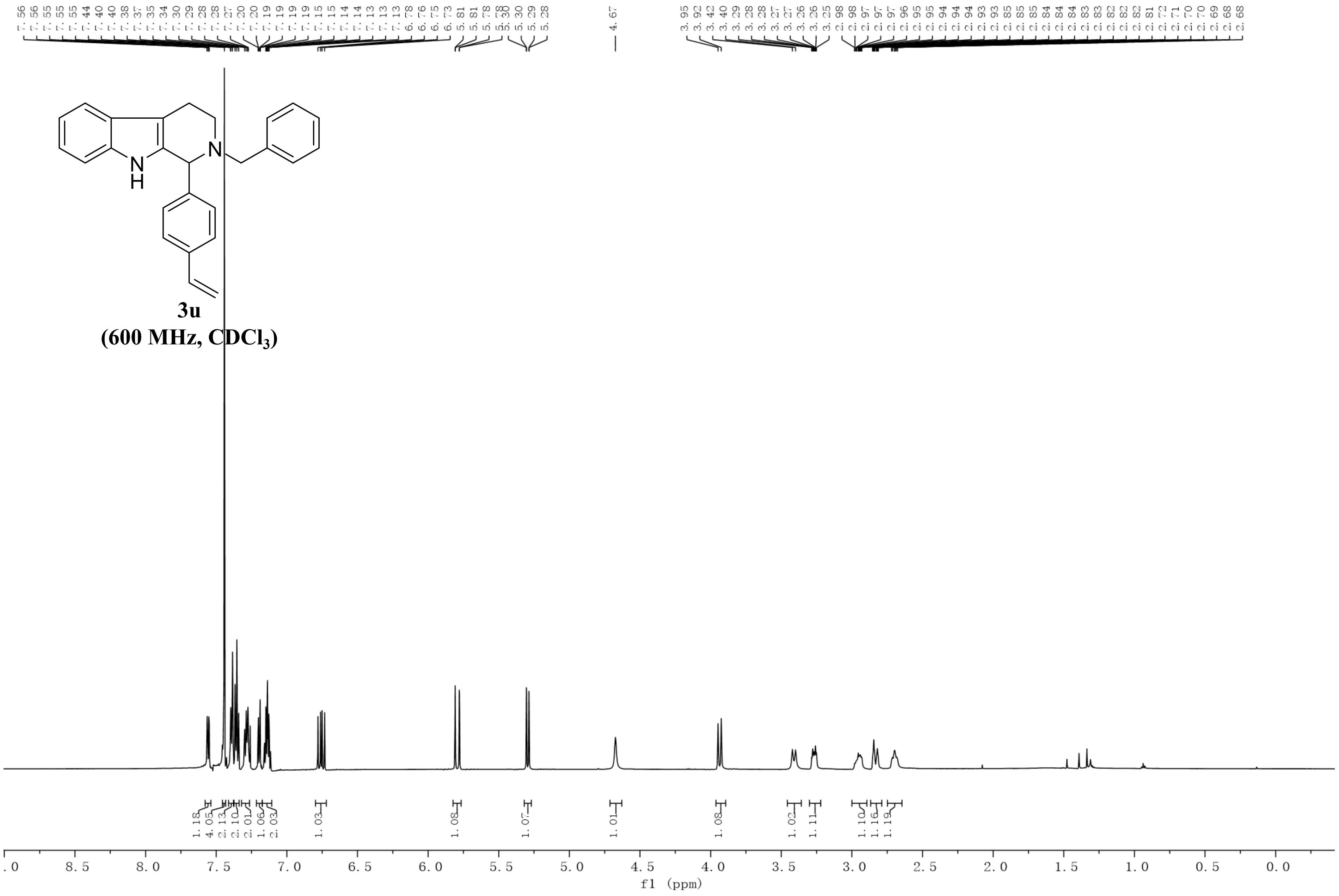
3u
(600 MHz, CDCl3)
f1 (ppm)

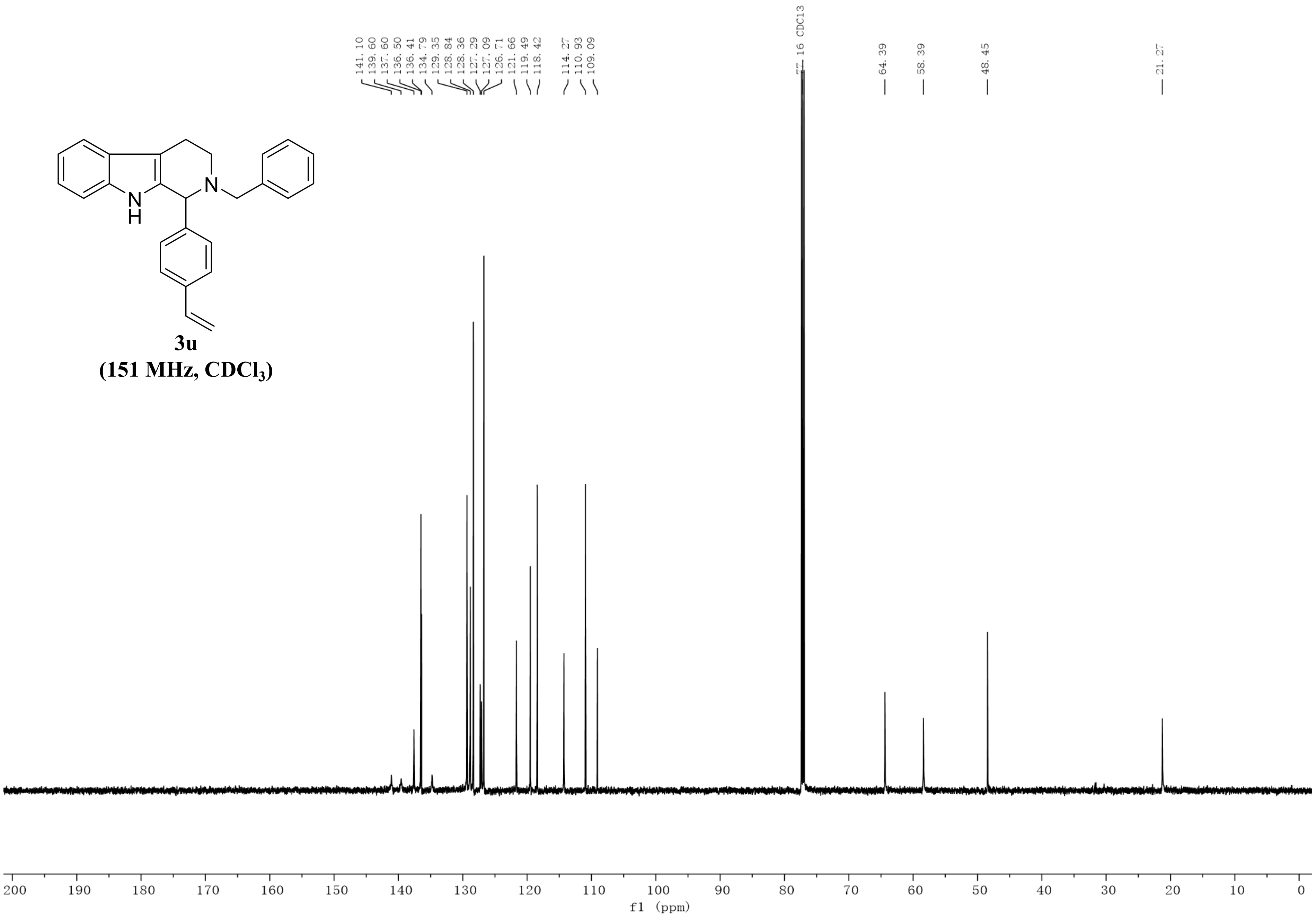
3u
(151 MHz, CDCl3)
141.10
139.60
137.60
136.50
136.41
134.79
129.35
128.84
128.36
127.29
127.09
126.71
121.66
119.49
118.42
114.27
110.93
109.09
77.16 CDCl3
64.39
58.39
48.45
21.27
200
190
180
170
160
150
140
130
120
110
100
90
80
70
60
50
40
30
20
10
0
f1 (ppm)

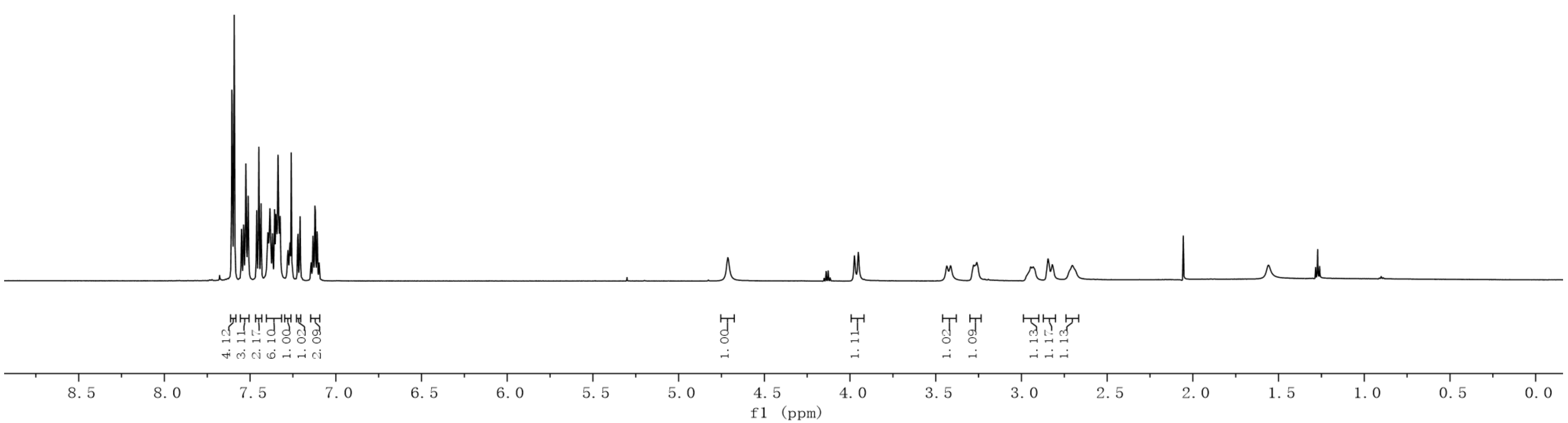

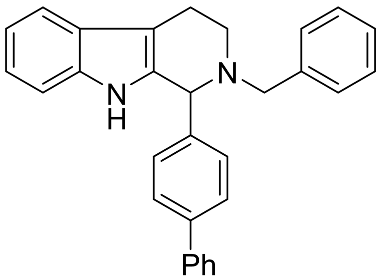

3v
(600 MHz, $CDCl_3$)

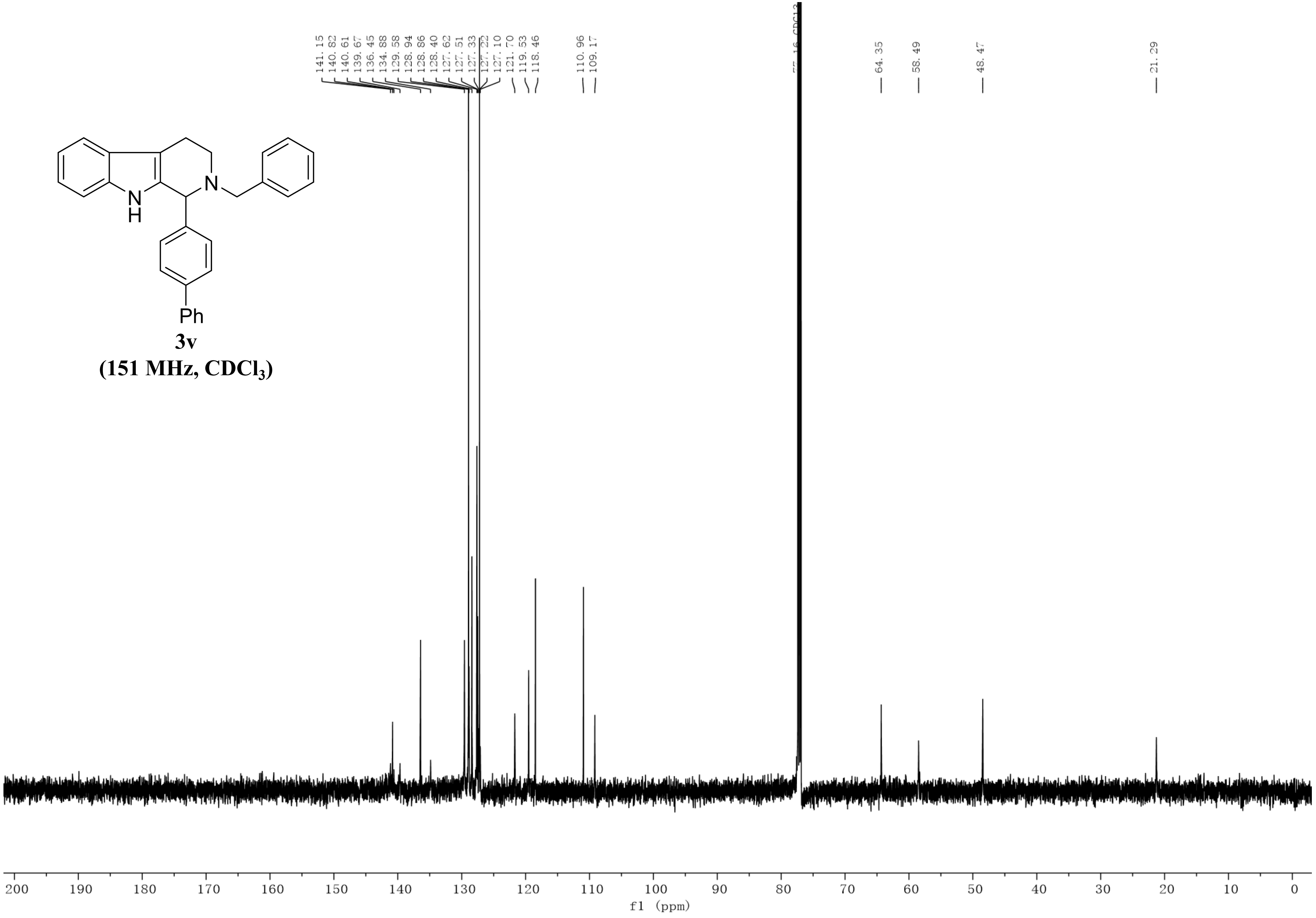

3v
(151 MHz, $CDCl_3$)

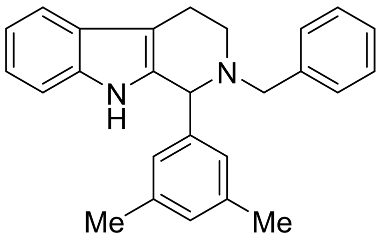

3w
(600 MHz, $CDCl_3$)

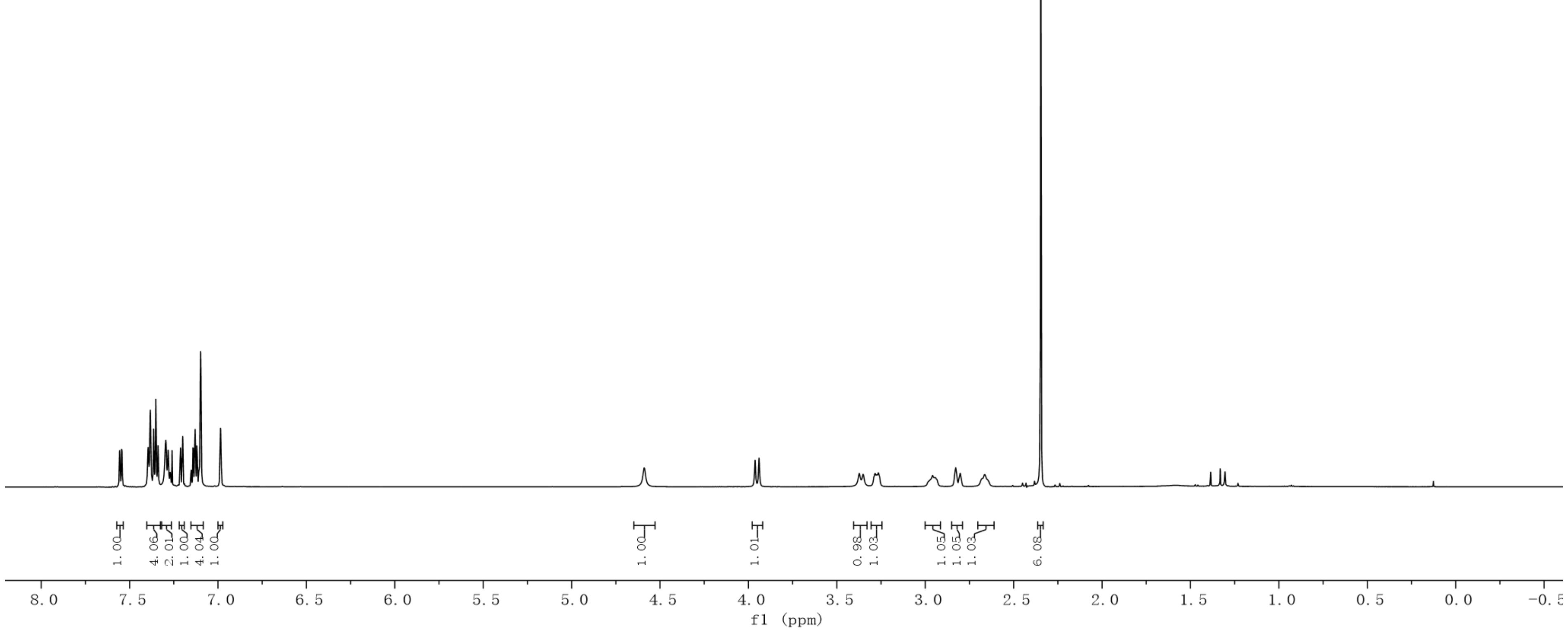

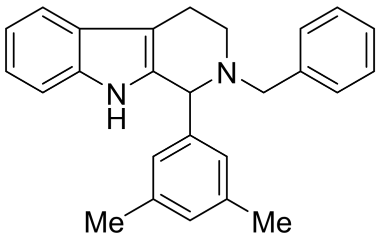

3w
(151 MHz, $CDCl_3$)

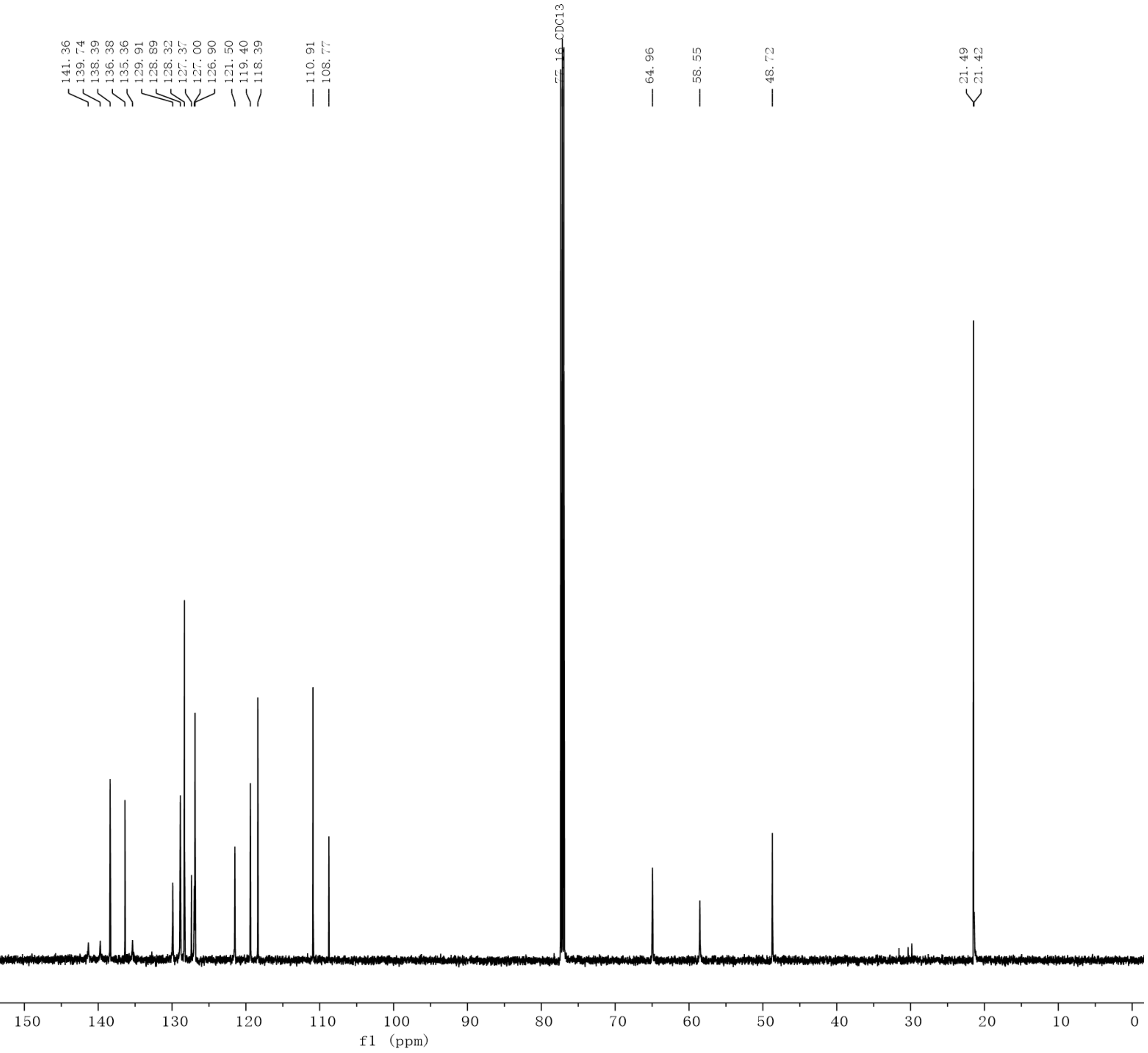

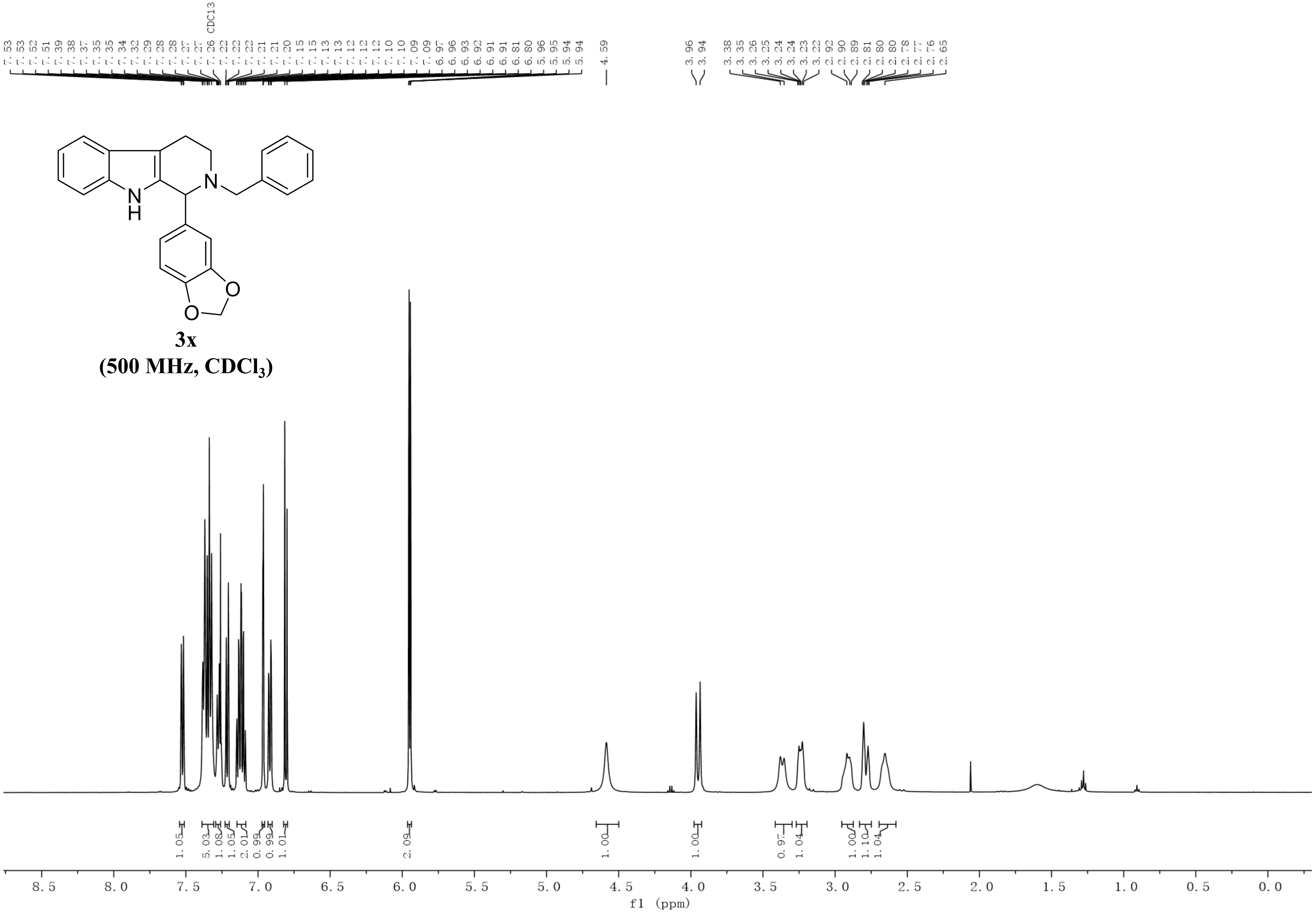

3x

(500 MHz, $CDCl_3$)

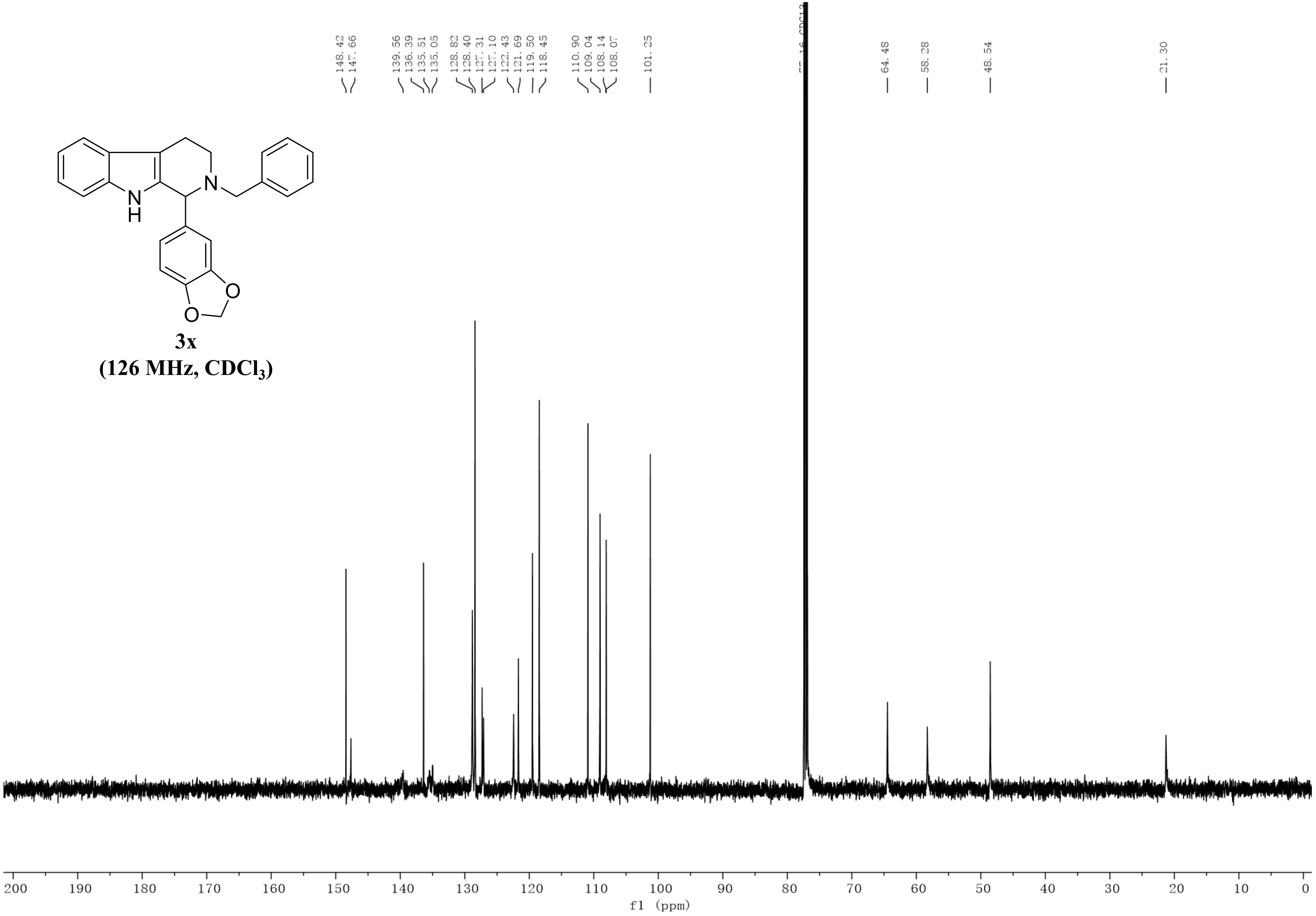

3x
(126 MHz, CDCl$_3$)

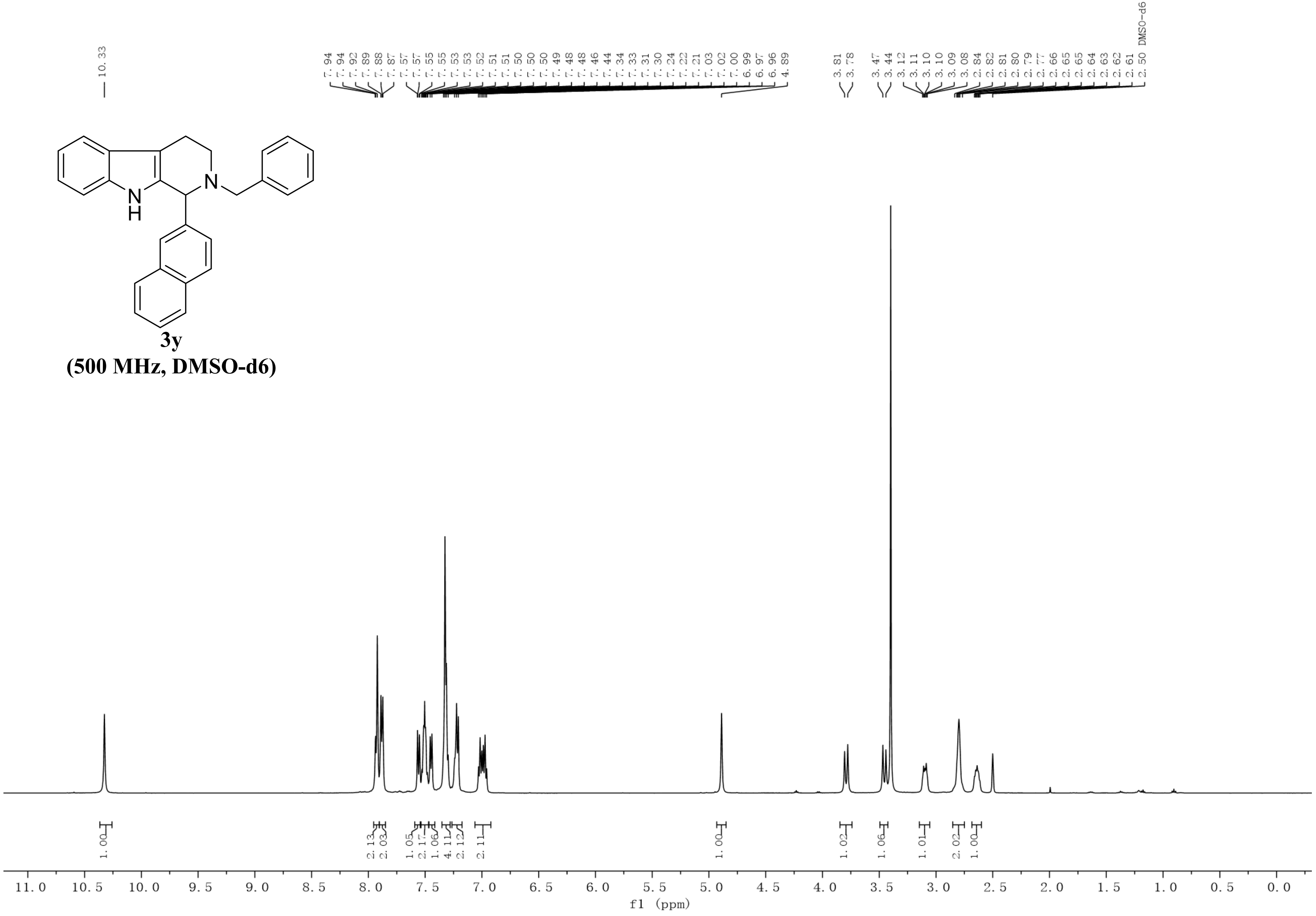
3y
(500 MHz, DMSO-d6)
f1 (ppm)

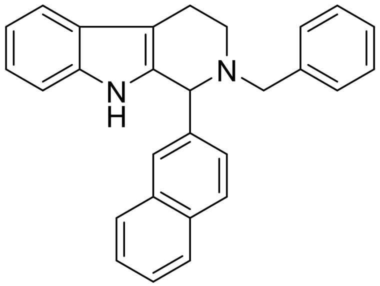

3y
(126 MHz, DMSO-d6)

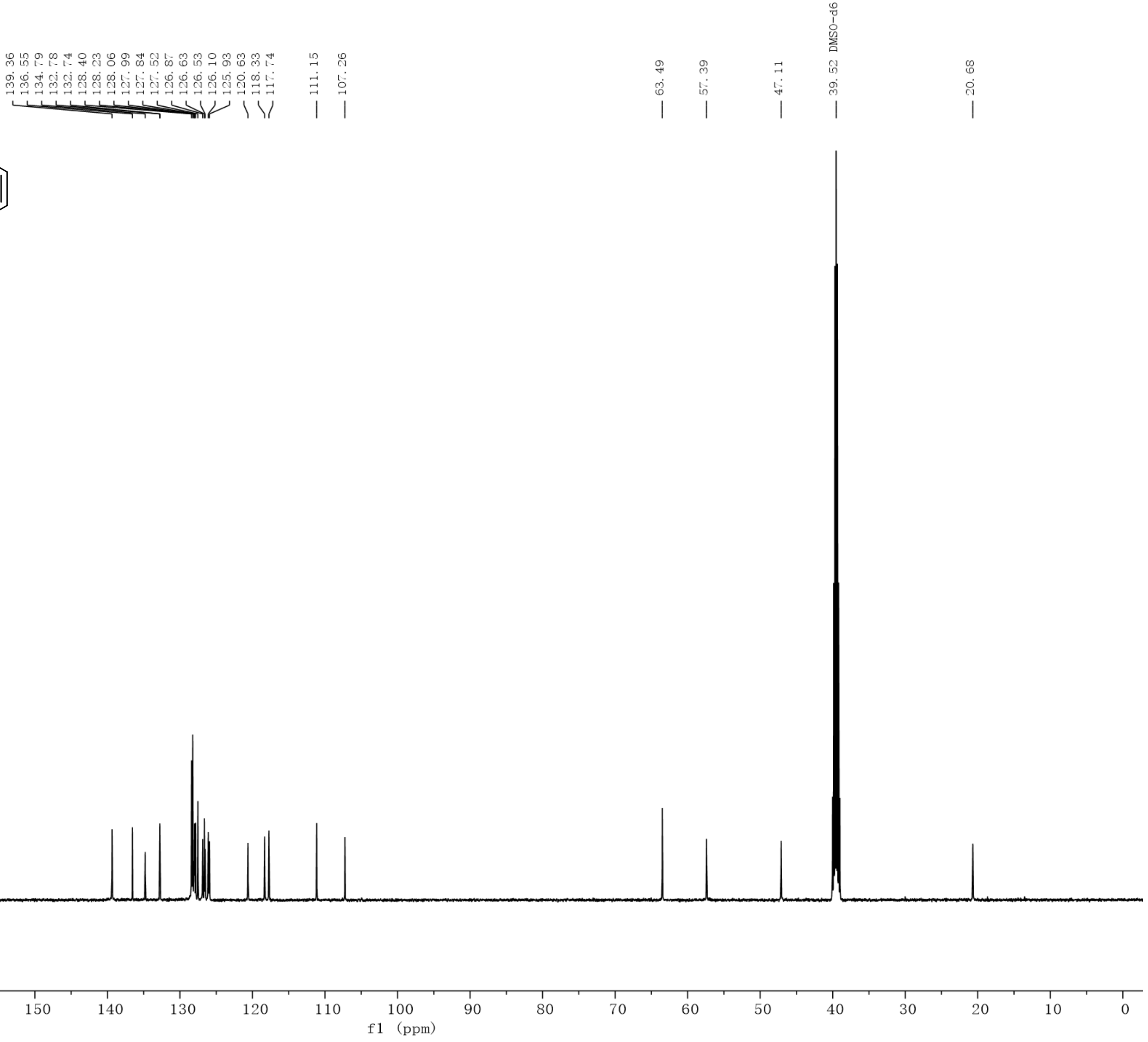

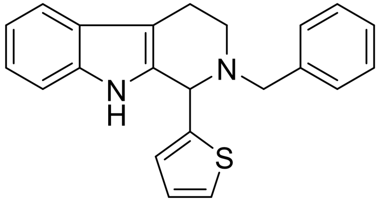

3z
(500 MHz, $CDCl_3$)

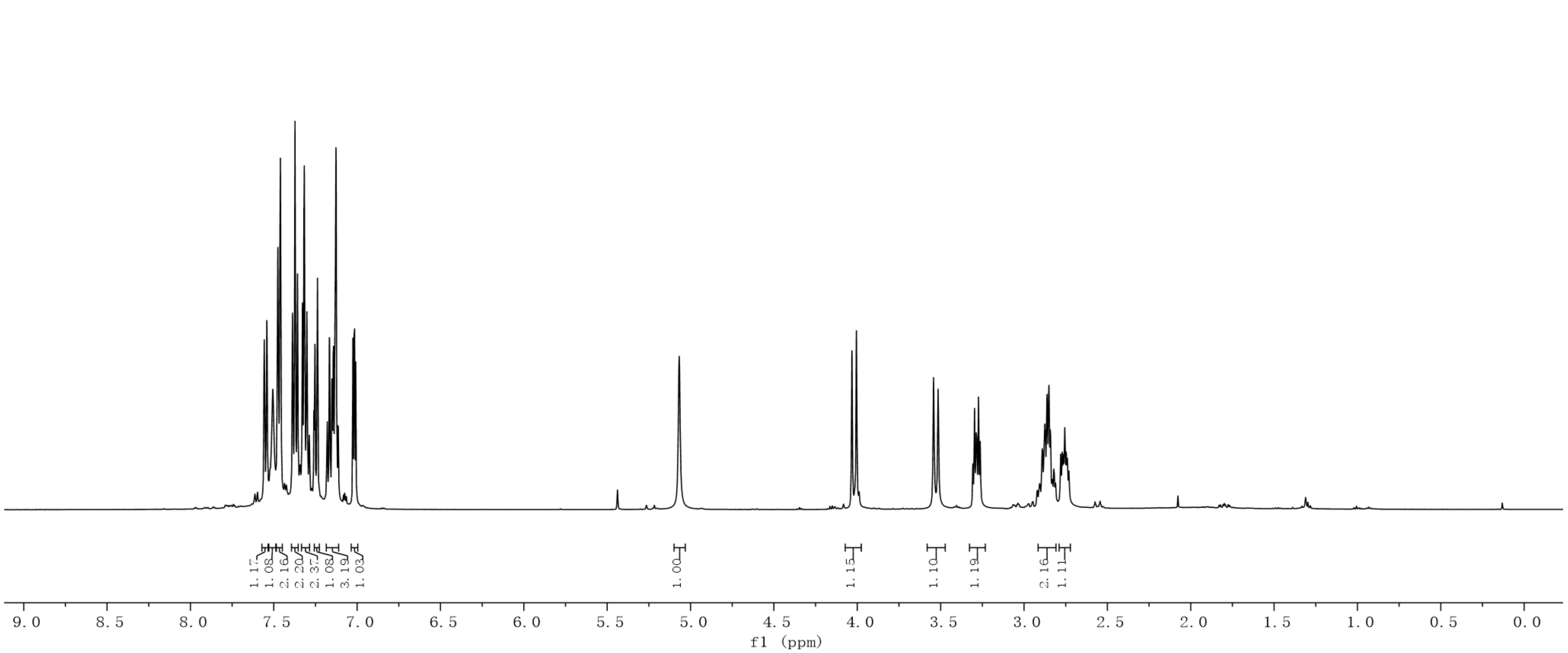

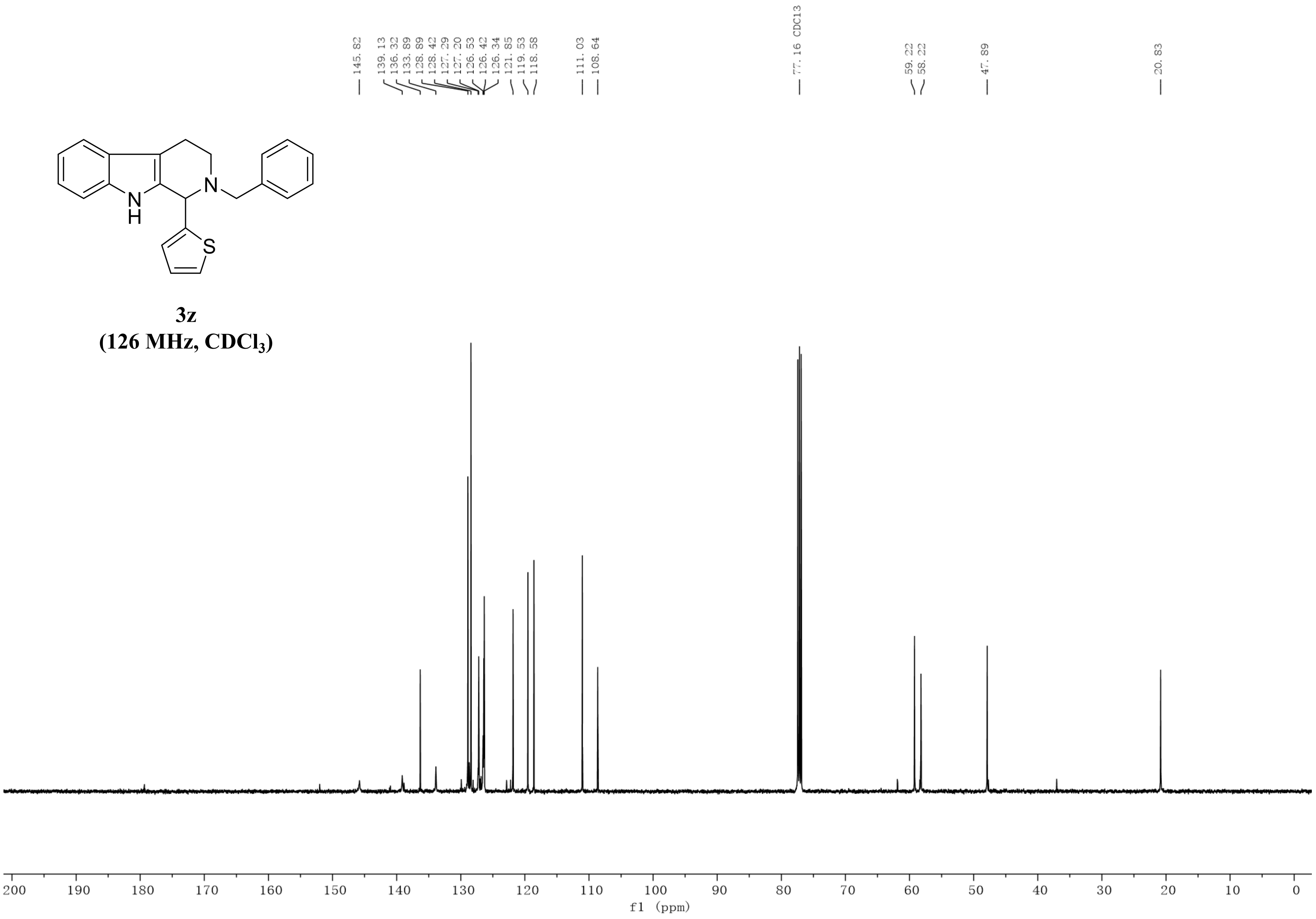

3z
(126 MHz, $CDCl_3$)

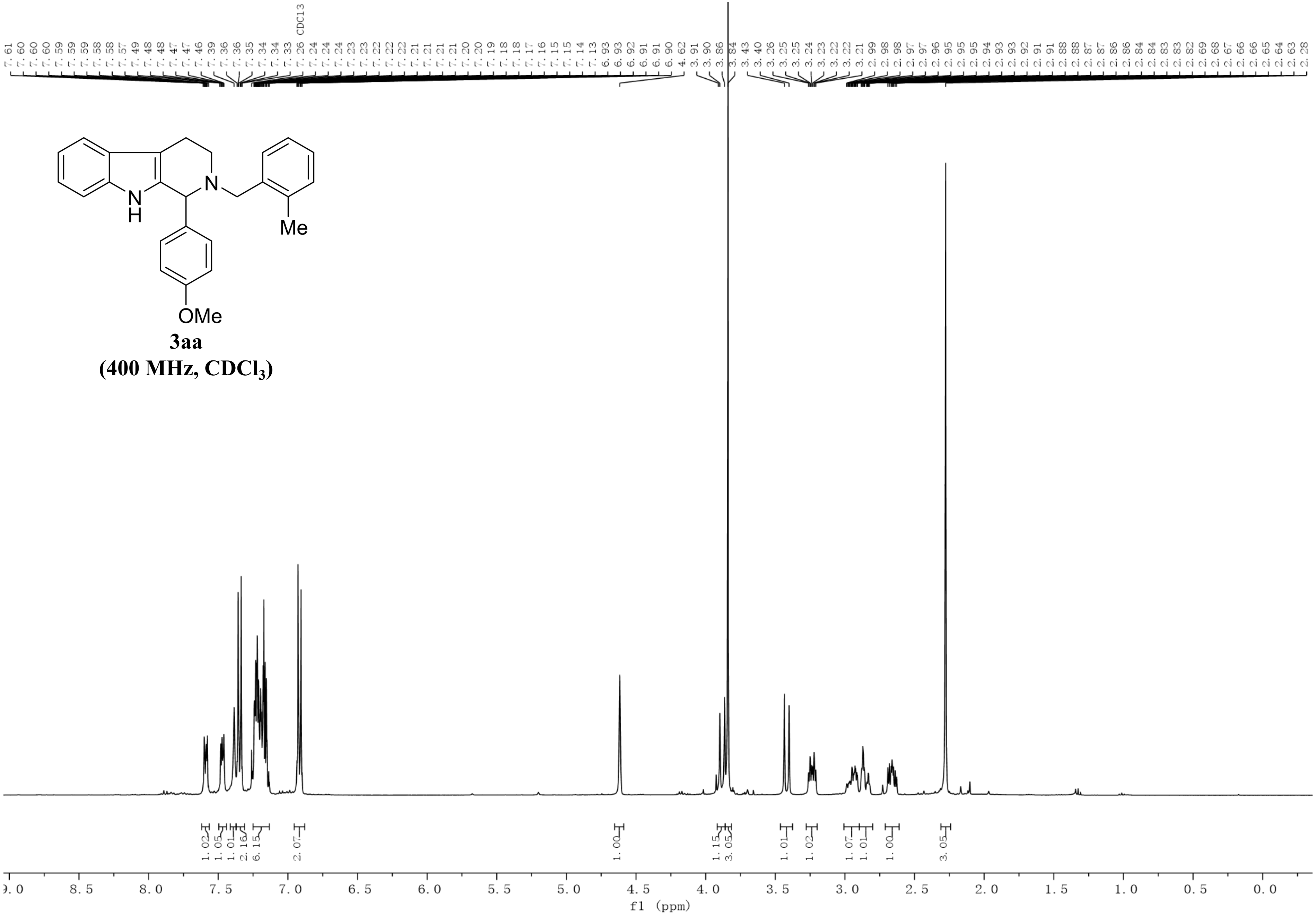

3aa
(400 MHz, $CDCl_3$)

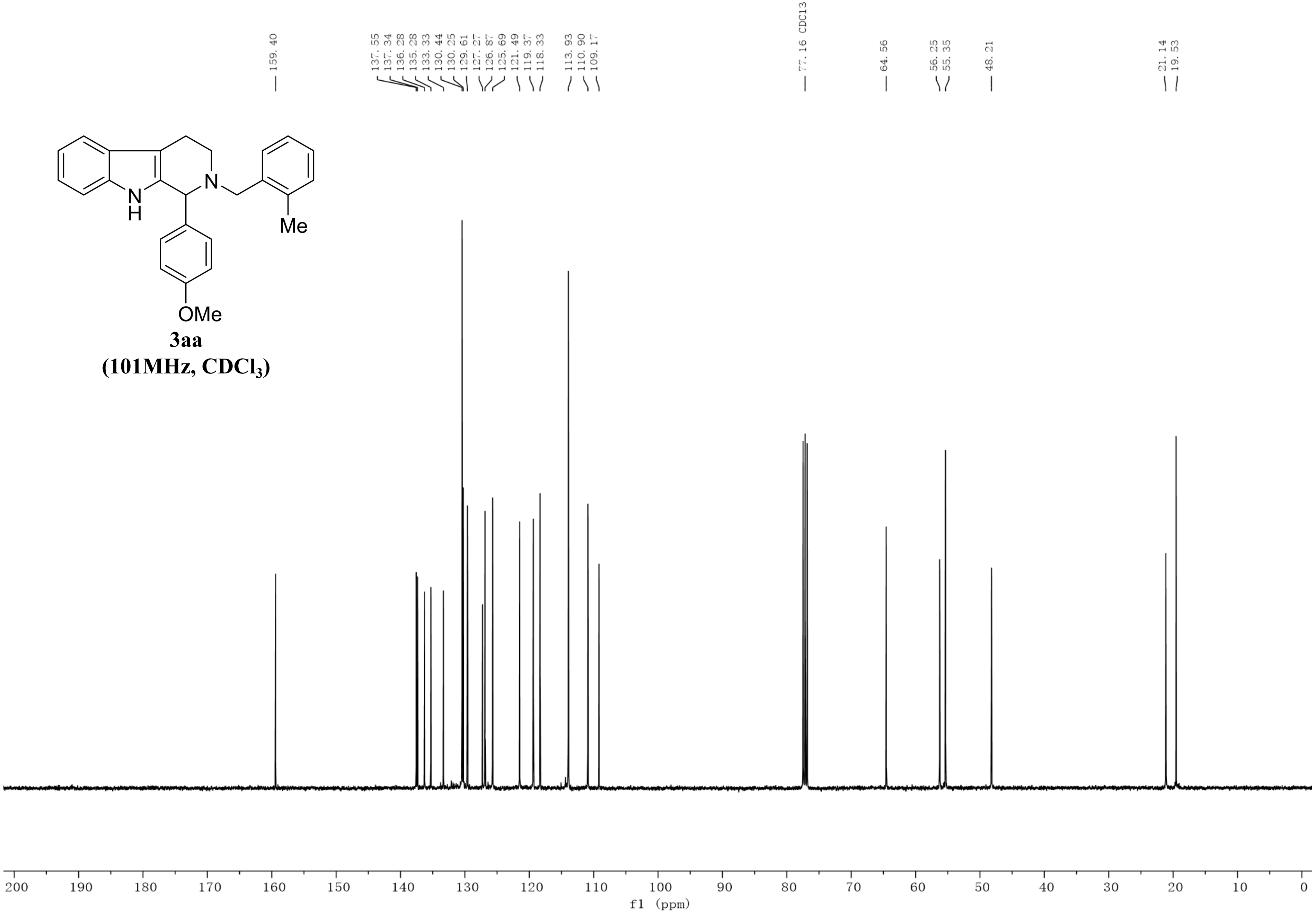

3aa
(101MHz, $CDCl_3$)

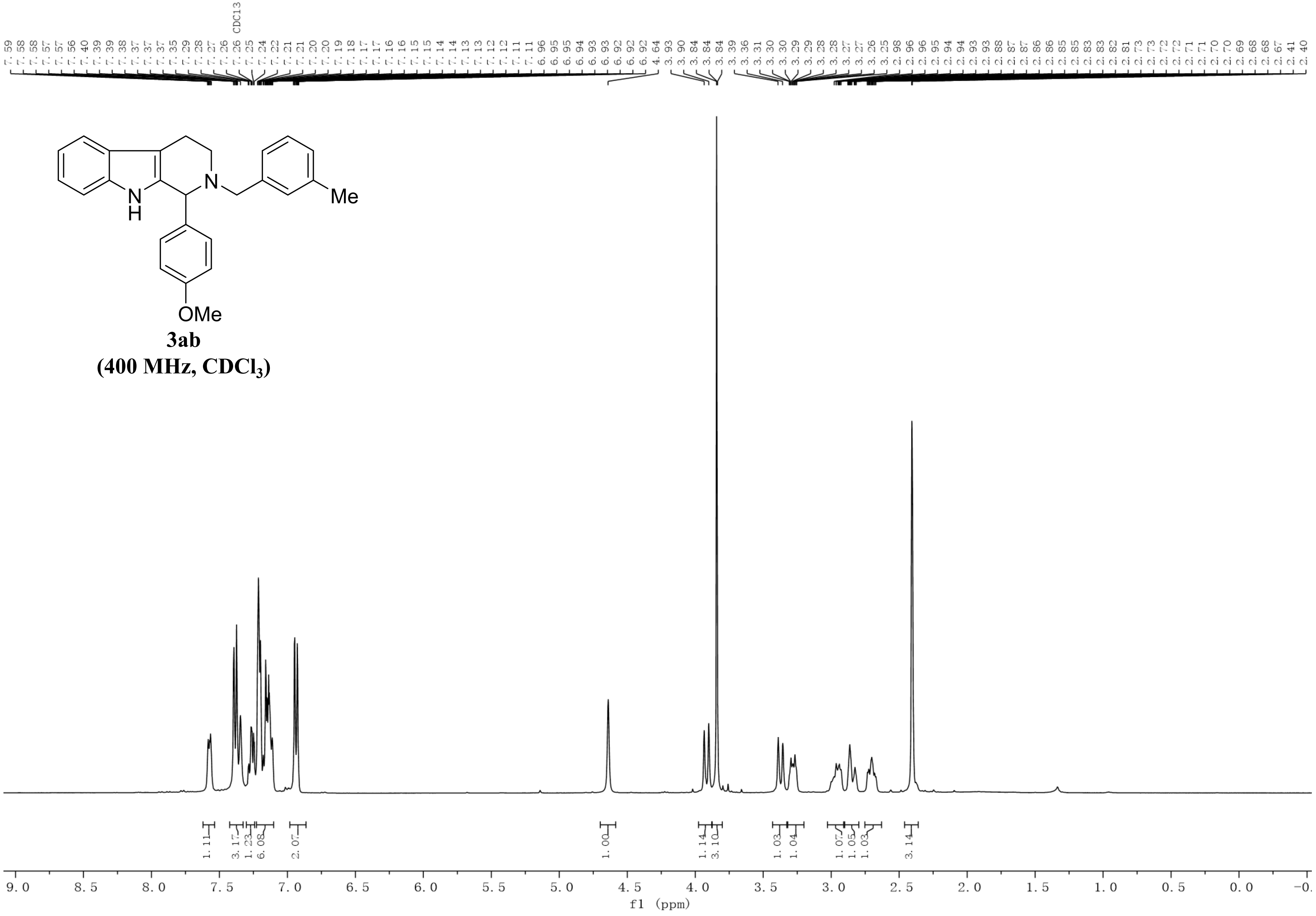
3ab
(400 MHz, CDCl3)
CDCl3
f1 (ppm)

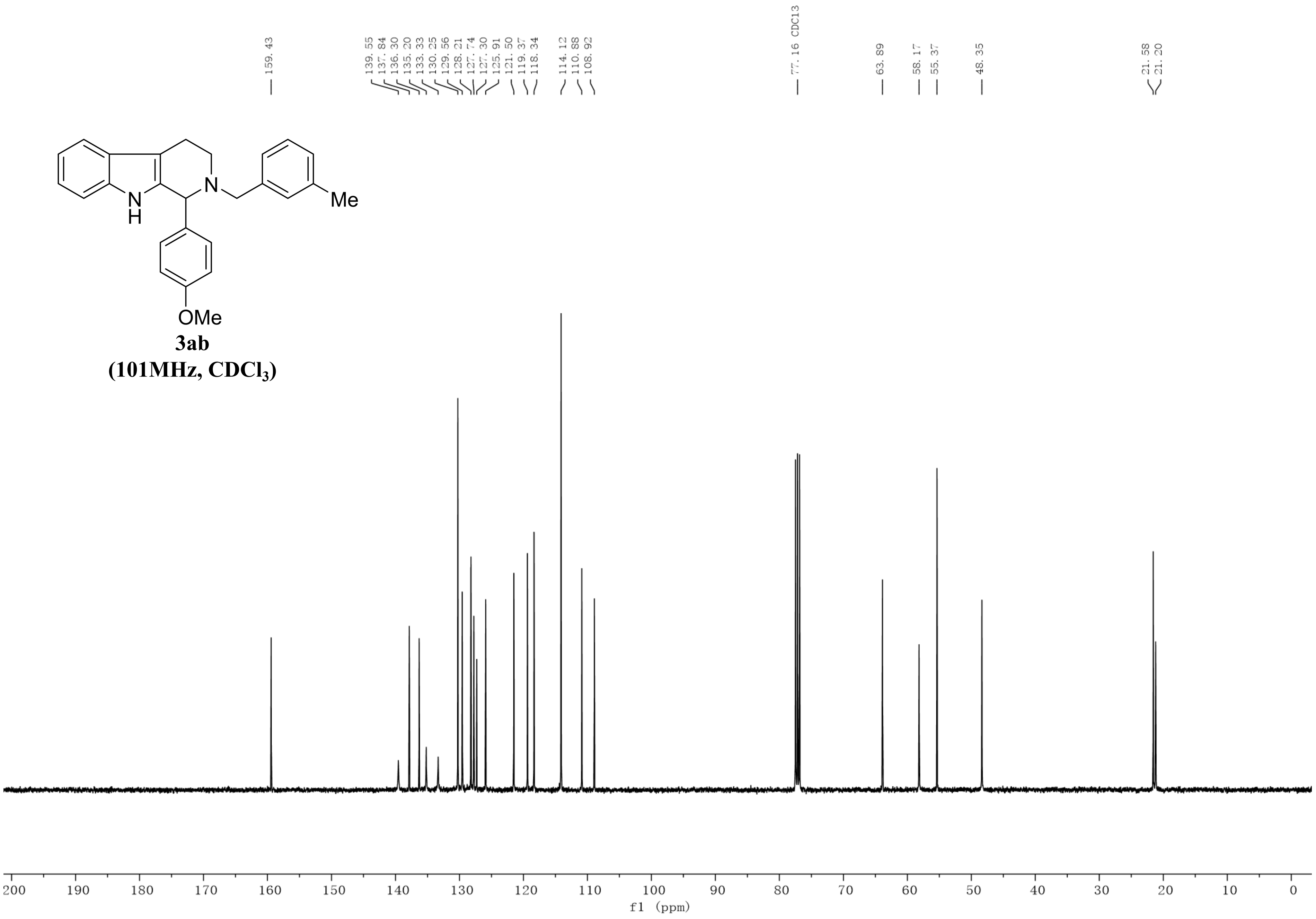

3ab

(101MHz, CDCl$_3$)

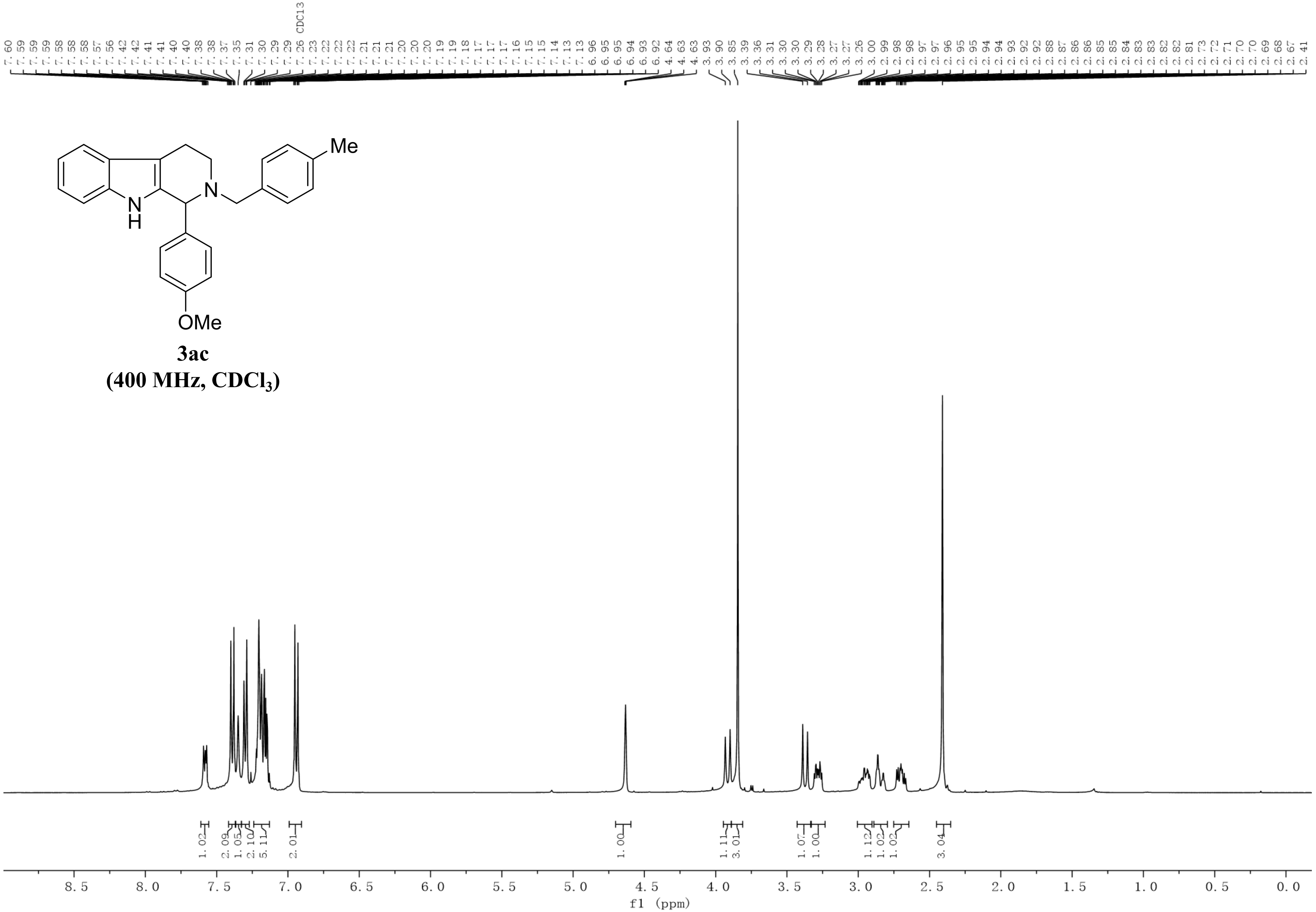

3ac
(400 MHz, $CDCl_3$)

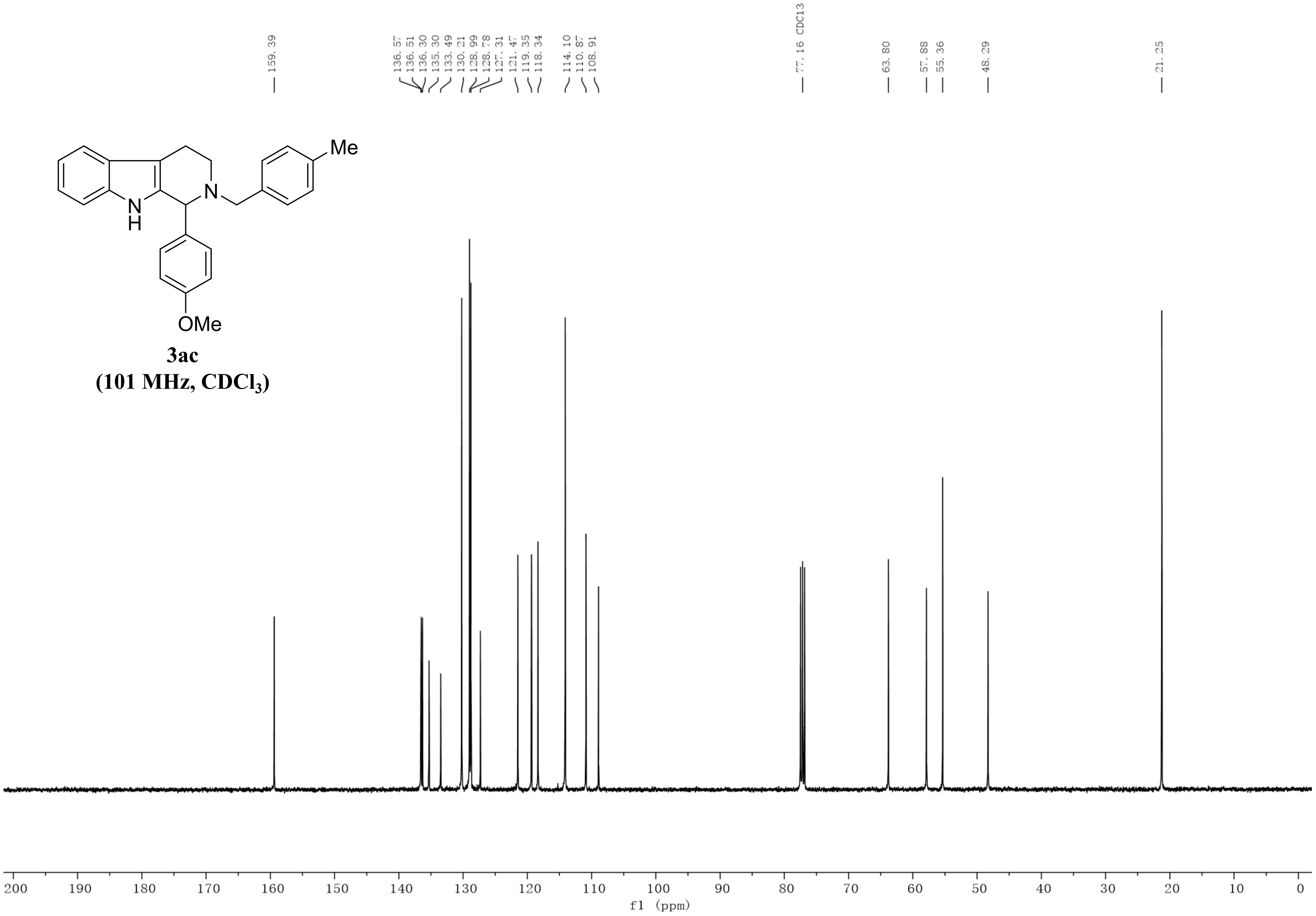

3ac
(101 MHz, $CDCl_3$)

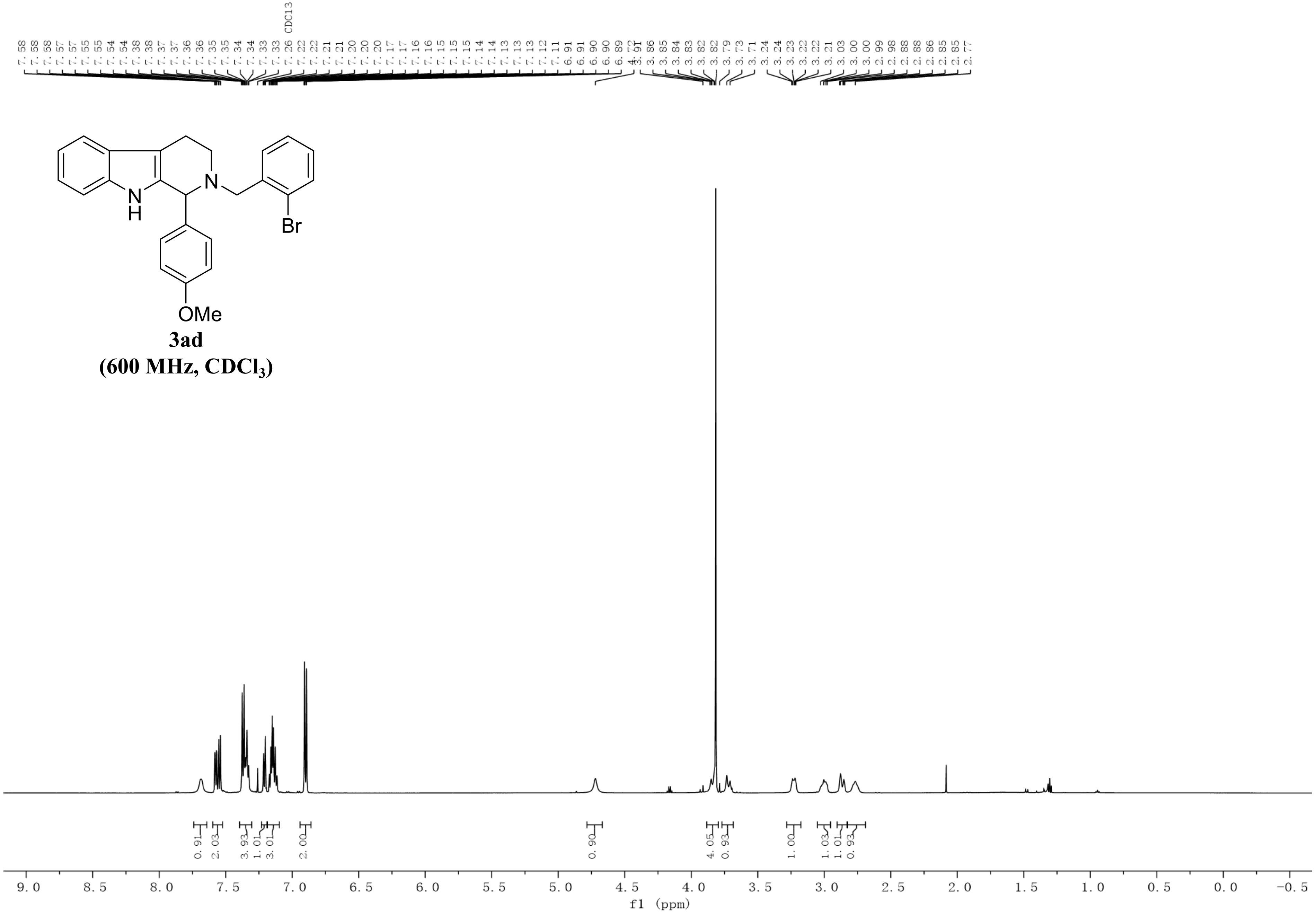

3ad
(600 MHz, $CDCl_3$)

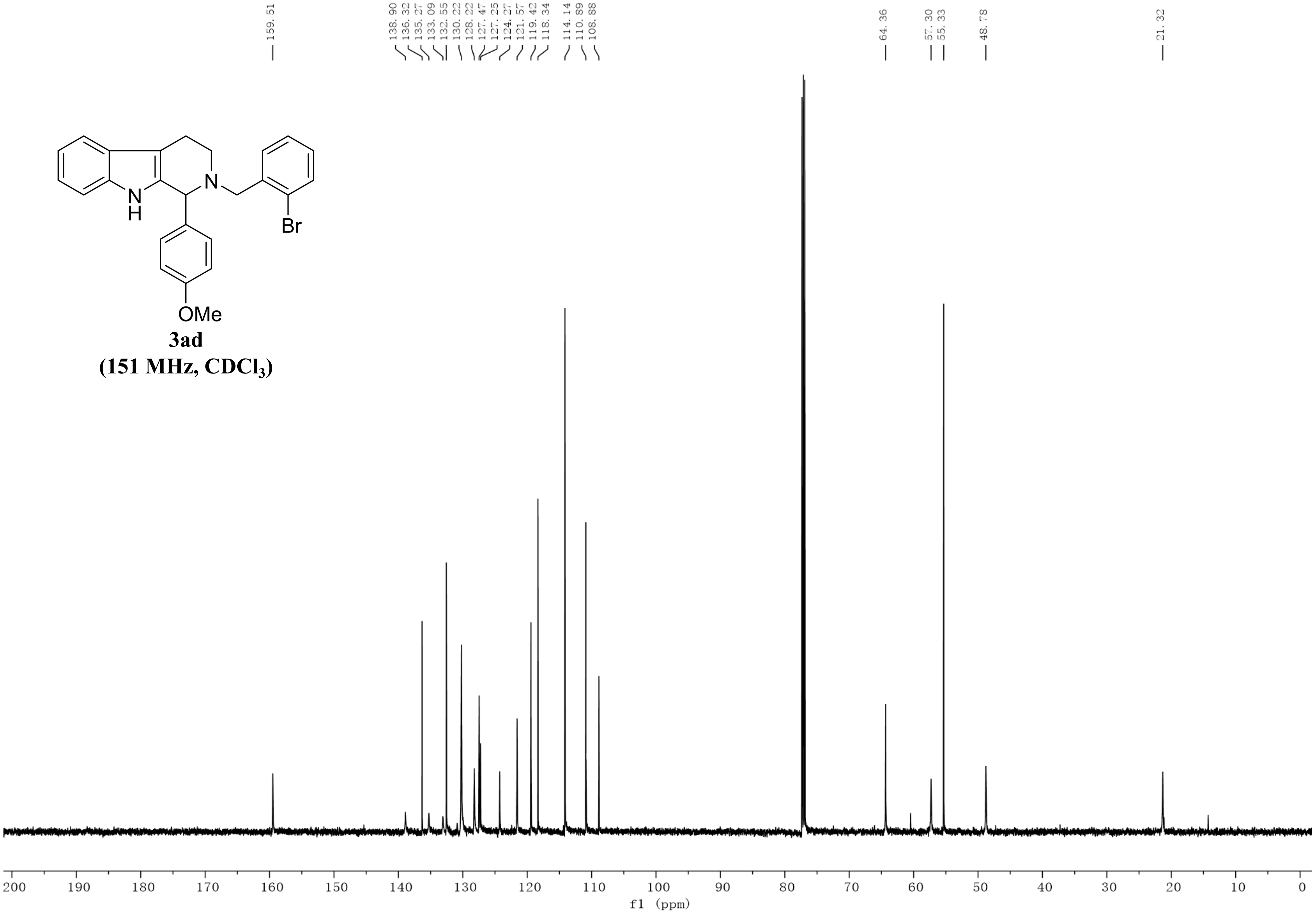

159.51
138.90
136.32
135.27
133.09
132.55
130.22
128.22
127.47
127.25
124.27
121.57
119.42
118.34
114.14
110.89
108.88
64.36
57.30
55.33
48.78
21.32
N
H
N
Br
OMe
3ad
(151 MHz, CDCl3)
200
190
180
170
160
150
140
130
120
110
100
90
80
70
60
50
40
30
20
10
0
f1 (ppm)

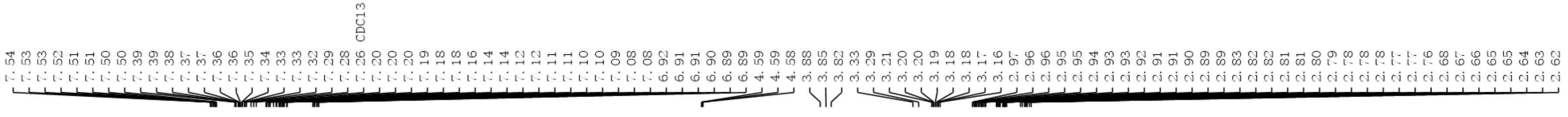

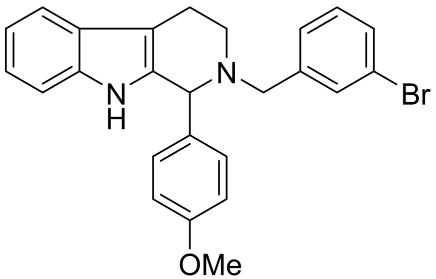

3ae
(400 MHz, $CDCl_3$)

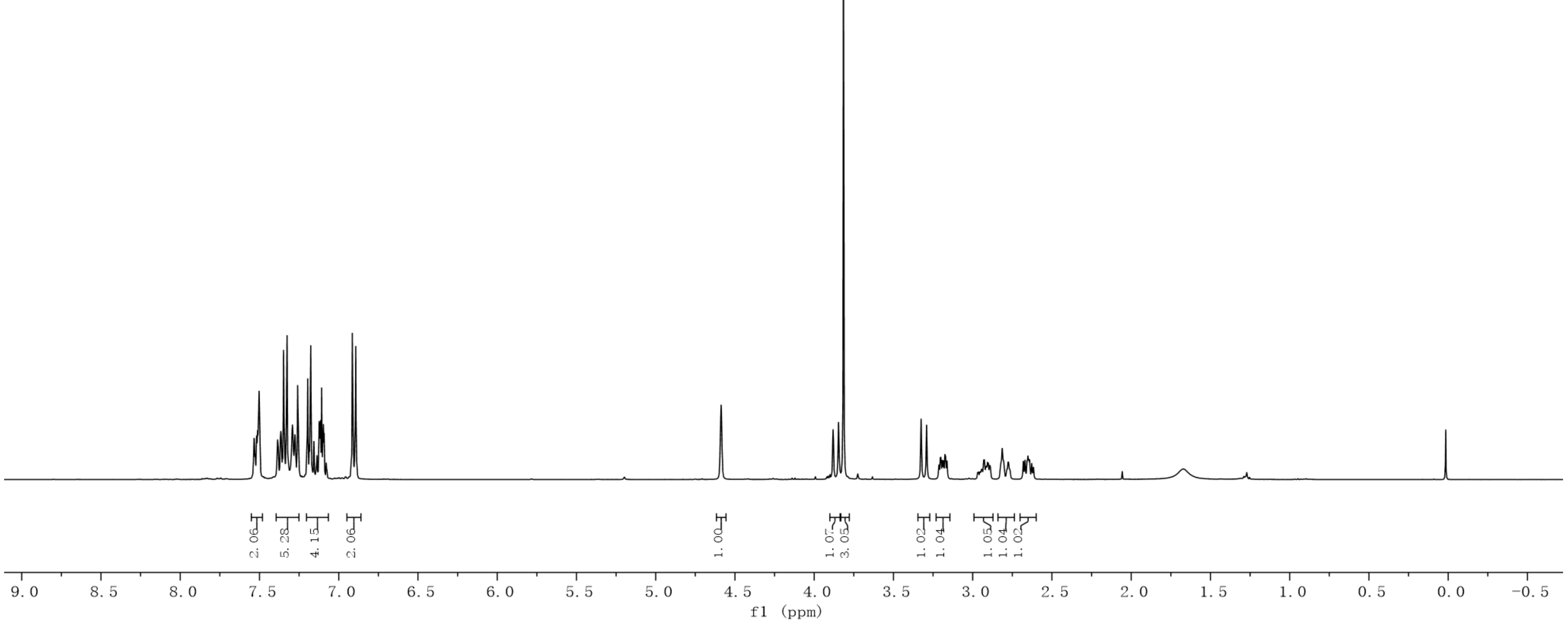

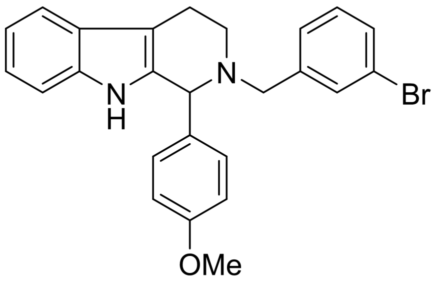

3ae
(101 MHz, $CDCl_3$)

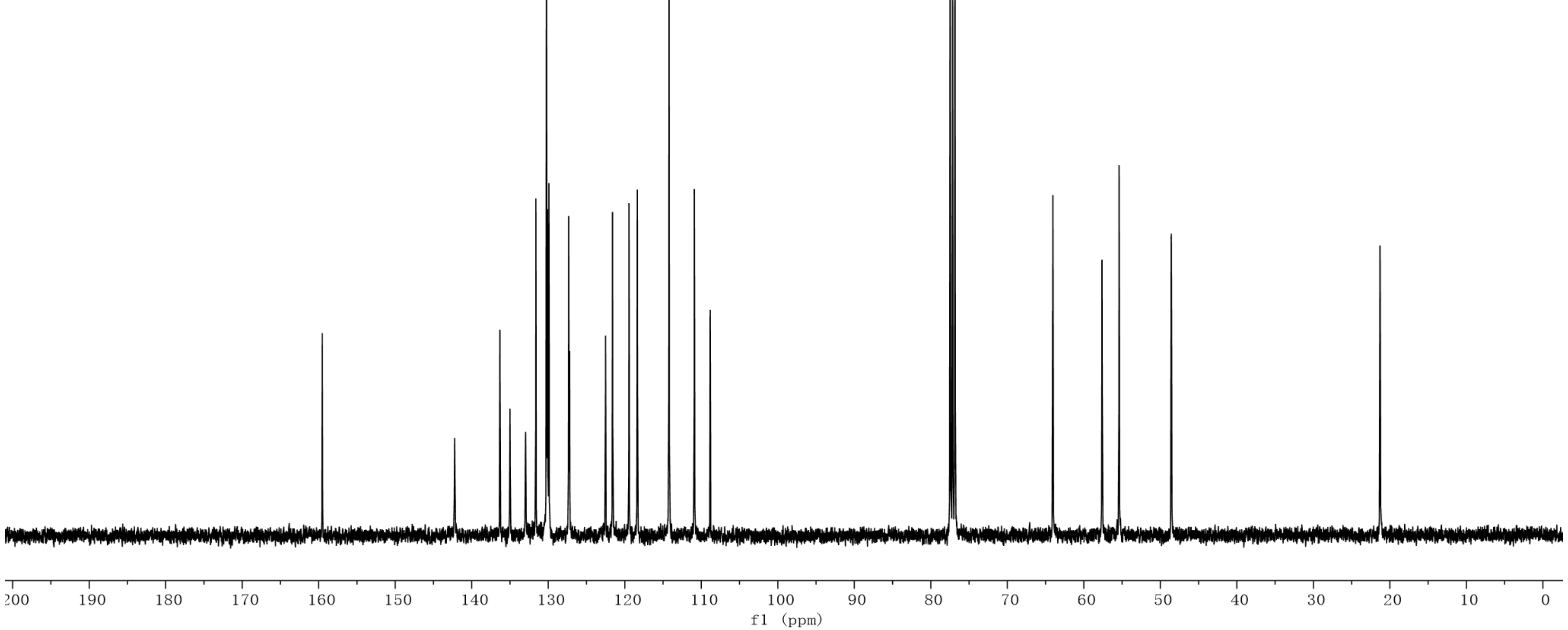

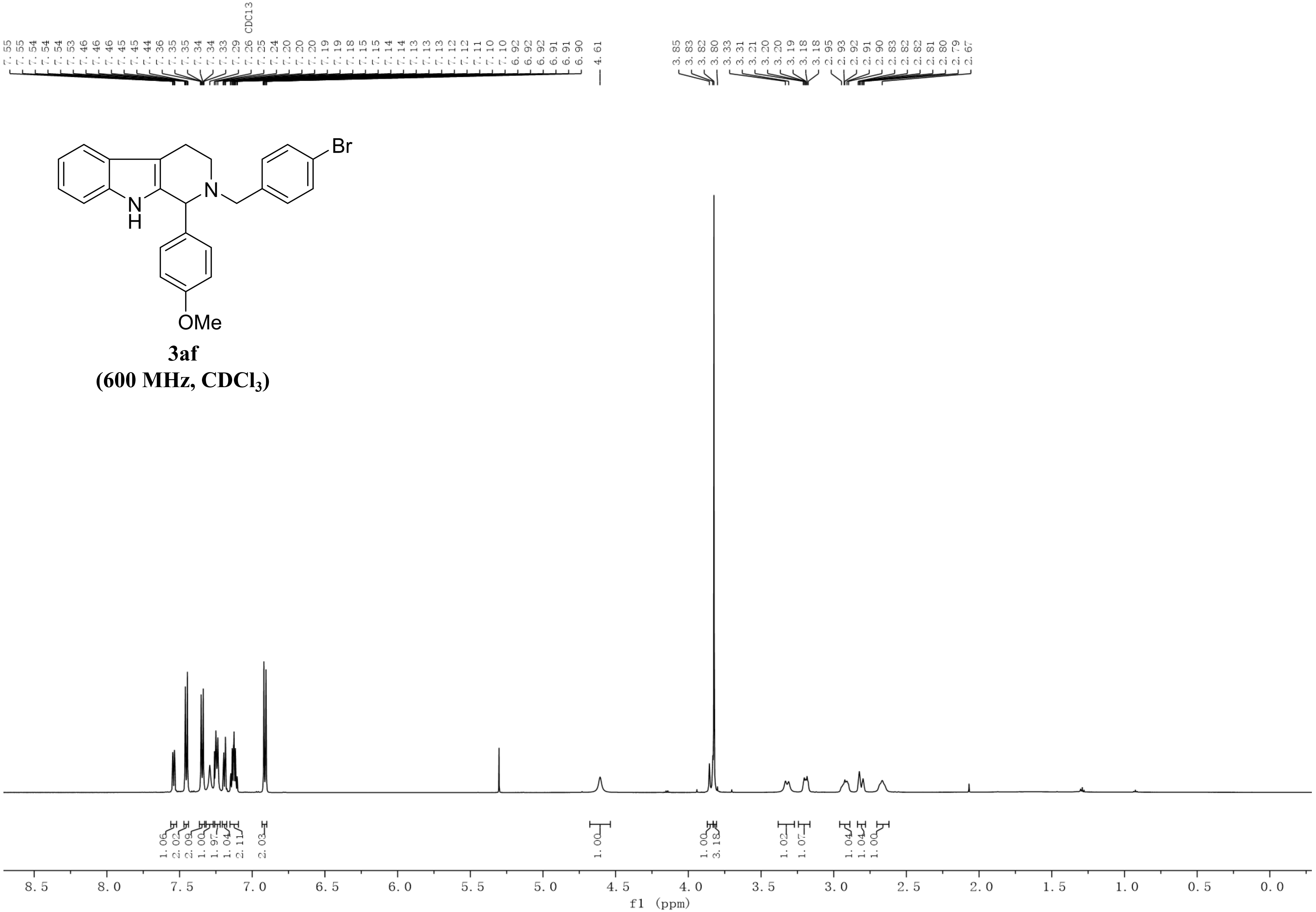

3af
(600 MHz, $CDCl_3$)

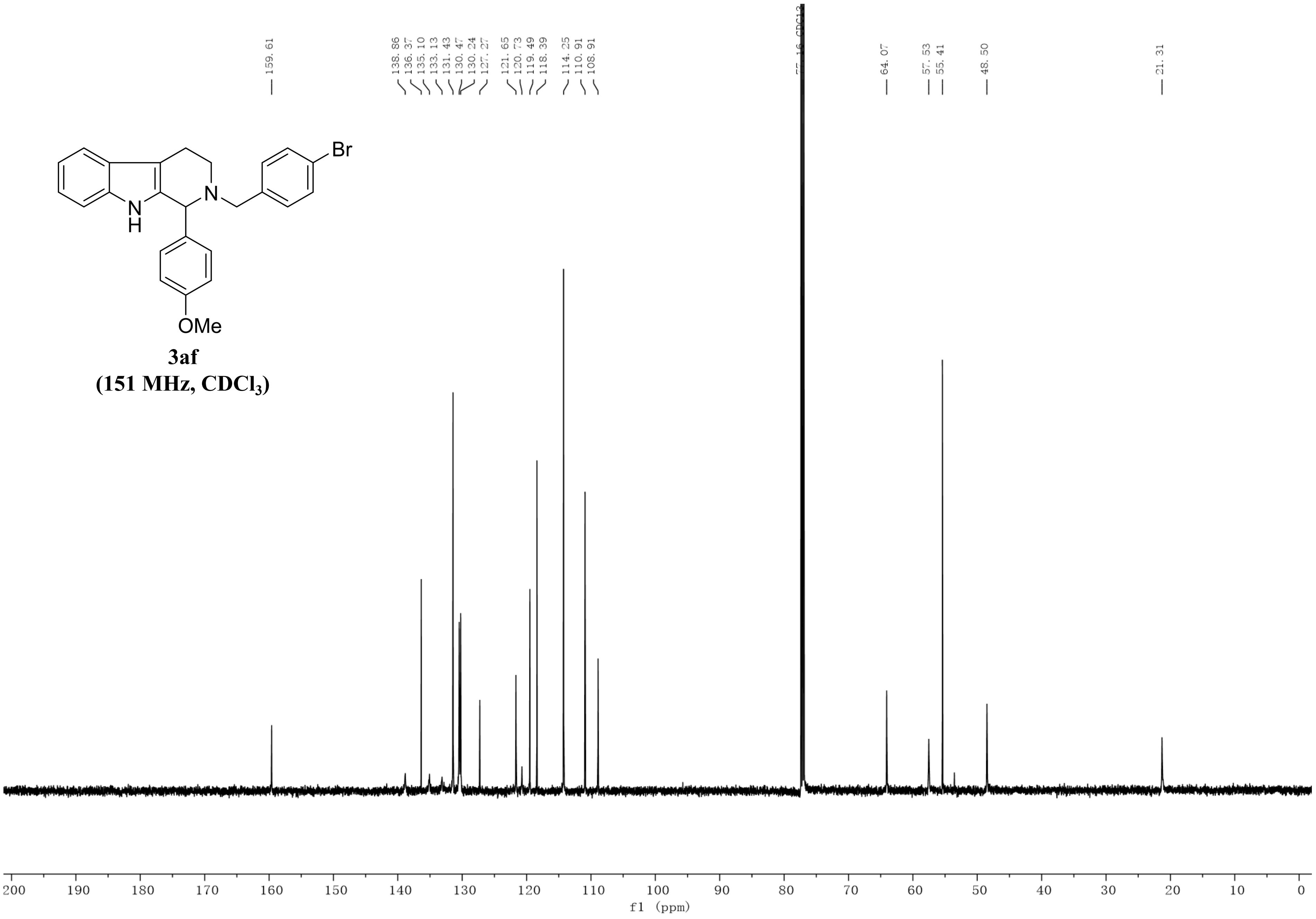

3af
(151 MHz, $CDCl_3$)

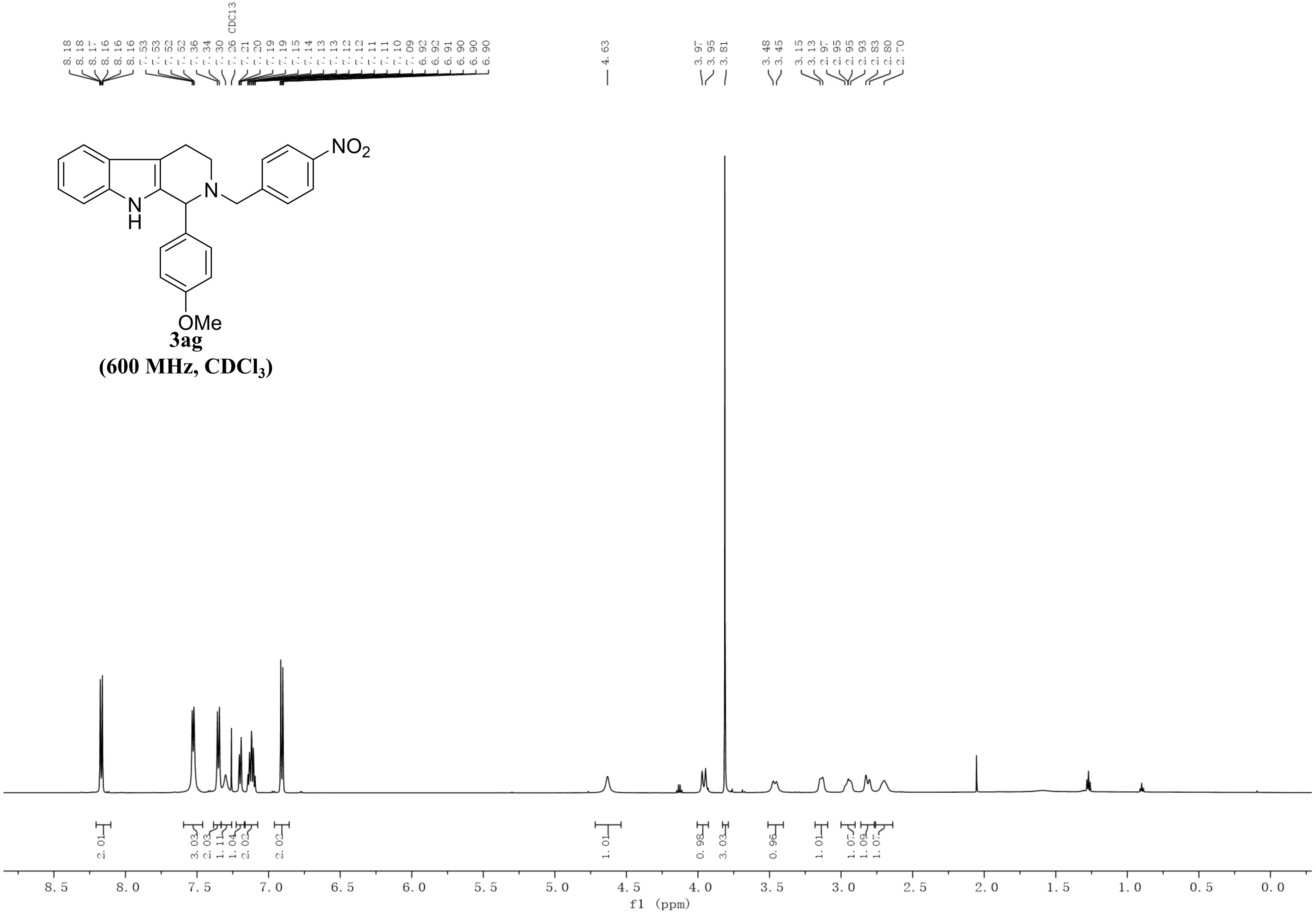
NO2
N
N
H
OMe
3ag
(600 MHz, CDCl3)
f1 (ppm)

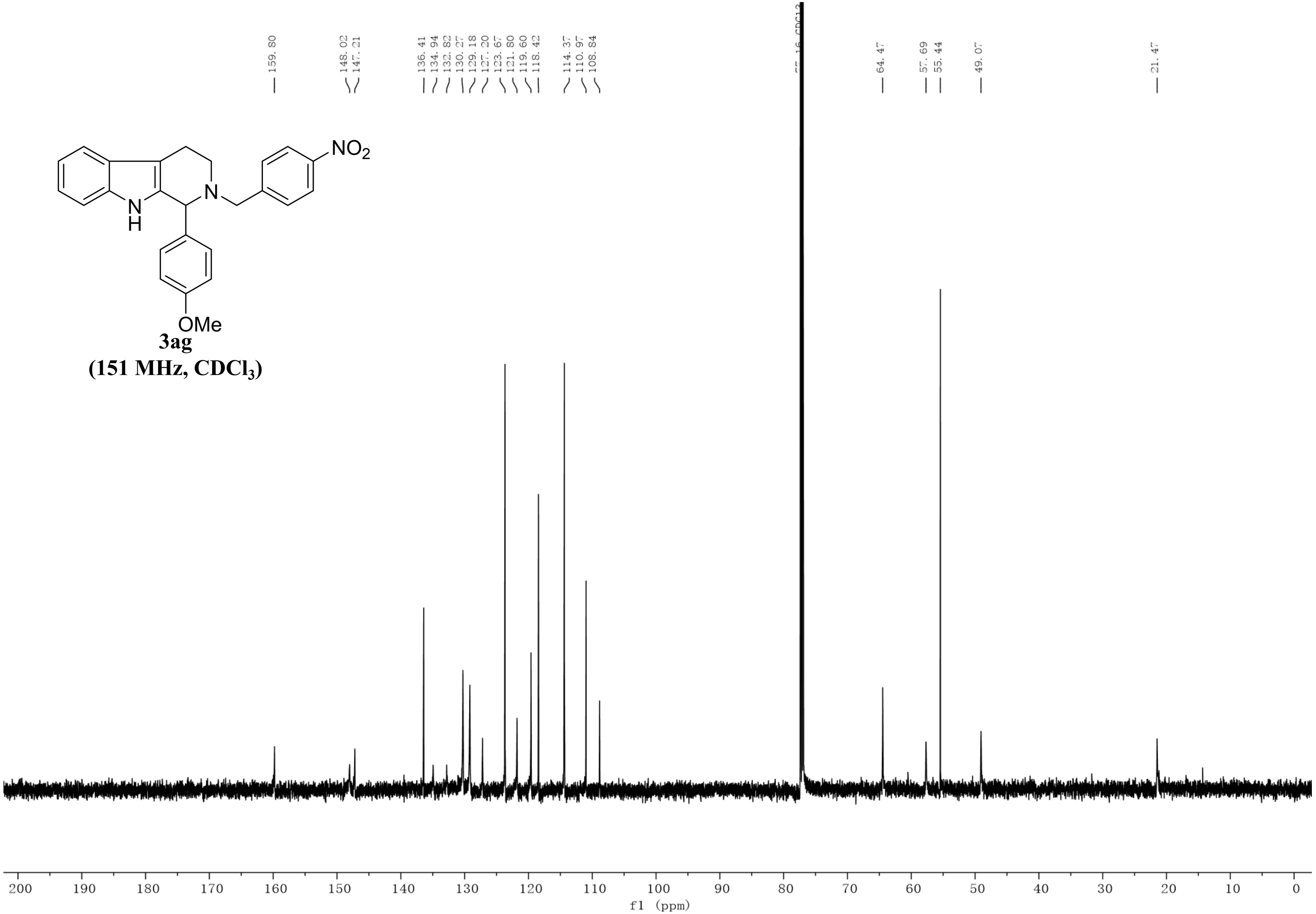

3ag
(151 MHz, $CDCl_3$)

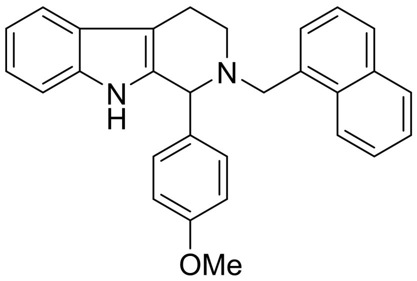

3ah
(400 MHz, $CDCl_3$)

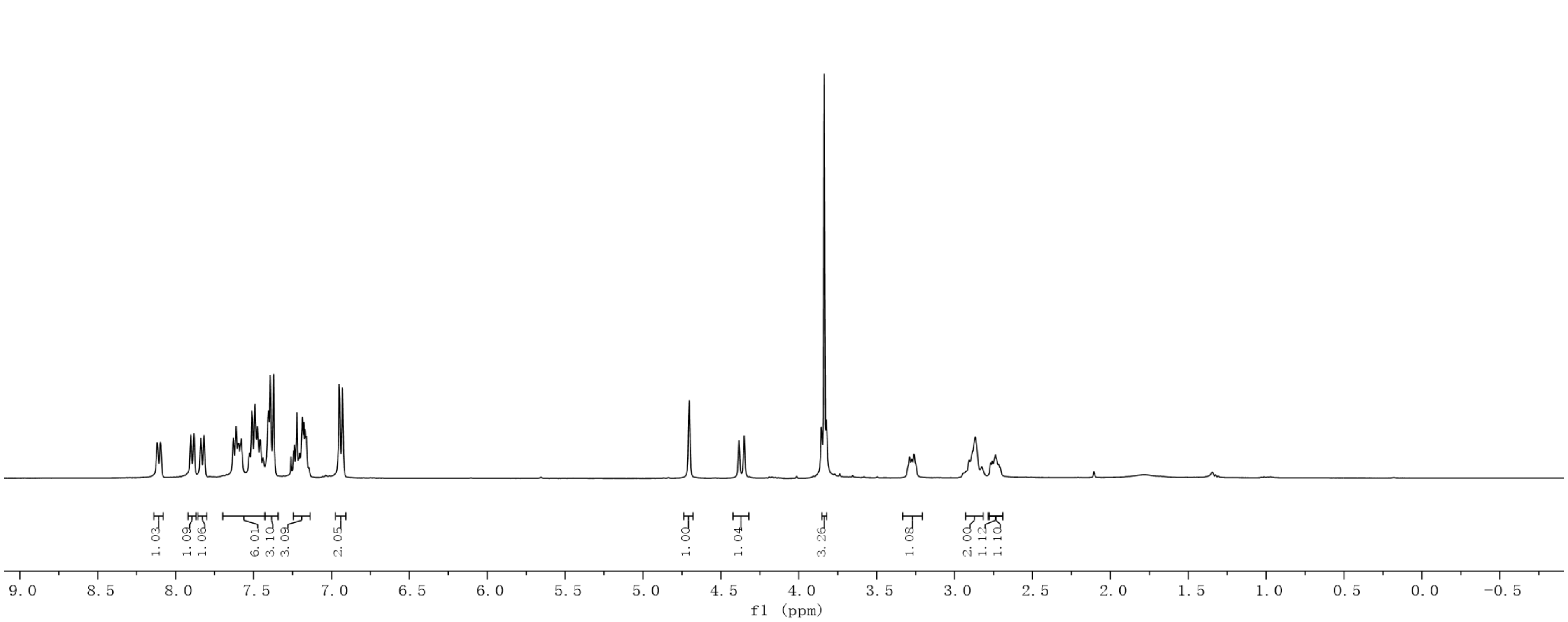

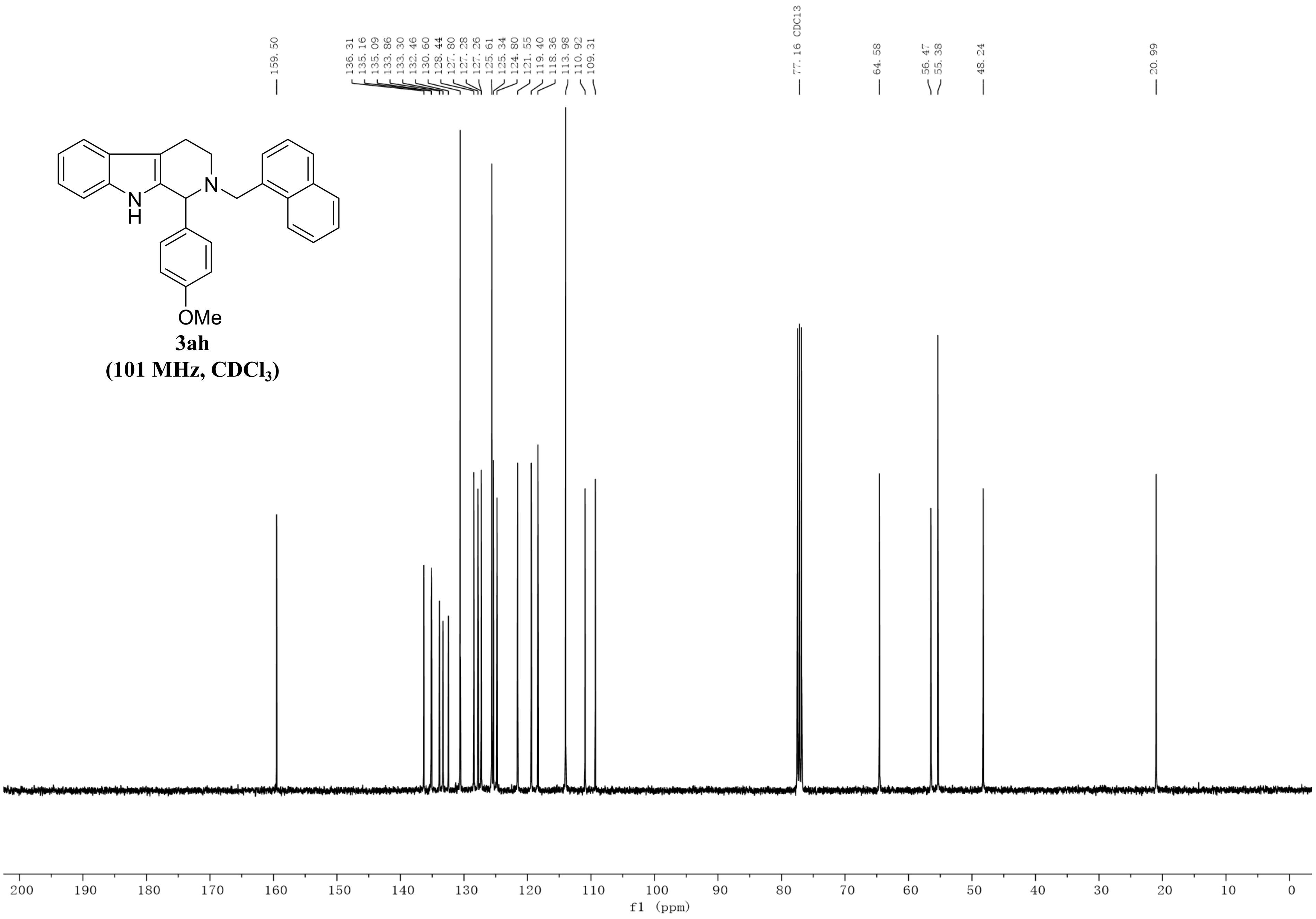
159.50
136.31
135.16
135.09
133.86
133.30
132.46
130.60
128.44
127.80
127.28
127.26
125.61
125.34
124.80
121.55
119.40
118.36
113.98
110.92
109.31
77.16 CDCl3
64.58
56.47
55.38
48.24
20.99
OMe
N
N
H
3ah
(101 MHz, CDCl3)
200 190 180 170 160 150 140 130 120 110 100 90 80 70 60 50 40 30 20 10 0
f1 (ppm)

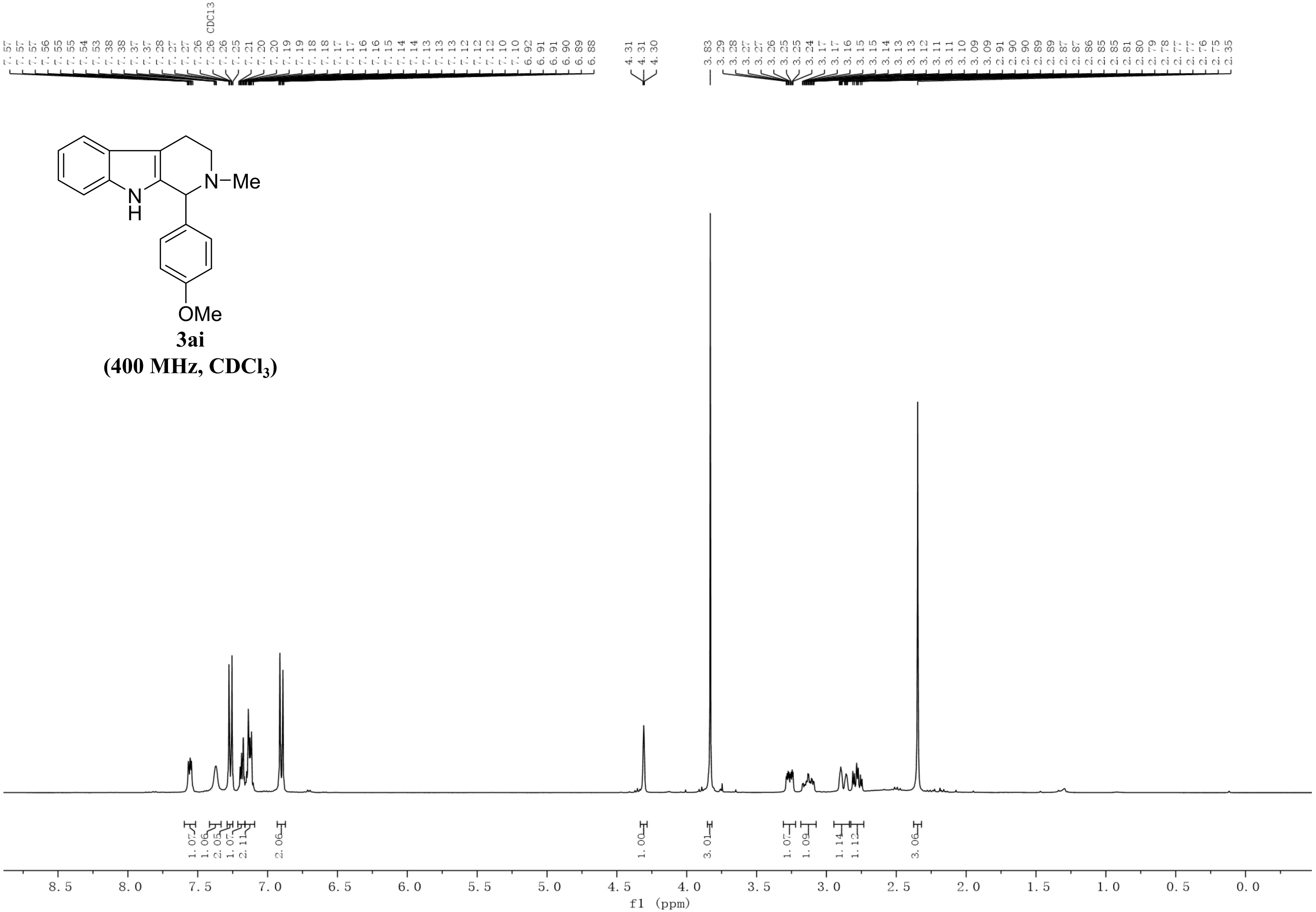
N−Me
N
H
OMe
3ai
(400 MHz, CDCl$_3$)
f1 (ppm)

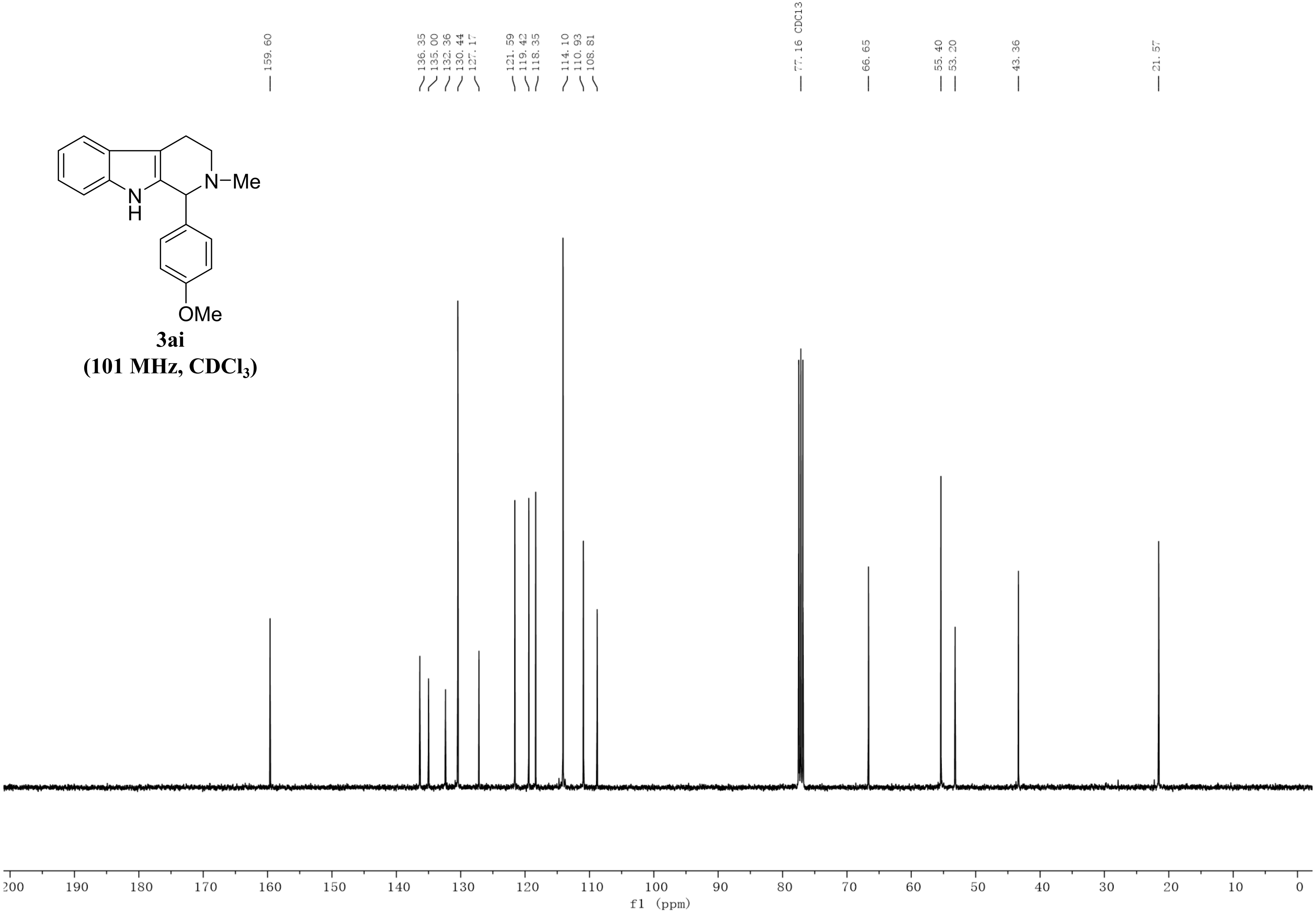

3ai
(101 MHz, $CDCl_3$)

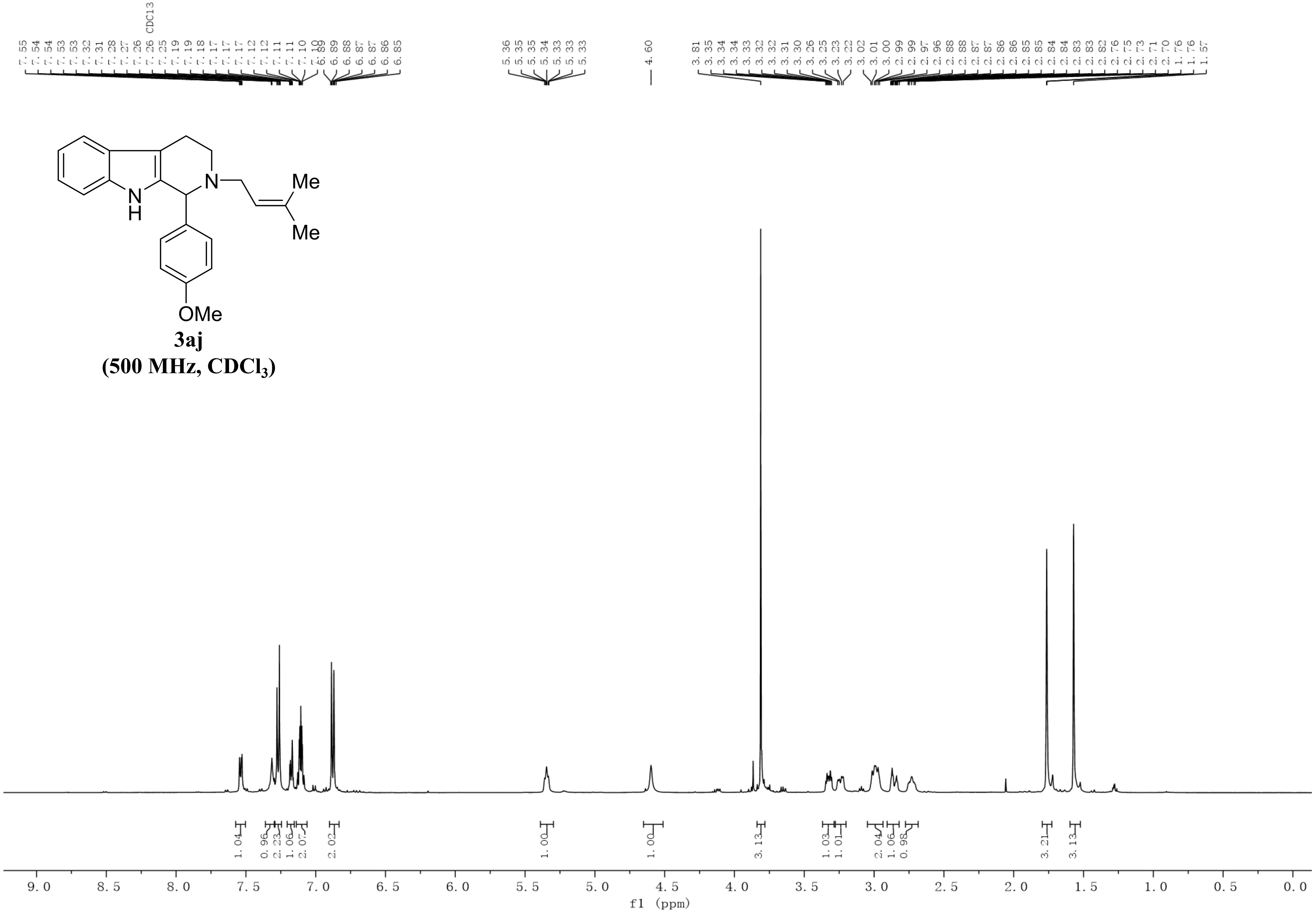

3aj
(500 MHz, $CDCl_3$)

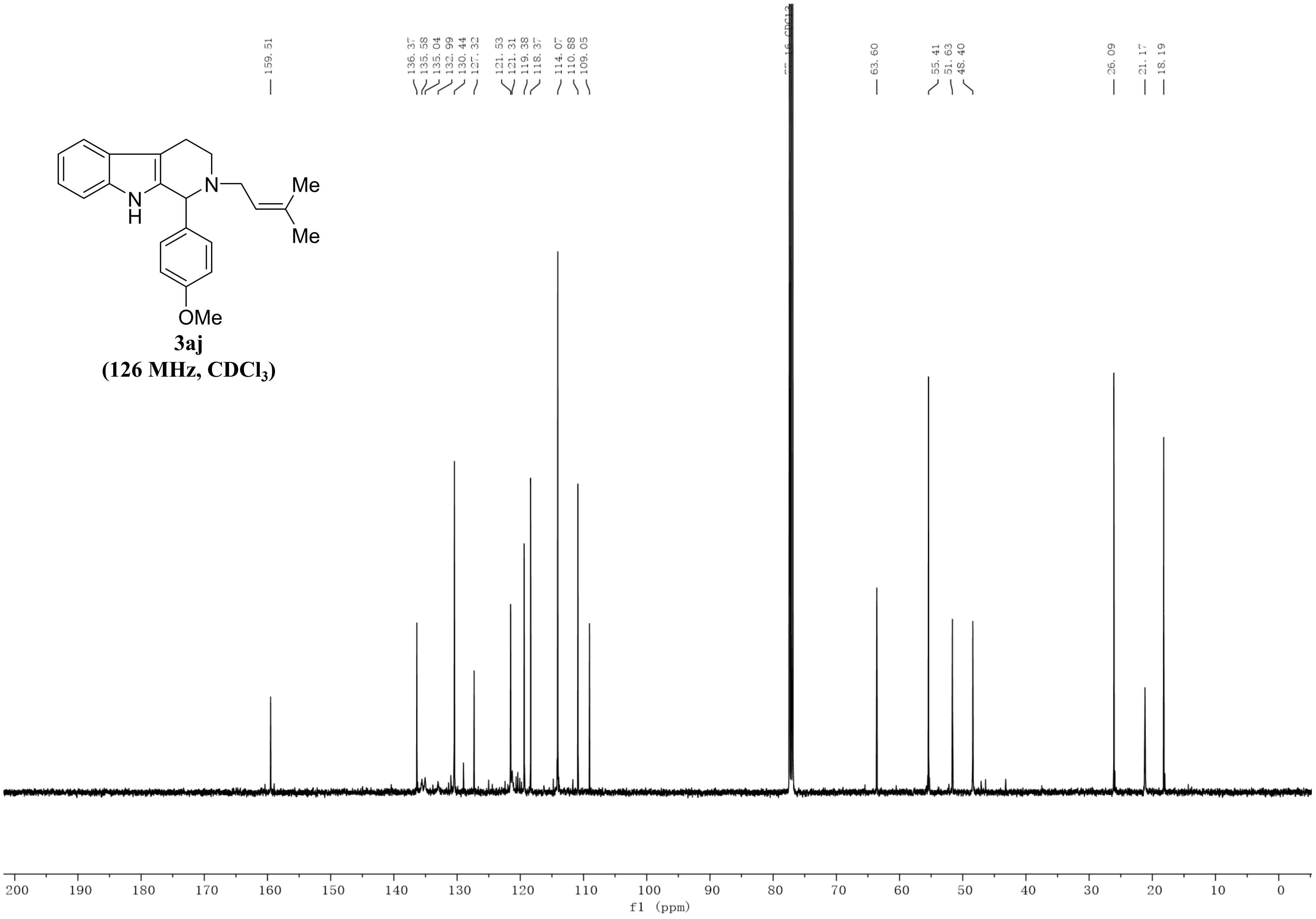

3aj
(126 MHz, $CDCl_3$)

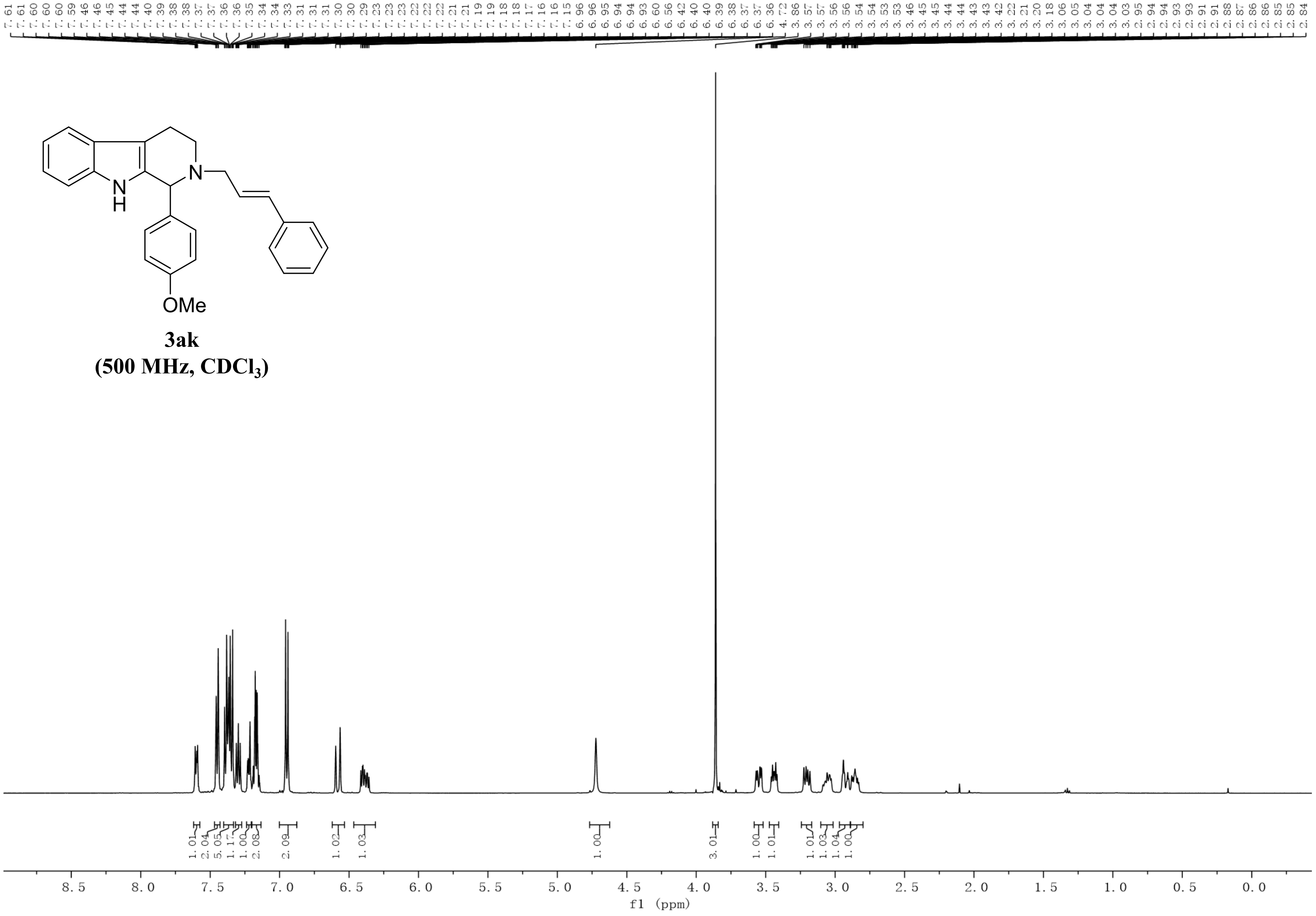
7.61 7.61 7.60 7.60 7.59 7.46 7.46 7.45 7.44 7.44 7.40 7.39 7.38 7.38 7.37 7.37 7.36 7.36 7.35 7.34 7.34 7.33 7.31 7.31 7.31 7.30 7.30 7.29 7.23 7.23 7.23 7.22 7.22 7.22 7.21 7.21 7.19 7.19 7.18 7.18 7.17 7.16 7.16 7.15 6.96 6.95 6.94 6.94 6.93 6.60 6.56 6.42 6.40 6.40 6.39 6.38 6.37 6.37 6.36 4.72 3.86 3.57 3.57 3.56 3.54 3.54 3.53 3.53 3.46 3.45 3.45 3.44 3.43 3.43 3.42 3.22 3.21 3.20 3.18 3.06 3.05 3.04 3.04 3.04 3.03 2.95 2.94 2.94 2.93 2.93 2.91 2.91 2.88 2.87 2.86 2.86 2.85 2.85 2.84
N
H
N
OMe
3ak
(500 MHz, $CDCl_3$)
1.01 2.04 5.05 1.17 1.00 2.08 2.09 1.02 1.03 1.00 3.01 1.00 1.01 1.01 1.03 1.04 1.00
8.5 8.0 7.5 7.0 6.5 6.0 5.5 5.0 4.5 4.0 3.5 3.0 2.5 2.0 1.5 1.0 0.5 0.0
f1 (ppm)

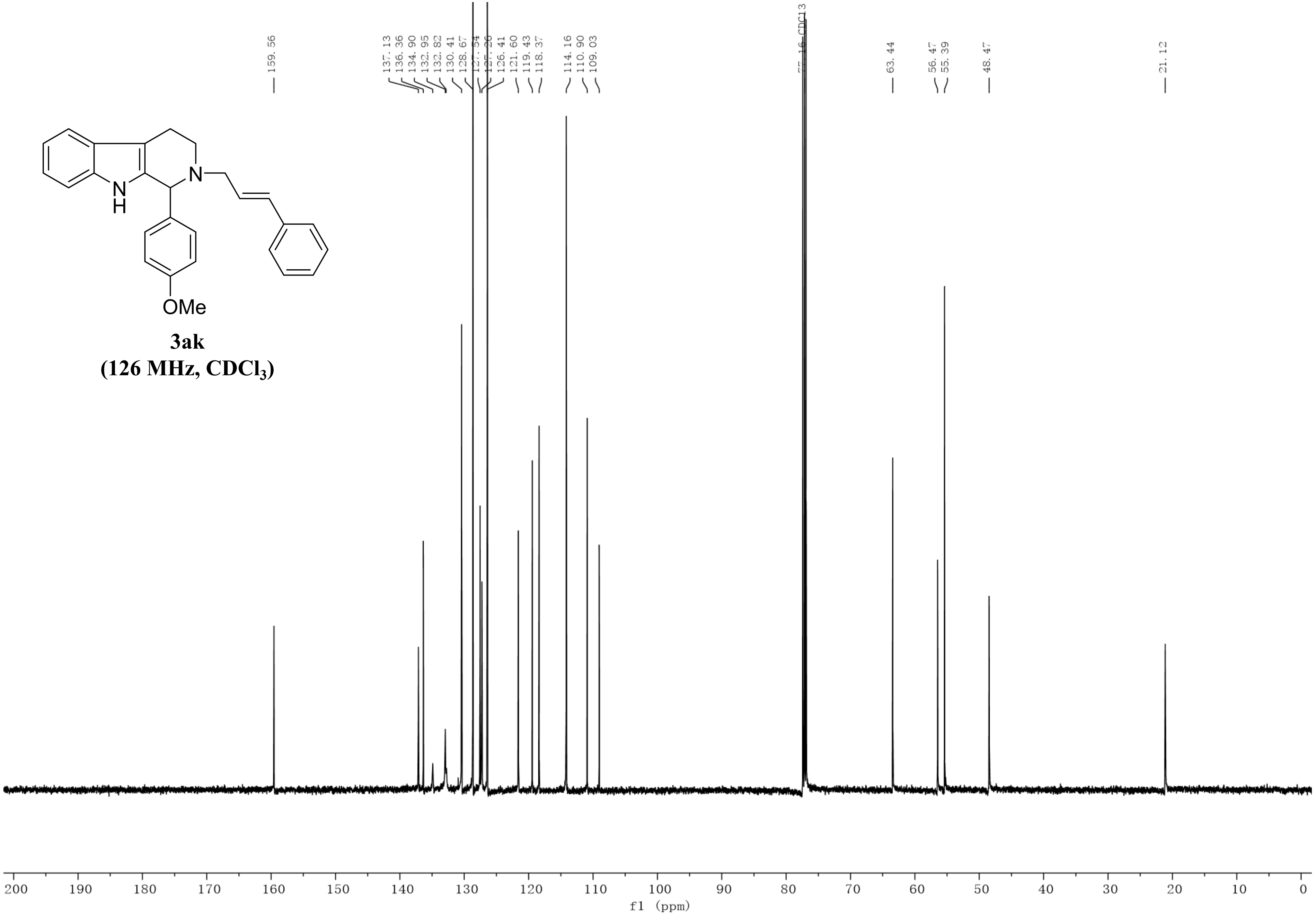

3ak
(126 MHz, $CDCl_3$)

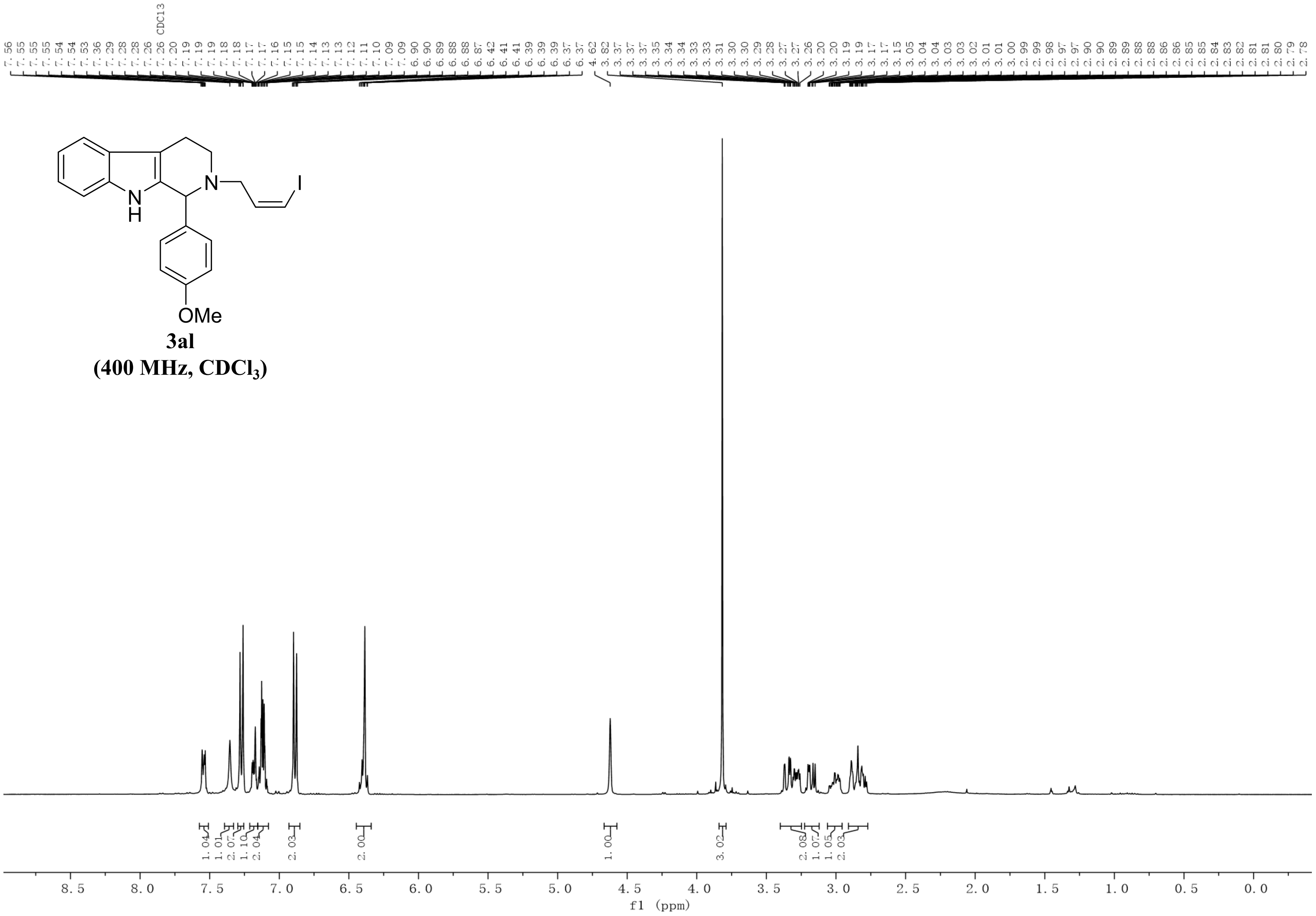
OMe
3al
(400 MHz, CDCl$_3$)
f1 (ppm)

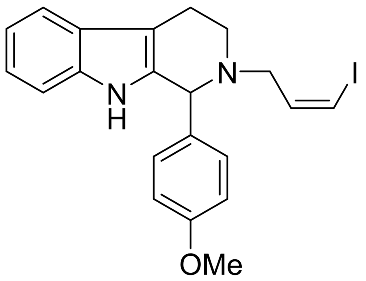

3al
(101 MHz, $CDCl_3$)

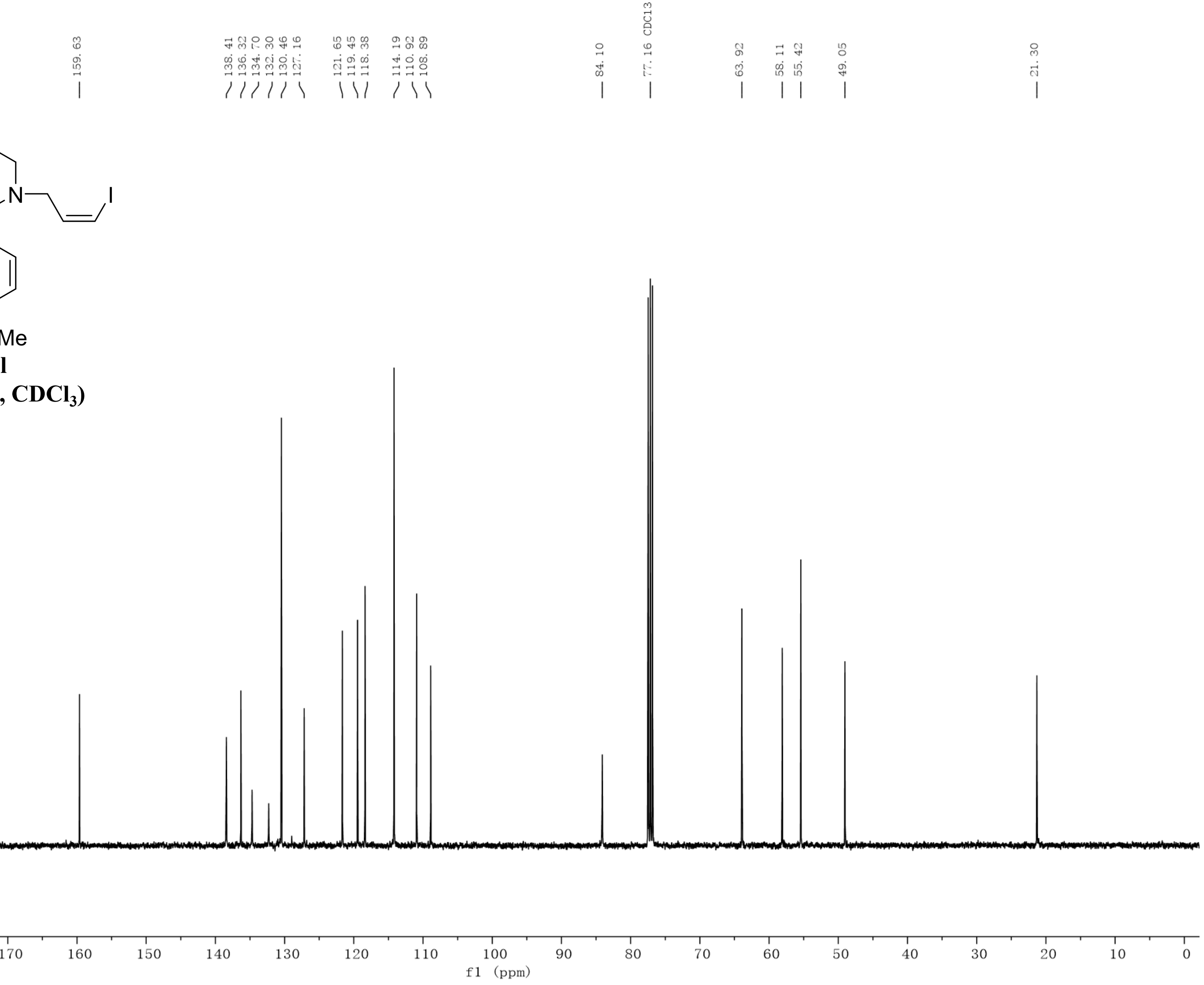

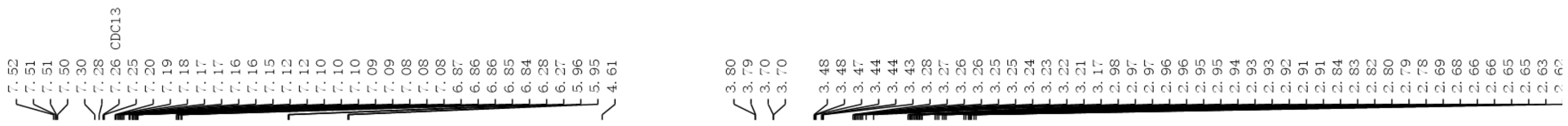

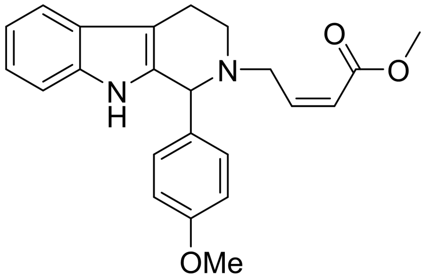

3am
(400 MHz, $CDCl_3$)

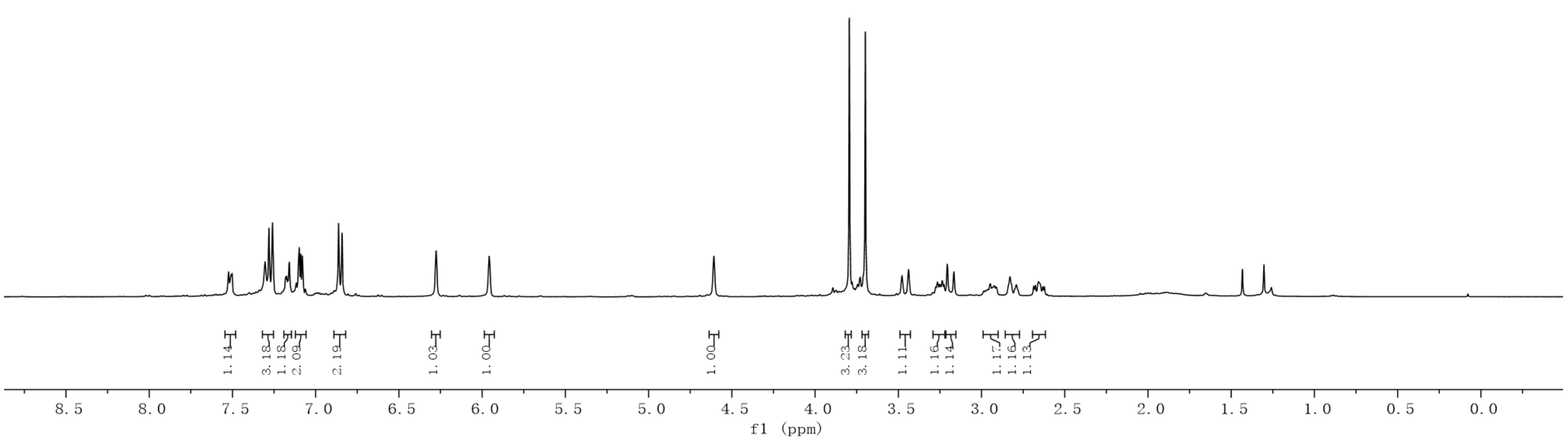

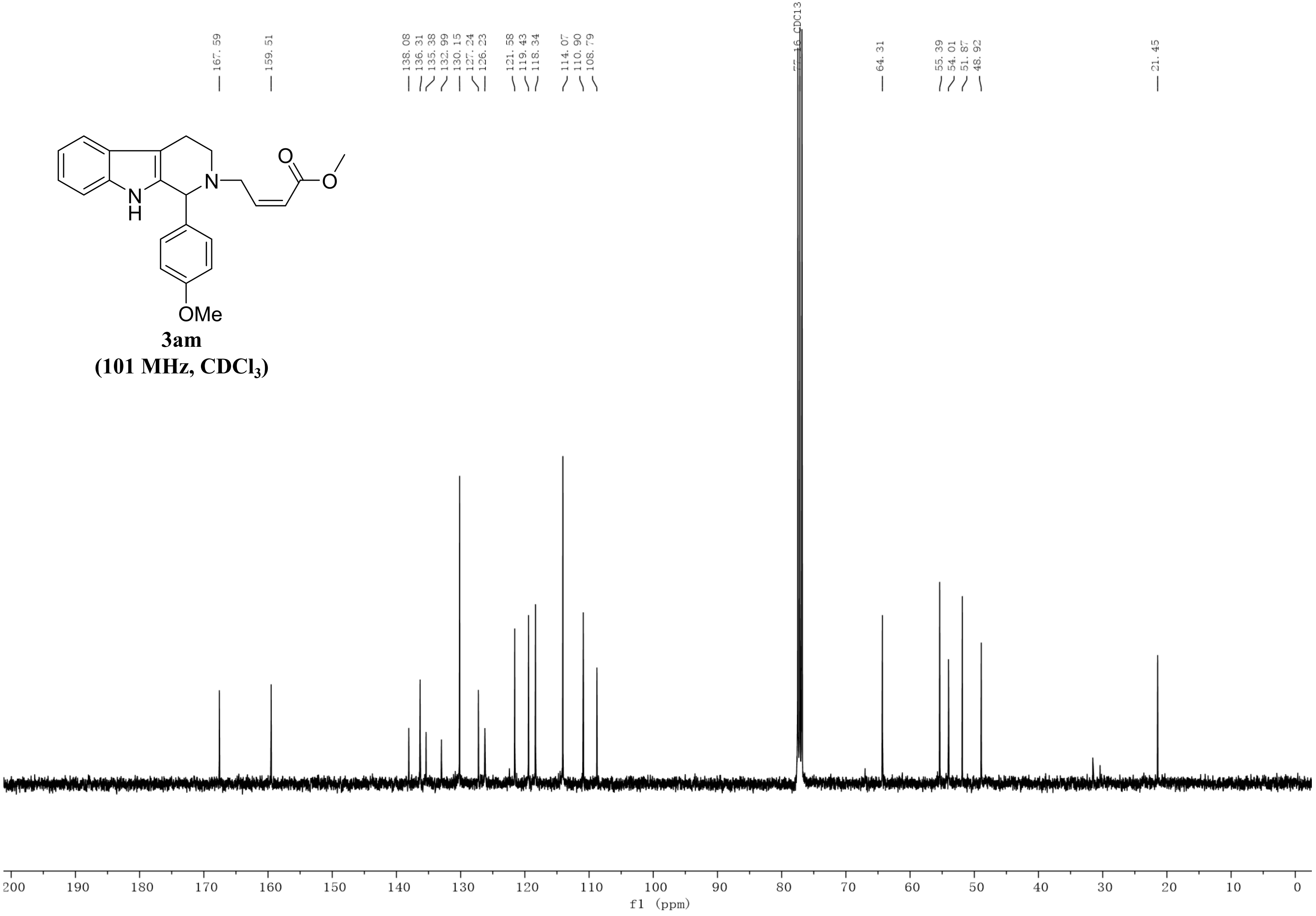

3am
(101 MHz, $CDCl_3$)

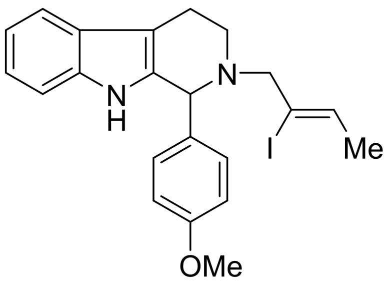

3an
(400 MHz, $CDCl_3$)

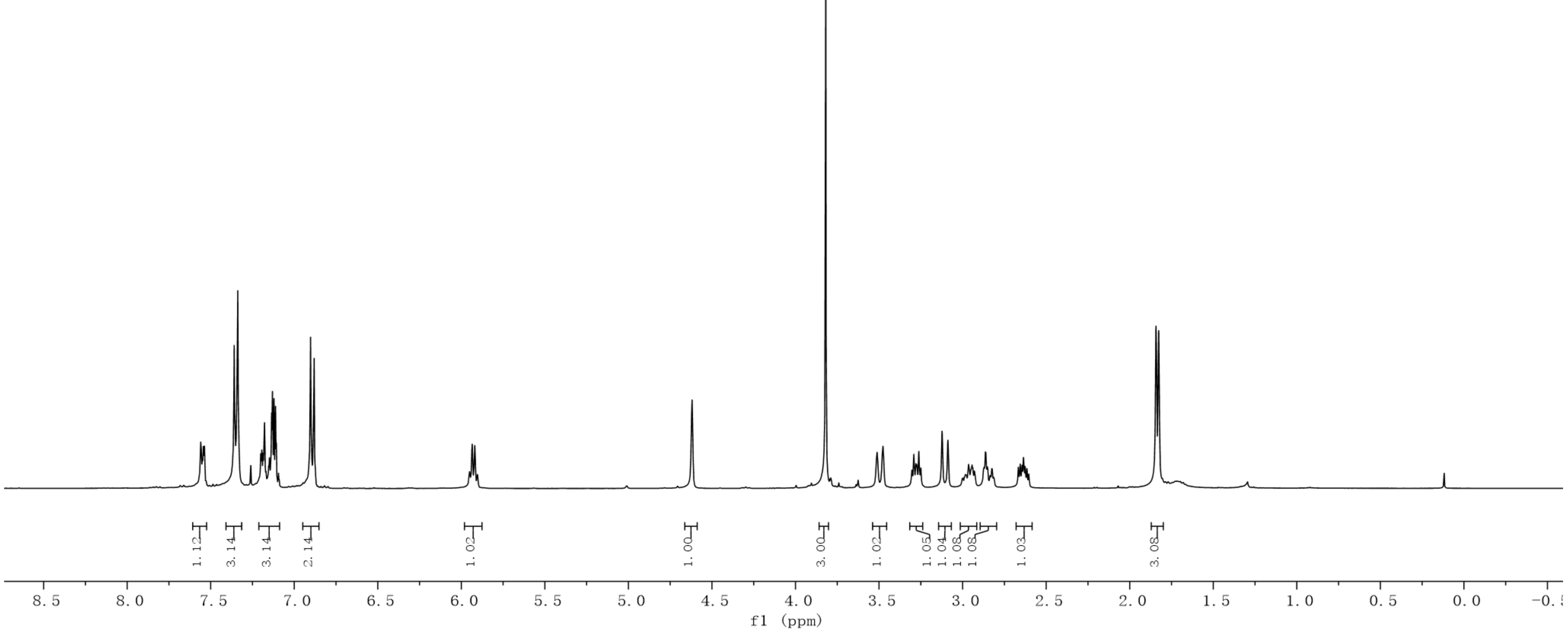

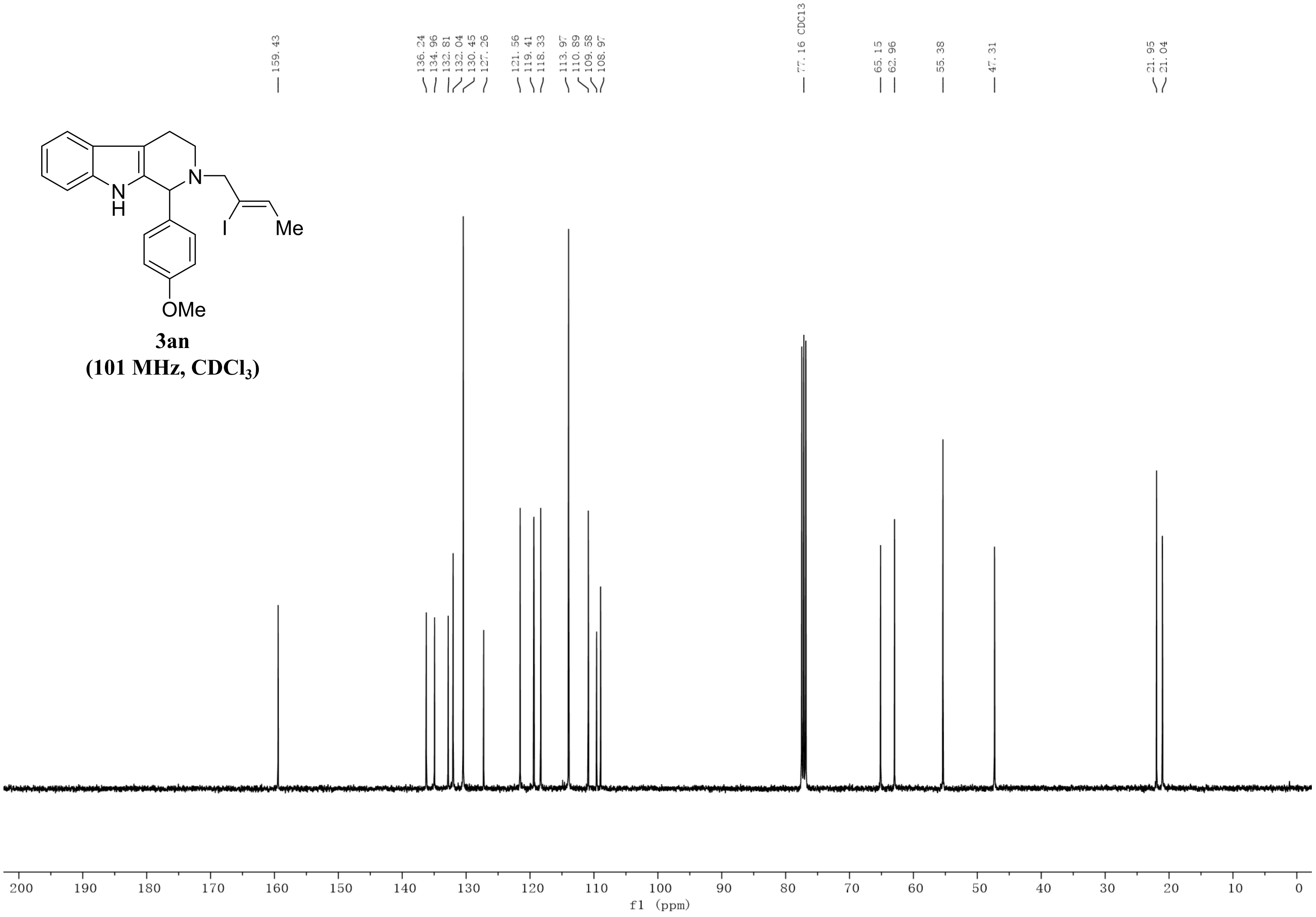

3an
(101 MHz, $CDCl_3$)

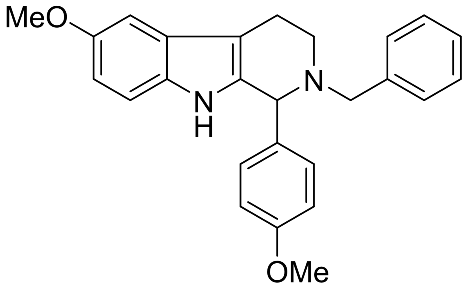

3ao
(600 MHz, $CDCl_3$)

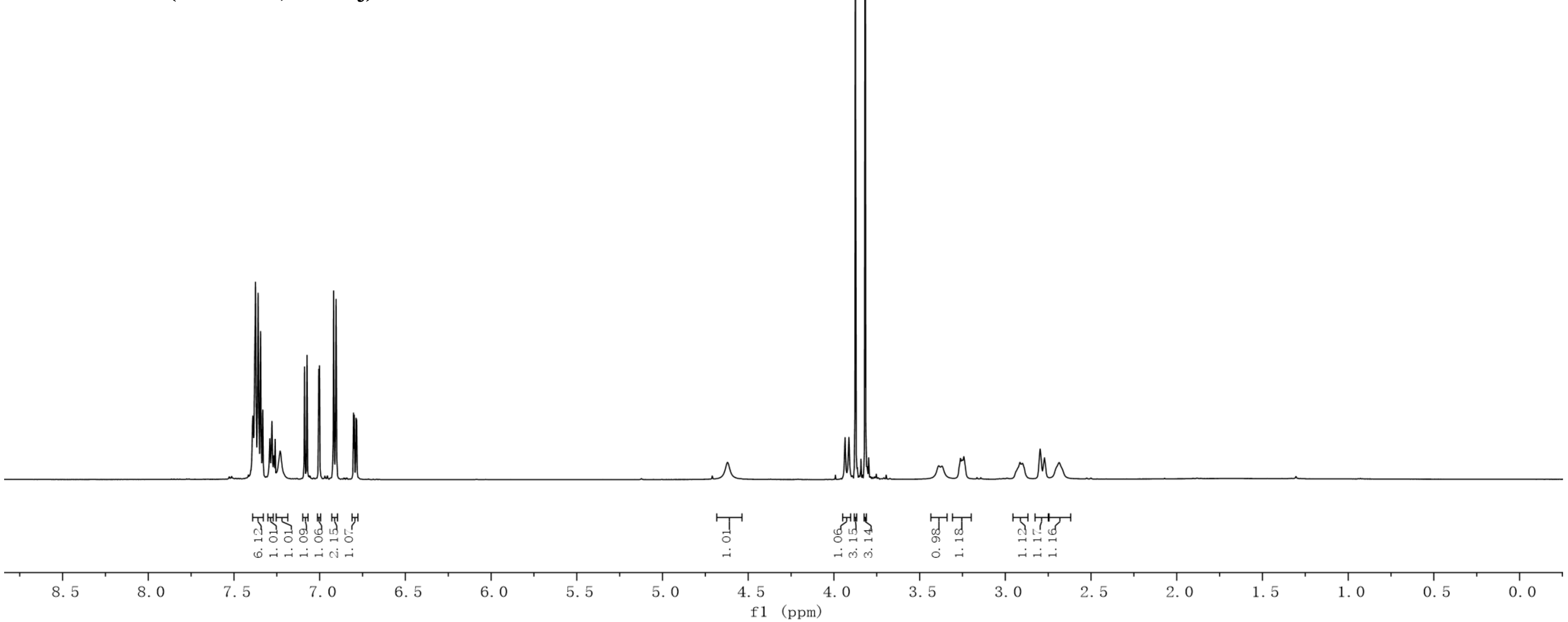

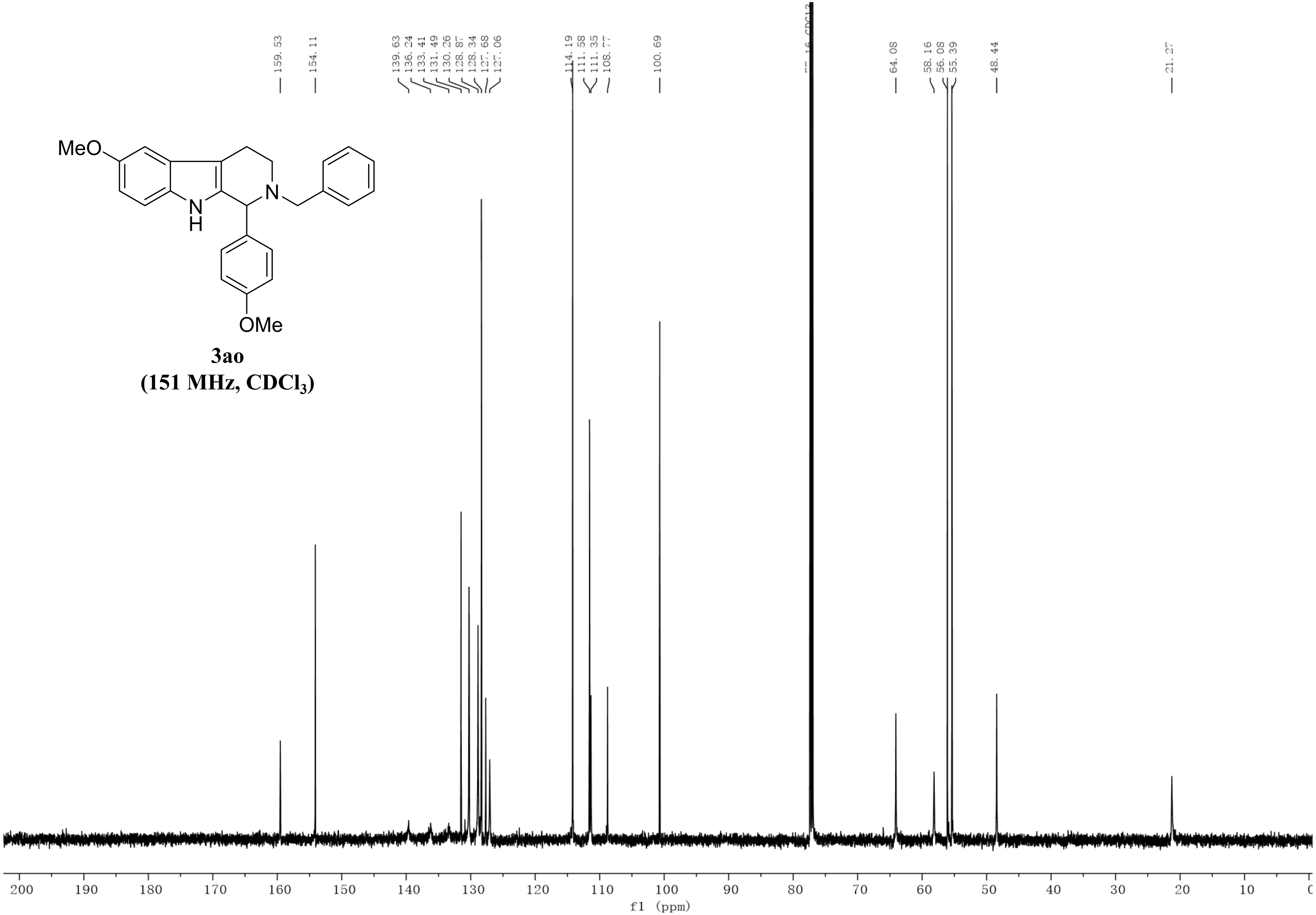

3ao
(151 MHz, $CDCl_3$)

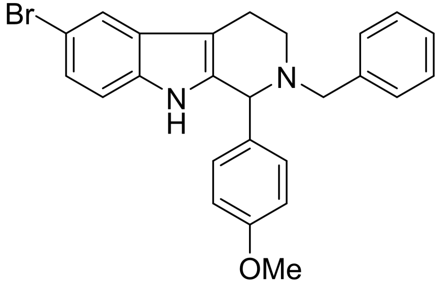

3ap
(500 MHz, DMSO-d6)

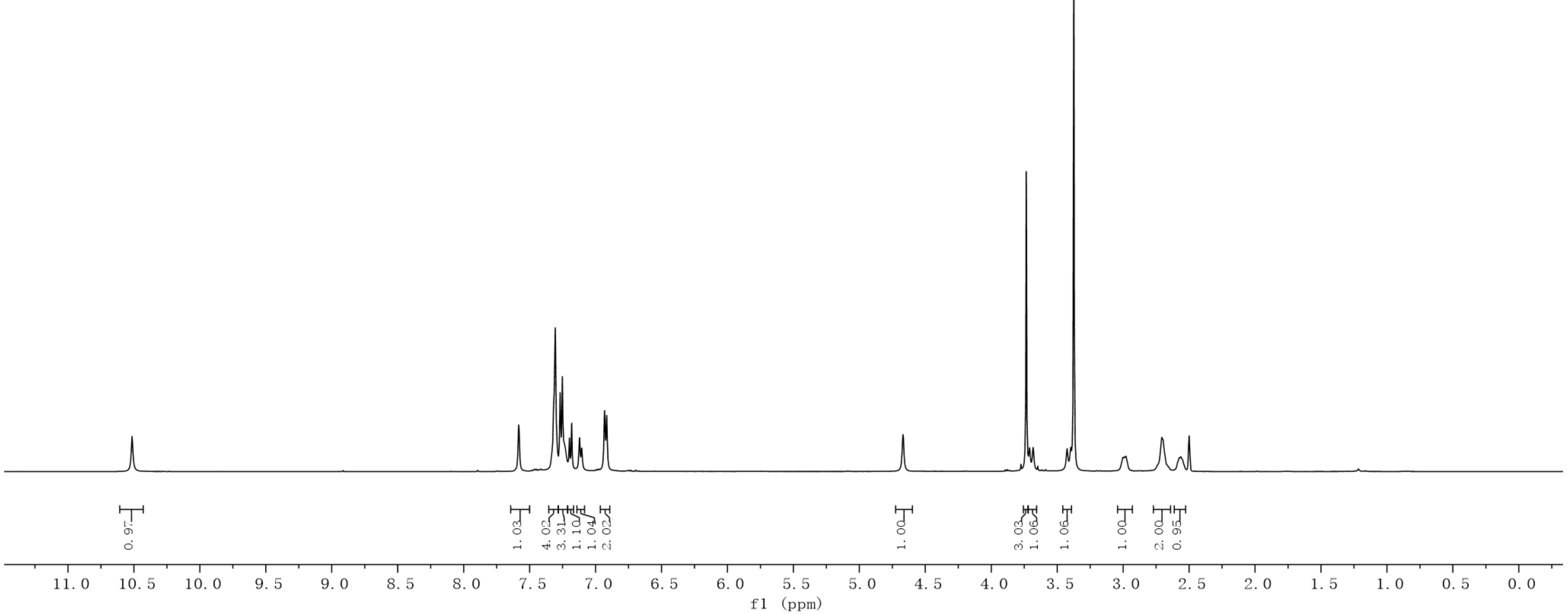

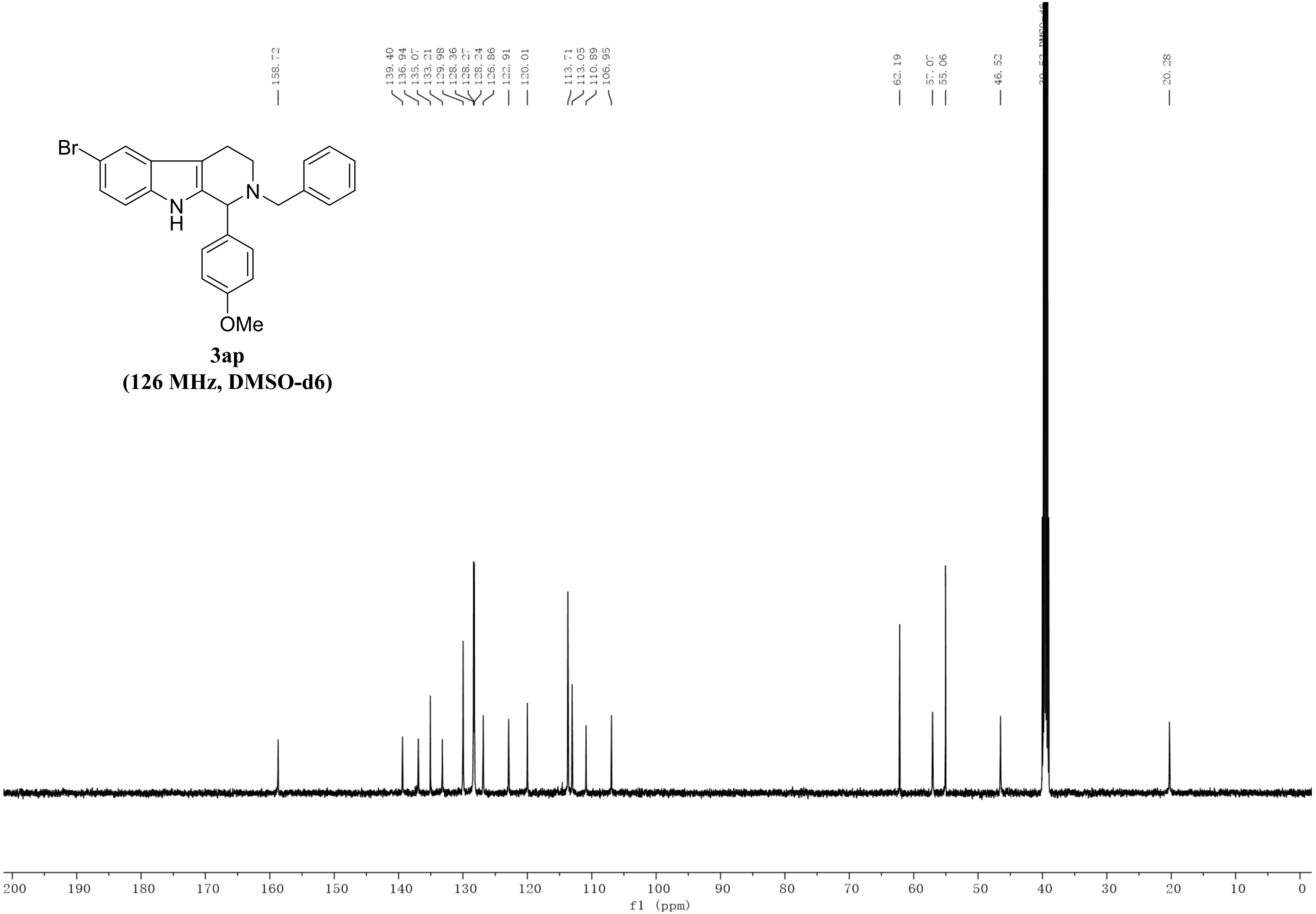

3ap
(126 MHz, DMSO-d6)

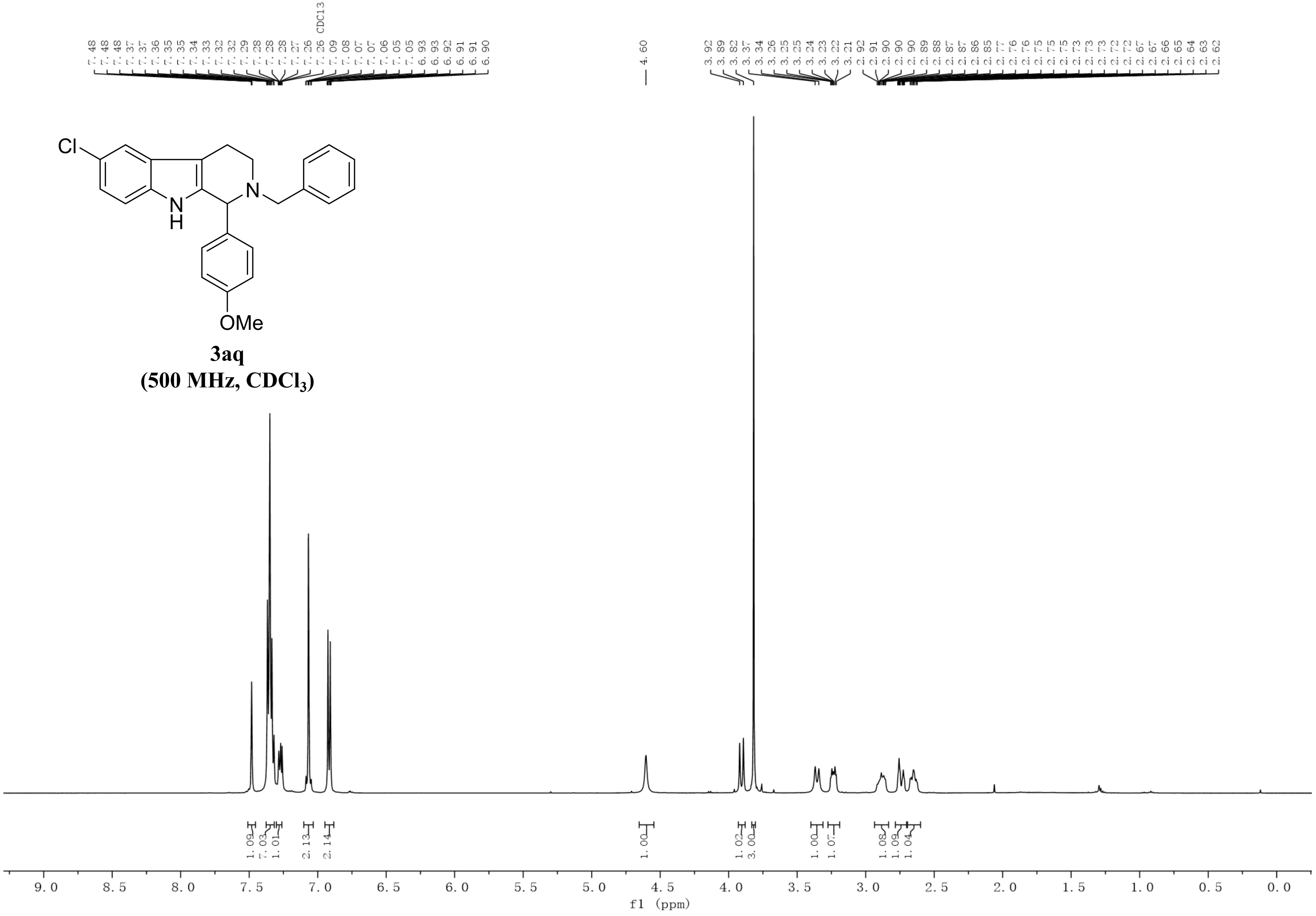

3aq
(500 MHz, $CDCl_3$)

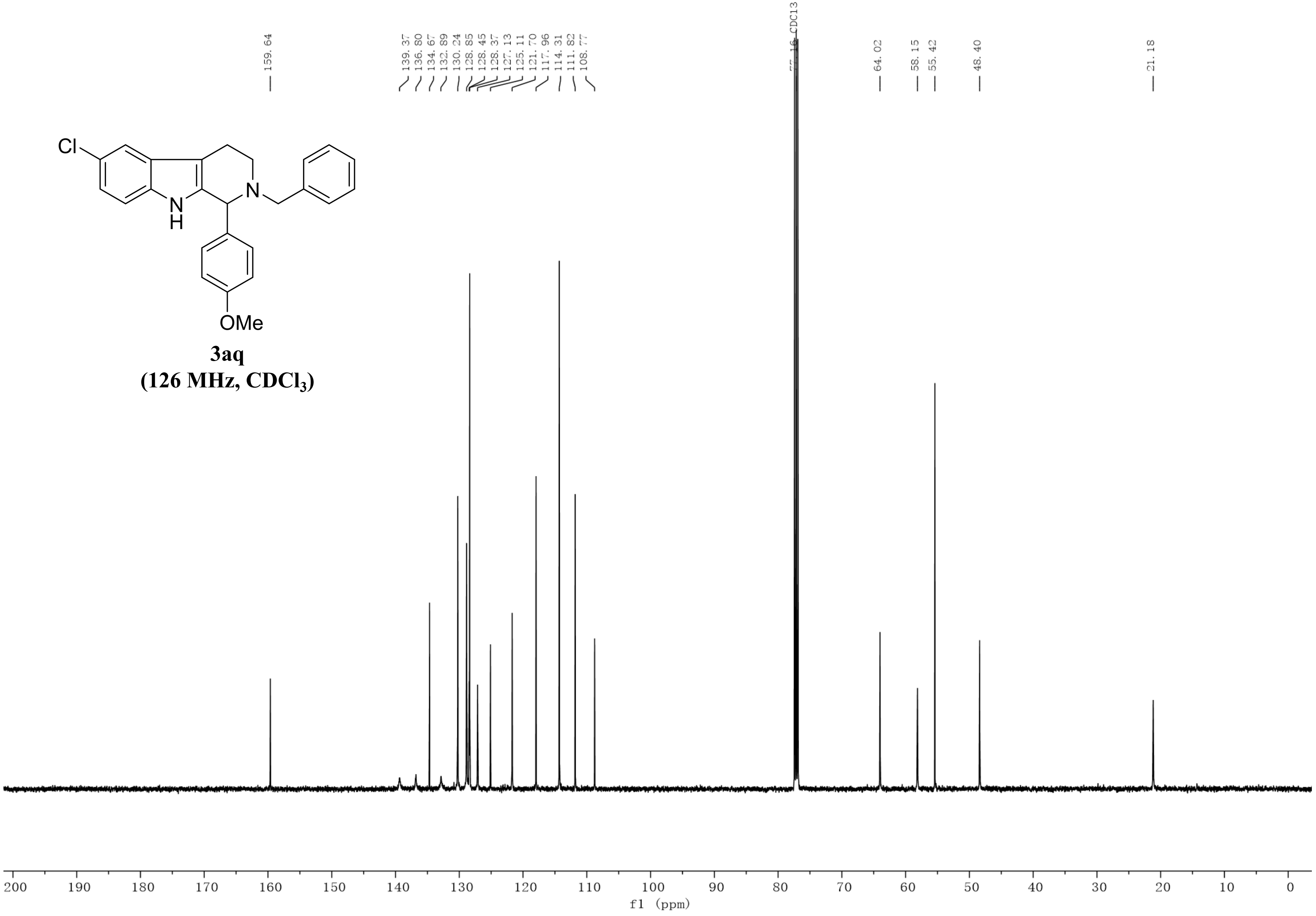

3aq
(126 MHz, $CDCl_3$)

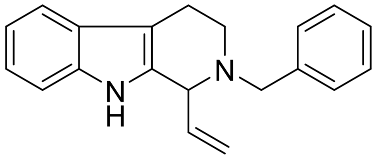

3b
(400 MHz, $CDCl_3$)

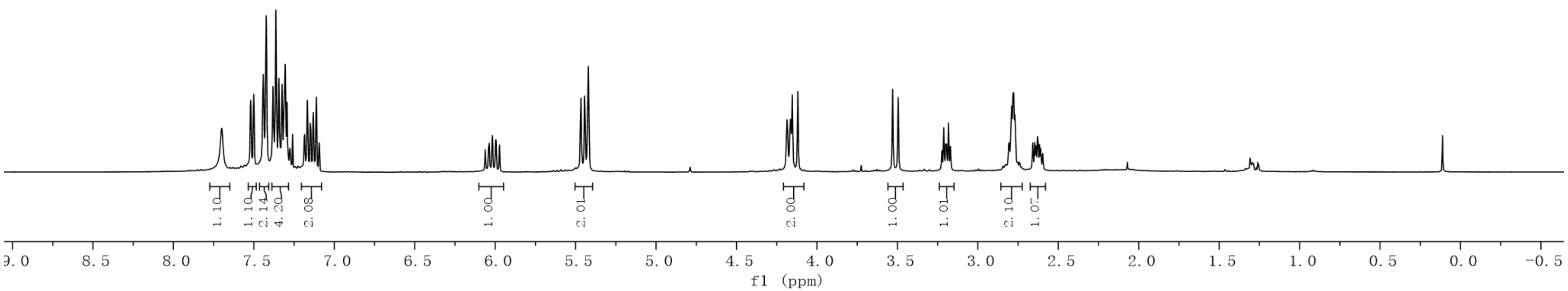

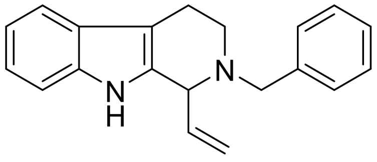

3b
(101 MHz, $CDCl_3$)

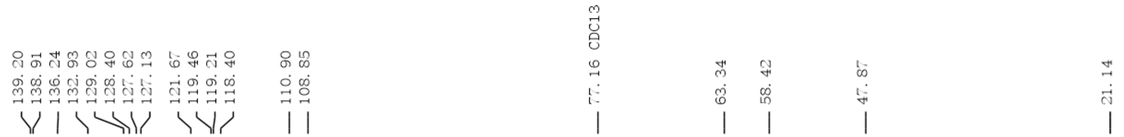

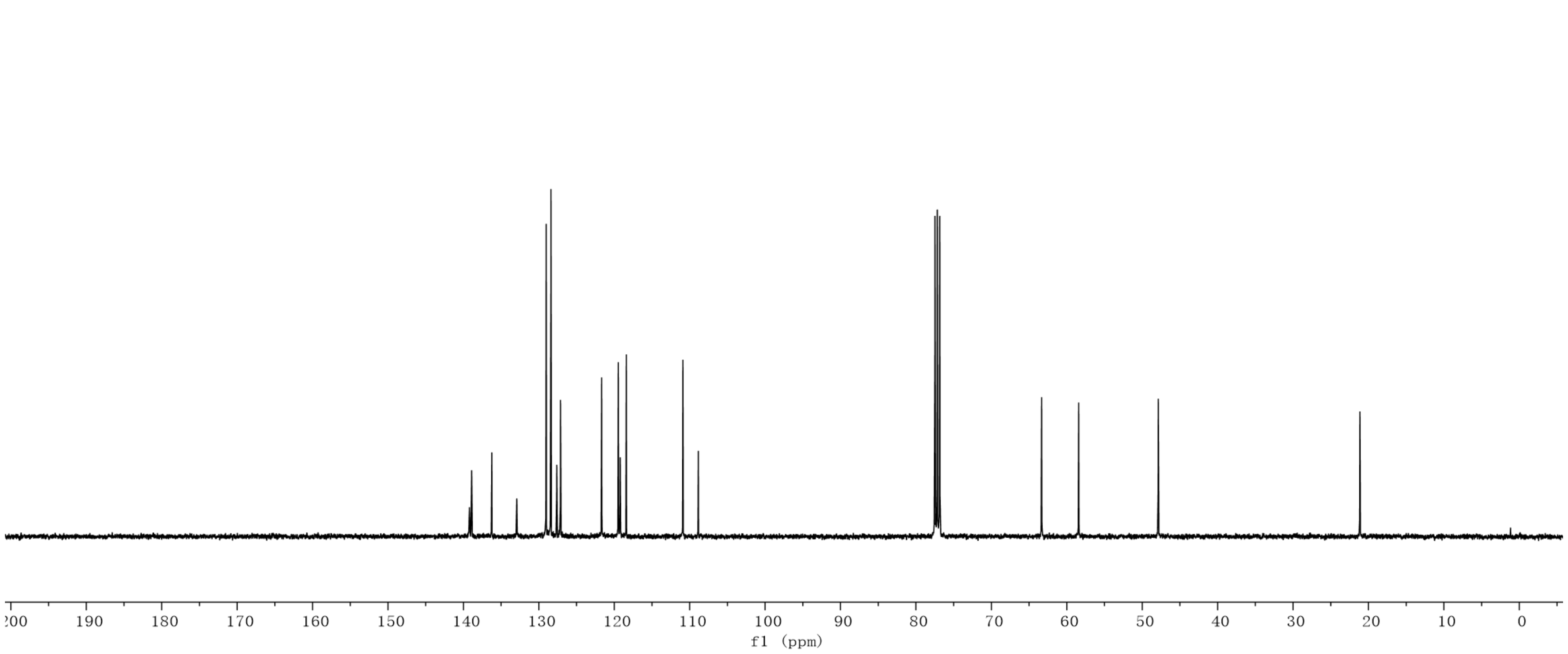

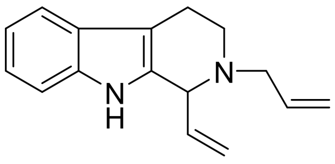

3as
(400 MHz, $CDCl_3$)

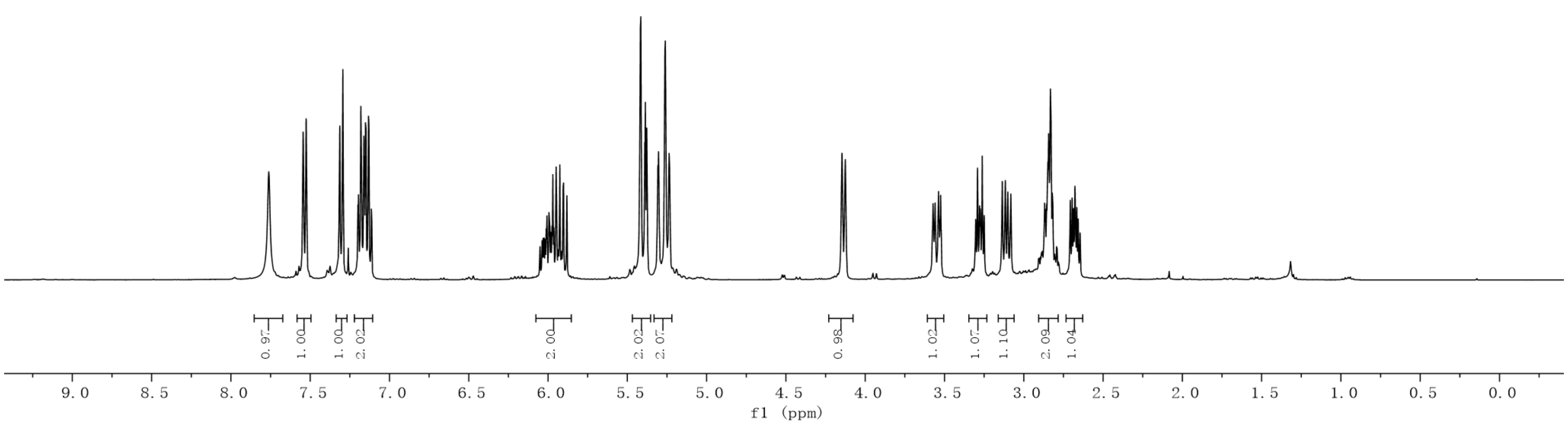

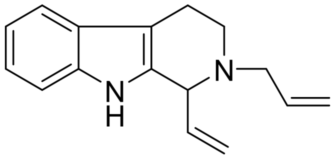

3as
(101 MHz, $CDCl_3$)

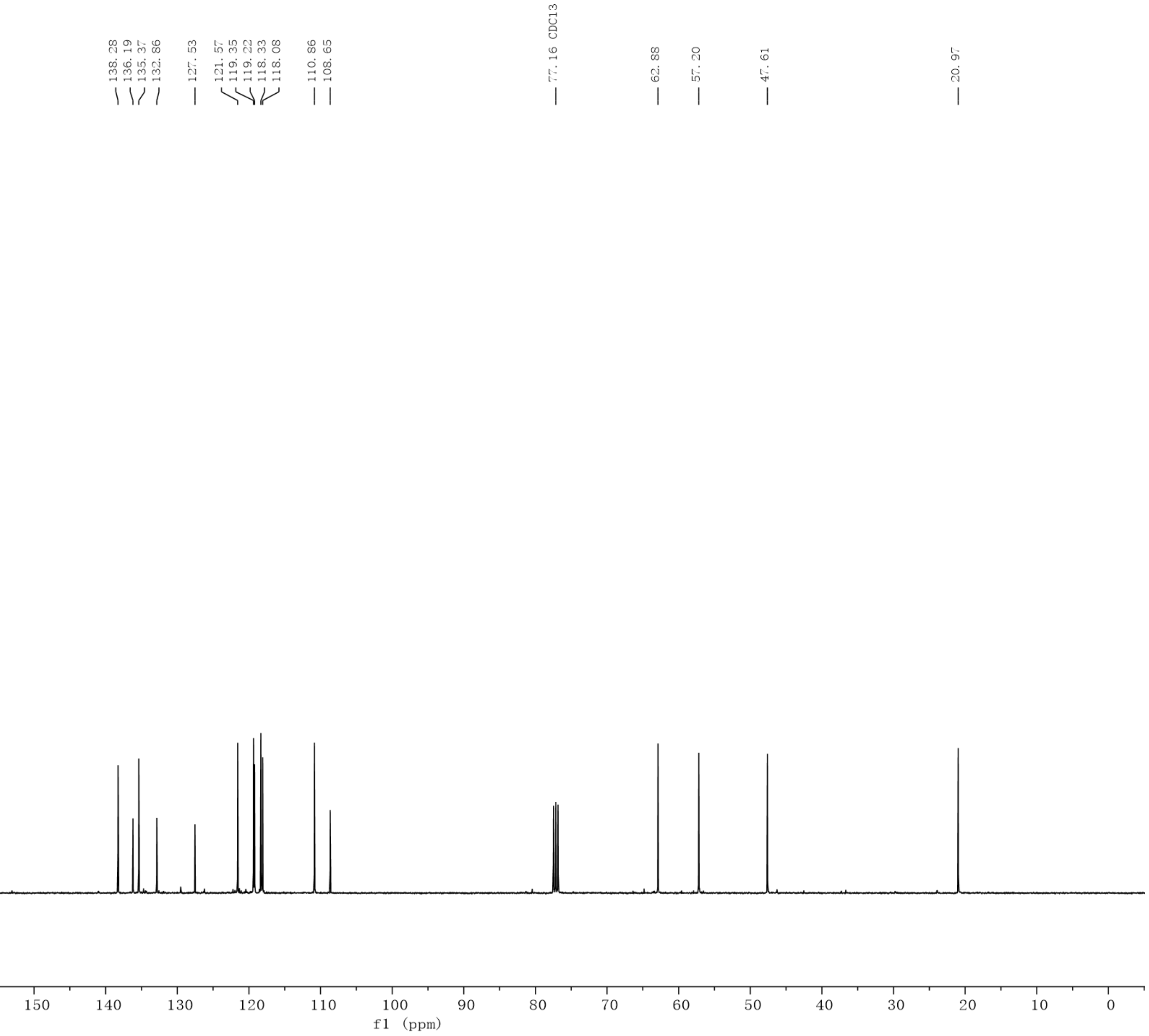

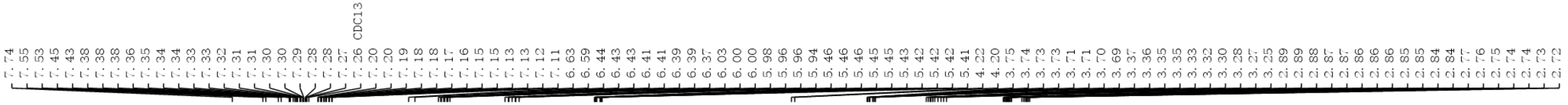

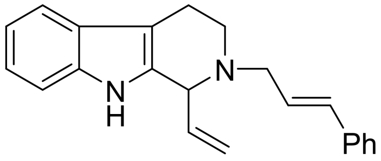

3at
(400 MHz, $CDCl_3$)

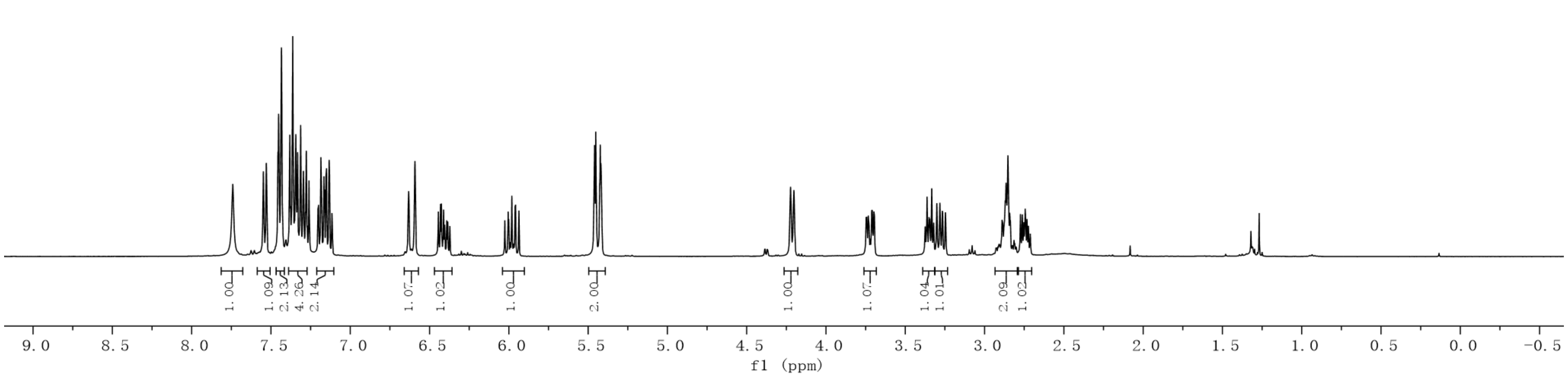

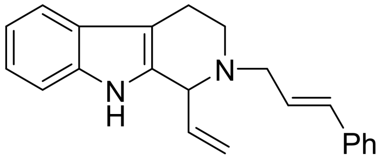

3at
(101 MHz, $CDCl_3$)

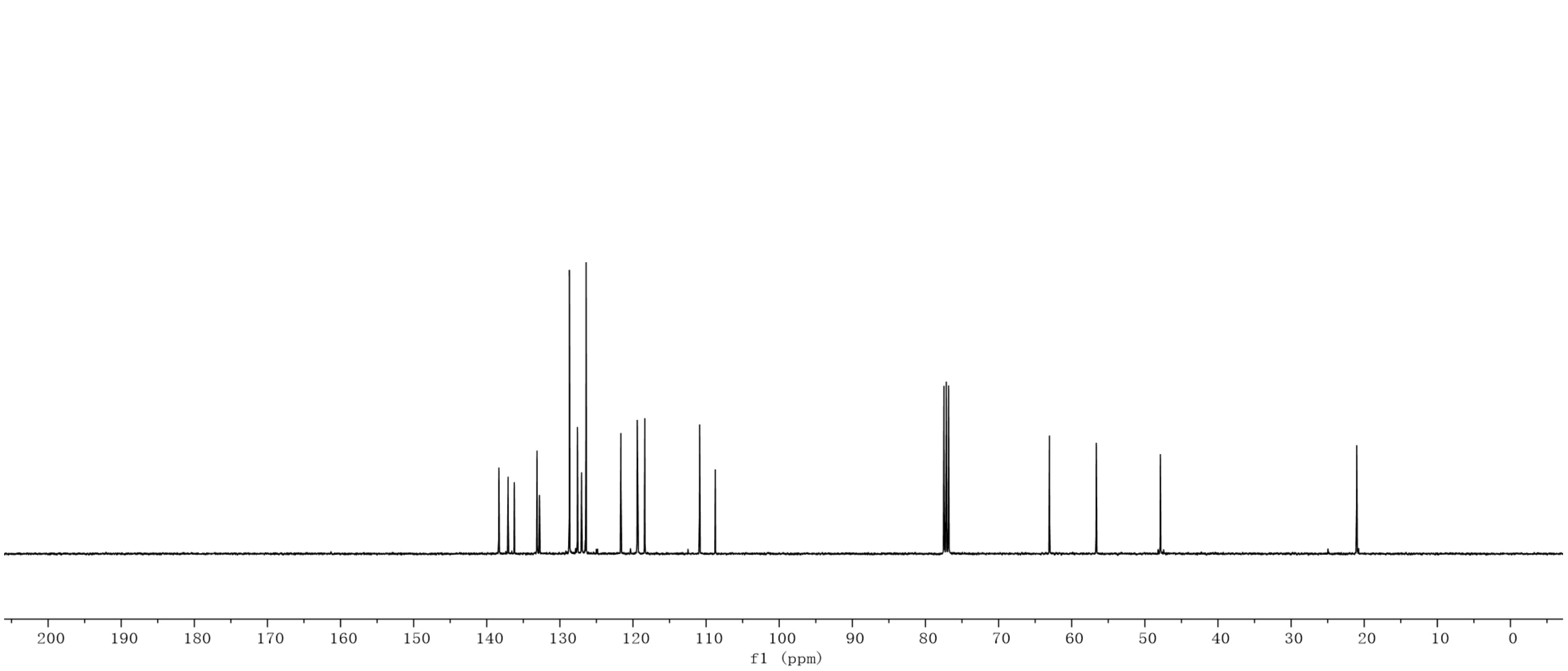

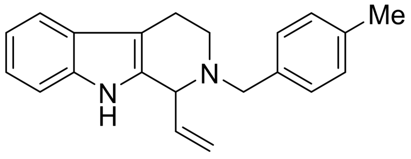

3au
(400 MHz, $CDCl_3$)

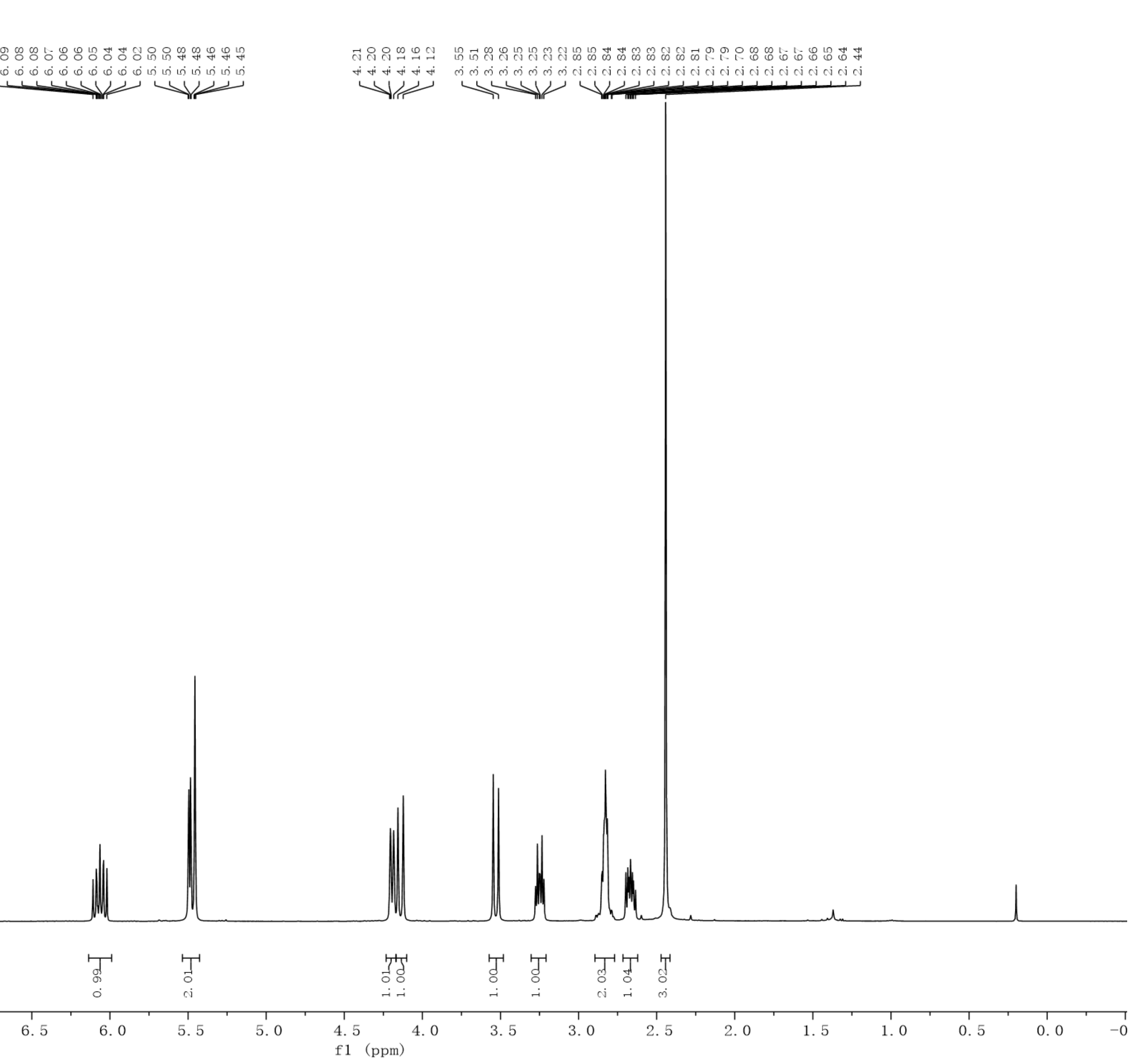

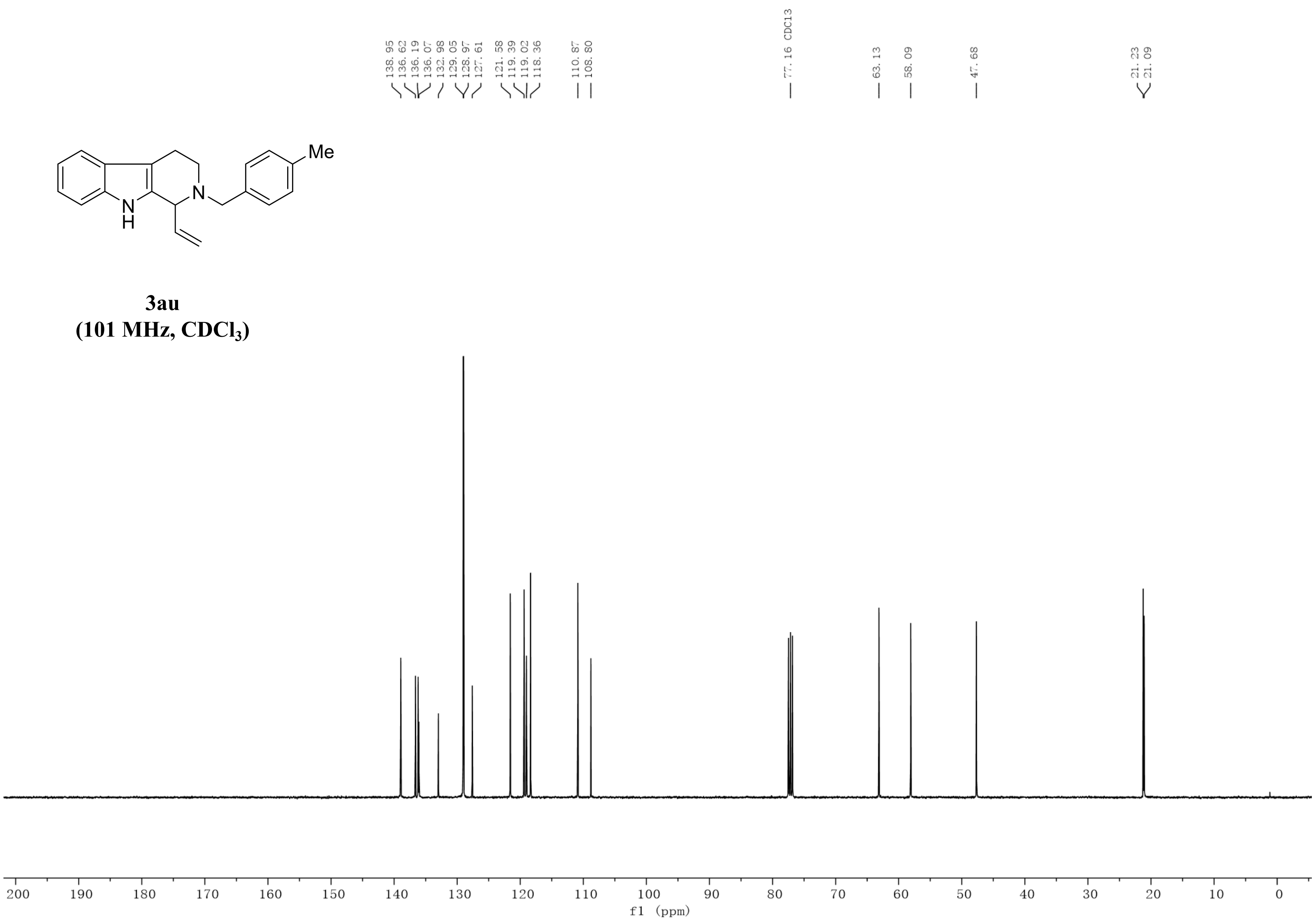
138.95
136.62
136.19
136.07
132.98
129.05
128.97
127.61
121.58
119.39
119.02
118.36
110.87
108.80
77.16 CDCl3
63.13
58.09
47.68
21.23
21.09
Me
N
N
H
3au
(101 MHz, CDCl3)
200 190 180 170 160 150 140 130 120 110 100 90 80 70 60 50 40 30 20 10 0
f1 (ppm)

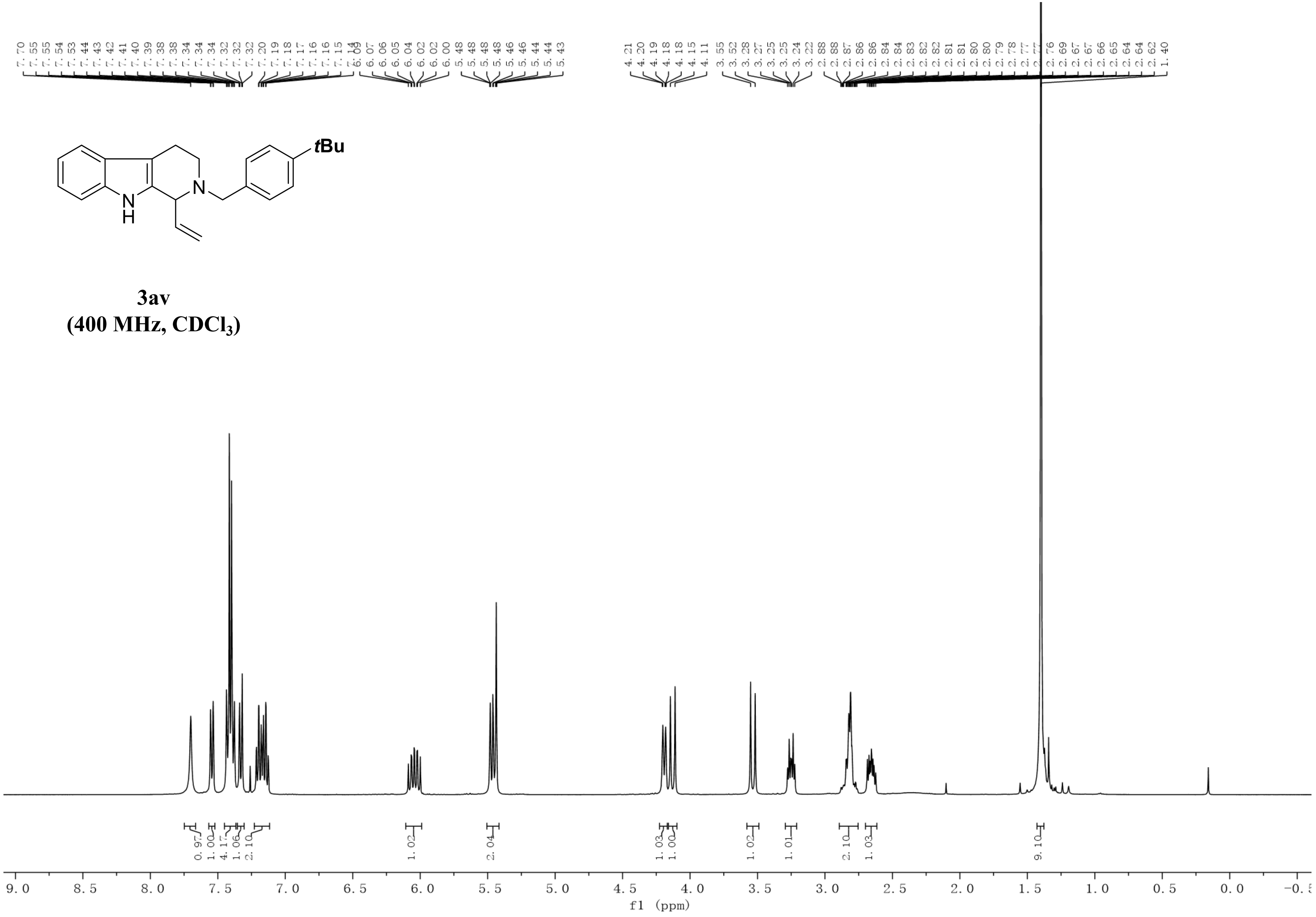

3av
(400 MHz, $CDCl_3$)

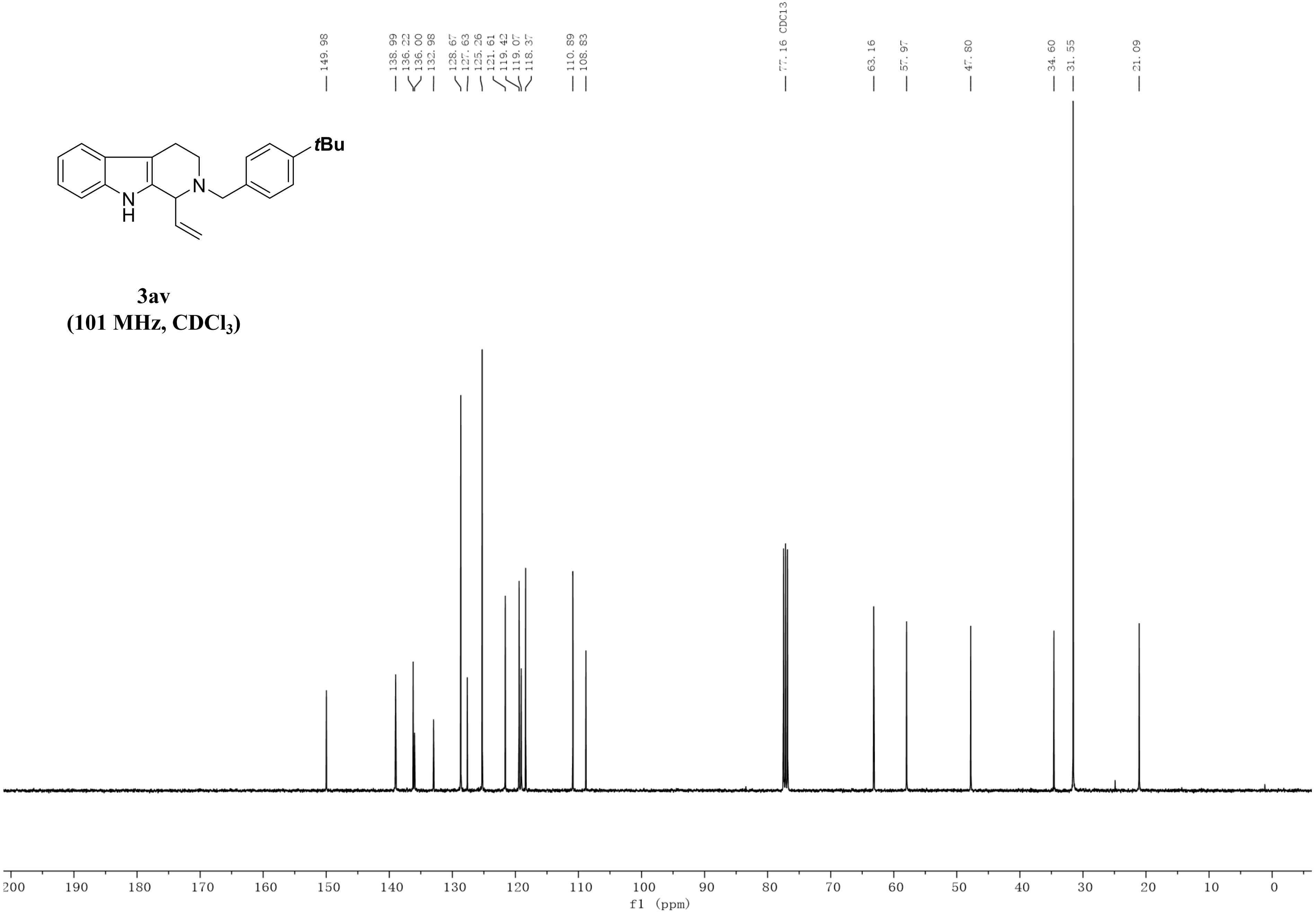

tBu
N
N
H
3av
(101 MHz, CDCl3)
149.98
138.99
136.22
136.00
132.98
128.67
127.63
125.26
121.61
119.42
119.07
118.37
110.89
108.83
77.16 CDCl3
63.16
57.97
47.80
34.60
31.55
21.09
200
190
180
170
160
150
140
130
120
110
100
90
80
70
60
50
40
30
20
10
0
f1 (ppm)

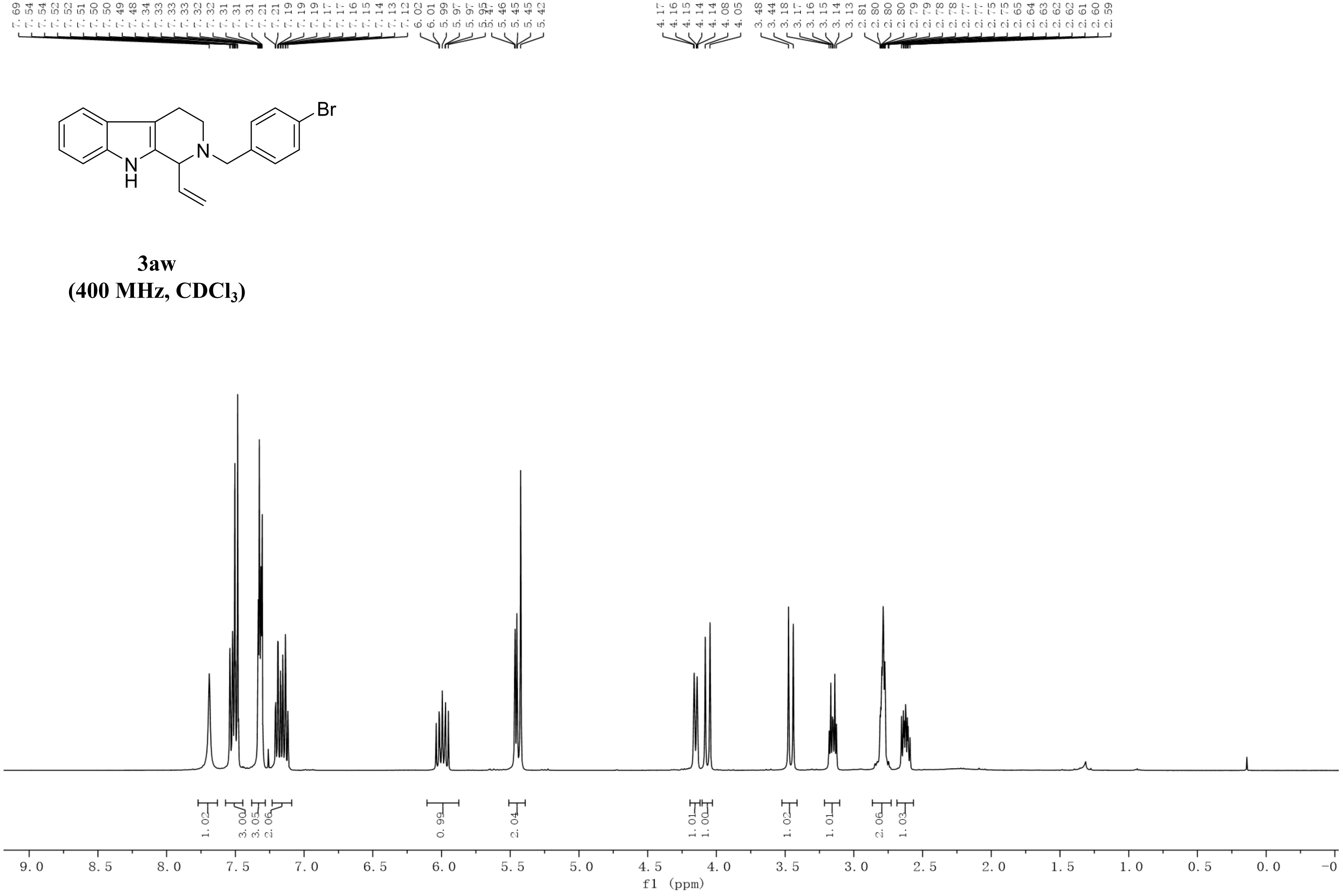

3aw
(400 MHz, $CDCl_3$)

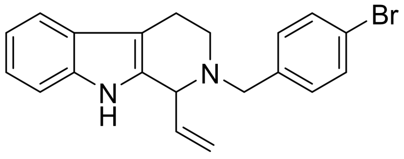

3aw
(101 MHz, $CDCl_3$)

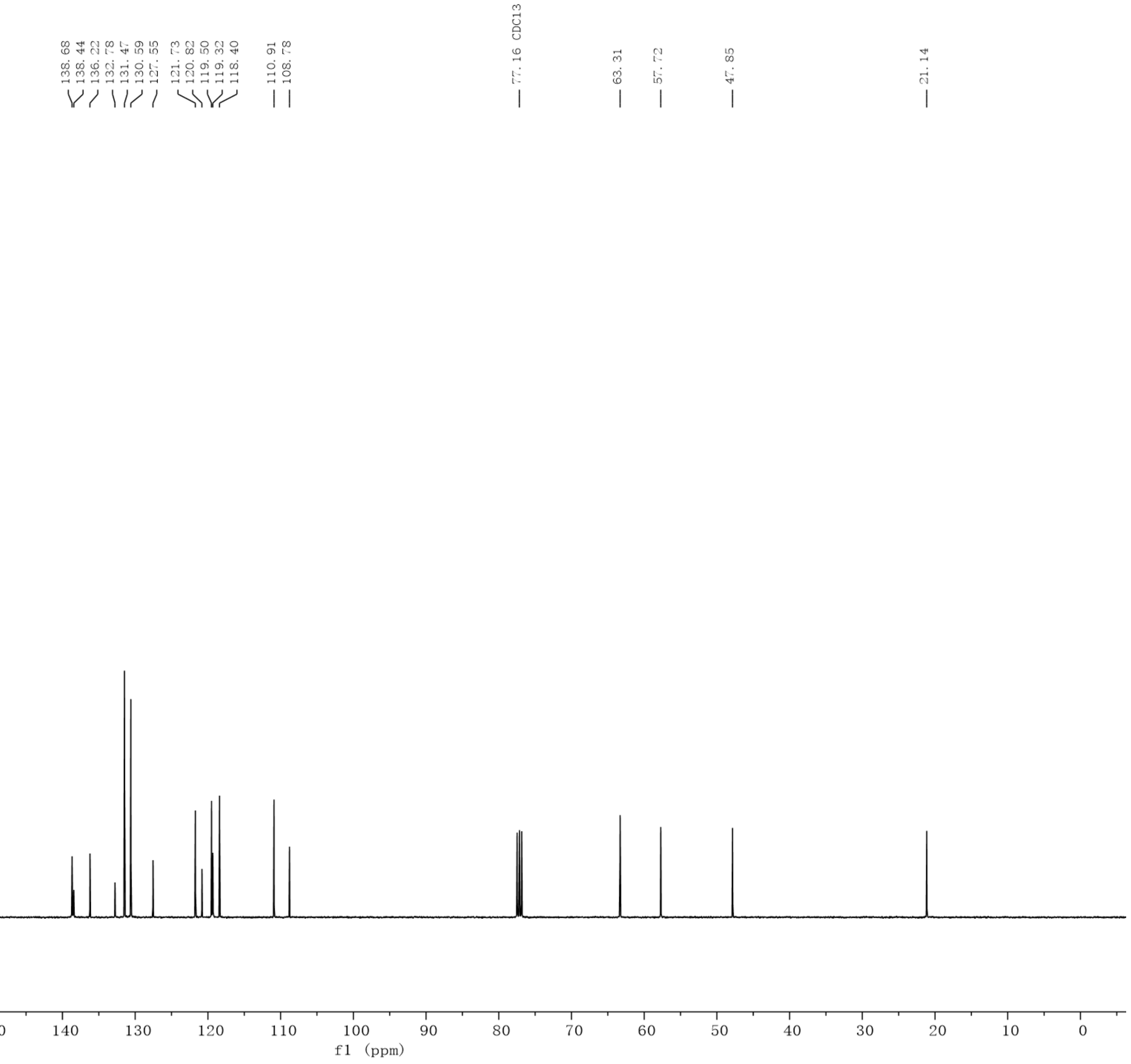

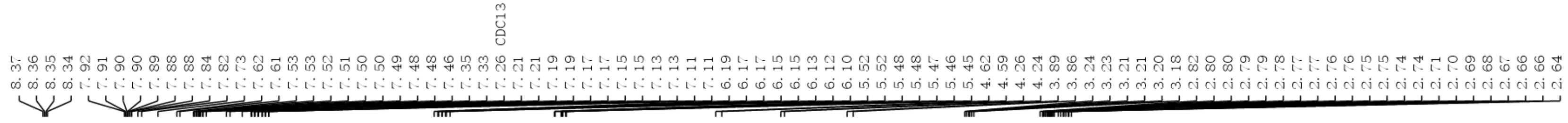

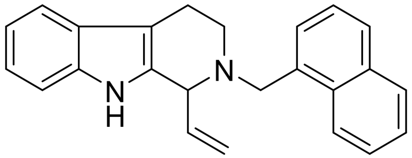

3ax
(400 MHz, $CDCl_3$)

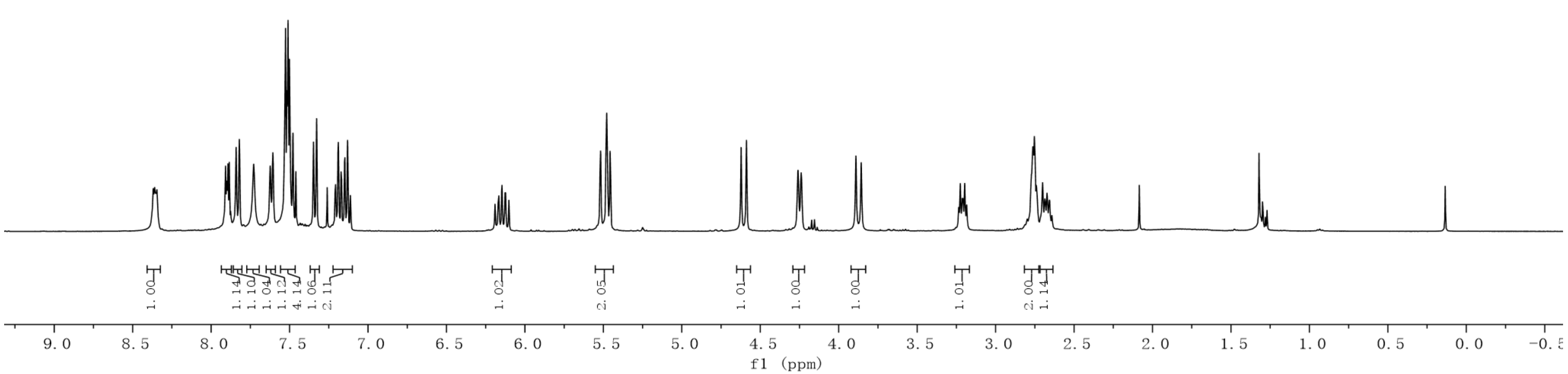

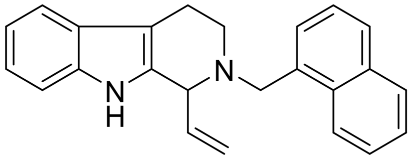

3ax
(101 MHz, $CDCl_3$)

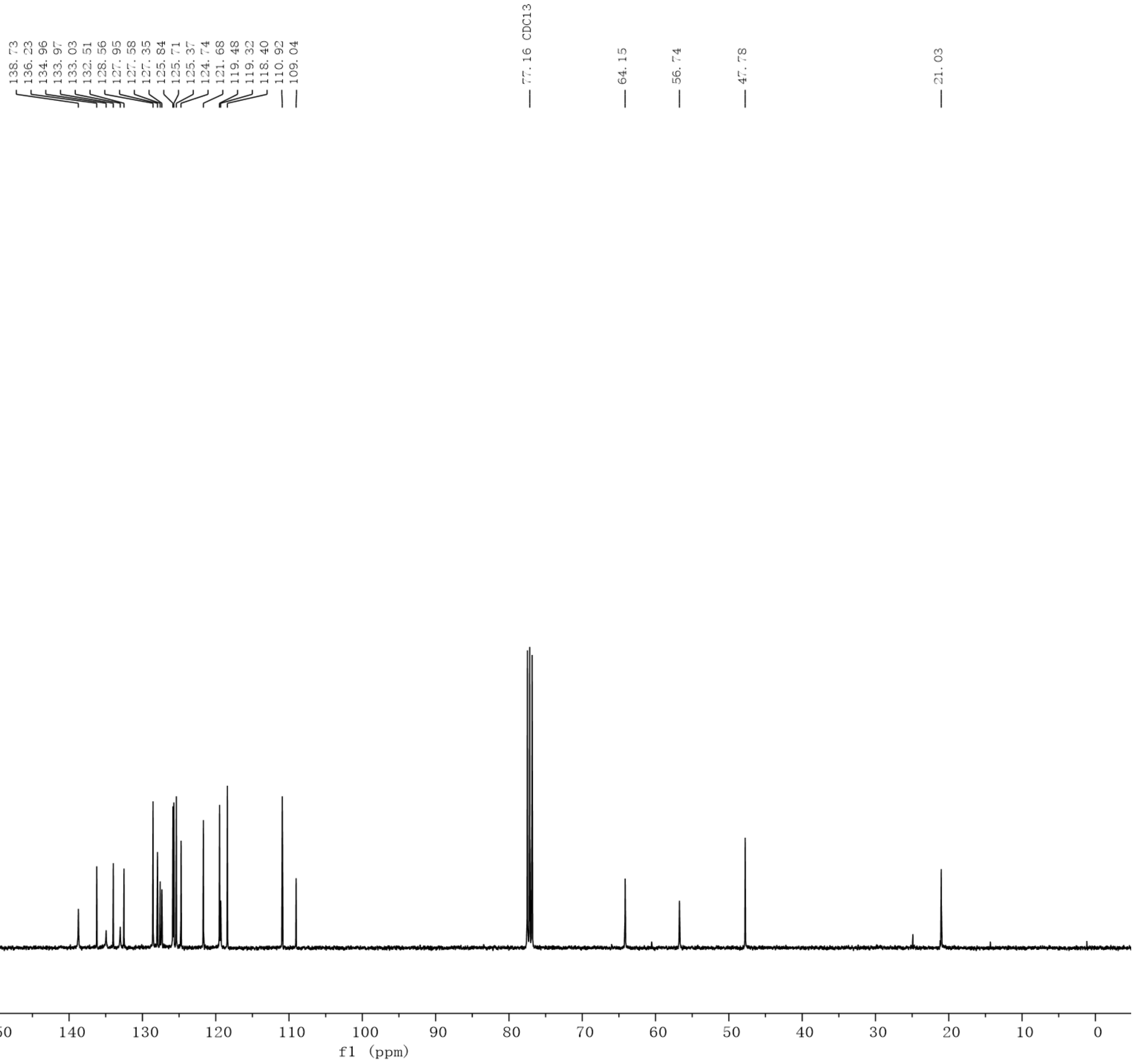

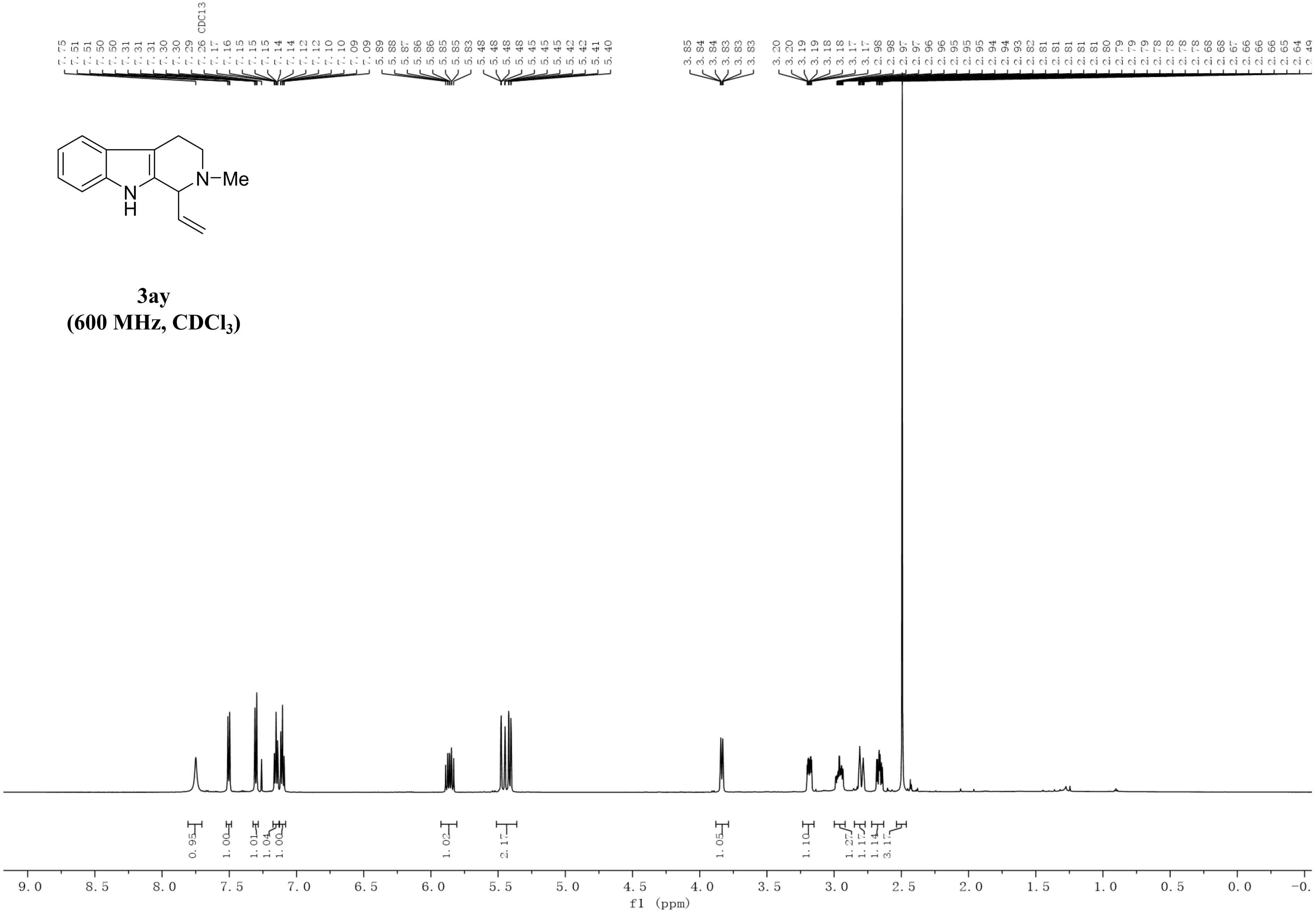

3ay
(600 MHz, $CDCl_3$)

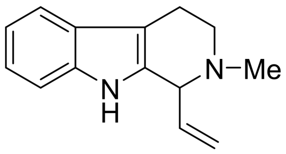

3ay
(151 MHz, $CDCl_3$)

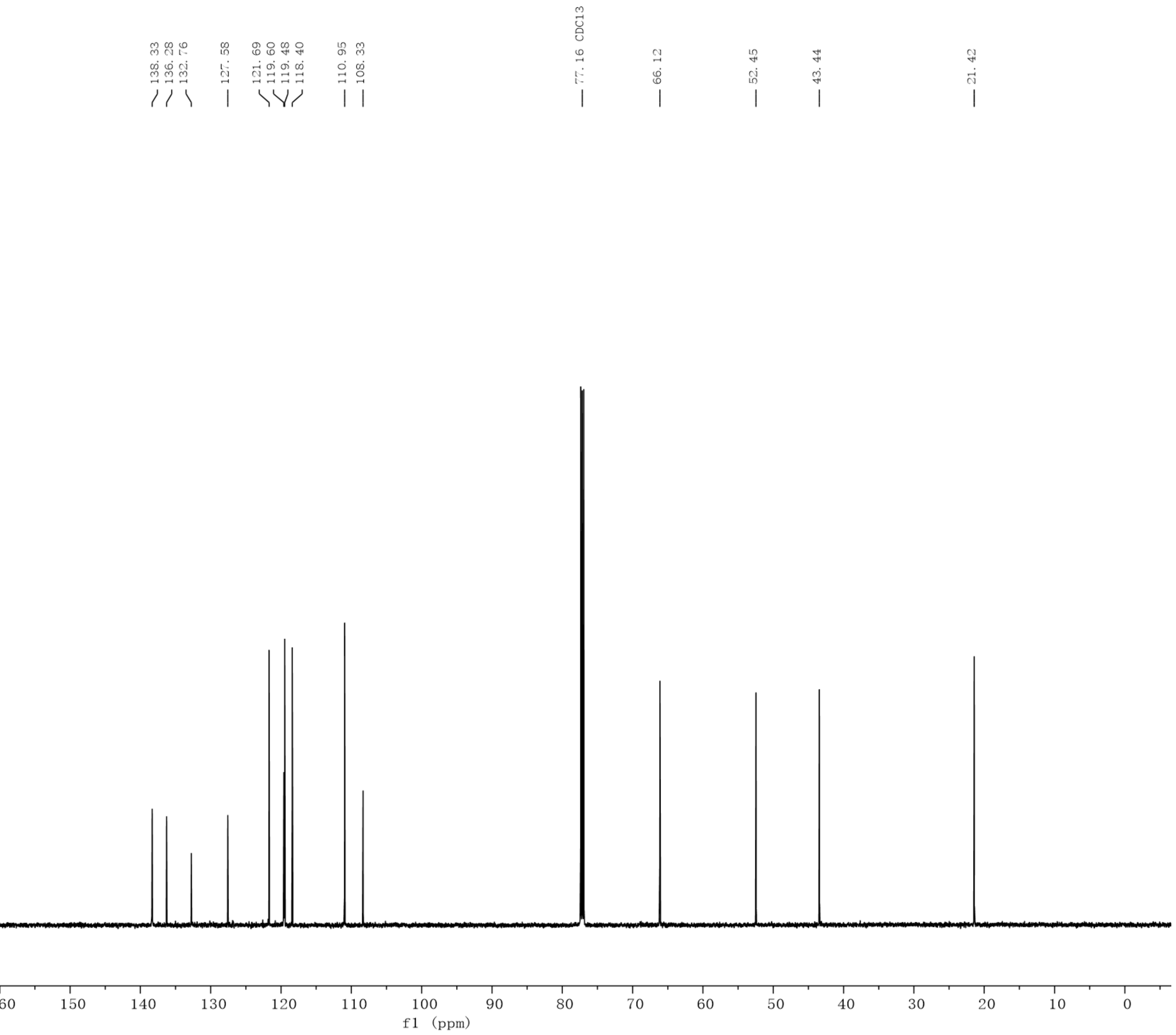

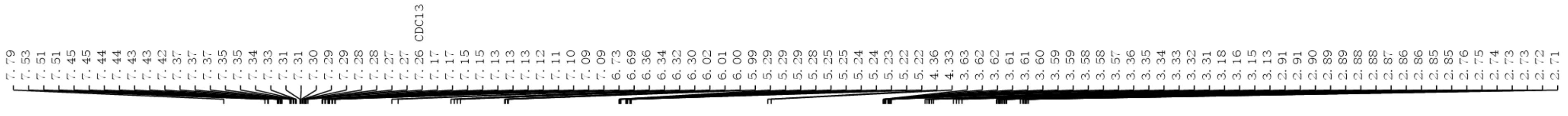

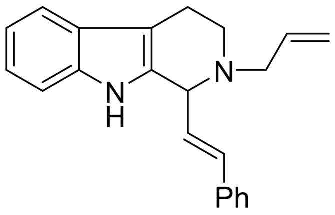

3az
(400 MHz, $CDCl_3$)

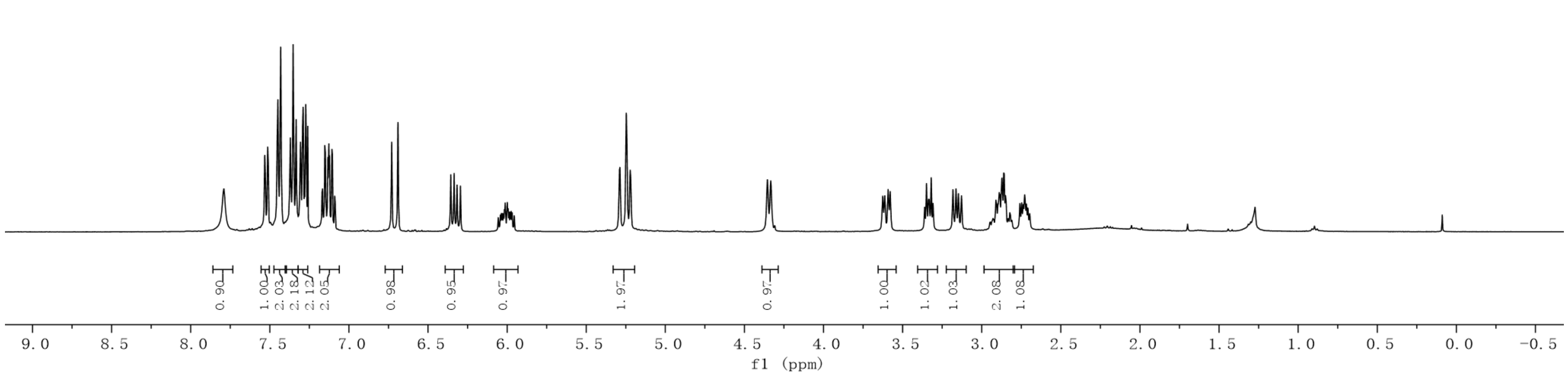

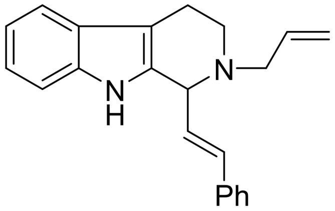

3az
(101 MHz, CDCl$_3$)

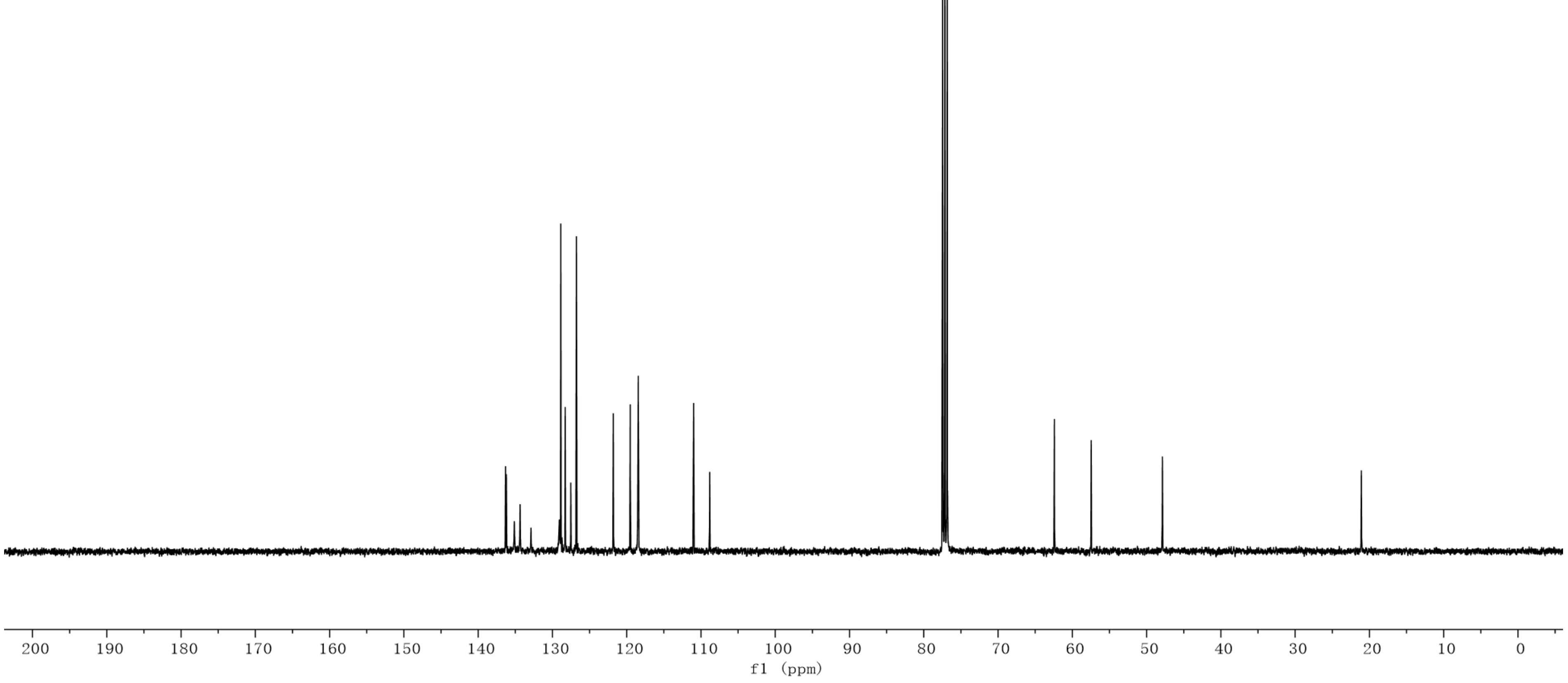

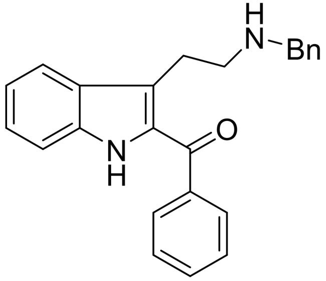

4

(600 MHz, $CDCl_3$)

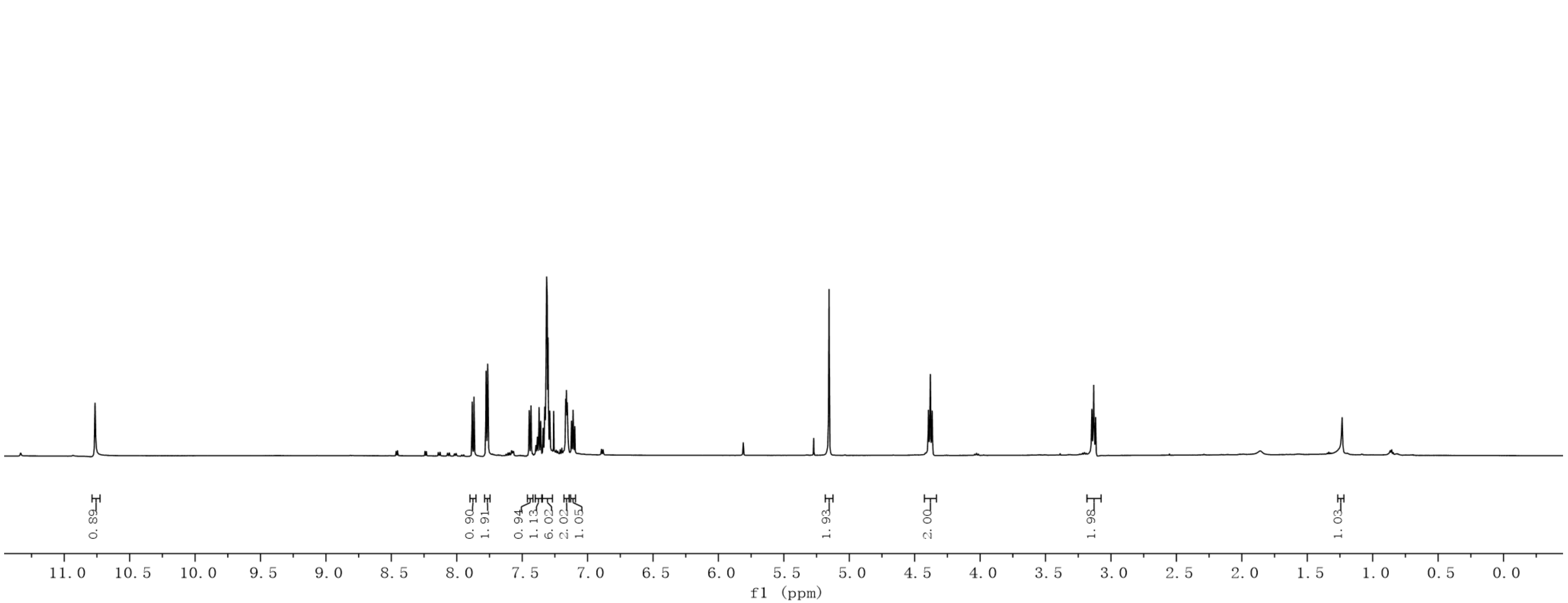

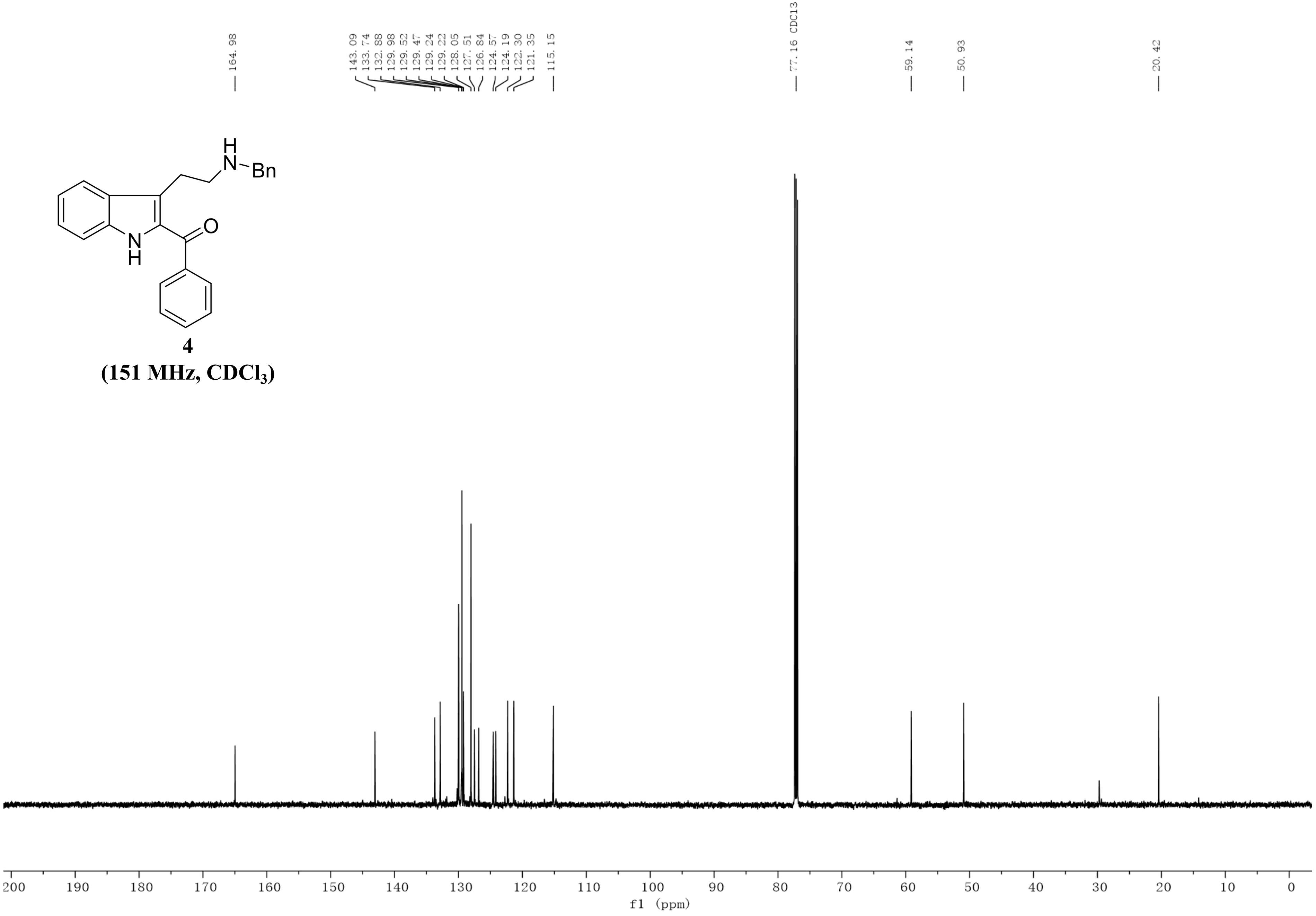
164.98
143.09
133.74
132.88
129.98
129.52
129.47
129.24
129.22
128.05
127.51
126.84
124.57
124.19
122.30
121.35
115.15
77.16 CDCl3
59.14
50.93
20.42
N
H
Bn
O
N
H
4
(151 MHz, $CDCl_3$)
200
190
180
170
160
150
140
130
120
110
100
90
80
70
60
50
40
30
20
10
0
f1 (ppm)

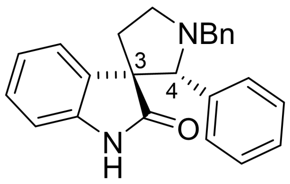

5
(500 MHz, $CDCl_3$)

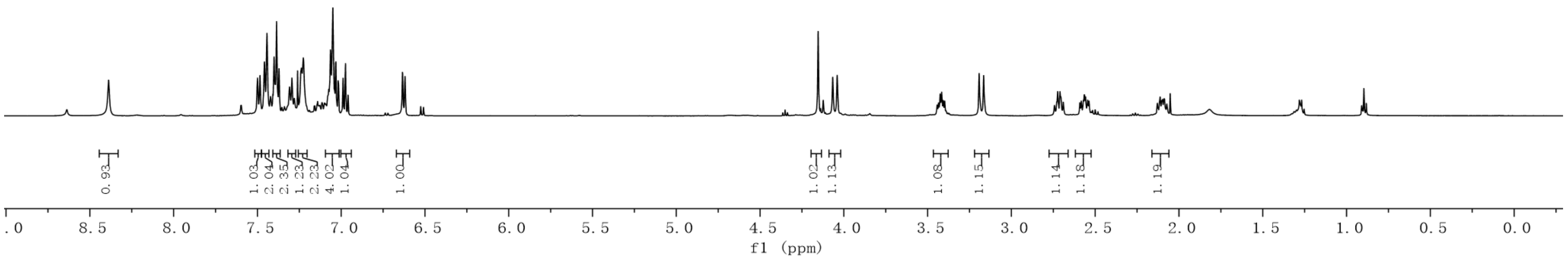

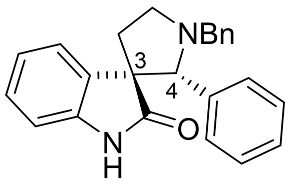

5
(126 MHz, $CDCl_3$)

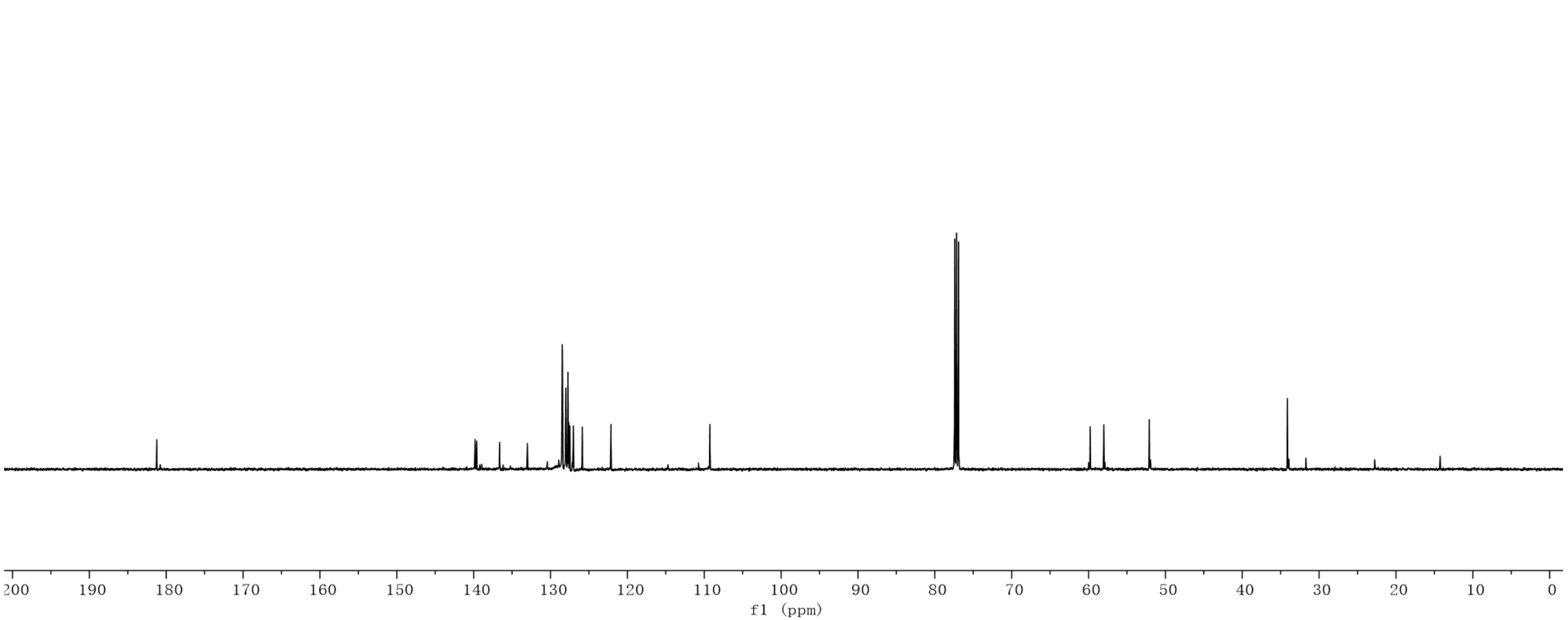

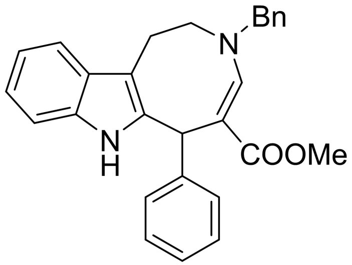

6

(600 MHz, $CDCl_3$)

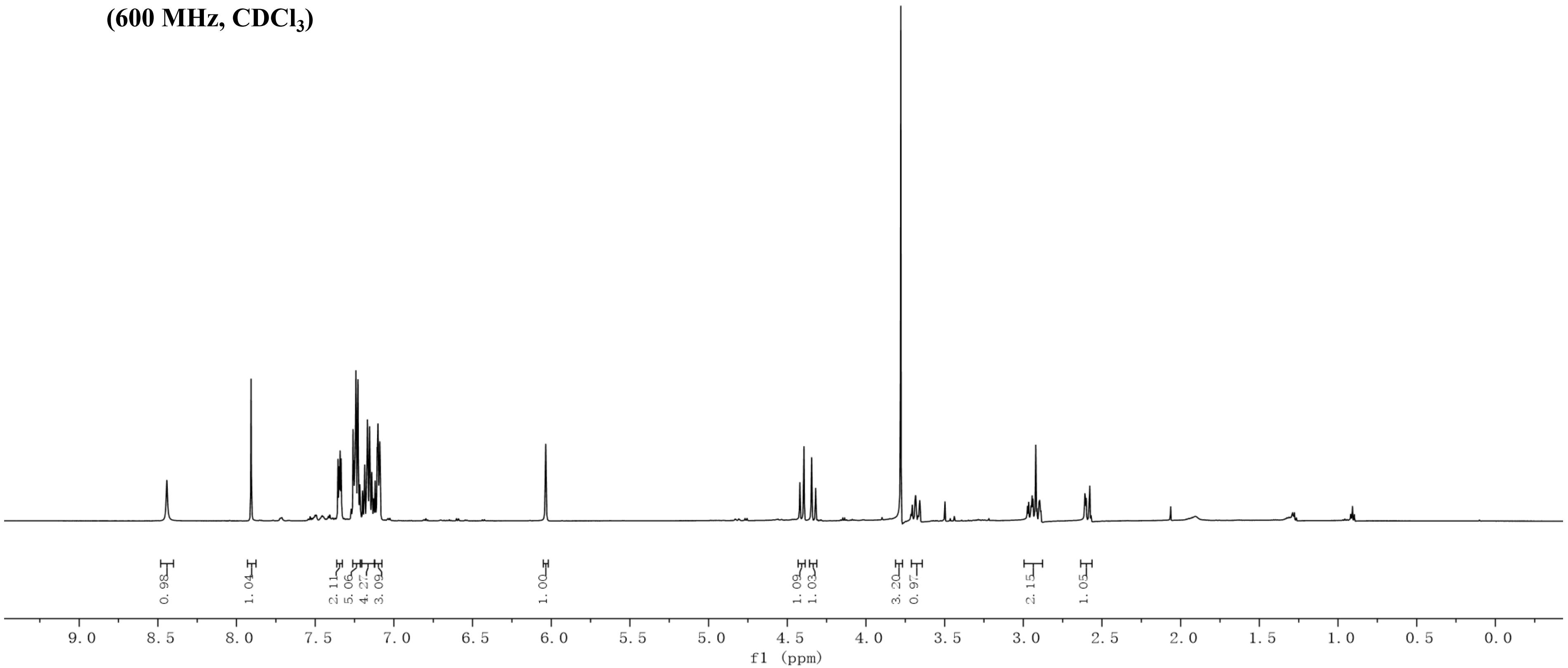

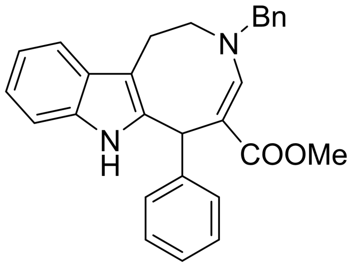

6

(151 MHz, $CDCl_3$)

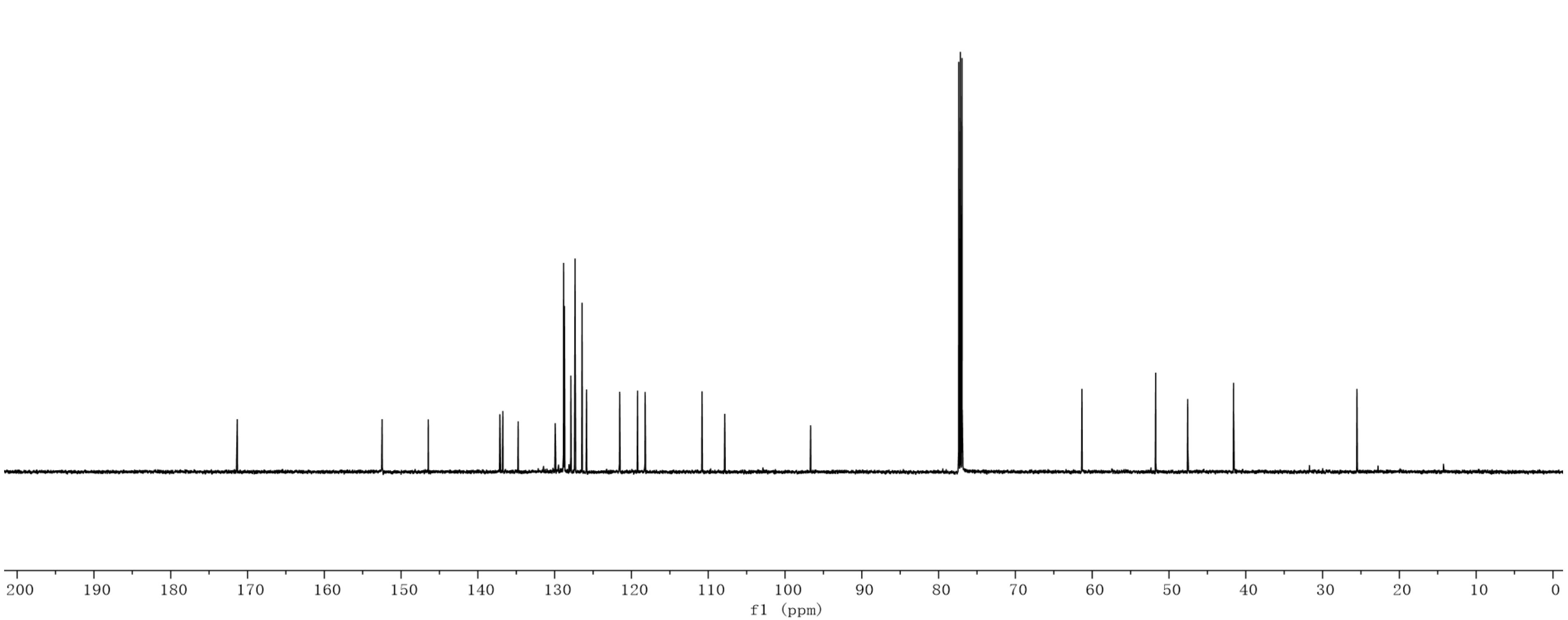

Supplement: Supplementary file 1 [file ol6c01466_si_001.pdf]
